# DNA copy number analysis of fresh and formalin-fixed specimens by shallow whole-genome sequencing with identification and exclusion of problematic regions in the genome assembly

**DOI:** 10.1101/gr.175141.114

**Published:** 2014-12

**Authors:** Ilari Scheinin, Daoud Sie, Henrik Bengtsson, Mark A. van de Wiel, Adam B. Olshen, Hinke F. van Thuijl, Hendrik F. van Essen, Paul P. Eijk, François Rustenburg, Gerrit A. Meijer, Jaap C. Reijneveld, Pieter Wesseling, Daniel Pinkel, Donna G. Albertson, Bauke Ylstra

**Affiliations:** 1 Department of Pathology, VU University Medical Center, 1007 MB Amsterdam, The Netherlands;; 2 Department of Pathology, Haartman Institute and HUSLAB, FIN-00014 University of Helsinki and Helsinki University Central Hospital, Helsinki, Finland;; 3 Helen Diller Family Comprehensive Cancer Center, University of California San Francisco, San Francisco, California 94158, USA;; 4 Department of Epidemiology and Biostatistics, University of California San Francisco, San Francisco, California 94158, USA;; 5 Department of Epidemiology and Biostatistics, VU University Medical Center, 1007 MB Amsterdam, The Netherlands;; 6 Department of Mathematics, VU University, 1181 HV Amsterdam, The Netherlands;; 7 Department of Neurology, VU University Medical Center, 1007 MB Amsterdam, The Netherlands;; 8 Department of Neurology, Academic Medical Centre, 1105 AZ Amsterdam, The Netherlands;; 9 Department of Pathology, Radboud University Medical Centre, 6500 HB Nijmegen, The Netherlands;; 10 Department of Laboratory Medicine, University of California San Francisco, San Francisco, California 94153, USA;; 11 Bluestone Center for Clinical Research, New York University College of Dentistry, New York, New York 10010-4086, USA

## Abstract

Detection of DNA copy number aberrations by shallow whole-genome sequencing (WGS) faces many challenges, including lack of completion and errors in the human reference genome, repetitive sequences, polymorphisms, variable sample quality, and biases in the sequencing procedures. Formalin-fixed paraffin-embedded (FFPE) archival material, the analysis of which is important for studies of cancer, presents particular analytical difficulties due to degradation of the DNA and frequent lack of matched reference samples. We present a robust, cost-effective WGS method for DNA copy number analysis that addresses these challenges more successfully than currently available procedures. In practice, very useful profiles can be obtained with ∼0.1× genome coverage. We improve on previous methods by first implementing a combined correction for sequence mappability and GC content, and second, by applying this procedure to sequence data from the 1000 Genomes Project in order to develop a blacklist of problematic genome regions. A small subset of these blacklisted regions was previously identified by ENCODE, but the vast majority are novel unappreciated problematic regions. Our procedures are implemented in a pipeline called QDNAseq. We have analyzed over 1000 samples, most of which were obtained from the fixed tissue archives of more than 25 institutions. We demonstrate that for most samples our sequencing and analysis procedures yield genome profiles with noise levels near the statistical limit imposed by read counting. The described procedures also provide better correction of artifacts introduced by low DNA quality than prior approaches and better copy number data than high-resolution microarrays at a substantially lower cost.

Alteration in chromosomal copy number is one of the main mechanisms by which cancerous cells acquire their hallmark characteristics (Pinkel et al. 1998; Hanahan and Weinberg 2011). For > 20 yr, these alterations have been routinely detected first by genome-wide comparative genomic hybridization (CGH) (Kallioniemi et al. 1992) and subsequently by array-based CGH (Snijders et al. 2001) or single nucleotide polymorphism (SNP) arrays (Ylstra et al. 2006). Now whole-genome sequencing (WGS) offers an alternative to microarrays for many genome analysis applications, including copy number detection.

Several methods have been developed to estimate DNA copy number from WGS data. They can be grouped into the following four categories, each of which has its own set of requirements, strengths, and weaknesses (Teo et al. 2012): (1) Assembly-based methods construct the genome piece by piece from the sequence reads instead of aligning them to a known reference; these methods have the greatest sensitivity to detect deviations from the reference genome, including copy number changes and genome rearrangements, but require high sequence coverage (typically 40×) (Li et al. 2010) and therefore incur high cost; (2) split-read and (3) read-pair methods map sequence reads from both ends of size-fractionated genomic DNA molecules onto the reference genome; these methods can provide information on copy number and genome rearrangements, but they impose requirements on molecule sizes and therefore are highly sensitive to DNA integrity; and (4) depth of coverage (DOC) methods infer copy number from the observed sequence depth across the genome and do not require both ends of the molecule to be sequenced.

Archival tissue is an invaluable resource for biomarker detection studies (Casparie et al. 2007). Projects investigating cancers with long survival, such as diffuse low-grade gliomas (LGGs) with a subset of patients surviving > 25 yr after diagnosis (van Thuijl et al. 2012), require long-term clinical follow-up. Archival FFPE tissue is often the only source of material for study (Blow 2007). The use of such samples has been challenging due to poor DNA quality; hence, array CGH results, for example, have been variable (Mc Sherry et al. 2007; Hostetter et al. 2010; Krijgsman et al. 2012; Warren et al. 2012). To make large archival sample series accessible for genome research, a robust technique is required that performs well on diverse sample types, with high resolution, quality and reproducibility, and at low cost without the necessity for a (matched) normal sample. Here we focus exclusively on DOC methods, because they are theoretically most compatible with DNA isolated from FFPE material.

Typically, DOC methods for copy number divide the reference genome into bins and count the number of reads in each, although there are also bin-free intensity-based implementations (Shen and Zhang 2012). Copy number is then inferred from the observed read counts across the genome. To compensate for technological bias, many DOC algorithms, such as CNV-seq (Xie and Tammi 2009), SegSeq (Chiang et al. 2009), BIC-seq (Xi et al. 2011), and CNAnorm (Gusnanto et al. 2012), compare tumor signal to a normal reference signal, similar to array CGH. Commonly, a pool of different individuals is used as a normal reference DNA. In many applications, including cancer genome analysis, matched normal DNA from the same patient is preferable to avoid detection of germline copy number variants (Feuk et al. 2006), allowing focus solely on somatic aberrations (Perry et al. 2008).

Two DOC methods, readDepth (Miller et al. 2011) and FREEC (Boeva et al. 2011), do not require a reference signal. This has three principal advantages: the cost is reduced by half, archival material for which matched normal reference tissue is unavailable (most cases) can be analyzed, and measurement noise from the reference sample is avoided. Achieving these benefits requires accurate computational correction for biases in the DOC sequence data since they are no longer being normalized by comparison with data from a matched reference specimen.

Here we describe a multiplexed, single-read (SR), shallow WGS procedure based on the Illumina platform that produces improved DOC copy number profiles. Because DOC profiles are fundamentally based on counting the number of sequence reads, the minimum achievable noise can be easily calculated. We show that a larger proportion (most) of the samples we have analyzed with our procedures show noise levels at the theoretical minimum than with other analysis methods. We achieve the improved performance by simultaneous (rather than sequential) correction of primary read counts for sequence mappability and GC content, and by using a comprehensive empirical approach for recognition and filtering of problematic genome regions. We also show that compared to previous shallow WGS analysis procedures, our approach provides improved correction of spurious localized profile variations, which are presumably due to sample quality problems; and microarray analysis costs more and yields a poorer signal-to-noise ratio than shallow WGS. Thus our DOC profiles provide a more accurate representation of the genome copy number structure than can be obtained by other approaches and should allow segmentation and calling algorithms to more sensitively recognize true aberrations.

## Results

### Shallow WGS and alignment to the reference genome

Shallow WGS was performed with DNA isolated from FFPE sections of 15 LGGs (van Thuijl et al. 2014), two oral squamous cell carcinomas (SCCs AB042 and AB052) (Bhattacharya et al. 2011), and the breast cancer cell line BT474 on the Illumina HiSeq 2000 using run mode SR50, which sequences only one end of the DNA molecules for 50 base pairs. In general, these DNA samples were multiplexed with others so that each HiSeq sequencing lane contained between 18 and 22 total samples. We use sample LGG150 to illustrate our analysis procedures in the main article text and figures because it contains a range of different types of genome alterations that are typical for solid tumors. Complete analyses of all LGG samples, BT474, AB042, and AB052, including whole-genome plots and enlarged views of chromosome 1, are presented in Supplemental Figures S1–S3. In addition, we present noise data from more than 1000 mostly formalin-fixed archival specimens obtained from many hospitals throughout Europe.

On average, we obtained 9.2 million total reads per sample (range 3.1–23.9) for the multiplexed samples, of which 8.2 million (range 3.0–22.9) aligned to the human reference genome with the sequence alignment algorithm BWA (Li and Durbin 2009). We filtered out PCR duplicate reads and reads with mapping qualities lower than 37 (highest value returned by BWA), resulting in a final average read count of 6.0 million (range 2.4–18.1) per sample. Read counts for the 15 LGGs, AB042, AB052, and BT474 are provided in Supplemental Table S1.

### Binning of sequence reads

**Figure 1.**
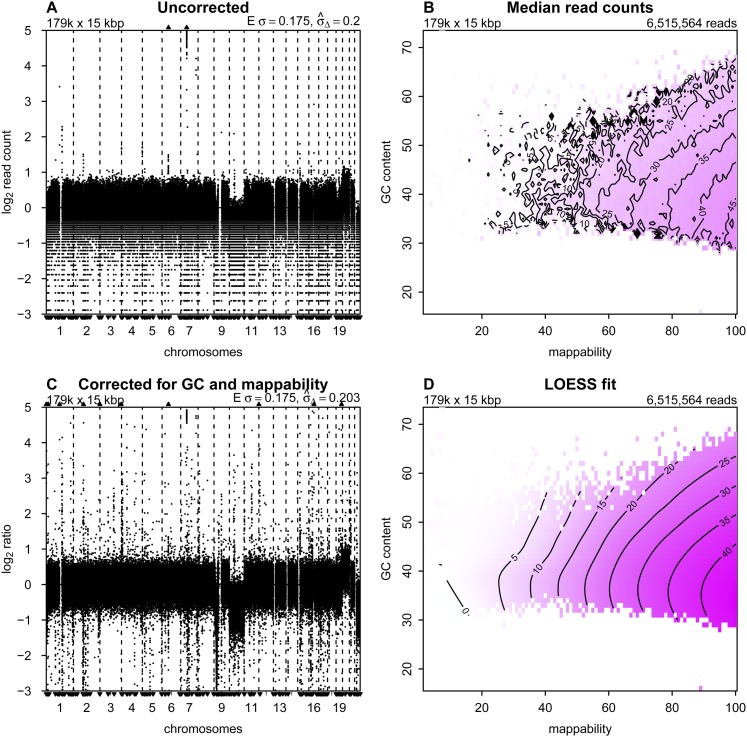
Correction to read counts. Copy number profiles from (*A*) uncorrected and (*C*) corrected read counts; (*B*) median read counts per bin as a function of GC content and mappability; and (*D*) the corresponding LOESS fit for sample LGG150. Regions of the isobar plots that are white contain no bins with that combination of GC and mappability. In the copy number profiles, bins are ordered along the *x*-axis by their genomic positions, and the *y*-axis shows median-normalized log_2_-transformed data. Small triangles at the *top* and *bottom* edges represent data points that fall outside the plot area. *Upper left* corners show the number and size of bins. *Upper right* corners of the median read counts plot shows the total number of sequence reads, and *upper right* corners of the copy number profiles the expected and measured standard deviation. The expected standard deviation (E *σ*) is defined as 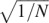, where *N* is the average number of reads per bin. The measured standard deviation 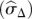 is calculated from the data with a mean-scaled and 0.1%-trimmed first-order estimate, prior to log_2_ transforming the data for plotting (see text).

We divided the human reference genome into nonoverlapping, fixed-sized bins. We use 15-kb bins in the analysis presented here because this results in approximately the same number of bins as the number of array elements on 180K oligonucleotide CGH arrays and provides reasonable noise levels with as few as 6 million reads. We note, however, that any bin size could be used, and such an option is provided in the accompanying software package, QDNAseq. Removal of 12,893 bins that were completely composed of uncharacterized bases (denoted with N’s in the human reference genome sequence) resulted in a total number of 179,187 autosomal bins. We determined raw copy number estimates by counting the number of reads in each bin. The median-normalized log_2_-transformed read counts, the raw copy number profile, for sample LGG150 is shown in Figure 1A. Regions of low-level loss and gain (e.g., on chromosomes 10 and 20, respectively) are apparent in the profile. In addition, some very narrow regions of highly elevated read counts and a substantial number of bins with very low read counts are present. The horizontal stripes of data points are due to the integer nature of the read counts. Experience based on classical cytogenetics and array CGH suggests that many features of this profile reflect characteristics of the sequencing and analysis process rather than true copy number variation (Baldwin et al. 2008).

### Correction of read counts

It is well established that raw read counts are affected by GC content and mappability of the sequence reads (Benjamini and Speed 2012; Derrien et al. 2012; Rieber et al. 2013). Published analysis methods generally correct for these factors independently if corrections for both are used. Although independent correction is effective for many cases, genome profiles from some samples, especially those that are formalin-fixed, contain clearly artifactual variations. Independent correction for GC and mappability is appropriate only if these two factors do not interact in their effects on read counts. We desired to determine if simultaneous correction might provide improved read count profiles. We implemented simultaneous correction by calculating the median read count for all bins with the same combinations of GC and mappability (Fig. 1B). We then fit a LOESS surface through the medians (Fig. 1D). To correct the raw read count of a bin, we divided the raw count by the LOESS value of its combination of GC and mappability. (Fitting the LOESS has the benefit of stabilizing the values for bins with closely related GC and mappability.) Following this procedure, the corrected profile for LGG150, after log_2_-transformation and centering, is much cleaner (Fig. 1C) than before correction. The correction of bins with low counts is particularly noticeable, but at the cost of introducing bins with read counts that appear to be anomalously high. Copy number profiles and plots of the median read counts as a function of GC content and mappability are shown for the 15 LGGs, AB042, AB052, and BT474 in Supplemental Figures S1 and S2.

### Blacklisting bins to exclude problematic regions

Examination of Figure 1C shows the presence of multiple very narrow peaks and some apparent deletions that might indicate aberrations. Some of these structures, for example many of the narrow peaks, appear to have been introduced by the GC-mappability correction. Many of these features are highly recurrent across, both tumor and normal, samples (data not shown). Recurrence alone may imply that these peaks represent common germline copy number variations (CNVs). The observation that they are frequently located in (peri-)centromeric and (sub-)telomeric regions, however, suggested that a large number are artifacts.

**Figure 2.**
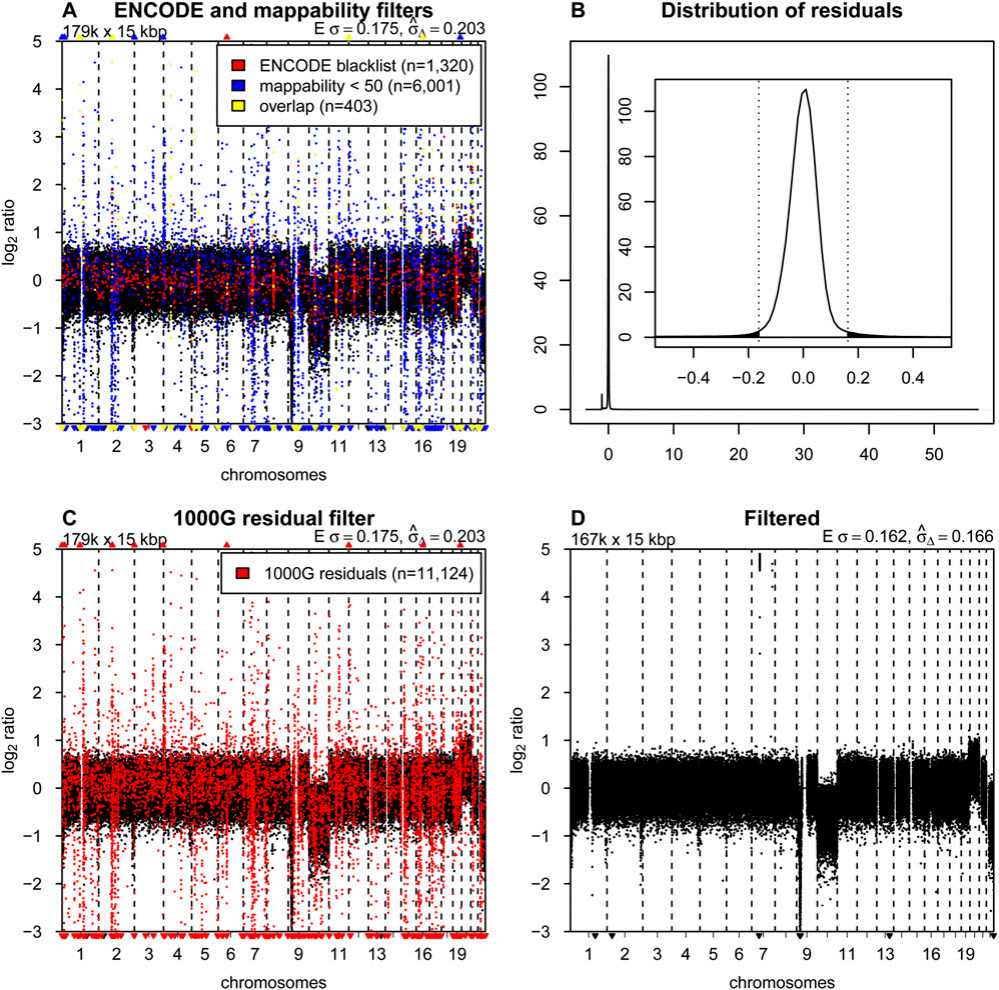
Blacklisting problematic regions. (*A*) Copy number profile for sample LGG150 with bins overlapping with the ENCODE blacklist highlighted in red, bins with mappabilities below 50 highlighted in blue, and the overlap between the two in yellow. (*B*) Distribution of median residuals per bin from the 1000 Genomes Project across the 38 samples. Residuals are defined as the distance between observed read counts and the fitted LOESS surface, divided by the LOESS value. The *outer* plot shows the entire range of values with two discrete peaks. The minor peak around −1.0 results from repetitive sequences. Reads that align equally well to multiple locations in the genome are filtered out. Repetitive sequences therefore have a lower than expected number of reads mapped. The major peak around zero contains most of the bins, and the *inset* shows a magnification of the peak, with the dotted vertical bars and the shaded area showing the cutoff of 4.0 standard deviations (as estimated with a robust first-order estimator) for blacklisting. (*C*) Copy number profile of sample LGG150 with bins in the novel blacklist based on residuals of the 1000 Genomes samples highlighted in red. (*D*) The final copy number profile of sample LGG150 after filtering out bins in the ENCODE and 1000G blacklists.

The presence of chromosomal regions with anomalous behavior is well established and has led others, for example, the ENCODE Project Consortium, to develop blacklists of sequences to exclude from their analyses (The ENCODE Project Consortium 2012). Some of these sequences map to regions with known repeat elements, such as satellites, centromeric, and telomeric repeats. Therefore we tested the effect of removing bins with mappabilities below the arbitrary threshold of 50 and bins overlapping with the ENCODE blacklists (Fig. 2A). Clearly, the profiles are improved, but many regions of potentially artifactual variation remain (indicated by black dots in Fig. 2A). Changing the mappability threshold affects the results to some degree but fails to sufficiently remove the problematic regions without also removing a major proportion of the bins (see Supplemental Fig. S4).

Given the insufficiency of the ENCODE blacklist for copy number analysis and the apparent recurrence of the problematic regions, we developed our own data-driven list of problematic genome regions. We started by analysis of a collection of normal genomes, which has the potential to identify problematic sequence motifs as in ENCODE, unknown problems in the reference genome sequence, and common CNVs. We obtained the required sequence data from the publicly available WGS data set from the 1000 Genomes Project (1000G) (The 1000 Genomes Project Consortium 2012). After selecting samples that were sequenced in a manner similar to our experimental setup (Illumina platform, low-coverage, SR50), we identified and downloaded 38 cases. The individuals have a substantial range of ethnic backgrounds (nine CEU, eight JPT, seven YRI, five CHB, three ASW, two PUR, one CLM, one IBS, one LWK, and one MXL).

The 38 samples were then processed as described above. The difference between the actual count and the LOESS fitted value was determined for each bin based on its GC and mappability values. These residuals were recorded for each sample, and the median of the residuals across the 38 samples was calculated per bin. The distribution of the median residual values is sharply peaked, which reflects the fact that normal diploid samples are being analyzed, but has “fat” tails, representing bins with anomalous behavior and those with CNVs (Fig. 2B). We chose to blacklist all bins with median residuals greater than 4.0 standard deviations, using a robust first-order estimator (von Neumann et al. 1941) that focuses on the width of the central peak to determine the standard deviation. This procedure removed 10,413 bins. We based our choice of the cutoff on the distributions of residuals found with a number of different bin sizes ranging from 1 to 1000 kb (Supplemental Fig. S5). The cutoff can be adjusted in the QDNAseq package if other values are desired. Changing it by one standard deviation in either direction, however, does not materially affect the results.

We were concerned that the initial presence of bins with high residuals, which were candidates for blacklisting, had the potential to affect the LOESS fit of the initial read counts. Therefore, we implemented an iterative process, recalculating the LOESS correction after removal of the problematic bins found in the previous cycle and again determining the residual distribution. Bins with residuals greater than the same numerical cutoff values established in the first iteration were removed. The list of excluded bins, our blacklist, stabilized at 11,124 bins after 14 iterations. Figure 2C shows the profile of LGG150 with our blacklisted bins highlighted. This blacklist contains many bins not included in the ENCODE list and also includes 97% (6200 of 6404) of the bins with mappabilities below 50. Overlaps between our blacklist, the ENCODE list, and bins with mappabilities below 50 are presented in Supplemental Figure S6. We intentionally were not conservative in blacklisting, since the copy number of a blacklisted locus can be imputed from neighboring bins (assuming no very focal aberrations are present), and most analytical packages handle this imputation automatically (van de Wiel et al. 2011).

For analysis of experimental samples, we routinely remove bins contained in the union of the ENCODE blacklist and our 1000 Genomes-based list at the beginning of the analysis so that their anomalous values do not affect the LOESS GC-mappability fit; although in practice the procedure seems to be fairly robust to the presence of these outliers. Similarly, the LOESS fit could be affected by copy number aberrations present in the data. Therefore, our software allows the correction described in the previous section to be implemented iteratively. After the initial analysis, bins with large LOESS residuals, which presumably are located in copy number aberrations, are excluded and the analysis is repeated. This cycle is iterated until the list of bins that are used stabilizes. We found this approach to be of little benefit in most cases, and the data presented in this paper have been corrected without this iterative step.

In total, this procedure removed 12,278 of the 15-kb bins (6.9%). Together with the 12,893 bins that consist of only uncharacterized nucleotides (N’s in the reference genome sequence), they form 954 separate continuous regions, which are listed in Supplemental Table S2. We also list the 2273 genes that fall within these regions, which thus includes genes in common germ-line CNVs. Figure 2D shows the final profile of sample LGG150 with the blacklist filtering and GC-mappability correction applied. Whole-chromosome losses can be seen involving chromosomes 10 and 22, and a gain of 20. A focal amplification is also present on chromosome 7, as well as a homozygous deletion on 9p. Final profiles for all LGG samples, BT474, AB042, and AB052 are shown in Supplemental Figures S1–S3.

### Noise and detection limits

Noise in copy number profiles has contributions from the statistics of counting sequence reads as well as the many steps in the analytical chain from sample acquisition and fixation through DNA isolation, sequencing, and computational processing. Since the variances of independent noise sources are additive, it is convenient to use the variances of the profiles to investigate their noise characteristics. Profiles normalized so that the mean value is 1.0 have variances due to counting statistics equal to 1/*N*, where *N* is the average number of reads per bin (neglecting small effects due to copy number aberrations and the counting corrections). Thus, the difference between the variance of the copy number profile and the variance due to counting statistics (1/*N*) for that profile gives a measure of the noise contribution from the entire sample handling and analytical process, independent of sequence depth. Therefore we examined the dependence of the variances of our profiles on sequence depth.

**Figure 3.**
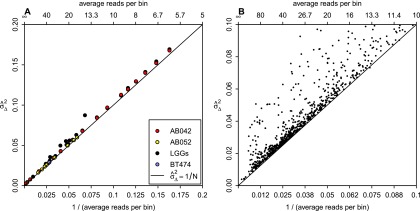
Dependence of variance on sequence depth. (*A*) The relationship between sequence depth and variance 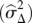 for 15 LGGs (black), cell line BT474 (blue), 10 independent library preparations of SCC sample AB052 (yellow), and subsamplings of AB042 data (red). All individual samples are within the *left* half of the graph, with the subsamplings extending to the *right* half as well. The black line shows the linear expectation of the variance as 1/*N*, where *N* is the average number of reads per bin. Lines fitted through the AB042 subsamplings and AB052 repeats have slopes of 1.026 and 1.003, and intercepts of 0.00107 and 0.000781, respectively. (*B*) The relationship between sequence depth and variance for more than a thousand samples sequenced at our institute.

We first tested the dependence of the variance on read depth alone by subsampling reads from a single data set with 108.4 million mapped reads of sample AB042. The subsampled data ranged over a factor of 100, from about 600 to 6 reads per bin. We performed the subsampling five times at each subsampling level and calculated the variances using a mean-scaled and 0.1%-trimmed first-order estimator. This estimator emphasizes bin-to-bin variation so that it is not affected by copy number aberrations (see Methods). Figure 3A shows the variance of the subsampled data for AB042 versus the variance due to counting statistics (1/*N*). A regression line fitted to the subsampled data has a slope of 1.026 and intercept of 0.00107, very close to the theoretical 1/*N* counting statistics (slope = 1; intercept = 0). The similar behavior of the measured and theoretical slopes indicates that variance versus read depth behaves essentially as expected. The fact that the intercept of the fitted regression line is close to zero indicates that the noise introduced from the sample quality and the analytical process is negligible. Thus, the noise is dominated by counting statistics at the read depths typical for shallow WGS analysis (30 reads per bin). [We note that copy number profiles are typically log_2_-transformed in our figures. If the variance were to be calculated based on the transformed profile, the contribution due to read depth would be log_2_(e)^2^/*N* ≈ 2.08/*N*].

We also examined the noise introduced by the library preparation and sequencing procedures by performing 10 independent sequencing runs from one DNA isolation of sample AB052. The variances of these profiles are also plotted in Figure 3A. The slope and intercept of the regression line are 1.003 and 0.000781, respectively. Thus, the total variance is again very close to the variance due to counting statistics (1/*N*), indicating that the library preparation and sequencing procedures have an insignificant contribution to the total variance. Further, the profiles from most of the LGG samples and the cell line BT474, which represent completely independent samples, also had variances very close to the theoretical counting statistics limit (Fig. 3A). Thus, DNA samples from a range of specimens obtained from our laboratories provided near optimal data using this measure.

Importantly, the variance characteristics shown in this small set of examples are generally representative of our experience with a large body of clinical specimens from many sources. We have now analyzed over a thousand samples obtained from more than 25 hospitals in five countries, mostly from FFPE tissues. A minority consisted of snap-frozen tissue samples or DNA extracted from cells freshly obtained from peripheral blood, sputum, swabs of the oral mucosa, or cancer cell lines. The samples represented a wide spectrum of neoplasms, mainly carcinomas, but also neuroectodermal and mesenchymal neoplasms, as well as non-neoplastic tissues and cells, generally submitted for detection of somatic aberrations. In most cases, DNAs were isolated in the laboratories that provided the specimens, using their local protocols. Samples were sequenced in pools of ∼20 per lane. Figure 3B shows the variances of the resulting profiles versus 1/*N* for these samples. This figure shows that for the vast majority of samples, the overall variance in our profiles is dominated by the read depth. In our experience, profiles with a variance corresponding to greater than 30 reads per bin, around 6 million total reads for 15-kb bins (∼0.1× sequence coverage), are suitable for most subsequent analyses. Because the noise is dominated by counting statistics for most samples, it is possible to make instructive estimates of the smallest aberrations that can be detected as a function of read depth. In Supplemental Figure S7, we present estimates for gain and loss as well as a simple analytical formula applicable for a wide range of situations.

We note that some samples have variances clearly above the 1/*N* line (Fig. 3B). New sample preparation and analysis of several of these excessively noisy samples indicates that the noise is reproducible, both in magnitude and in shape along the genome, suggesting that it has its origin in the sample. Most likely it is due to degraded/damaged DNA resulting from the fixation and storage. Increasing sequence depth will not reduce this noise relative to the (1/*N*) line for variance due to counting statistics.

### The software package QDNAseq

The software package QDNAseq was developed to implement the novel profile correction and blacklisting approach described above and to perform downstream segmentation and calling of aberrations using well established software tools. QDNAseq uses BAM files as input because they are produced by the commonly used alignment algorithms such as BWA (Li and Durbin 2009). The program is implemented in R (R Core Team 2014) and is available in Bioconductor (Gentleman et al. 2004). Detailed information concerning its operation is included in the Bioconductor vignette. Briefly, bin size, LOESS parameters, and blacklisting parameters are adjustable. Blacklisted bins can be visualized, as in Figure 2, A and C. Options are to either filter out bins overlapping with the ENCODE blacklist (1723 bins when using the 15-kb bin size) and/or the blacklist we developed from the 1000G data (11,124 bins). A key feature of QDNAseq is the use of fixed-sized bins, which is necessary for most published downstream procedures that handle series of tumor samples (van de Wiel et al. 2011). Use of fixed-sized bins furthermore allows calculation of annotation data (GC content, mappability, overlap with ENCODE blacklist, 1000G residuals) in advance, facilitating computation and analysis procedures. Analysis is therefore relatively rapid. For example, processing of the LGG150 sample included in this paper takes 75 sec from the input BAM file to the filtered and corrected profile in Figure 2D on a standard workstation or laptop with a 2.3 GHz Intel Core i5 CPU. Included in the QDNAseq package is also an option to compare to matched reference samples should that be desired (see Supplemental Fig. S8).

The output of QDNAseq is read counts per bin, which have been corrected, filtered, normalized, and optionally log_2_-transformed. QDNAseq was built in a modular fashion such that analysis tools and pipelines for downstream segmentation and copy number calling previously developed for microarrays (for review, see van de Wiel et al. 2011), for example, can be readily applied. Downstream analysis can also be performed and was tested with the commercially available software suite Nexus Copy Number (BioDiscovery). QDNAseq has also been made available in Chipster (Kallio et al. 2011) and Galaxy (Goecks et al. 2010) and allows export of the copy number results into the Integrative Genomics Viewer (IGV) (Thorvaldsdóttir et al. 2013). The popular segmentation package DNAcopy (Venkatraman and Olshen 2007) can be invoked directly from within QDNAseq. In addition to the existing user-definable parameters available in DNAcopy, an option to smooth signals over a specified number of consecutive bins has been added in QDNAseq. For calling (annotation of segments with copy number states such as gain, amplification, or loss), the package CGHcall (van de Wiel et al. 2007) can be invoked at the user's discretion.

### Comparison to other algorithms and array CGH

Multiple algorithms have been developed for DOC DNA copy number analysis. Most compare the tumor sample to a reference signal and thus require acquisition of an appropriate reference sample and additional sequencing. Two algorithms have been published for analysis of shallow WGS that do not require a reference signal, readDepth (Miller et al. 2011) and FREEC (Boeva et al. 2011). Both adjust read counts and/or filter out bins based on GC content and mappability, but lack other blacklisting options such as those based on ENCODE or the 1000 Genomes-based blacklist. Both have integrated segmentation and calling to identify gains and losses. Since the novel aspects of QDNAseq occur in the determination of the filtered and corrected read count profile, we opted to evaluate the performance of QDNAseq relative to the preprocessing parts of these other analysis packages. However, readDepth does not output bin-level data so we could only compare our results with FREEC. A third program, CLImAT, was recently published which, among other things, infers copy number from the observed sequence depth without requiring a reference signal (Yu et al. 2014). The goal of this program, however, is to use relatively deep (10× genome coverage) sequencing to obtain information that is not available from a small number of reads (0.1× genome coverage), which is the focus of our work. The CLImAT algorithm uses a simpler form of simultaneous GC and mappability correction that is likely to be too noisy at our read depth, so we did not evaluate it.

**Figure 4.**
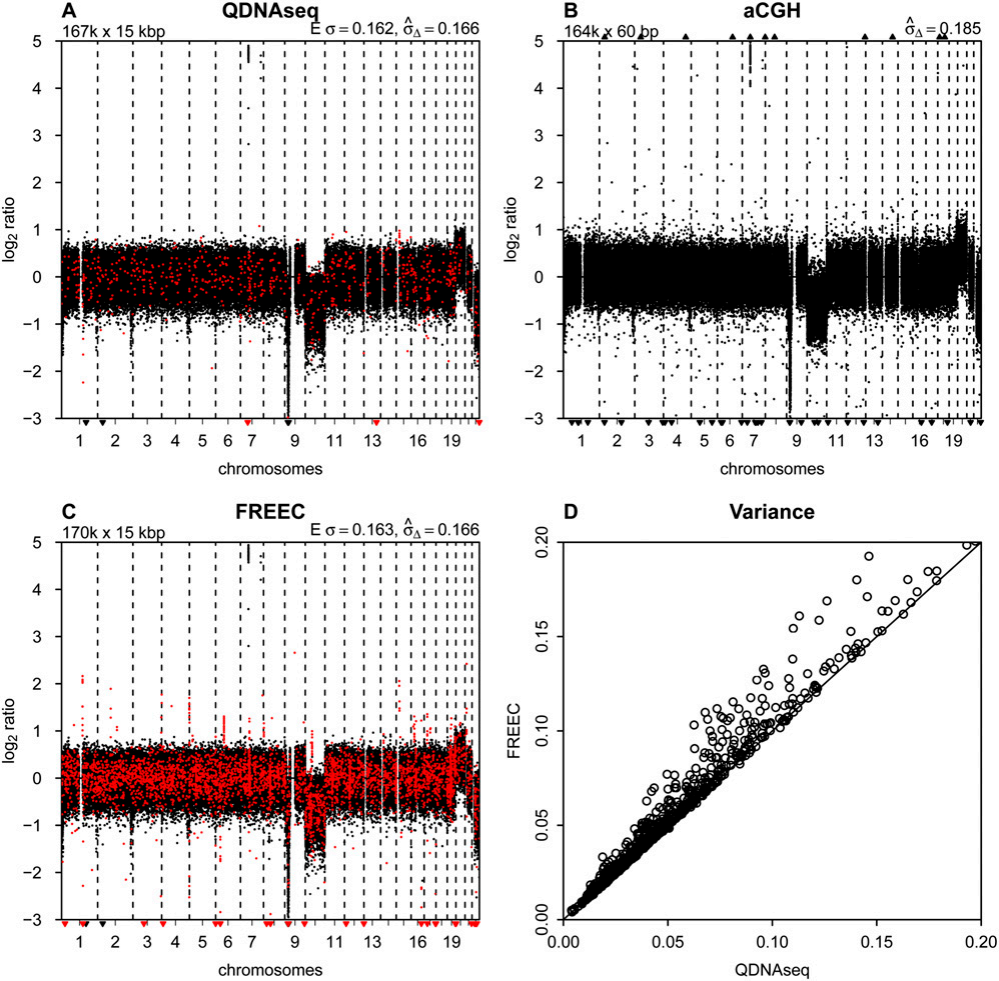
Comparison to other methods. (*A*) Final copy number profile of sample LGG150 obtained with QDNAseq after removing blacklisted bins and correcting read counts for GC content and mappability. This procedure results in 166,909 bins, and highlighted in red are those 750 bins that are not contained in the output of FREEC. (*B*) Copy number profile of sample LGG150 obtained with an Agilent 180K microarray with 164,378 unique array elements. (*C*) Copy number profile of sample LGG150 obtained with FREEC with 170,474 bins. Highlighted in red are those 4315 bins that are not contained in the output of QDNAseq. Note that many of the red bins are in focal peaks that have the potential of being called aberrations but which are probably spurious since they are contained in the QDNAseq blacklists. (*D*) Noise 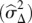 for QDNAseq versus FREEC calculated from the thousand samples in Figure 3B. Only the 166,159 bins present in the output of both algorithms were used in order to eliminate differences caused by blacklisting spurious bins.

Both QDNAseq and FREEC perform better than the Agilent array CGH platform at the sequencing depths used here. Figure 4, A, B, and C shows the profiles of sample LGG150 obtained with QDNAseq, array CGH, and FREEC, respectively. The data from QDNAseq and FREEC are very similar in their calculated noise, but FREEC contains several focal apparent gains and losses that are not present in the QDNAseq data due to blacklist filtering. These artifactual features in FREEC output are at risk of being interpreted as true aberrations. Array CGH has greater noise and more outliers than with both sequencing analyses, with a standard deviation of 0.19 compared to 0.17 for sequencing analyses. Moreover, the deflections for the copy number changes are larger for the sequencing methods than for array CGH. The average signal-to-noise ratio (SNR) for 12 whole-chromosome aberrations among the 15 LGG samples was 1.89, 1.91, and 1.40 for QDNAseq, FREEC, and aCGH, respectively (Supplemental Table S3).

Comparison of data obtained with QDNAseq and FREEC on the entire set of ∼1000 samples shows that the variance with FREEC was never lower than with QDNAseq and often somewhat higher (Fig. 4D). To assure that the comparison concentrates on differences in read count corrections and not the filtering of blacklisted bins, the variance calculations were performed only on the set of bins contained in the output of both programs. This shows that the simultaneous correction for GC content and mappability implemented in QDNAseq always performs at least as well as the sequential corrections in FREEC and is better for some samples.

Simultaneous correction for GC content and mappability outperforms separate corrections for cases in which the two parameters interact. The interaction can be seen from examination of the LOESS surfaces for the various samples. For sample LGG150 presented in Figure 1, read counts always increase with increasing mappability regardless of GC content. Thus to a reasonable approximation there is minimal interaction. In contrast, samples LGG155 and LGG259 both have read count maxima along the mappability-axis that vary with GC content (Supplemental Figs. S1, S2). Consequently, a single correction curve for GC that is applied to all mappabilities, and vice versa, will not properly correct these samples.

The major benefit of our simultaneous correction approach is seen in the removal of spurious regions of variation in the profiles. Supplemental Figure S9 shows profiles for the whole genome and for chromosome 1 generated from the same sequencing data by QDNAseq and FREEC for all 15 LGG samples, two SCCs, and BT474. Examination of the profiles for LGG151 and LGG155 shows clearly that small features in the profiles produced by FREEC are corrected by QDNAseq. Similar features also occur on other chromosomes. Improved correction of this sample-related variability allows use of more sensitive segmentation and calling procedures for a given level of false positives. Three of the 15 LGG samples showed significant improvement using QDNAseq. Thus our correction procedure facilitates correct biological interpretations from samples with a wider range of quality.

## Discussion

We have described a shallow WGS procedure designed to obtain high-quality DNA copy number information from fresh and archival samples. The method was developed in the process of analyzing over a thousand tumor DNA samples obtained from more than 25 hospitals in five countries, mostly from FFPE tissue. Our goal was to provide the best possible read count profiles so that subsequent segmentation and calling steps would be able to sensitively detect true aberrations at acceptable levels of false positives. The data presented show that our corrected profiles have noise levels very near the fundamental limit imposed by the statistics of read counting for most samples, and are less sensitive to DNA quality induced artifacts than profiles produced by prior approaches. The predictable nature of the major noise source of our read count profiles represent a considerable interpretive simplification compared to microarray DNA copy number profiles, in which the noise sources are obscure and copy number changes are frequently reduced in magnitude due to array performance (Snijders et al. 2001; Ylstra et al. 2006).

Our procedure contains two novel features: simultaneous correction of counts for GC content and mappability, and empirical recognition of problematic regions of the genome based on analysis of a group of normal samples. Here, we demonstrate the performance of QDNAseq on 1000 samples, mostly from archival FFPE cases. The simultaneous correction for GC and mappability, using a LOESS fit of the raw count data to the average values of these parameters for each sequencing bin, always performs at least as well, and in more degraded DNA samples better, than the separate corrections that are used by most existing algorithms. Nevertheless, it is also evident that our correction remains inadequate for some samples. It is likely that a more thorough understanding of the impact of formalin fixation and the distribution of base composition and mappabilities within the sequencing bins will result in improved ability to obtain useful copy number data from samples that remain problematic. Further, although our blacklist was developed from 38 normal samples representing a variety of ethnicities from the 1000 Genomes Project, similarly derived blacklists tailored to the ethnicity of the population from which the samples were obtained would allow more precise blacklisting of common germ-line CNVs relevant for that population.

Shallow WGS is cost effective. Our experience indicates that high quality DNA copy number information can be obtained with ∼6 million reads per sample (∼0.1× genome coverage). High-capacity instruments such as the Illumina HiSeq can obtain this read depth with a multiplex analysis of 20 samples per lane. We achieved a further increase in efficiency by sequencing only 50 bp from one end of the DNA molecules, which also allows the use of compromised samples with short DNA fragments. This level of sequence depth provides better resolution than is available from microarrays and costs significantly less. Shallow sequencing has a particular cost benefit if combined with exome sequencing because the initial preparation for both is the same. The additional cost of the shallow sequence run is marginal (∼5% extra) and provides high-resolution genome-wide copy number information. If the shallow sequencing run is performed prior to exome enrichment and the exome sequencing run, it can also serve as the ultimate quality control. Although we obtained most of our data on an Illumina HiSeq instrument, the use of smaller capacity sequencers, such as the Illumina MiSeq, offer rapid turnaround and have the required capacity for relatively infrequently submitted diagnostic samples.

## Methods

### Sample selection

Fifteen LGGs (van Thuijl et al. 2014), two SCCs (Bhattacharya et al. 2011), and the breast cancer cell line BT474 were used to develop and illustrate the shallow WGS pipeline presented. All material used from LGG and SCC tumors was derived from FFPE archival samples. Patient consent was obtained for SCCs as published previously (Bhattacharya et al. 2011). LGG samples were collected from five Dutch hospitals (VU University Medical Center in Amsterdam, Academic Medical Center in Amsterdam, Radboud University Medical Center in Nijmegen, St. Elisabeth Hospital in Tilburg, and Isala Klinieken in Zwolle). Sample collection was approved by the Medical Ethics Committees of all five hospitals. Areas containing > 60% tumor cells were outlined on hematoxylin and eosin-stained slides, and 10 subsequent 10-μm sections were used for DNA isolations.

DNA from the LGG samples was isolated as previously described (van Essen and Ylstra 2012). DNA concentrations were measured with the Nanodrop 2000 (Fisher Scientific), and 500 ng was used as input for Shallow WGS laboratory preparation. DNA from the SCC samples was isolated as previously described (Bhattacharya et al. 2011), DNA concentrations were measured with the Qubit 2.0 fluorometer dsDNA BR Assay (Life Technologies), and 250 ng DNA used as input for shallow WGS laboratory preparation. The BT474 breast tumor cell line was cultured and DNA isolated as previously described (Krijgsman et al. 2013). DNA concentration was measured with the Qubit fluorometer and 250 ng used as input.

### Shallow WGS laboratory preparation

DNA was sheared on a Covaris S2 (Covaris) with the following settings: duty cycle 10%, intensity 5.0, bursts per sec 200, duration 240 sec (FFPE), duration 300 sec (fresh and fresh-frozen), mode frequency sweeping, power 23V, temperature 5.5°C to 6°C, water level 15. Sample preparation was then performed with the TruSeq DNA kit V2 (Illumina). After end repair and 3′ adenylation, adapter ligation was performed with 1 μL of adapter index for fresh (frozen) samples and 0.55 μL of adapter index for FFPE samples. Final sequence library amplification was performed with 10 PCR cycles for FFPE derived DNA samples or eight cycles for DNA derived from fresh or fresh-frozen samples. One PCR cycle included 10 sec 98°C, 30 sec 60°C, and 30 sec 72°C. The PCR program started with 30 sec 98°C and ended with 5 min 72°C. The final holding temperature was 10°C.

The yield of the sequence library was assessed with a Bioanalyzer DNA 1000 and/or HS DNA (Agilent Technologies). Libraries with small PCR products (∼120 nt in length caused by unligated adapter dimers) or large PCR products (> 1000 nt in length caused by an exhausted PCR mix) were selected for cleaning. Cleaning was performed by using a double-sided bead size selection procedure with Agencourt AMPure XP beads (Beckman Coultier). Libraries were equimolarly pooled with 18–22 barcoded samples and 7 pM molarity loaded per lane of a HiSeq Single End Flowcell (Illumina). This was followed by cluster generation on a cBot (Illumina) and sequencing on a HiSeq 2000 (Illumina) in a single-read 50-cycle run mode (SR50).

### Alignment to reference genome

Sequence reads were aligned to the human reference genome build GRCh37/hg19 downloaded from Ensembl (Flicek et al. 2013) with BWA 0.5.9 (Li and Durbin 2009), with a maximum edit distance of 2 and base trimming quality of 40. PCR duplicates were marked with Picard 1.61 (http://broadinstitute.github.io/picard/), and filtered out with SAMtools 0.1.18 (Li et al. 2009) together with reads with mapping qualities (MAPQ) lower than 37. We note that the maximum possible value and the distribution of mapping qualities varies between aligners, and a different cutoff might be suitable for e.g., Bowtie (Langmead and Salzberg 2012), which was tested here but did not show an improvement over BWA for copy number assessment.

### Annotations for genomic bins

The genome was divided into nonoverlapping, fixed-sized bins of 15 kb. GC content of each bin was calculated as number of C and G nucleotides divided by number of A, C, G, and T nucleotides in the reference sequence. The percentage of characterized nucleotides was calculated by dividing the number of nucleotides A, C, G, and T with the bin size (15 kb). This is used to adjust read counts for bins partially covered by uncharacterized nucleotides (N’s) or incomplete bins at the very ends of chromosomes.

Mappability is a measure of the uniqueness of a specific sequence in the reference genome and depends on the length of the sequence and the number of mismatches allowed. If *F*_*k*_(*x*) is the frequency at which the *k-*mer sequence at position *x* is observed in the reference genome sequence and its reverse complement, the mappability of this position is defined as *M*_*k*_(*x*) = 1/*F*_*k*_(*x*). In this paper, we use the term mappability to refer to the average mappability of all 50-mer sequences within a bin, allowing for two mismatches, and scaling the value from 0 to 100. These values were calculated from the ENCODE alignability track for 50-mers (data version January 2010) with the *bigWigAverageOverBed* program downloaded from the UCSC Genome Browser (Kent et al. 2002; Rosenbloom et al. 2013).

The ENCODE blacklisted regions (March 2012 Freeze) were used to calculate percent overlap with each bin. Pregenerated bin annotations are available for human reference genome build GRCh37/hg19 and bin sizes of 1, 5, 10, 15, 30, 50, 100, 500, and 1000 kb.

### Binning and correction of read counts

The number of sequence reads in each bin was calculated. Read counts were adjusted for those 487 bins that are only partly covered by characterized nucleotides in the reference genome sequence. For a bin containing a proportion *r* of uncharacterized nucleotides, no reads could be mapped to this fraction of the bin, and the read count was therefore adjusted by dividing it by 1 − *r*.

Next, median read counts were calculated as a function of GC content and mappability. For this purpose, GC content and mappability values were rounded to integers (IEC 60559 standard). A two-dimensional LOESS surface was then fitted to the observed median read counts. The read count for each bin was then corrected by dividing it with the fitted LOESS value.

Optimal parameters for the LOESS correction were evaluated with an odd-even cross-validation as follows: Bins were divided into odd and even bins, and only odd ones were used to calculate the LOESS correction as above. The same correction was then applied to both odd and even bins, and the absolute values for differences in adjusted counts between adjacent odd and even bins were calculated. A test statistic was calculated as a trimmed mean of the absolute values after removal of the upper 10% to account for copy number breakpoints. Parameter values of span = 0.65 and family = ‘symmetric’ were chosen to minimize the value of the test statistic.

### 1000 Genomes residuals

The blacklist based on 1000 Genomes samples was generated as follows. Publicly available samples from the 1000 Genomes Project that matched the experimental setup were downloaded (Illumina single-read of at least 50 bp, low coverage, whole-genome sequencing). For samples with read lengths longer than 50 bp, the reads were truncated to the first 50 bp. In total, 38 samples that matched the experimental setup were available. Alignment and two-dimensional LOESS correction were then performed as outlined above. Residuals [(observed read count − LOESS fit)/LOESS fit] from the correction were recorded, and medians per bin were then calculated across the 38 samples. Cutoff for exclusion was set at 4.0 standard deviations (as estimated with a robust first-order estimator [von Neumann et al. 1941]). After bins exceeding this cutoff were removed, the LOESS correction was repeated without these anomalous bins and residuals calculated again. This process was repeated until the list of bins to be excluded stabilized.

### Noise, comparison to other algorithms, and array CGH

FREEC (version 6.4) (Boeva et al. 2011) was run with a bin size of 15 kb, mappability-based read count correction turned on, minimum mappability set to 50, and otherwise default settings. These settings were selected to mimic QDNAseq as closely as possible. As a measure of noise, we used an estimator based on first-order differences (von Neumann et al. 1941). This noise estimator is sensitive to uncorrelated bin-to-bin read count differences along the profile, but is largely unaffected by correlated behavior of groups of bins such as steps in the profile due to true copy number aberrations or long-range waviness. For robustness against large outliers, we excluded 0.1% of extreme values from both ends of the distribution. Unless specified otherwise, terms “standard deviation” and “variance” used in this paper refer to this mean-scaled 0.1%-trimmed first-order estimate and its square, respectively. They are calculated for a linear representation of the profiles even though we present log_2_-transformed profiles for display convenience. The standard deviation of a profile, denoted by 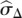, and the theoretically expected standard deviation based on read counting, denoted by E *σ*, are given above each profile.

All samples were profiled with CGH arrays that contained 180K in situ synthesized 60-mer oligonucleotides evenly distributed (every 17 kb) across the genome (Agilent Technologies). BT474 CGH arrays were performed previously (Krijgsman et al. 2013) and data downloaded from the GEO database (Edgar et al. 2002) with accession number GSM903069. Labeling, hybridization, scanning, and feature extraction were carried out as previously described (Krijgsman et al. 2013) with pooled normal reference samples. After median normalization, wave-correction was performed with NoWaves (van de Wiel et al. 2009), which would account for GC variation across the genome. The SCC samples have also been previously characterized by 2K BAC arrays which data are available in GEO with accession GSE28407 (Bhattacharya et al. 2011).

## Data access

QDNAseq package is available through Bioconductor (http://www.bioconductor.org/) (Gentleman et al. 2004). Source code is available in GitHub (https://github.com/ccagc/QDNAseq/), and for the version used to generate data presented in this paper, also in the Supplemental Material. Sequence and microarray data have been submitted to the European Genome-phenome Archive (EGA; http://www.ebi.ac.uk/ega/) (Leinonen et al. 2011) which is hosted at the EBI, under accession number EGAS00001000642.

## Supplementary Material

Supplemental Material
