# Supplementary material for: DNA copy number analysis of fresh and formalin-fixed specimens by shallow whole-genome sequencing with identification and exclusion of problematic regions in the genome assembly
Source: Genome Res. 2014 Dec;24(12):2022–32. doi: 10.1101/gr.175141.114 (PMC4248318; doi:10.1101/gr.175141.114)
Supplement: Supplemental Material [file supp_24_12_2022__index.html]

DNA copy number analysis of fresh and formalin-fixed specimens by shallow whole-genome sequencing with identification and exclusion of problematic regions in the genome assembly — DNA copy number analysis of fresh and formalin-fixed specimens by shallow whole-genome sequencing with identification and exclusion of problematic regions in the genome assembly — Supplemental Material 

# DNA copy number analysis of fresh and formalin-fixed specimens by shallow whole-genome sequencing with identification and exclusion of problematic regions in the genome assembly

## Supplemental Material

**Files in this Data Supplement:**

- Supplemental Figures.pdf
- Supplemental Material.zip
- Supplemental Tables.xlsx
