# Supplementary material for: DNA copy number analysis of fresh and formalin-fixed specimens by shallow whole-genome sequencing with identification and exclusion of problematic regions in the genome assembly
Source: Genome Res. 2014 Dec;24(12):2022–32. doi: 10.1101/gr.175141.114 (PMC4248318; doi:10.1101/gr.175141.114)
Supplement: Supplemental Material [file supp_gr.175141.114_Supplemental_Figures.pdf]

Supplementary Figures for

Scheinin and Sie et al.: DNA copy number analysis of fresh and formalin-fixed specimens by whole-genome sequencing with identification and exclusion of problematic regions in the genome assembly

September 11, 2014

Table of Contents

S1 Corrections to unfiltered read counts 2
S2 Corrections to filtered read counts 30
S3 Blacklisting problematic regions 58
S4 Distribution of mappabilities 86
S5 Distributions of median residuals 87
S6 Filter overlaps 92
S7 Detection limits 93
S8 Using a reference sample 96
S9 Comparisons to other methods 97

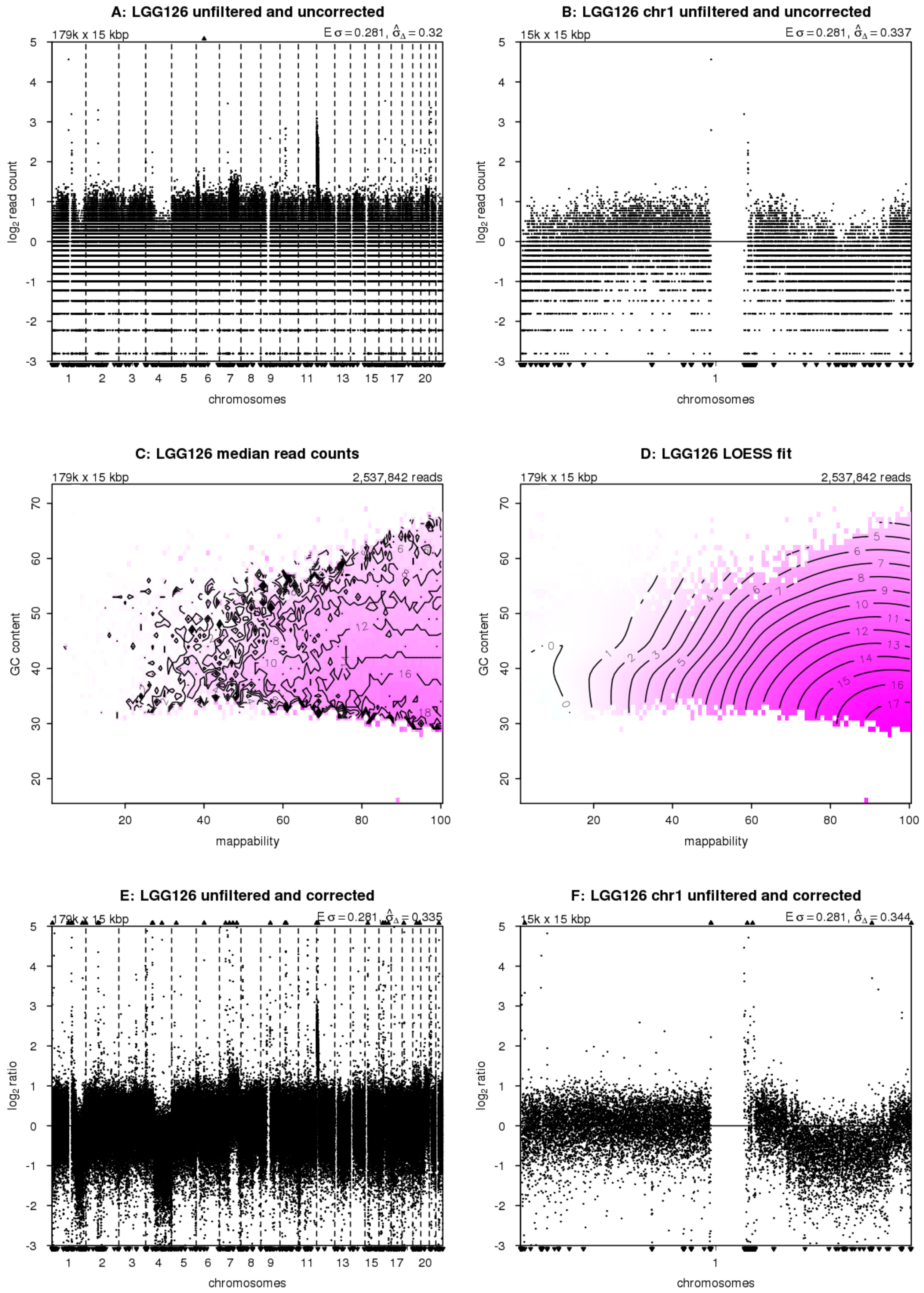

Figure S1: Corrections to unfiltered read counts. Copy number profiles from unfiltered and uncorrected data for (A) the whole genome and (B) chromosome 1, (C) median read counts per bin as a function of GC content and mappability, (D) the corresponding LOESS fit, and copy number profiles from unfiltered and corrected data for (E) the whole genome and (F) chromosome 1. In the copy number profiles, bins are ordered along the x-axis by their genomic positions, and y-axis shows median-normalized $\log_2$-transformed data. Small triangles at the top and bottom edges represent data points that fall outside the plot area. Top-left corners show the number and size of bins. Top-right corner of the read counts shows the total number of sequence reads, and the top-right corners of the copy number profiles the expected and measured standard deviation. The expected standard deviation (E σ) is defined as $\sqrt{1/N}$, where N is the average number of reads per bin. The measured standard deviation ($\hat{\sigma}_\Delta$) is calculated from the data with a 0.1%-trimmed first-order estimate (prior to $\log_2$-transforming the data for plotting).

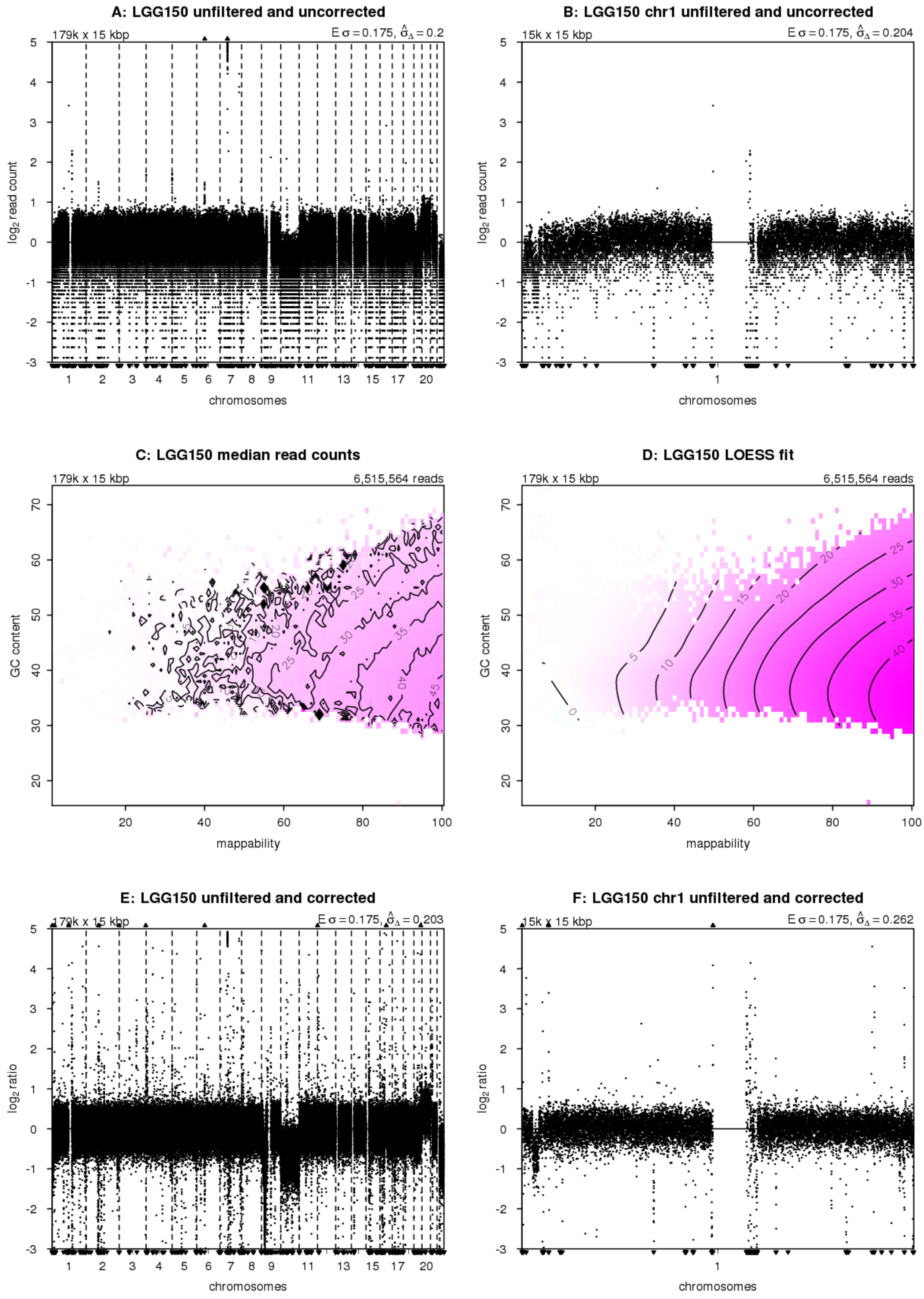

Figure S1: Corrections to unfiltered read counts (cont.)

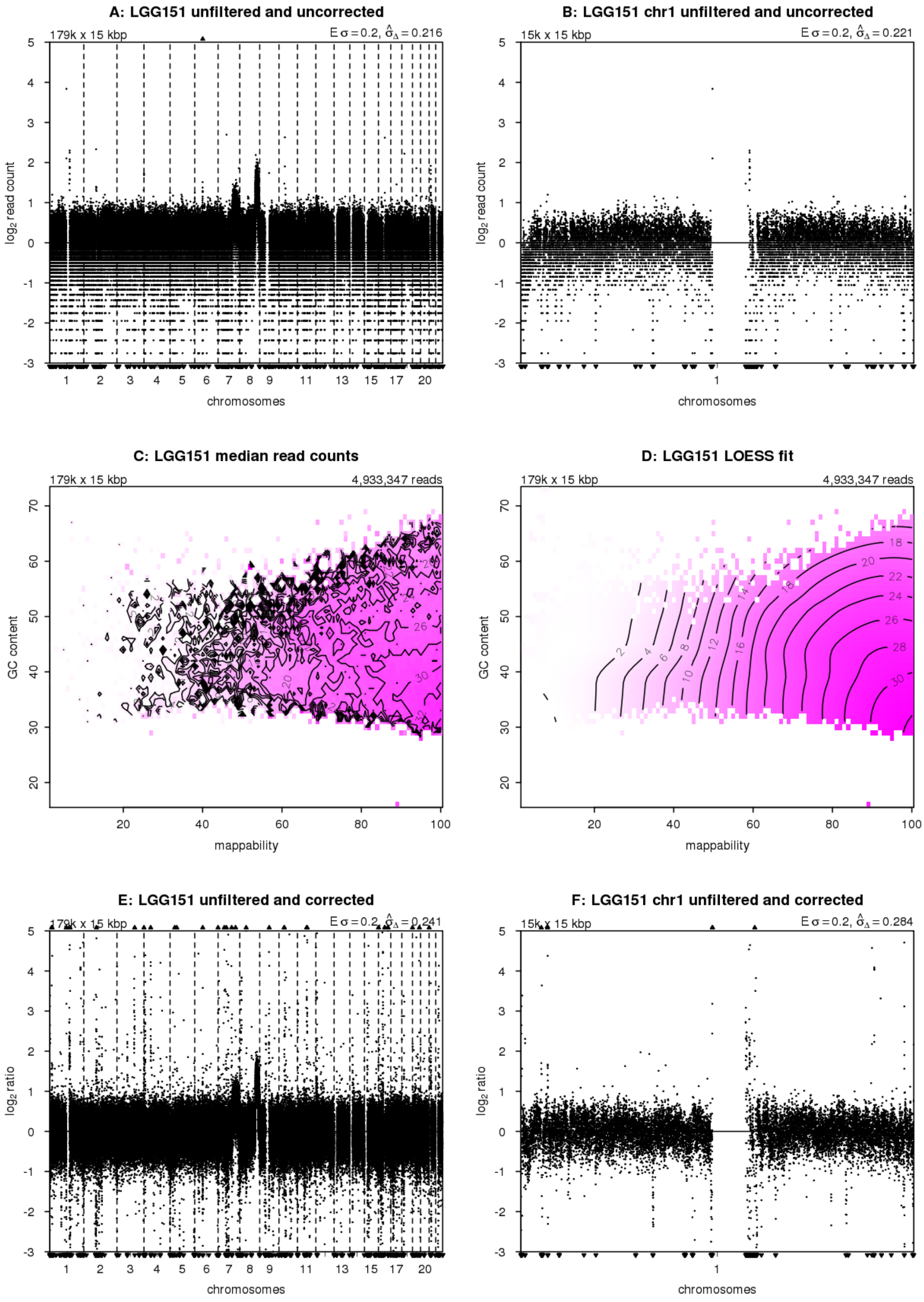

Figure S1: Corrections to unfiltered read counts (cont.)

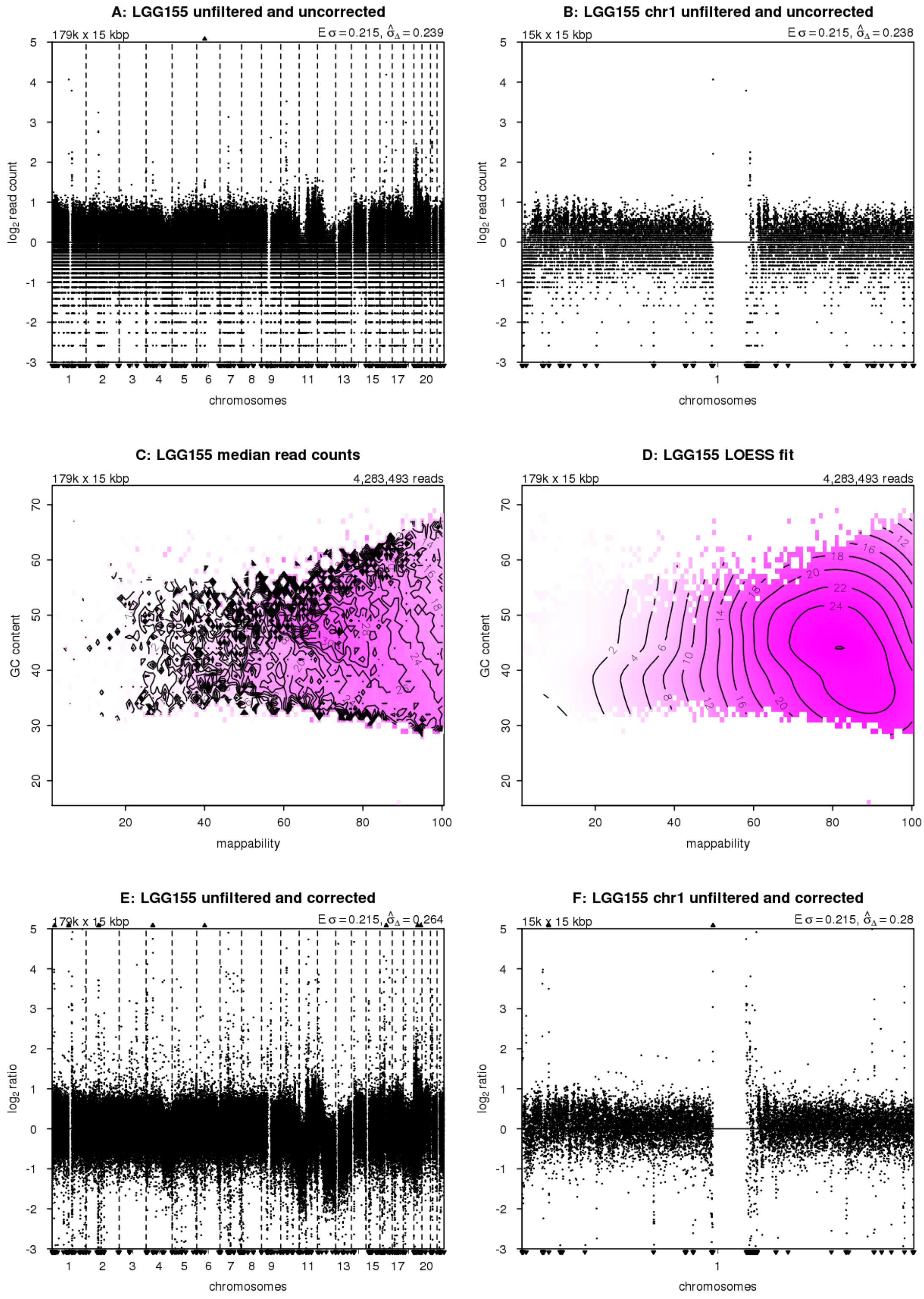

Figure S1: Corrections to unfiltered read counts (cont.)

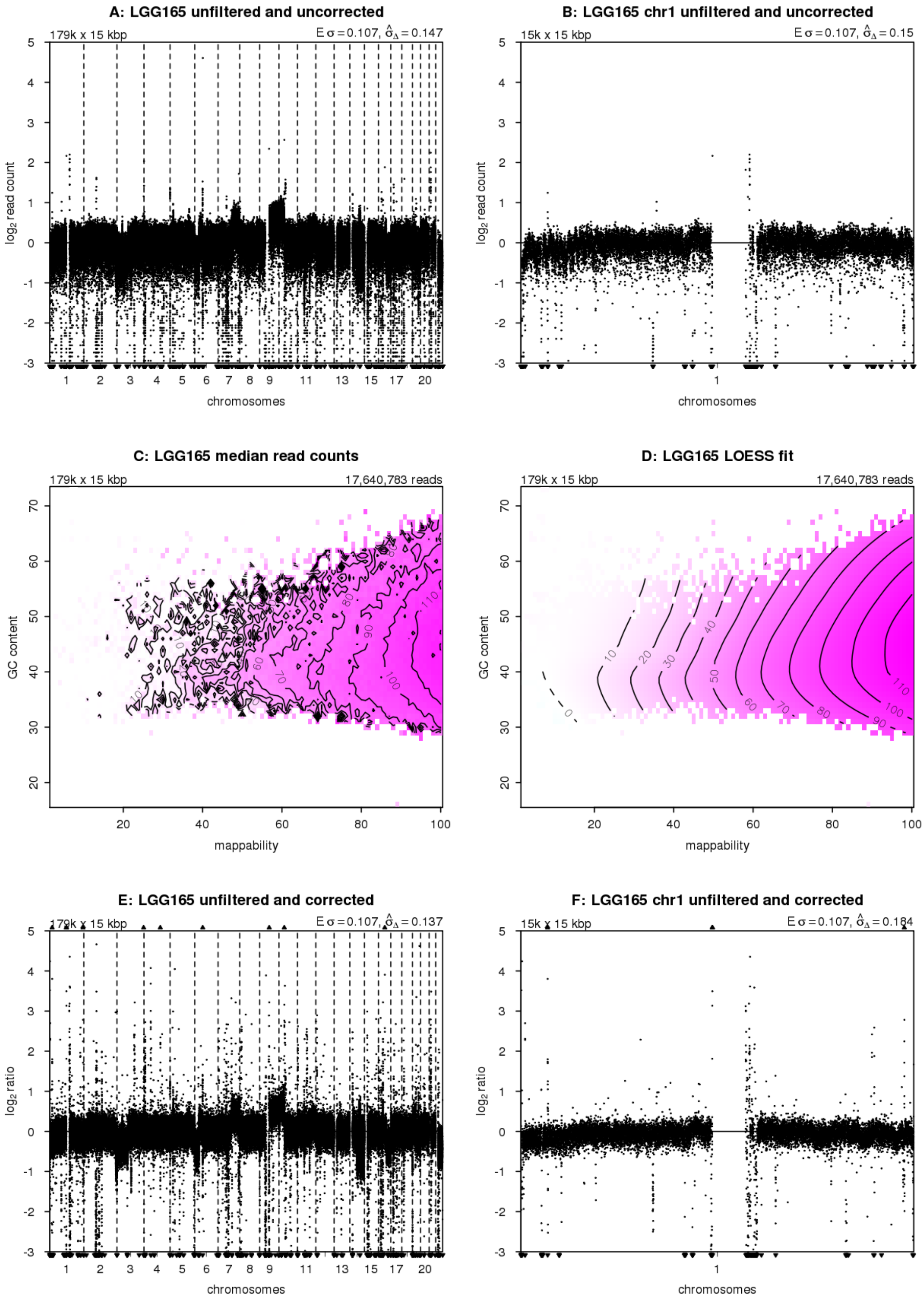

Figure S1: Corrections to unfiltered read counts (cont.)

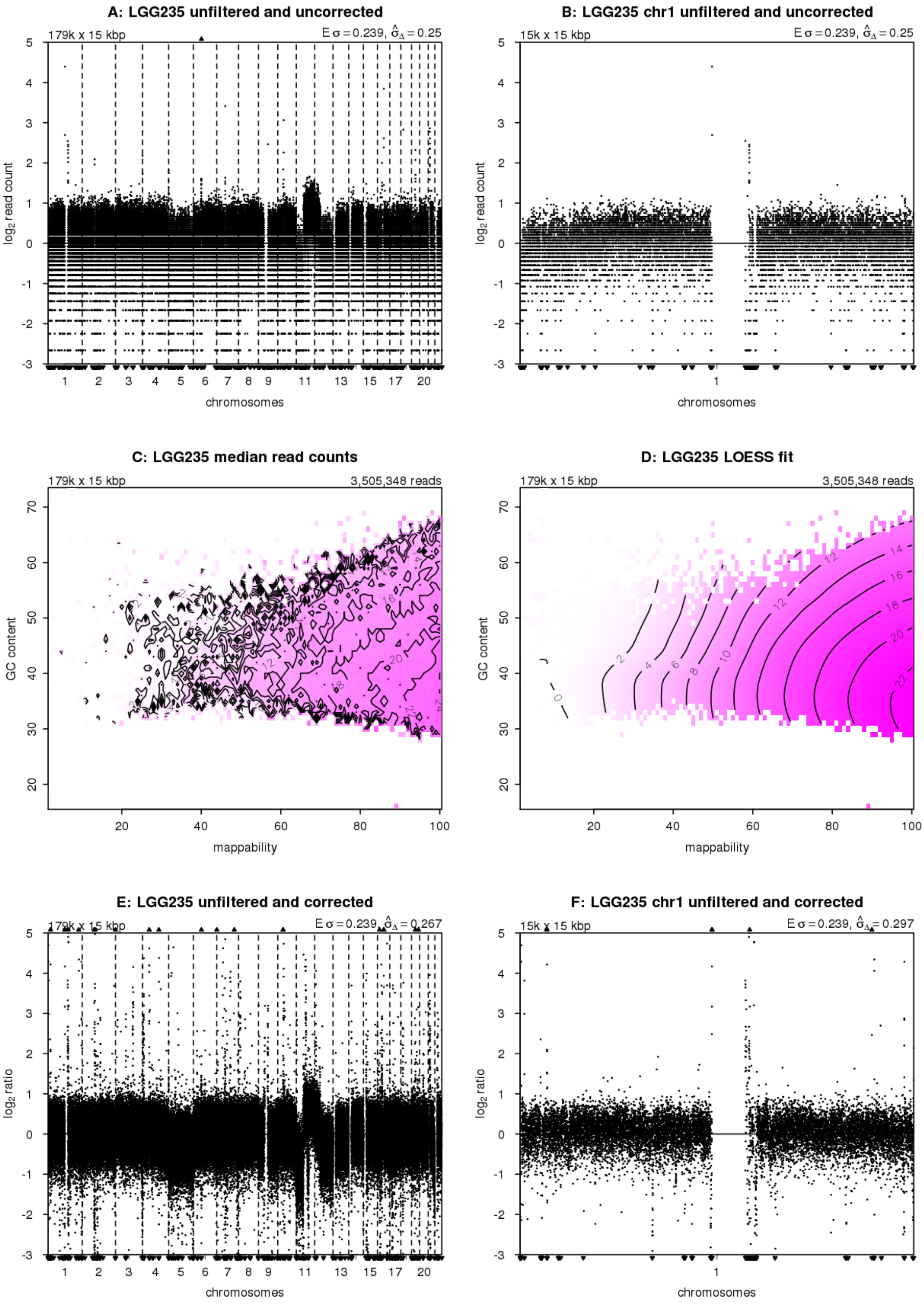

Figure S1: Corrections to unfiltered read counts (cont.)

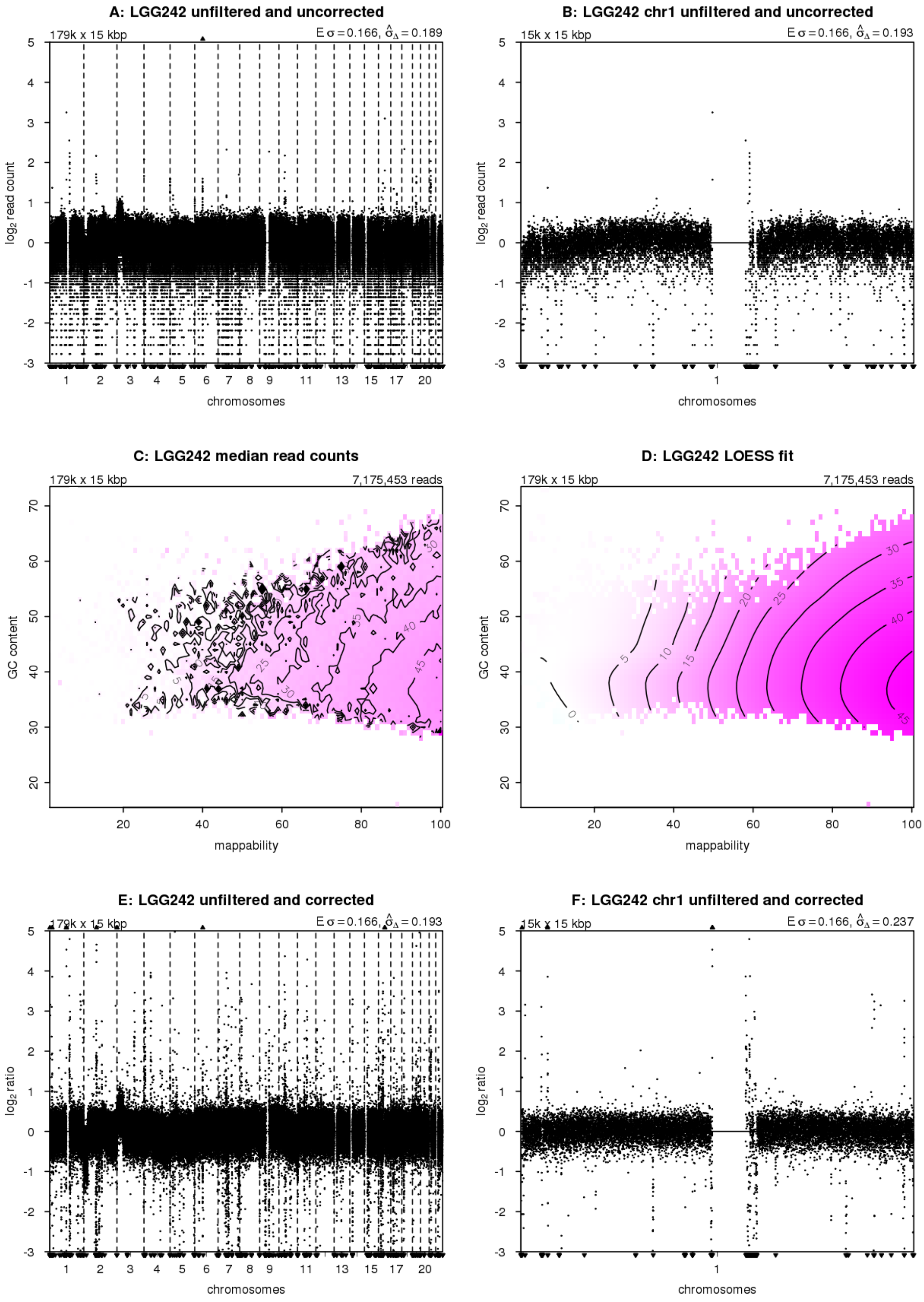

Figure S1: Corrections to unfiltered read counts (cont.)

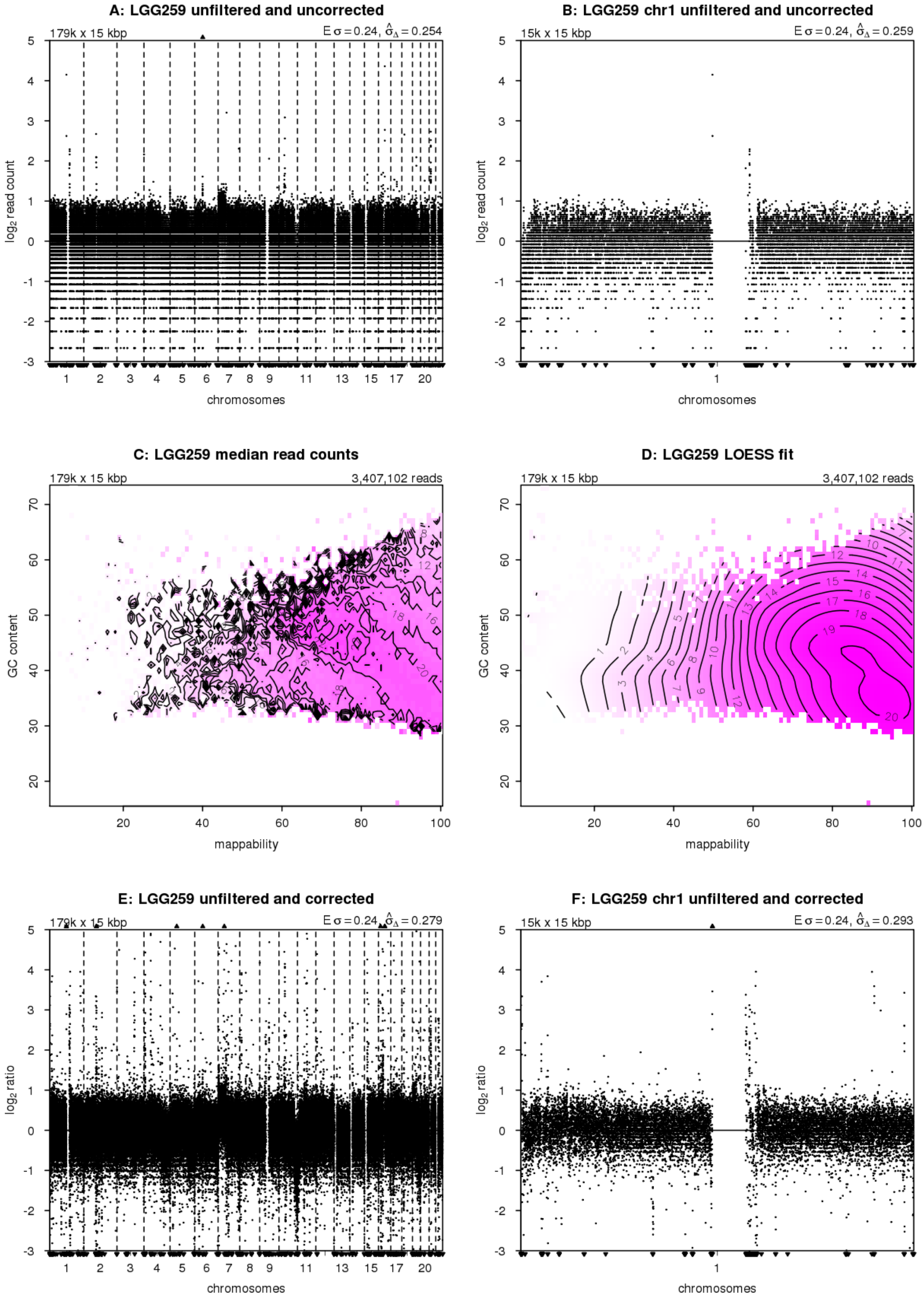

Figure S1: Corrections to unfiltered read counts (cont.)

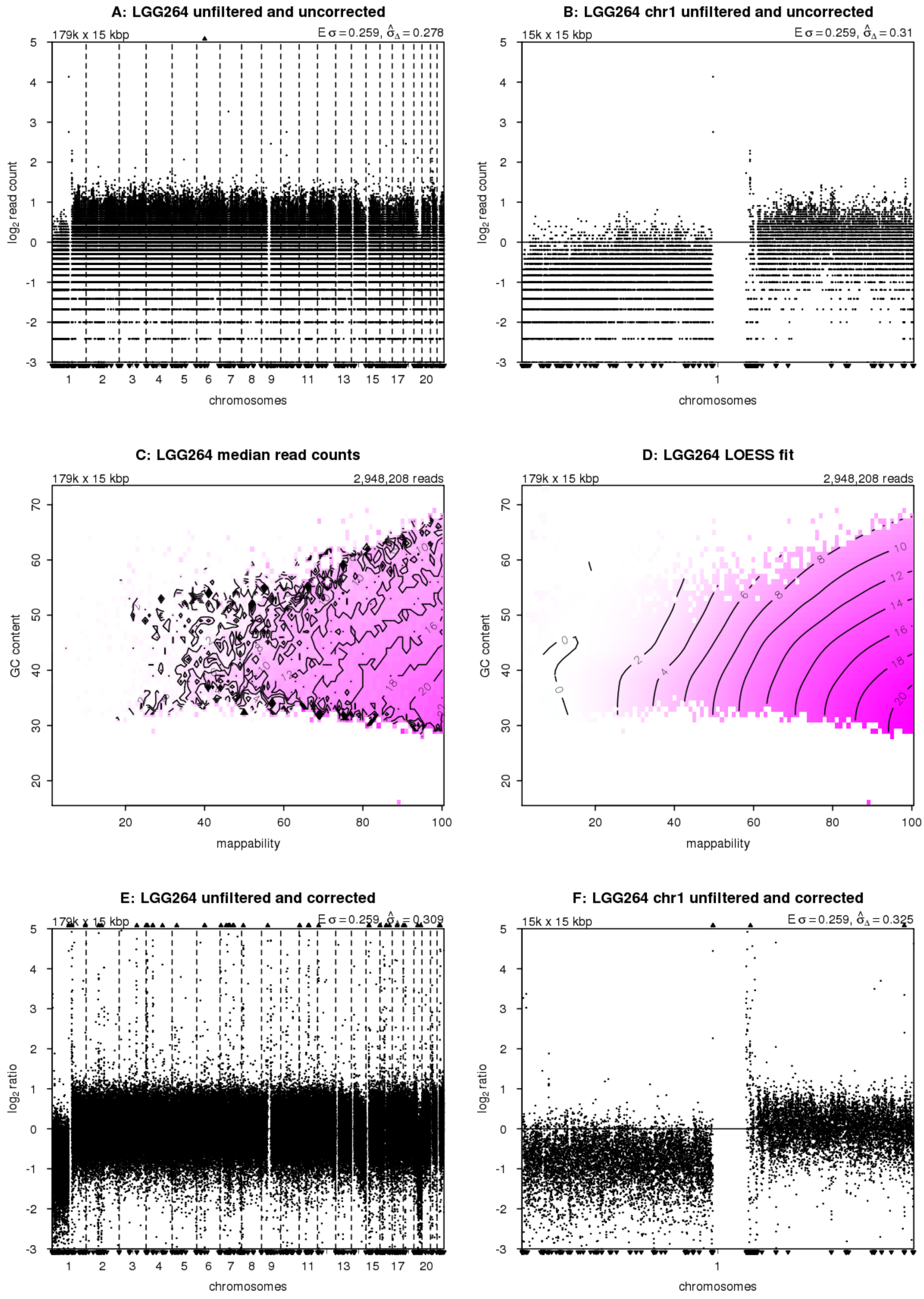

Figure S1: Corrections to unfiltered read counts (cont.)

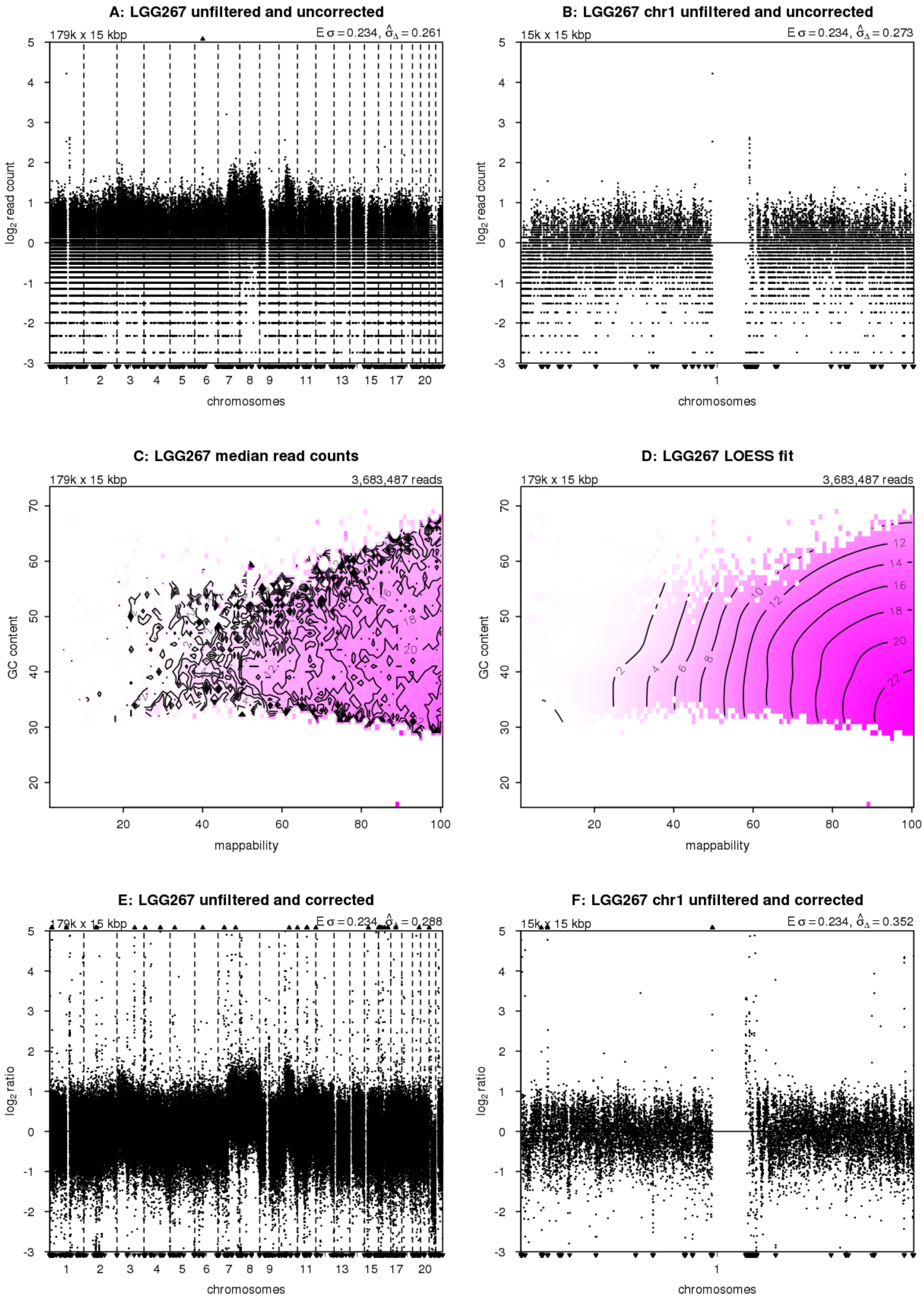

Figure S1: Corrections to unfiltered read counts (cont.)

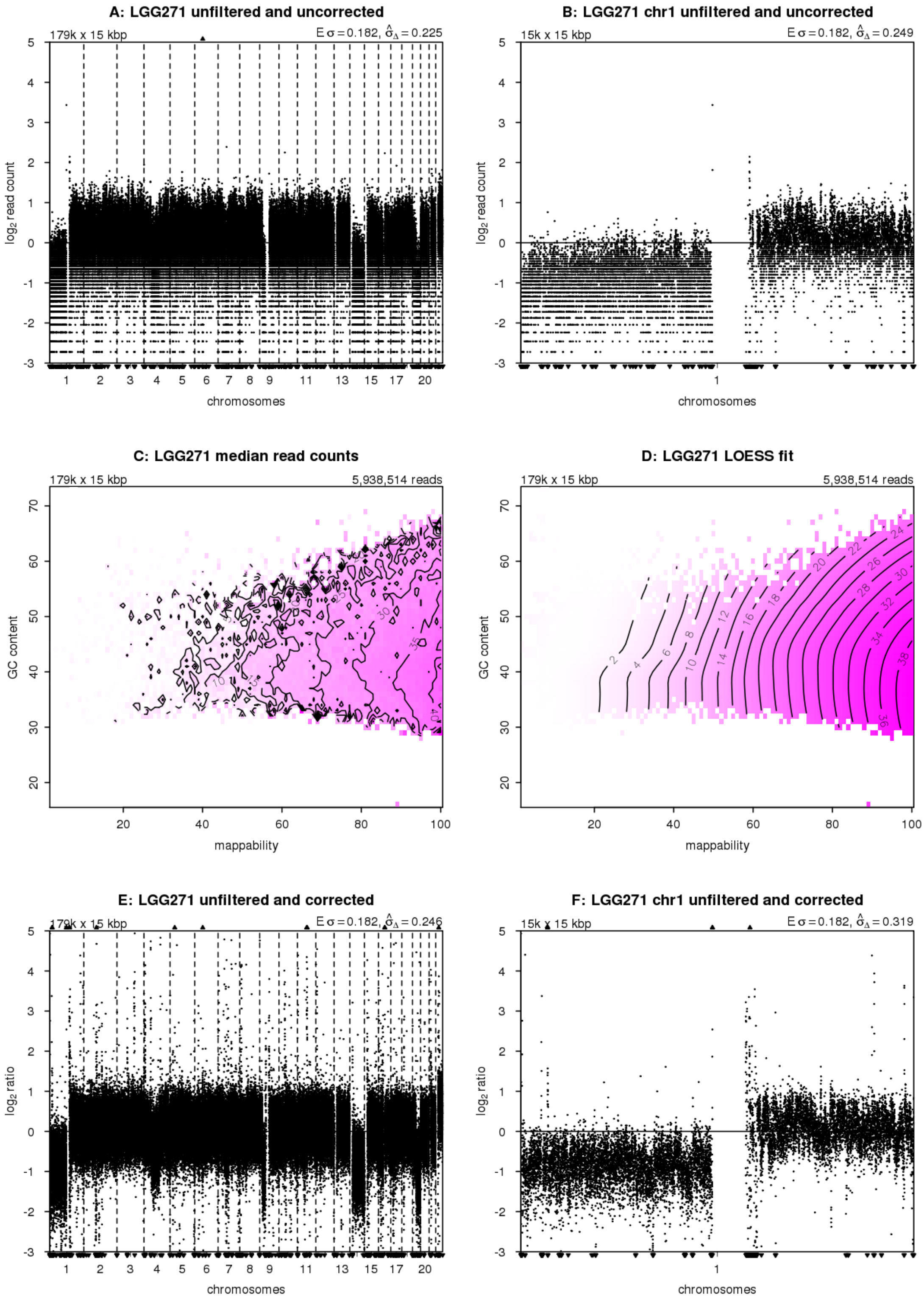

Figure S1: Corrections to unfiltered read counts (cont.)

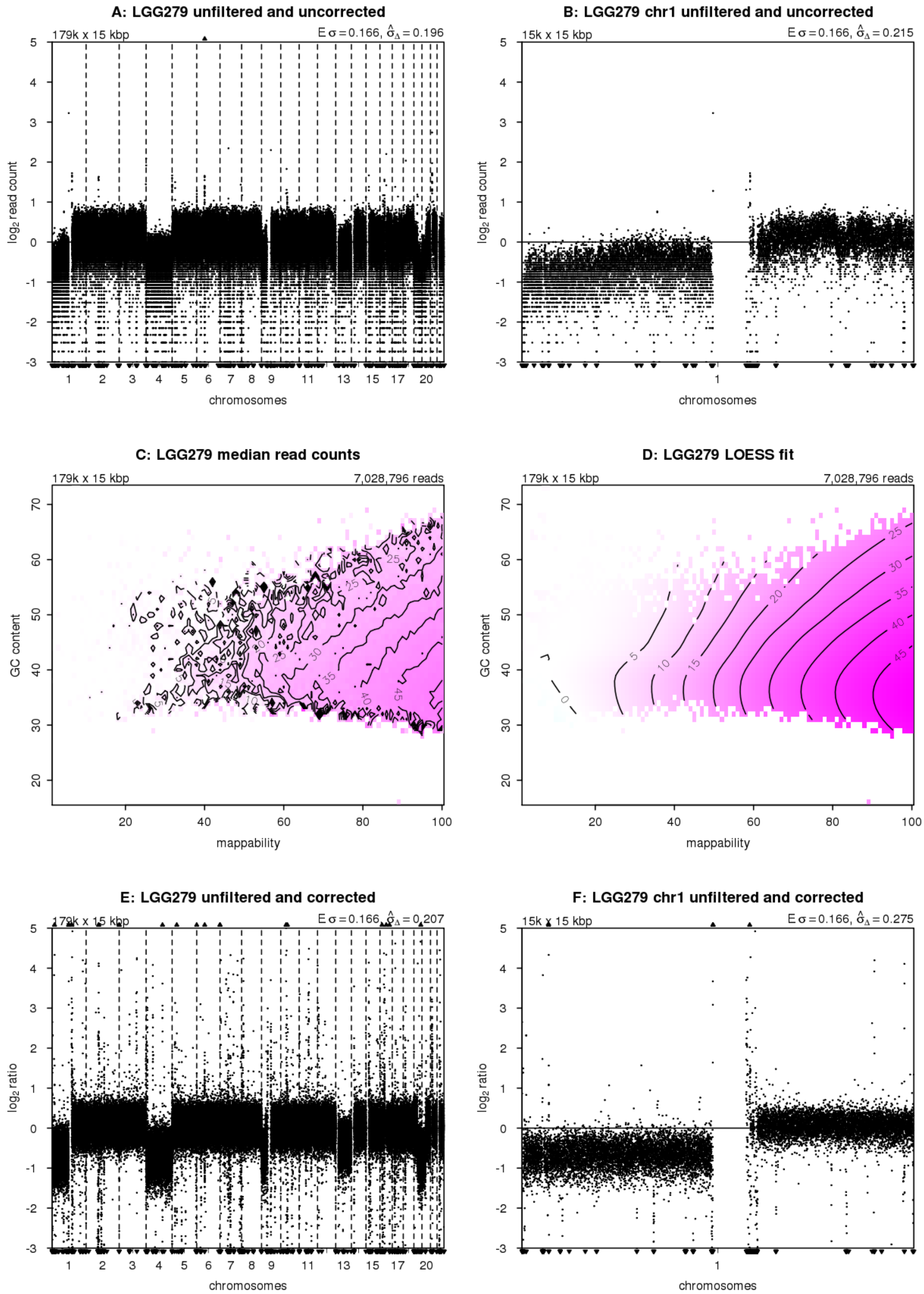

Figure S1: Corrections to unfiltered read counts (cont.)

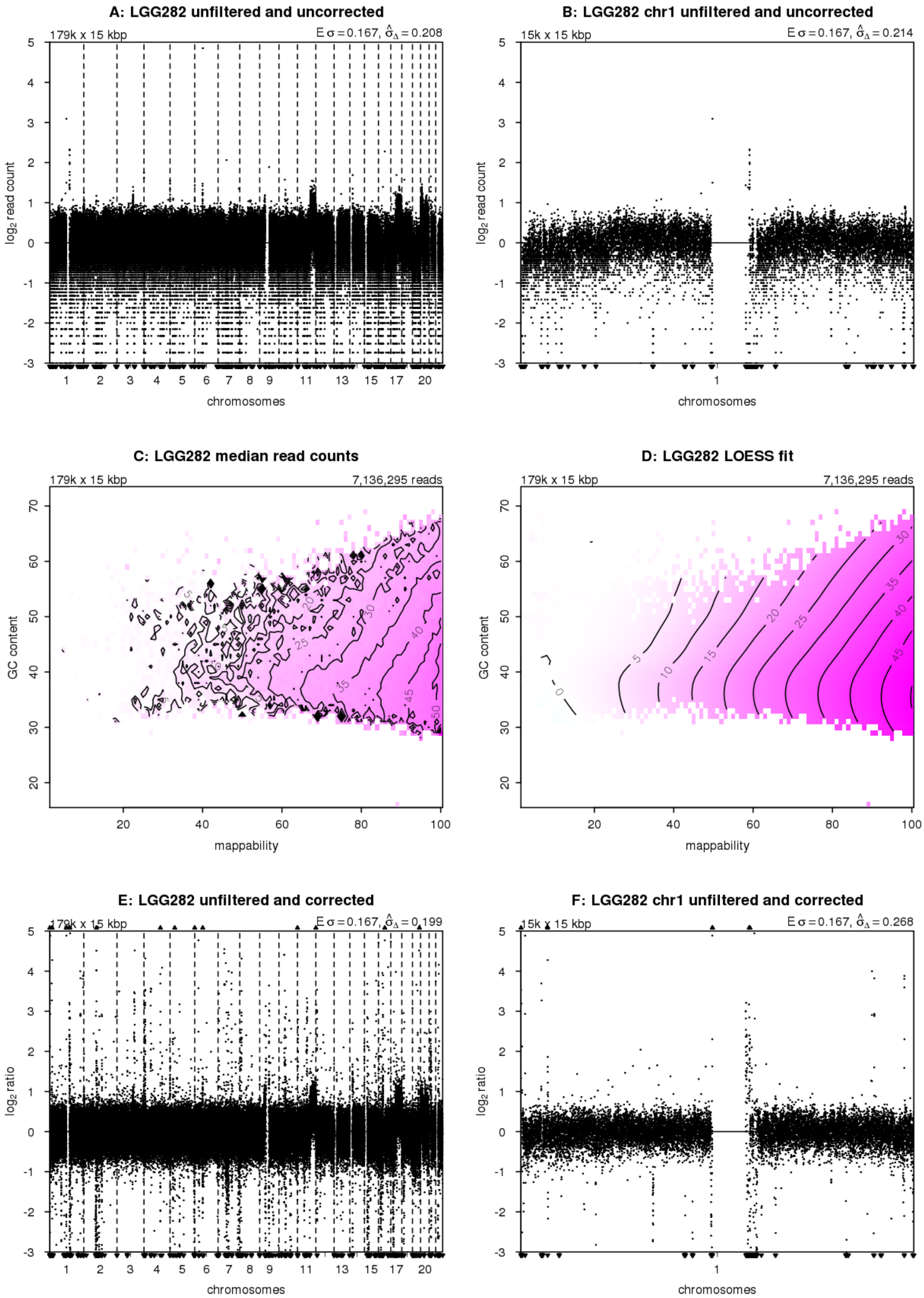

Figure S1: Corrections to unfiltered read counts (cont.)

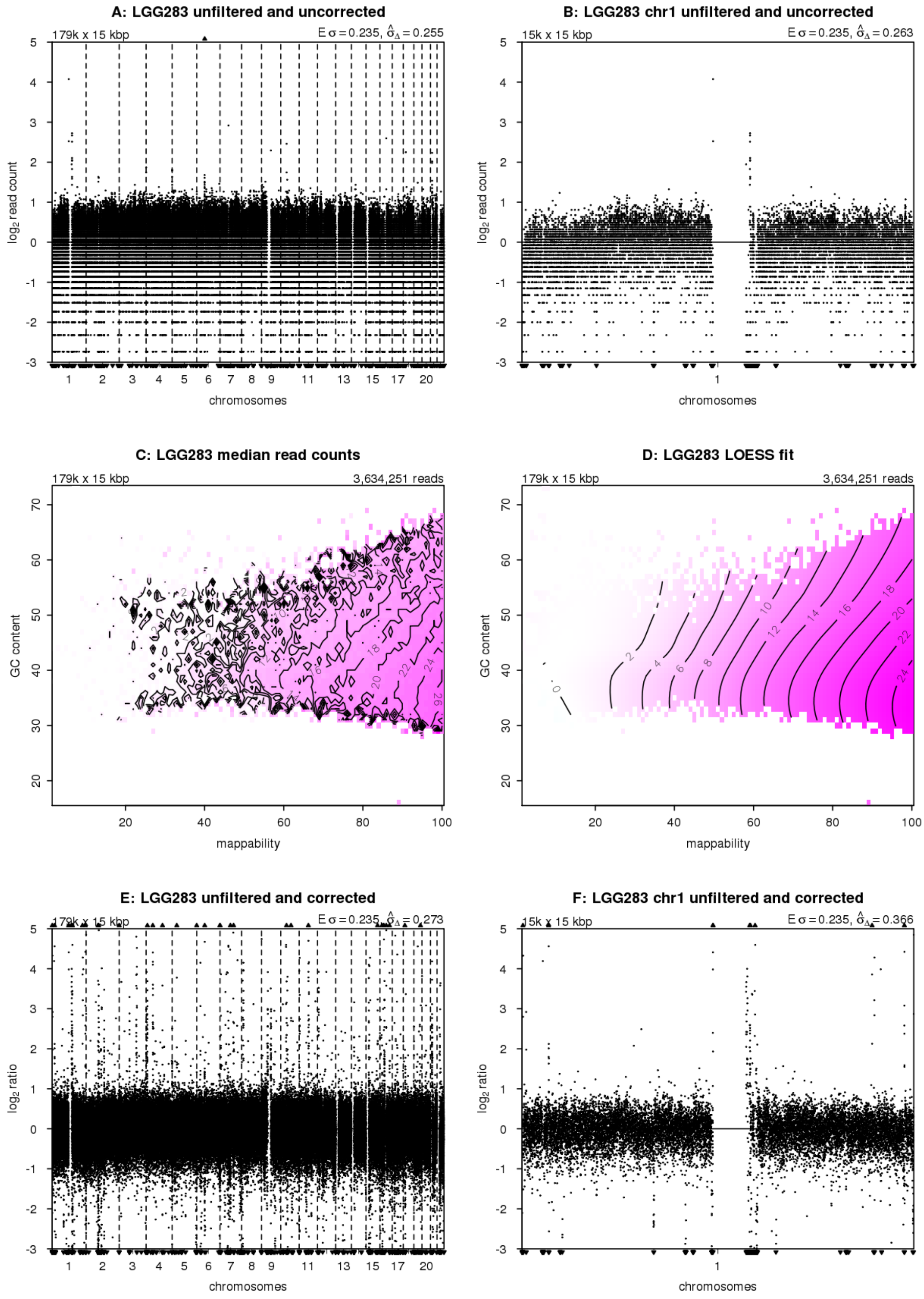

Figure S1: Corrections to unfiltered read counts (cont.)

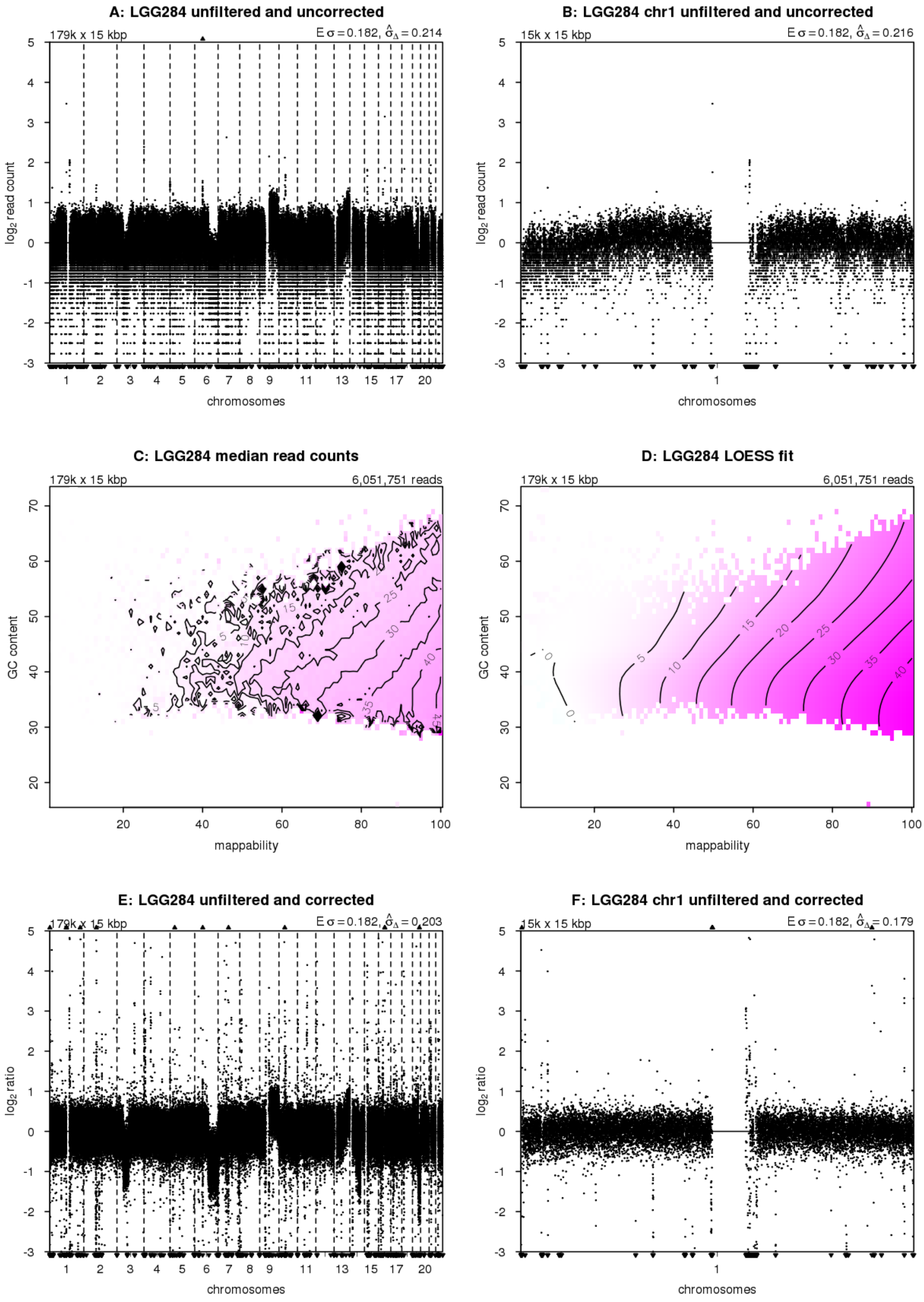

Figure S1: Corrections to unfiltered read counts (cont.)

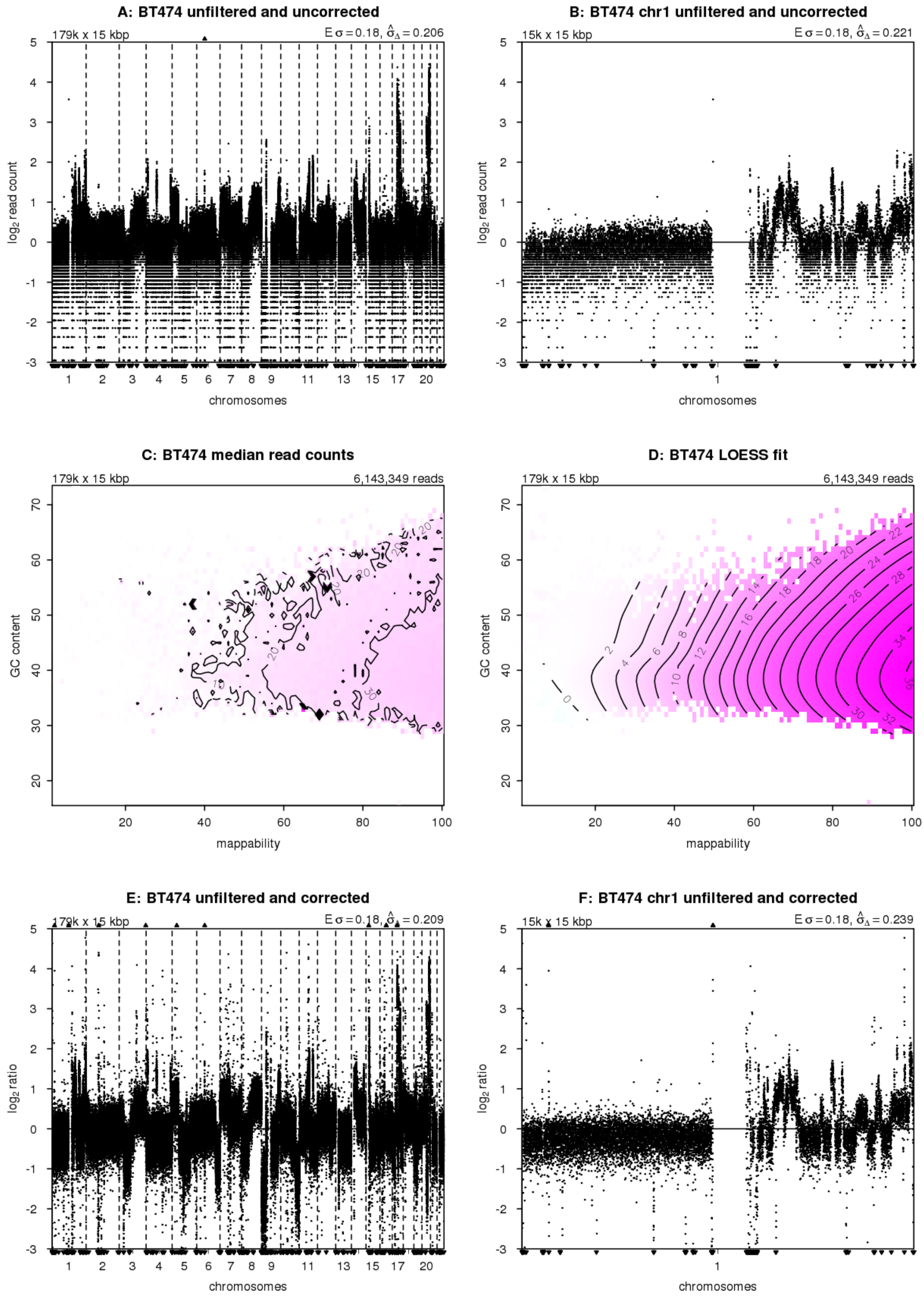

Figure S1: Corrections to unfiltered read counts (cont.)

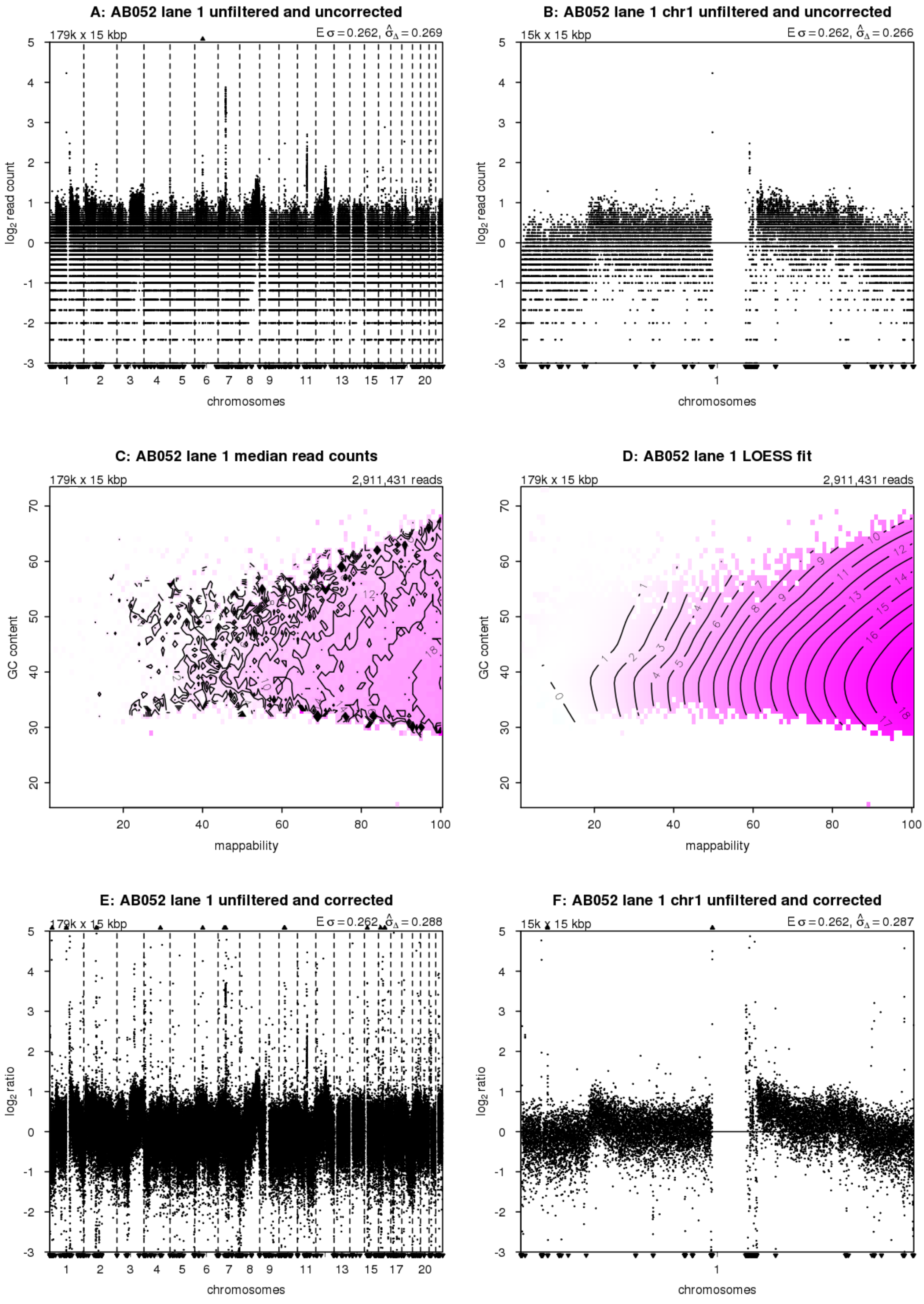

Figure S1: Corrections to unfiltered read counts (cont.)

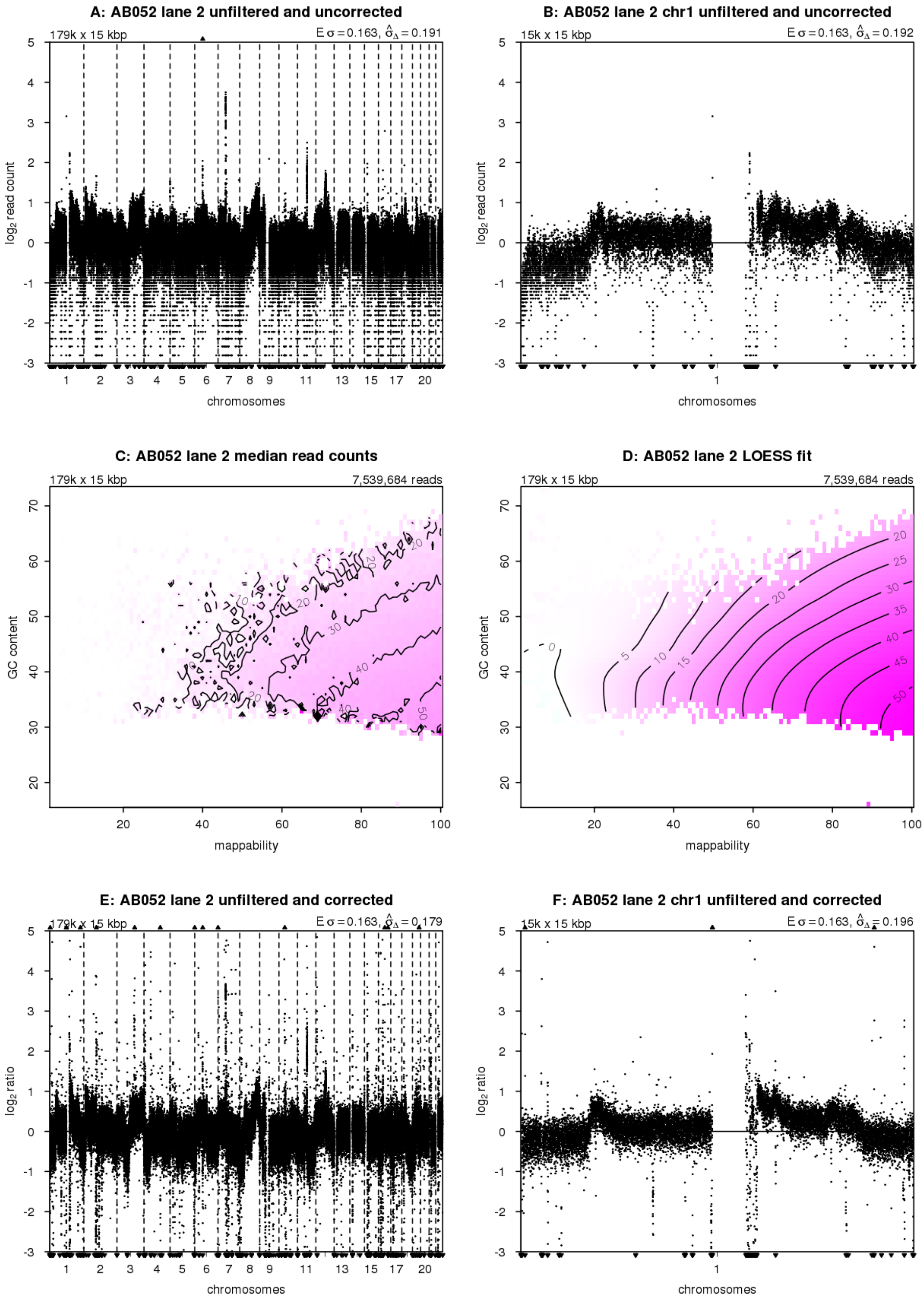

Figure S1: Corrections to unfiltered read counts (cont.)

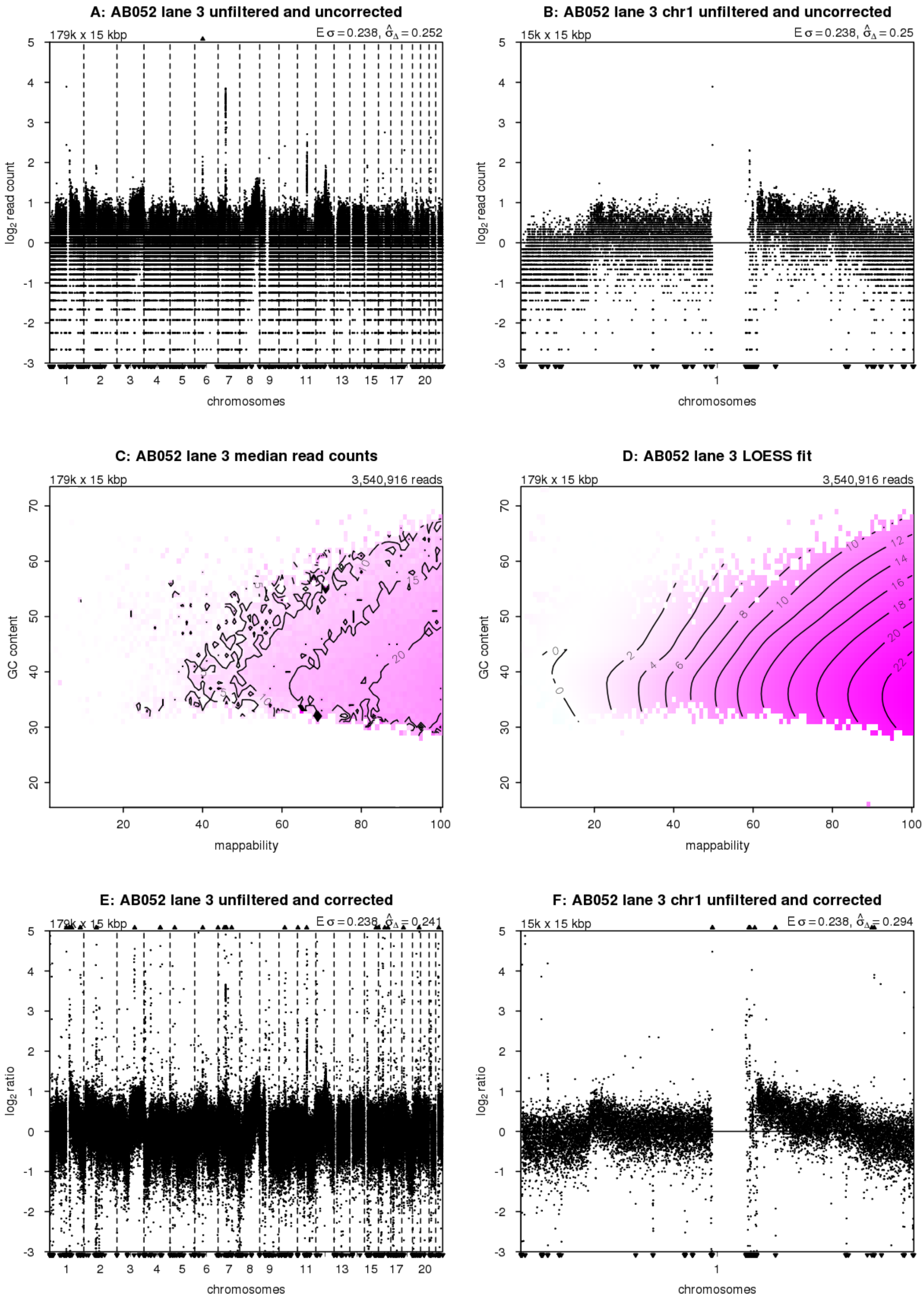

Figure S1: Corrections to unfiltered read counts (cont.)

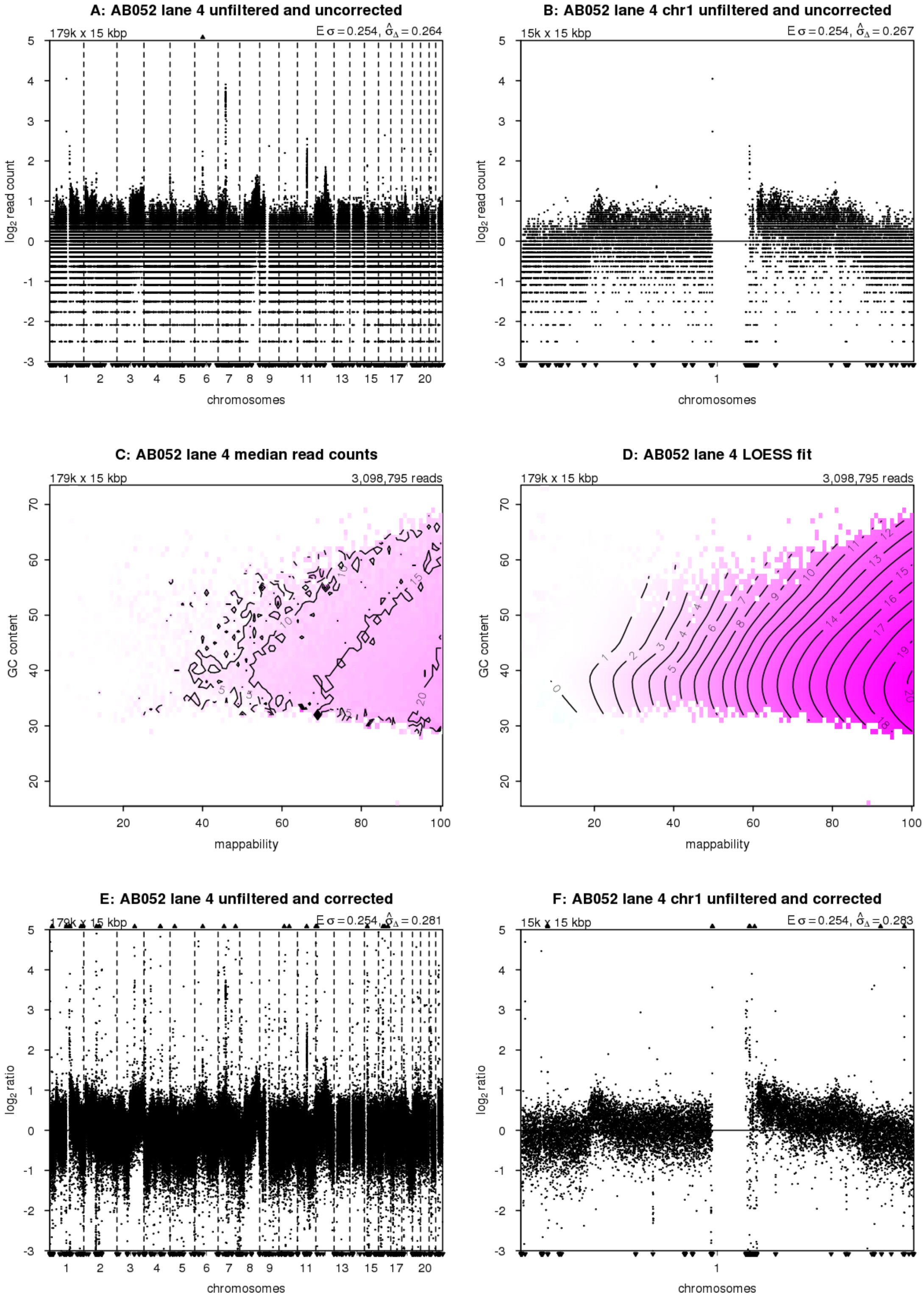

Figure S1: Corrections to unfiltered read counts (cont.)

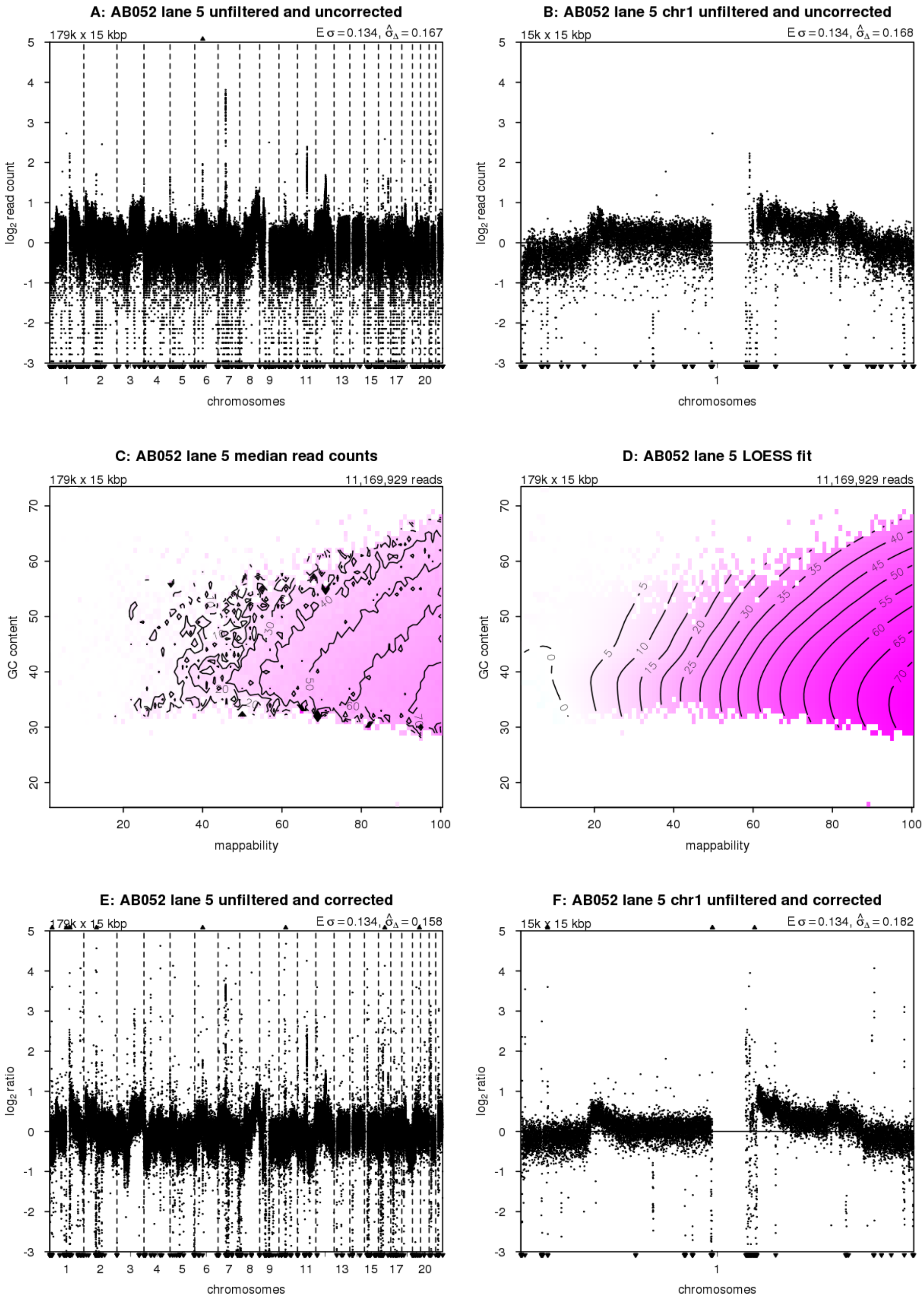

Figure S1: Corrections to unfiltered read counts (cont.)

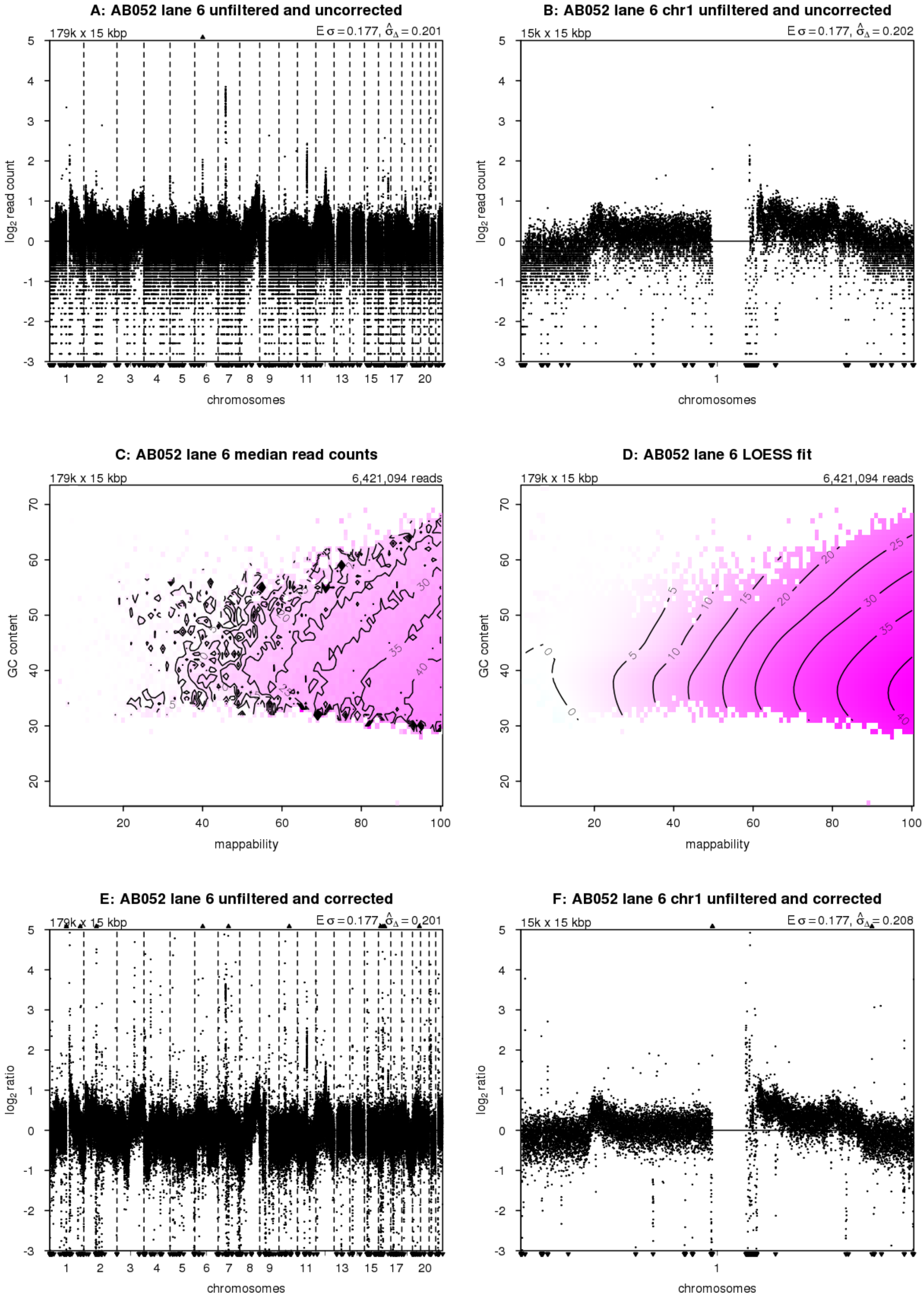

Figure S1: Corrections to unfiltered read counts (cont.)

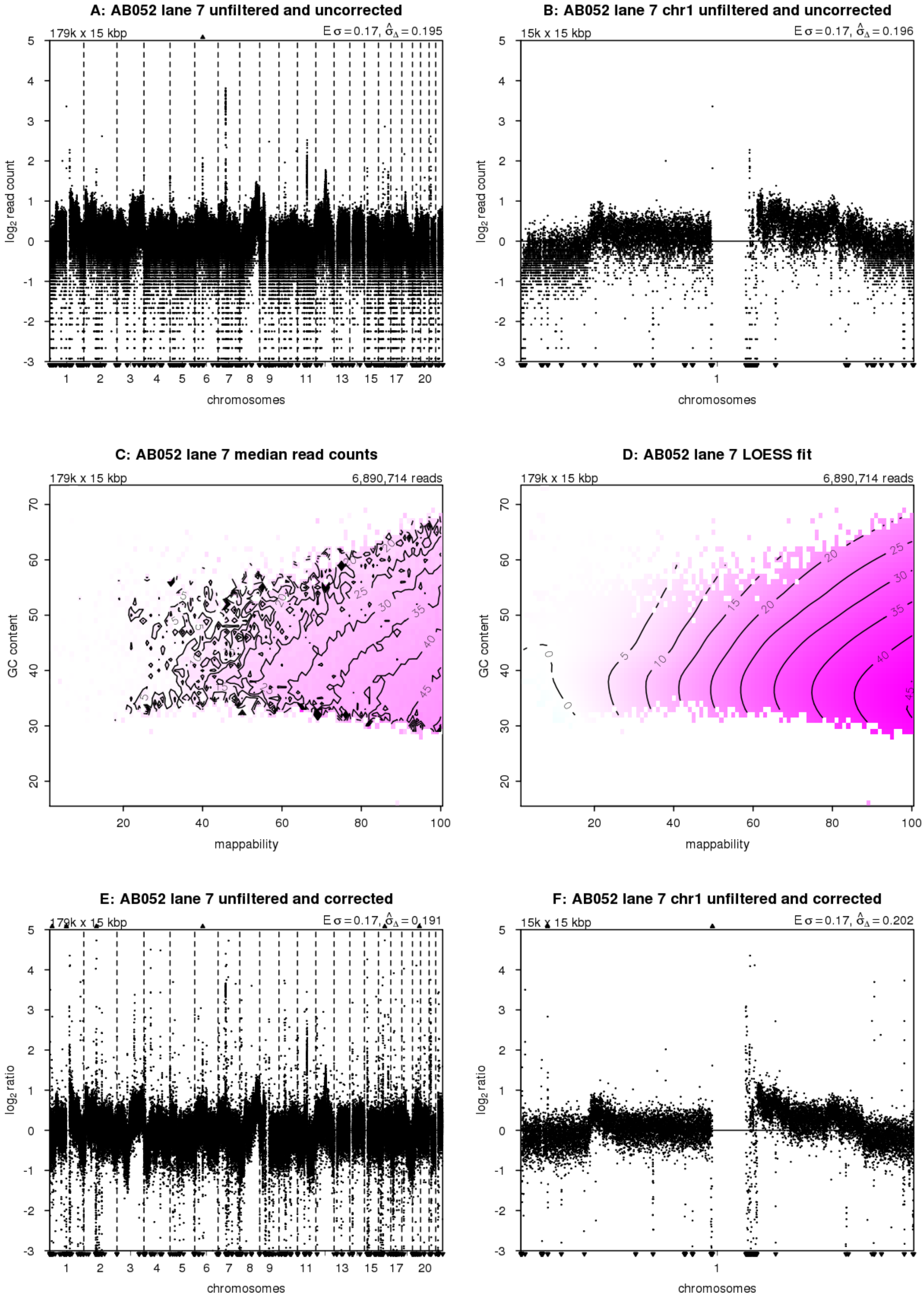

Figure S1: Corrections to unfiltered read counts (cont.)

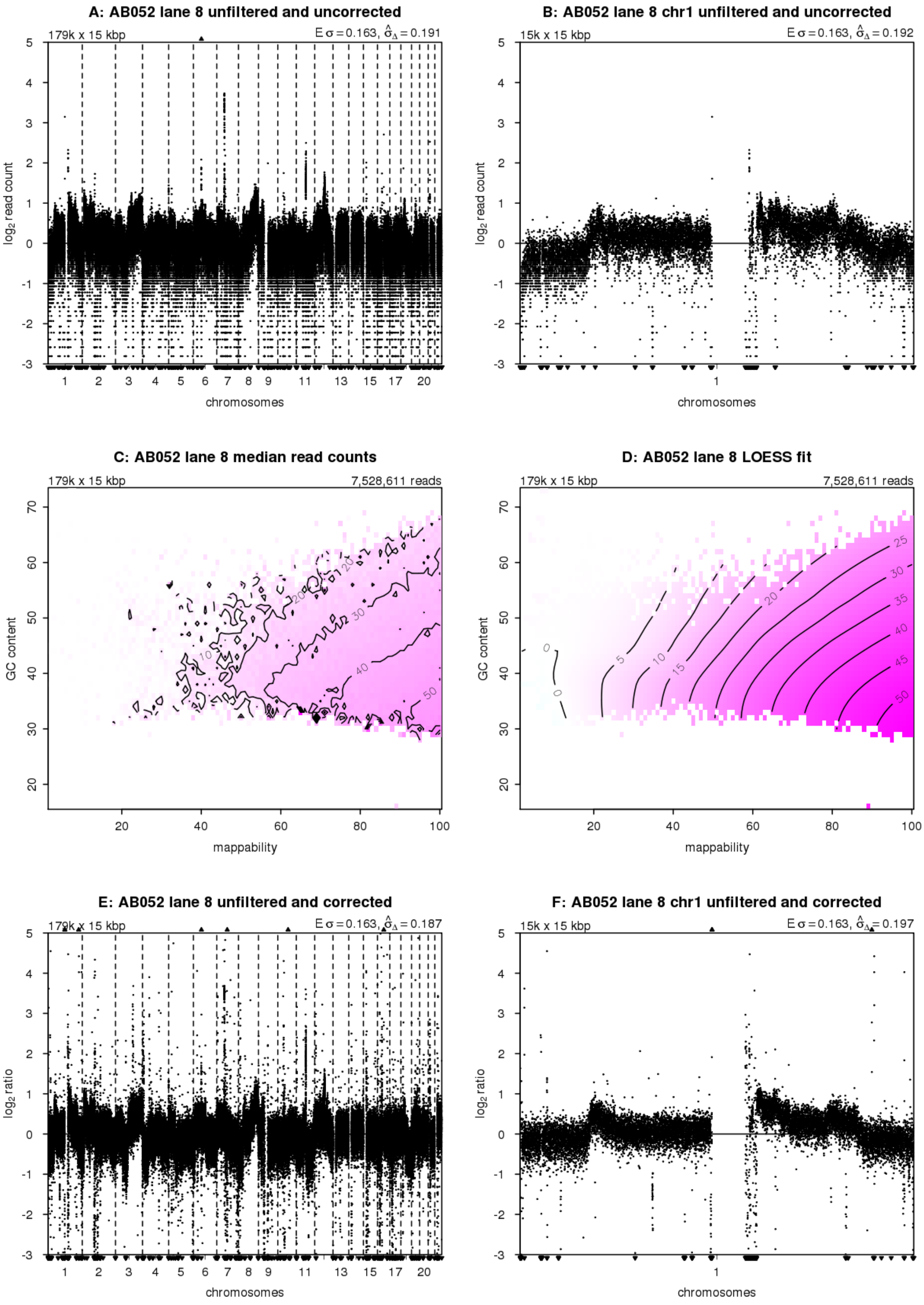

Figure S1: Corrections to unfiltered read counts (cont.)

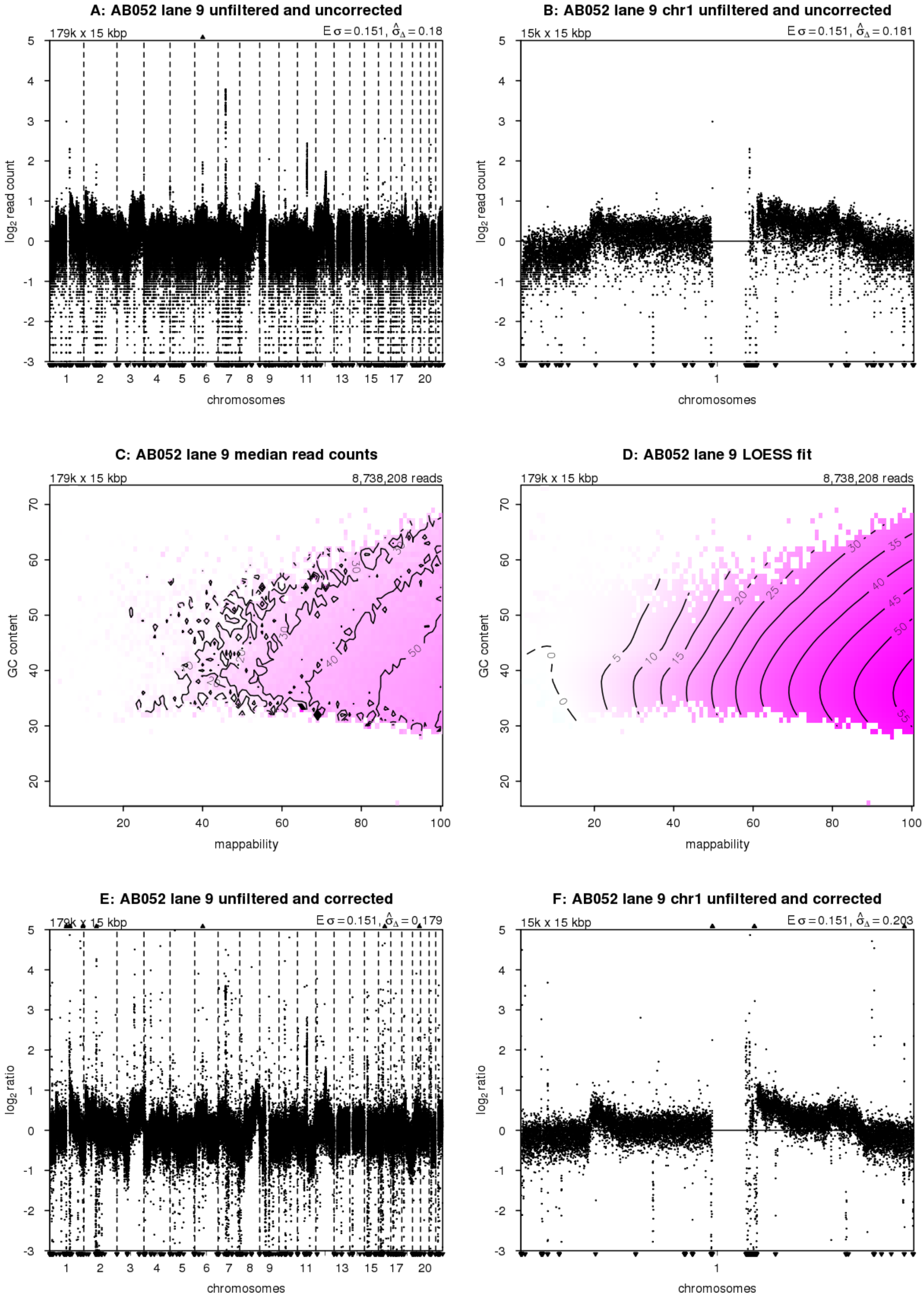

Figure S1: Corrections to unfiltered read counts (cont.)

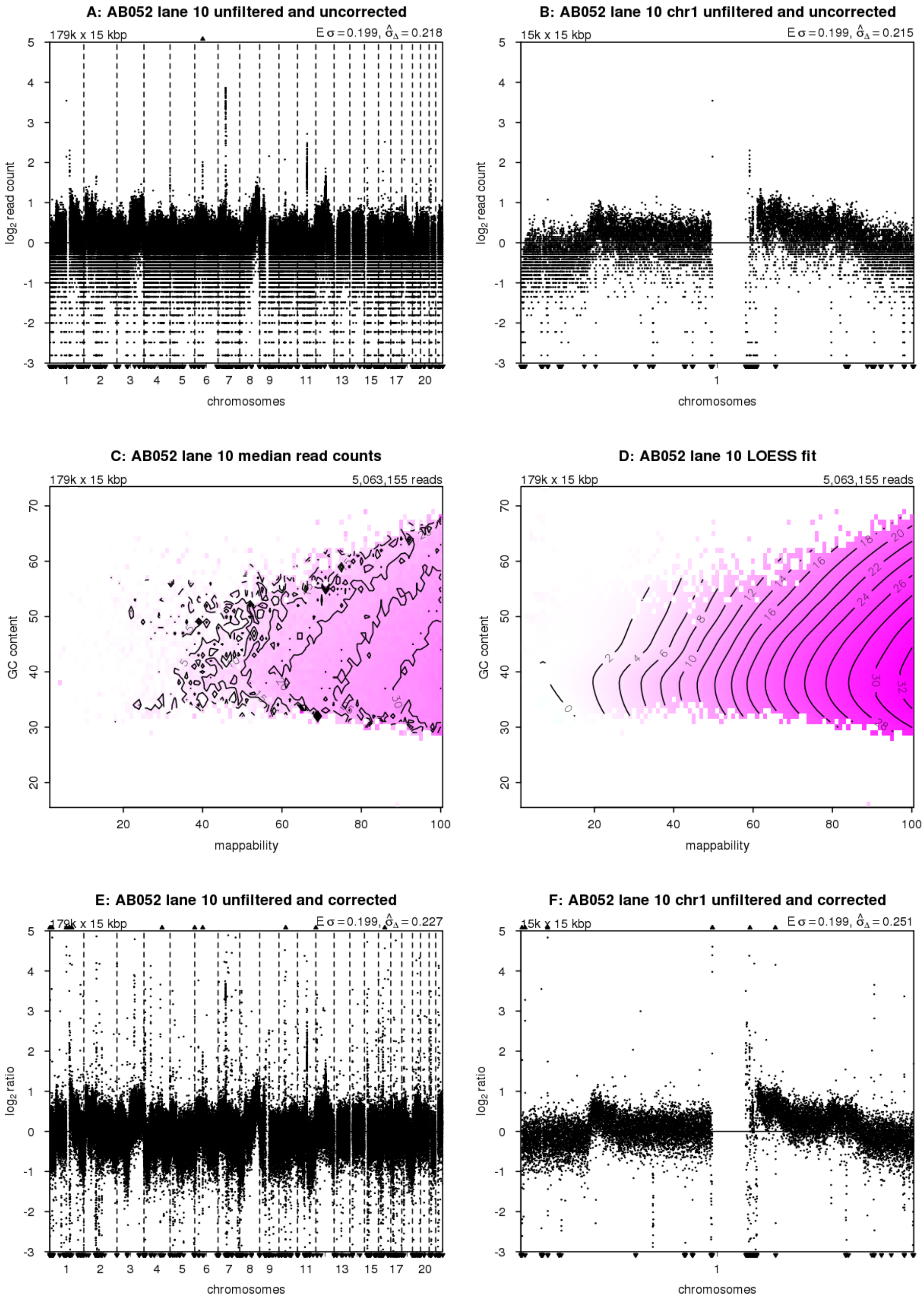

Figure S1: Corrections to unfiltered read counts (cont.)

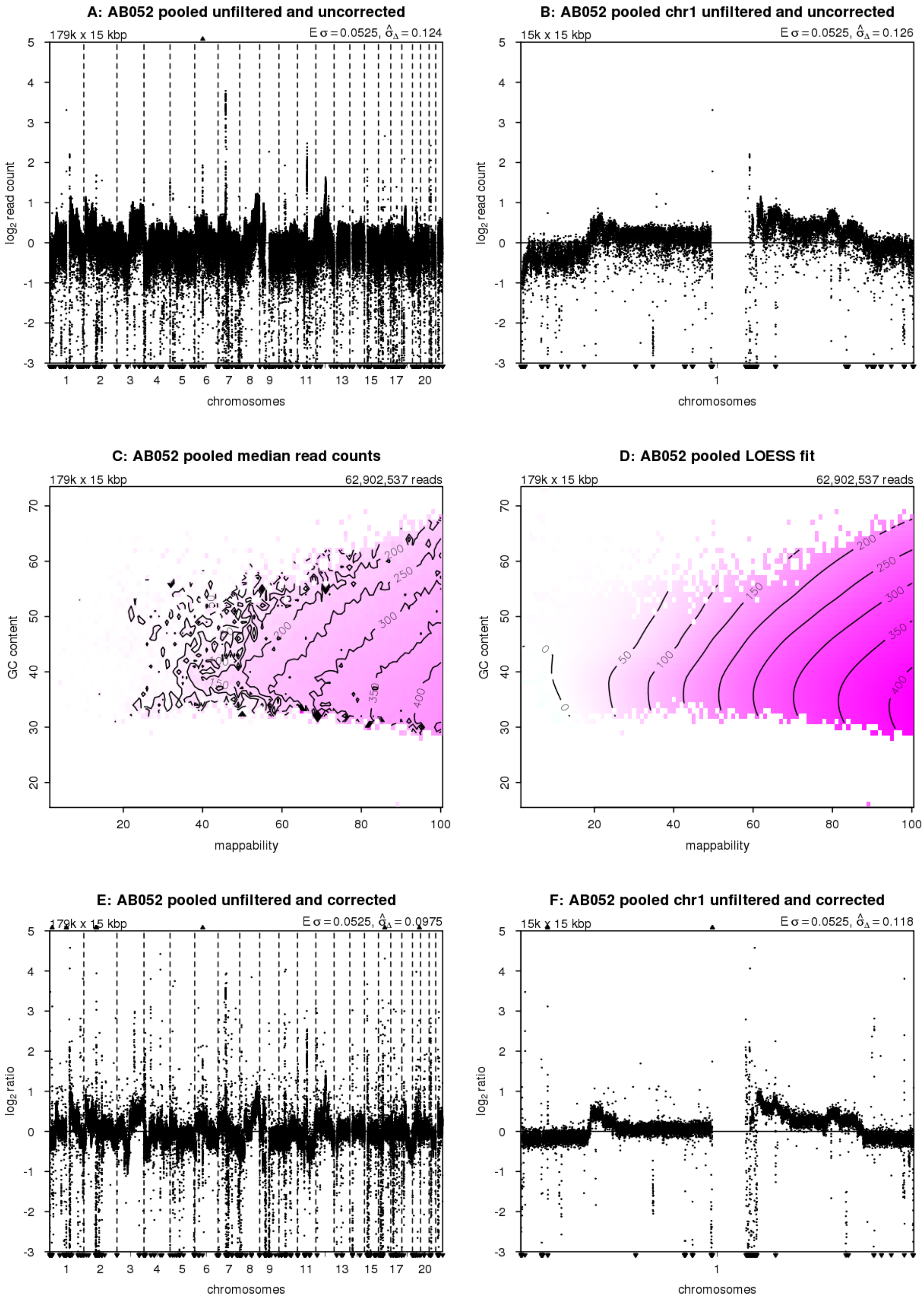

Figure S1: Corrections to unfiltered read counts (cont.)

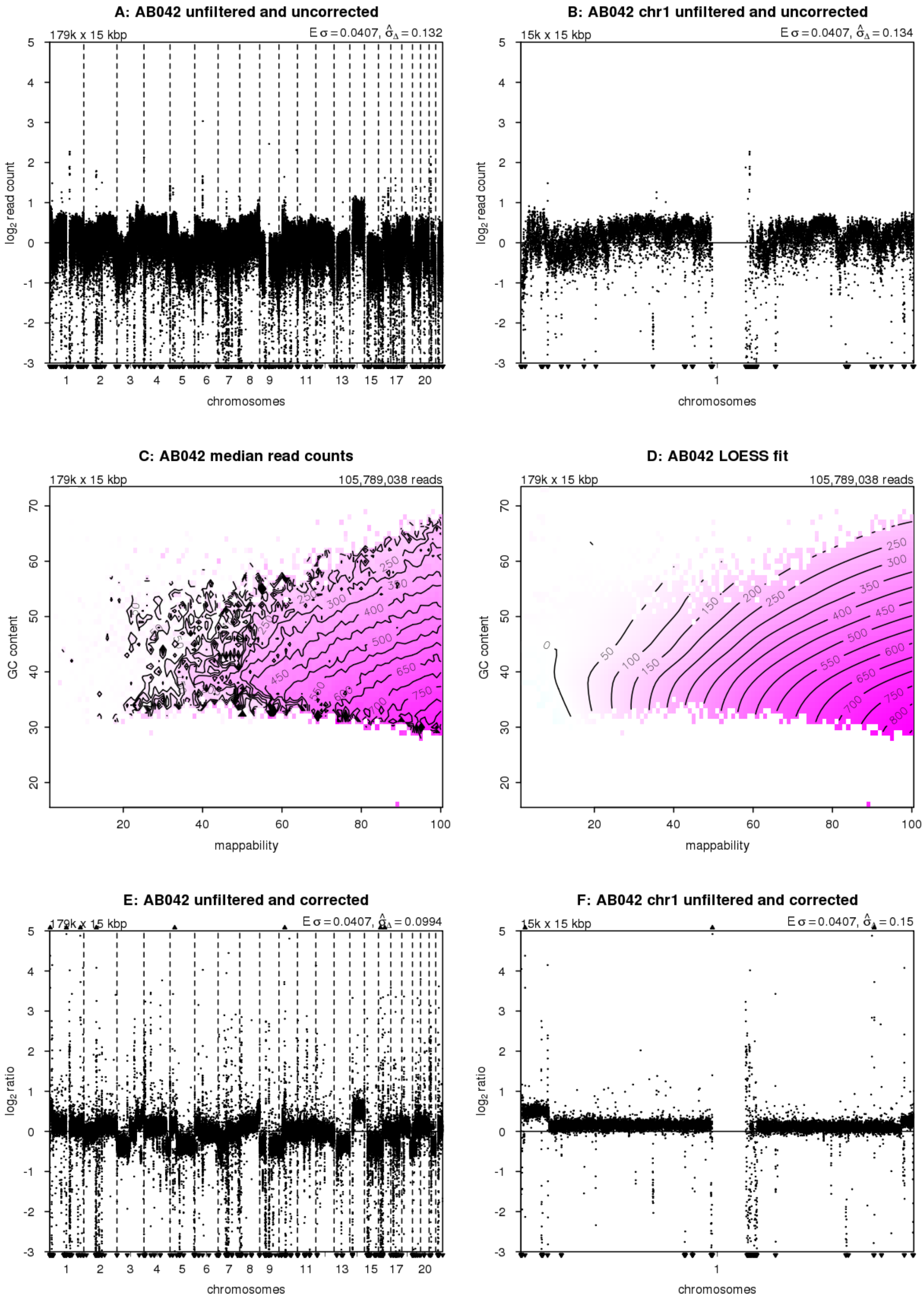

Figure S1: Corrections to unfiltered read counts (cont.)

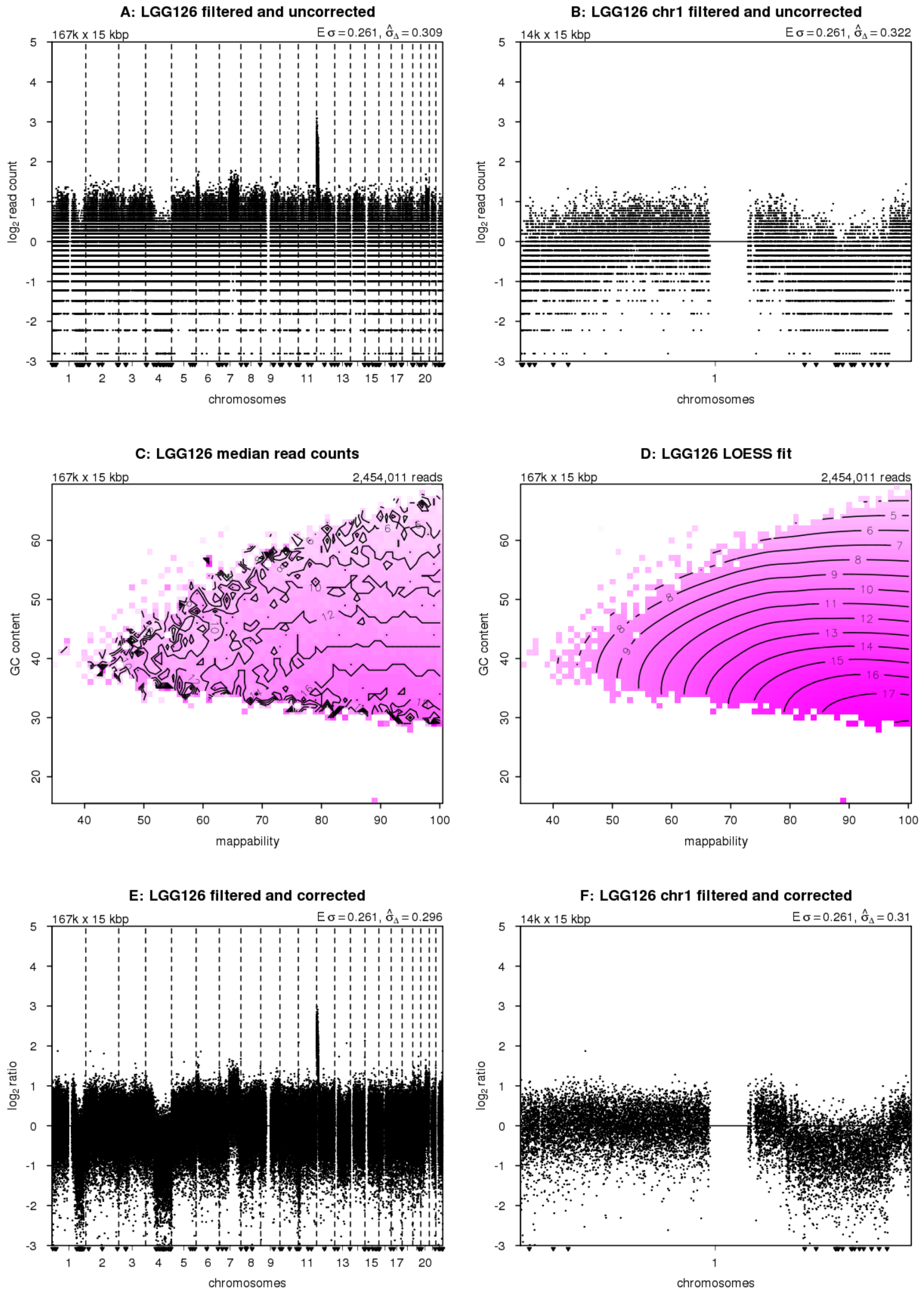

Figure S2: Corrections to filtered read counts. Copy number profiles from filtered and uncorrected data for (A) the whole genome and (B) chromosome 1, (C) median read counts per bin as a function of GC content and mappability, (D) the corresponding LOESS fit, and copy number profiles from filtered and corrected data for (E) the whole genome and (F) chromosome 1. In the copy number profiles, bins are ordered along the x-axis by their genomic positions, and y-axis shows median-normalized $\log_2$-transformed data. Small triangles at the top and bottom edges represent data points that fall outside the plot area. Top-left corners show the number and size of bins. Top-right corner of the read counts shows the total number of sequence reads, and the top-right corners of the copy number profiles the expected and measured standard deviation. The expected standard deviation ($\mathrm{E}\ \sigma$) is defined as $\sqrt{1/N}$, where N is the average number of reads per bin. The measured standard deviation ($\hat{\sigma}_\Delta$) is calculated from the data with a 0.1%-trimmed first-order estimate (prior to $\log_2$-transforming the data for plotting).

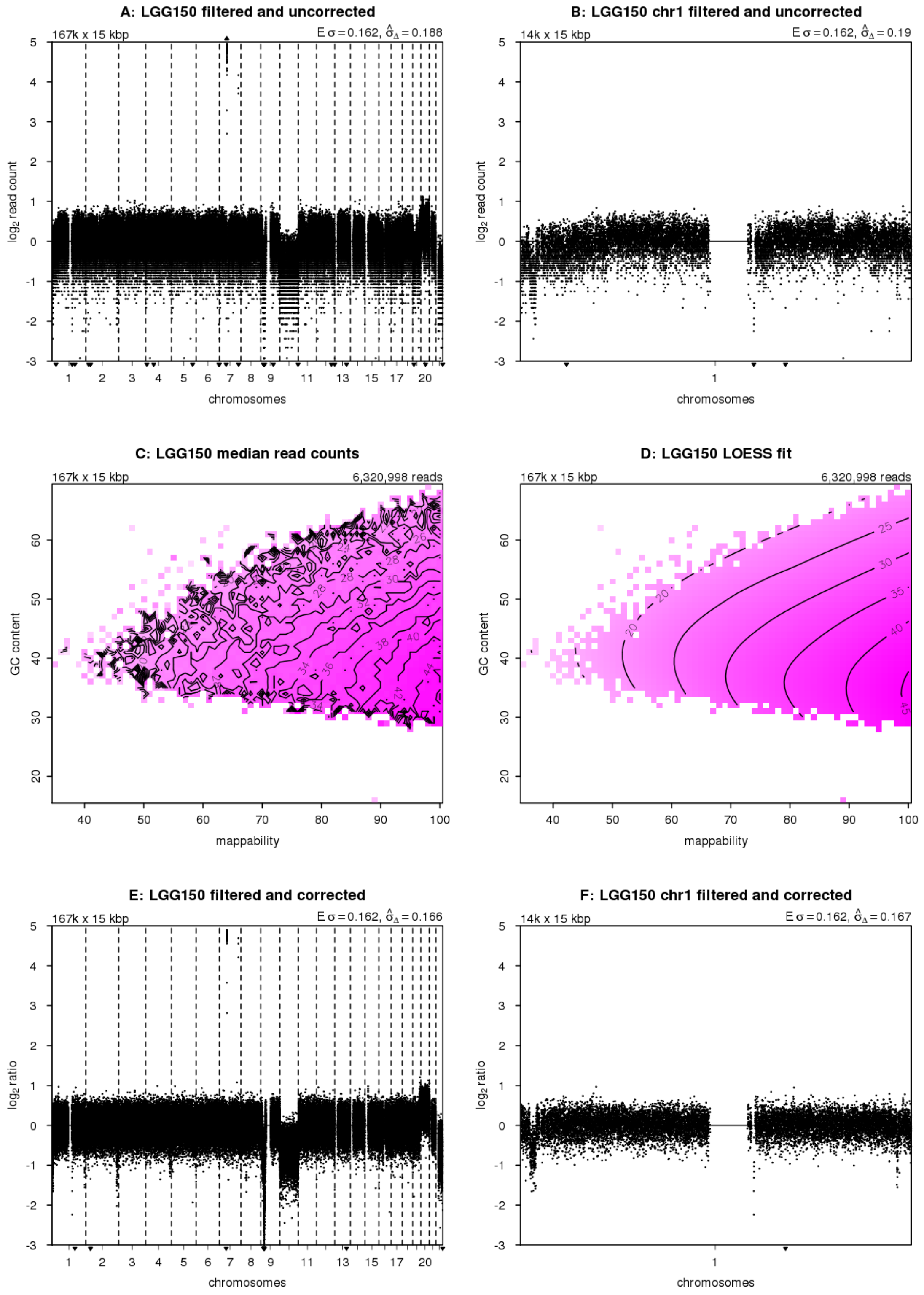

Figure S2: Corrections to filtered read counts (cont.)

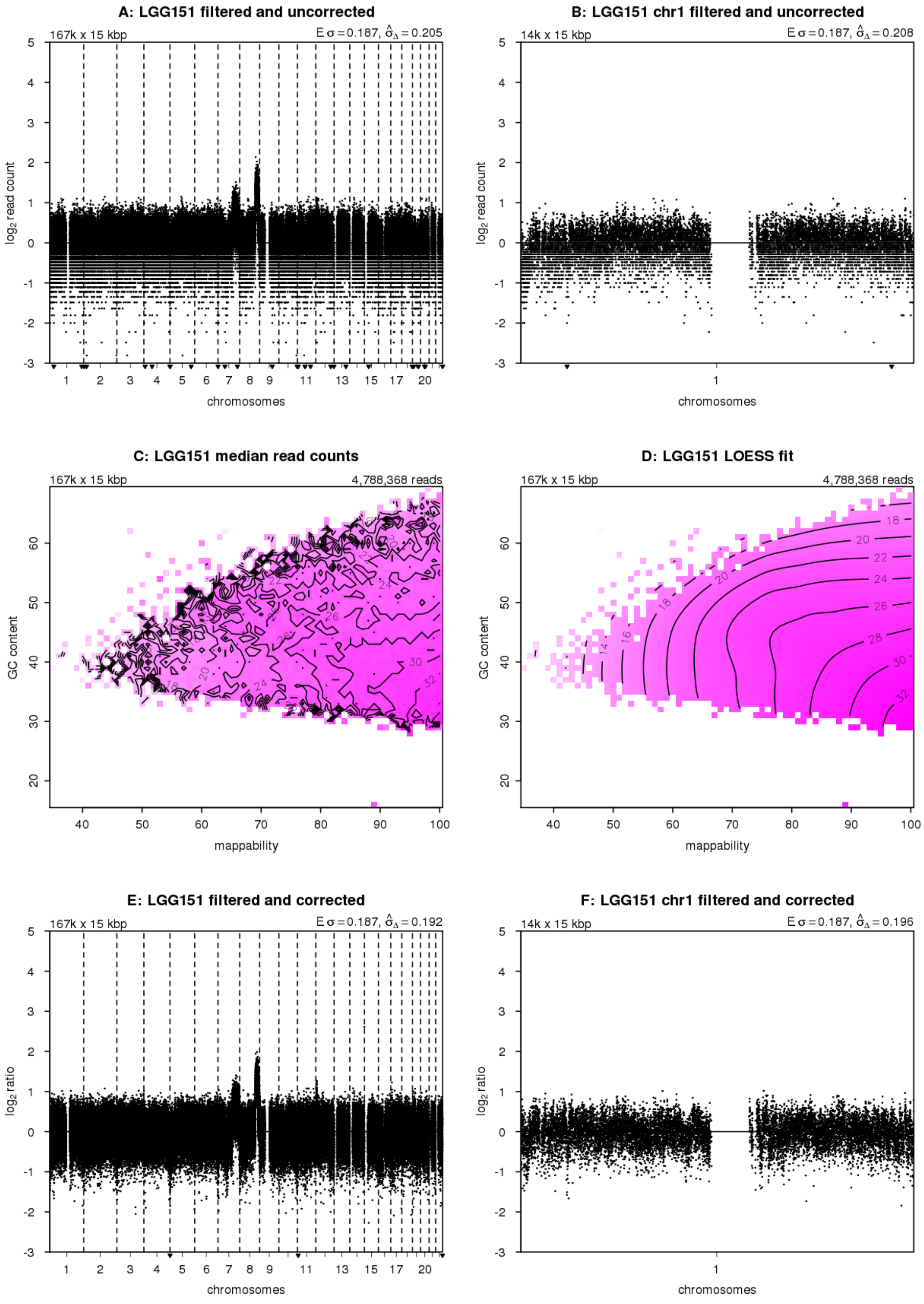

Figure S2: Corrections to filtered read counts (cont.)

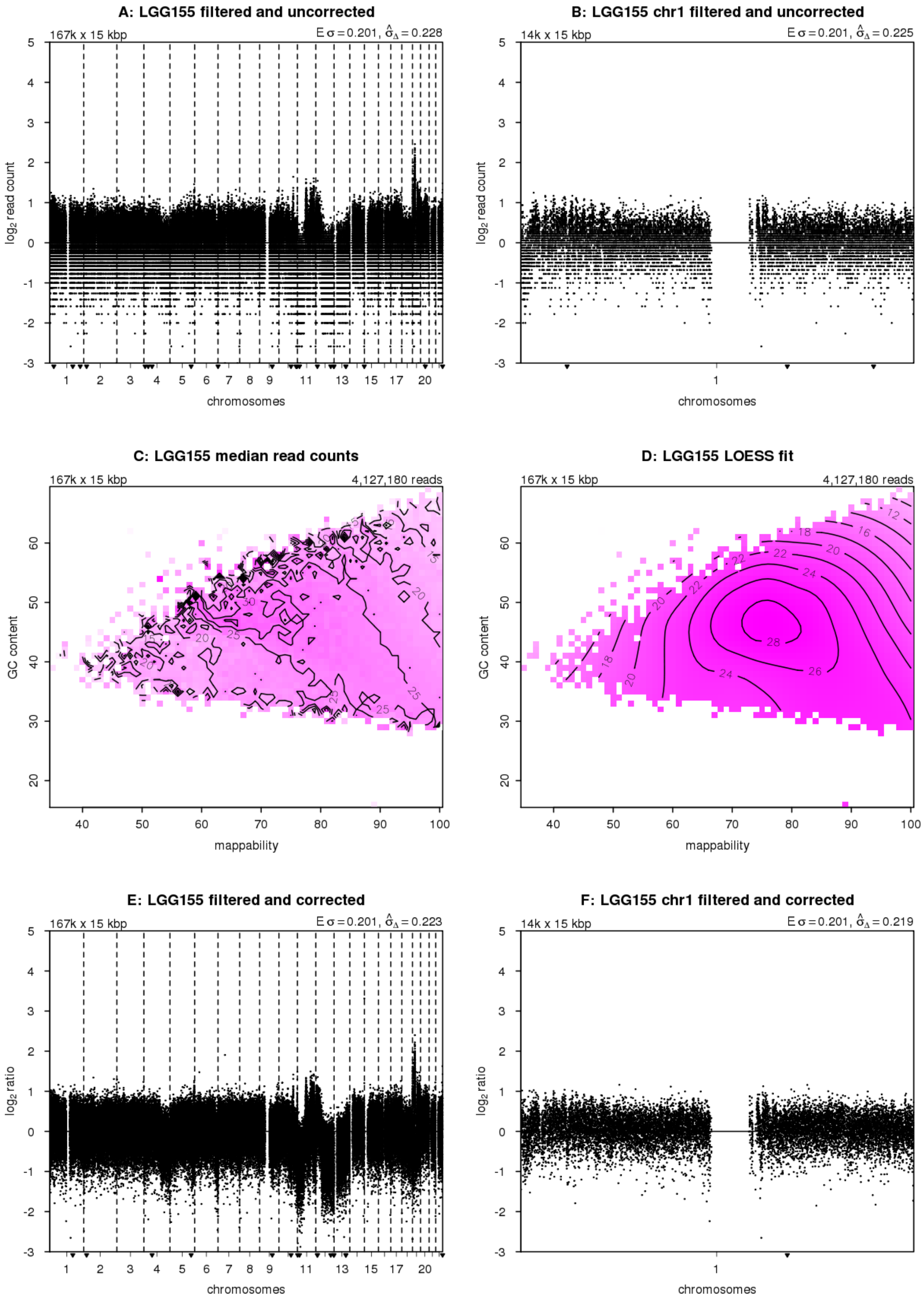

Figure S2: Corrections to filtered read counts (cont.)

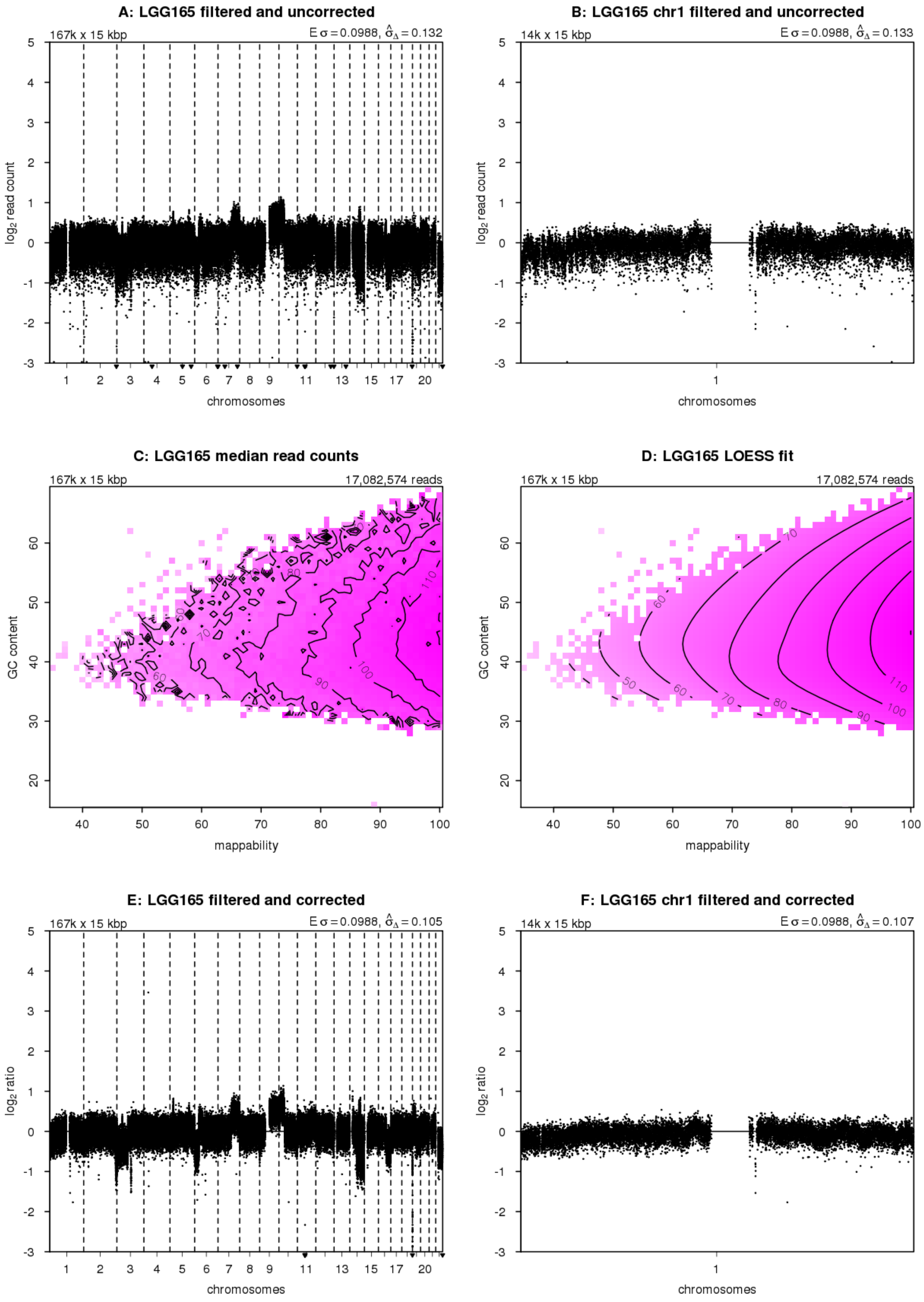

Figure S2: Corrections to filtered read counts (cont.)

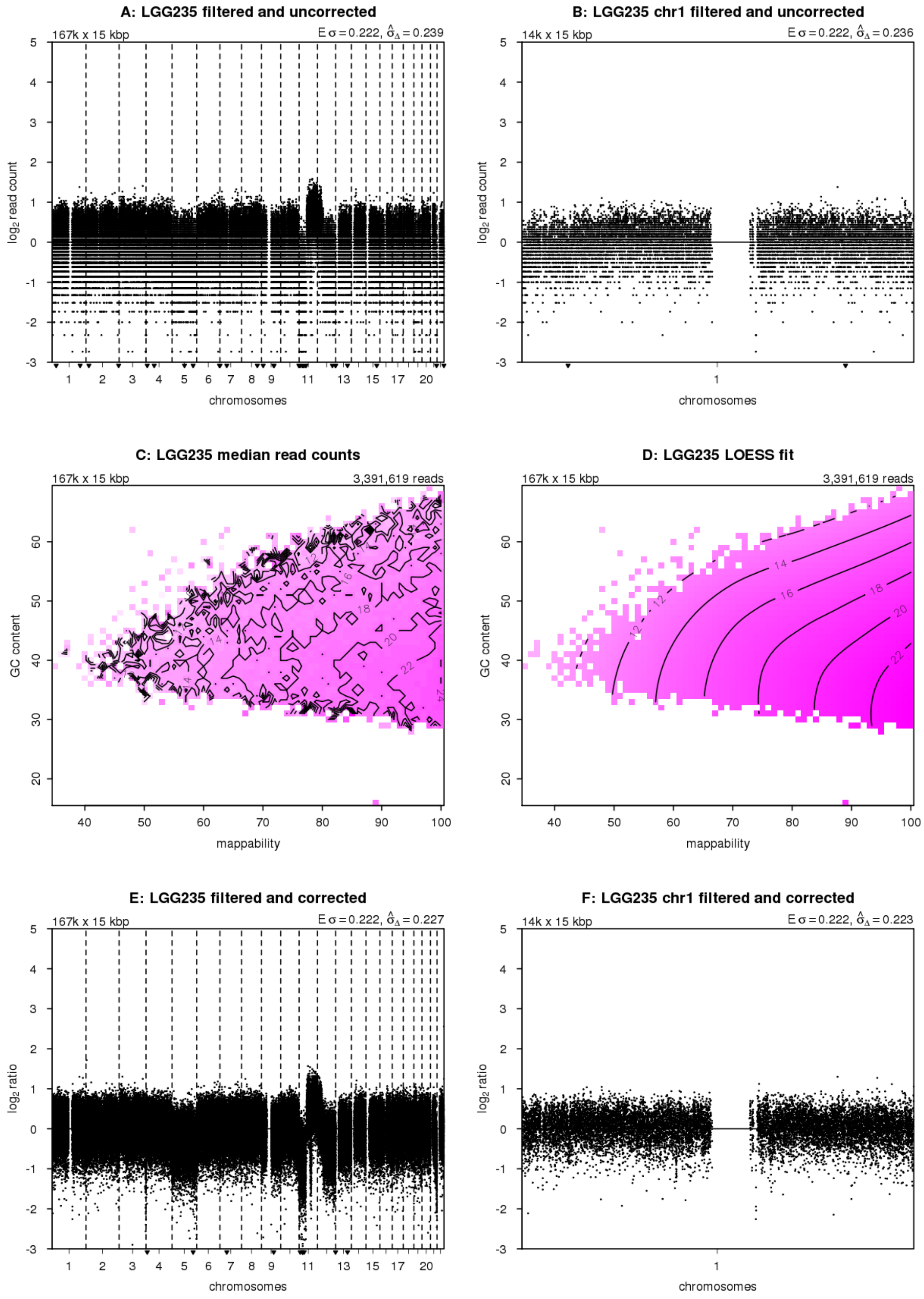

Figure S2: Corrections to filtered read counts (cont.)

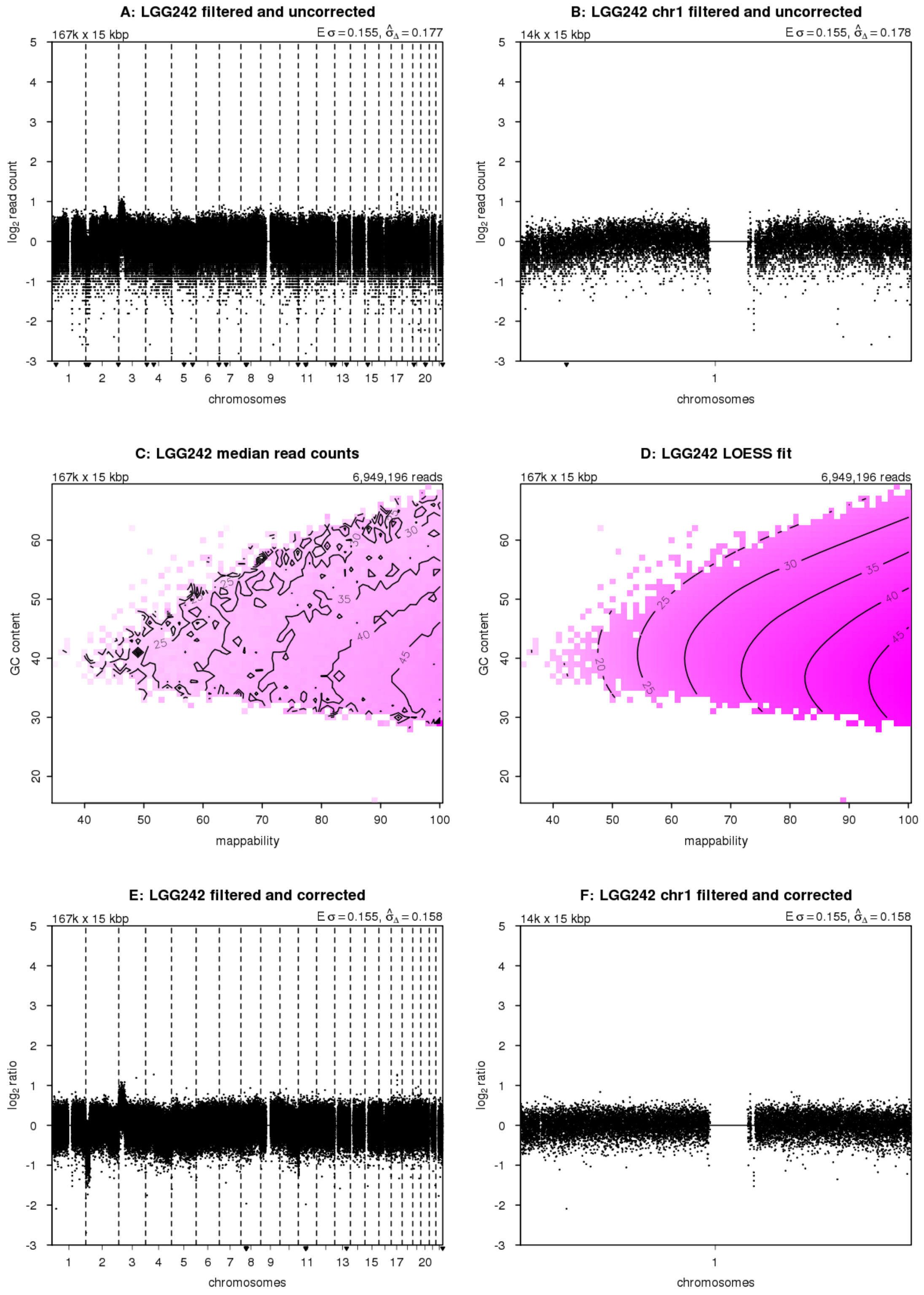

Figure S2: Corrections to filtered read counts (cont.)

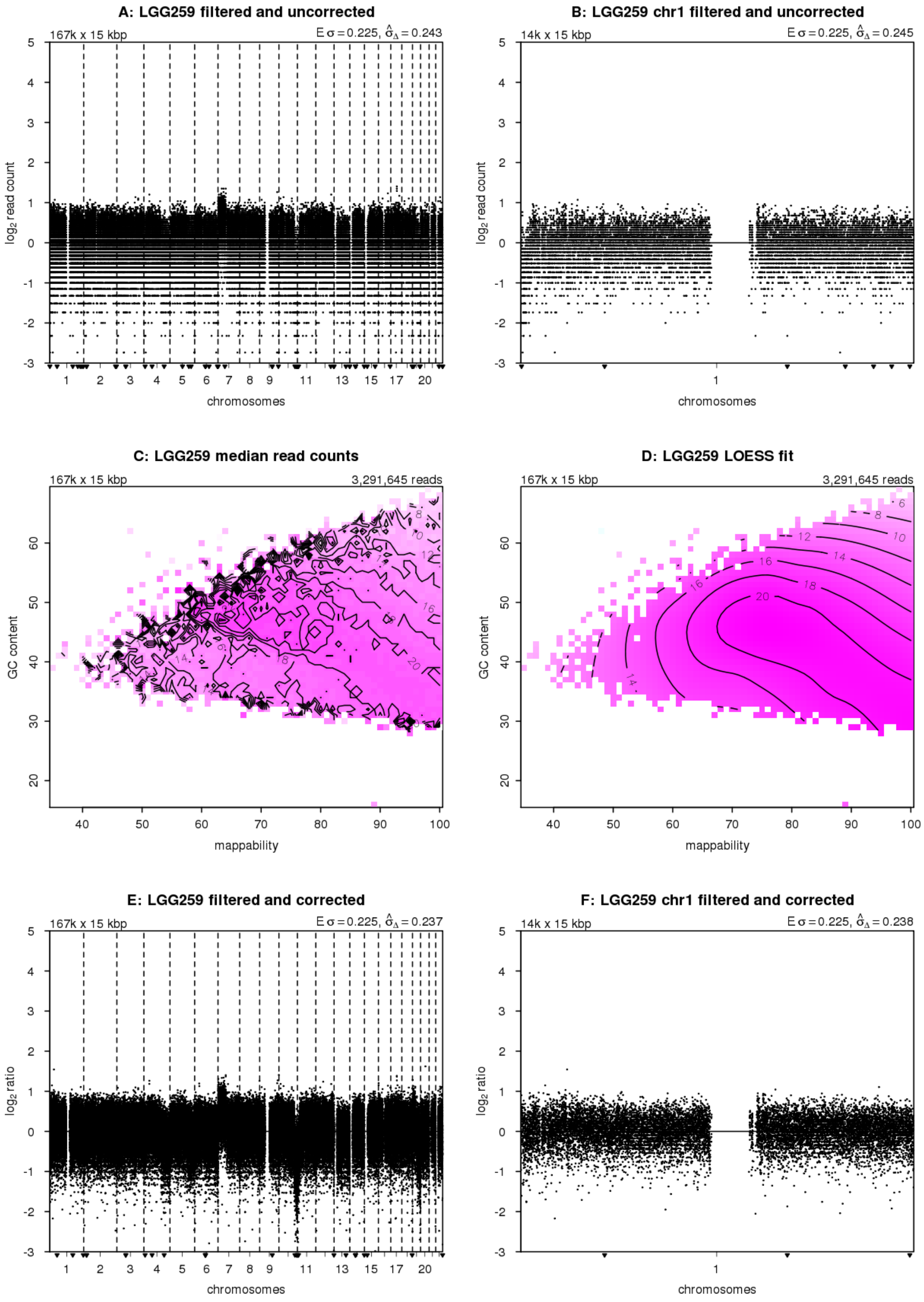

Figure S2: Corrections to filtered read counts (cont.)

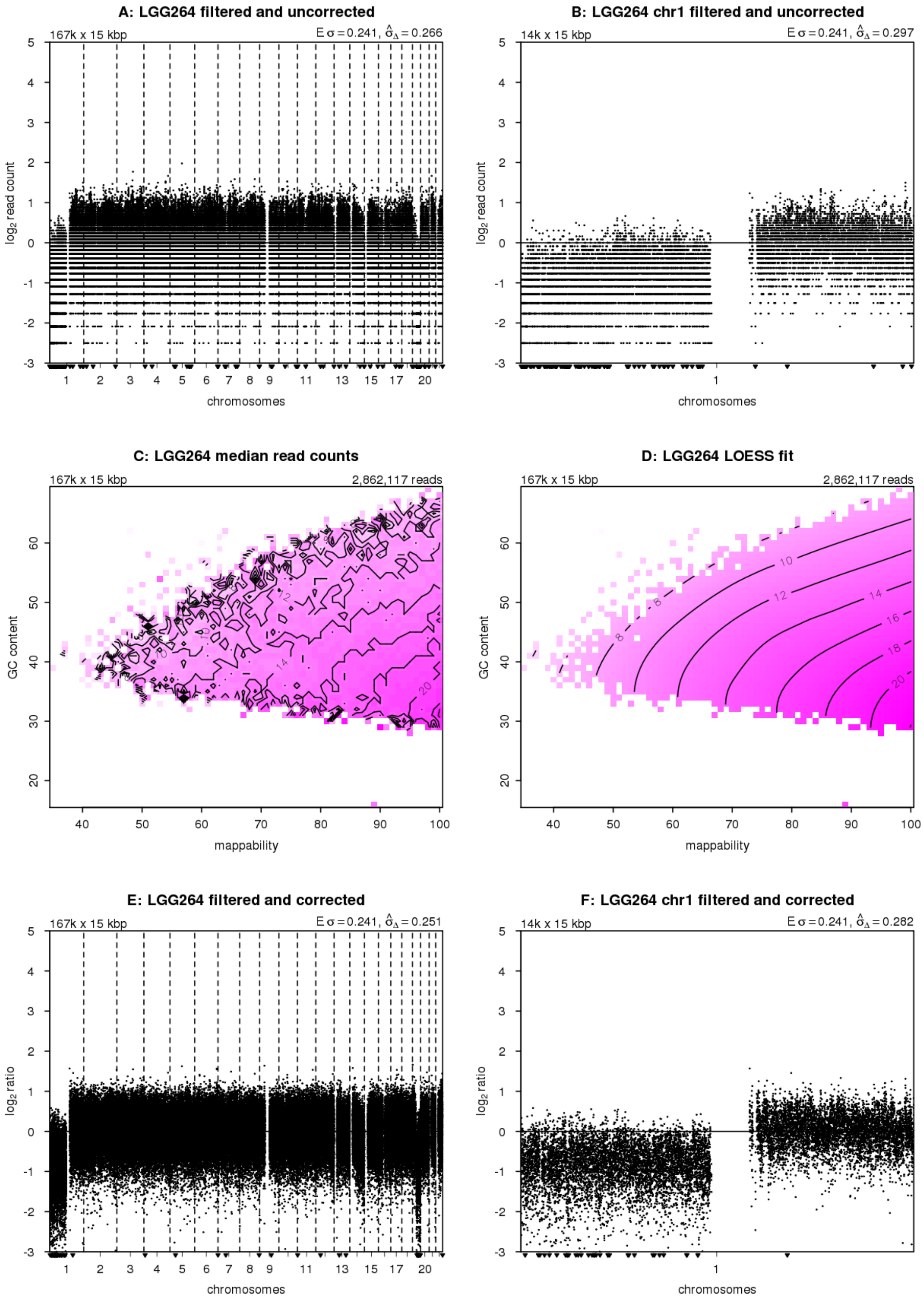

Figure S2: Corrections to filtered read counts (cont.)

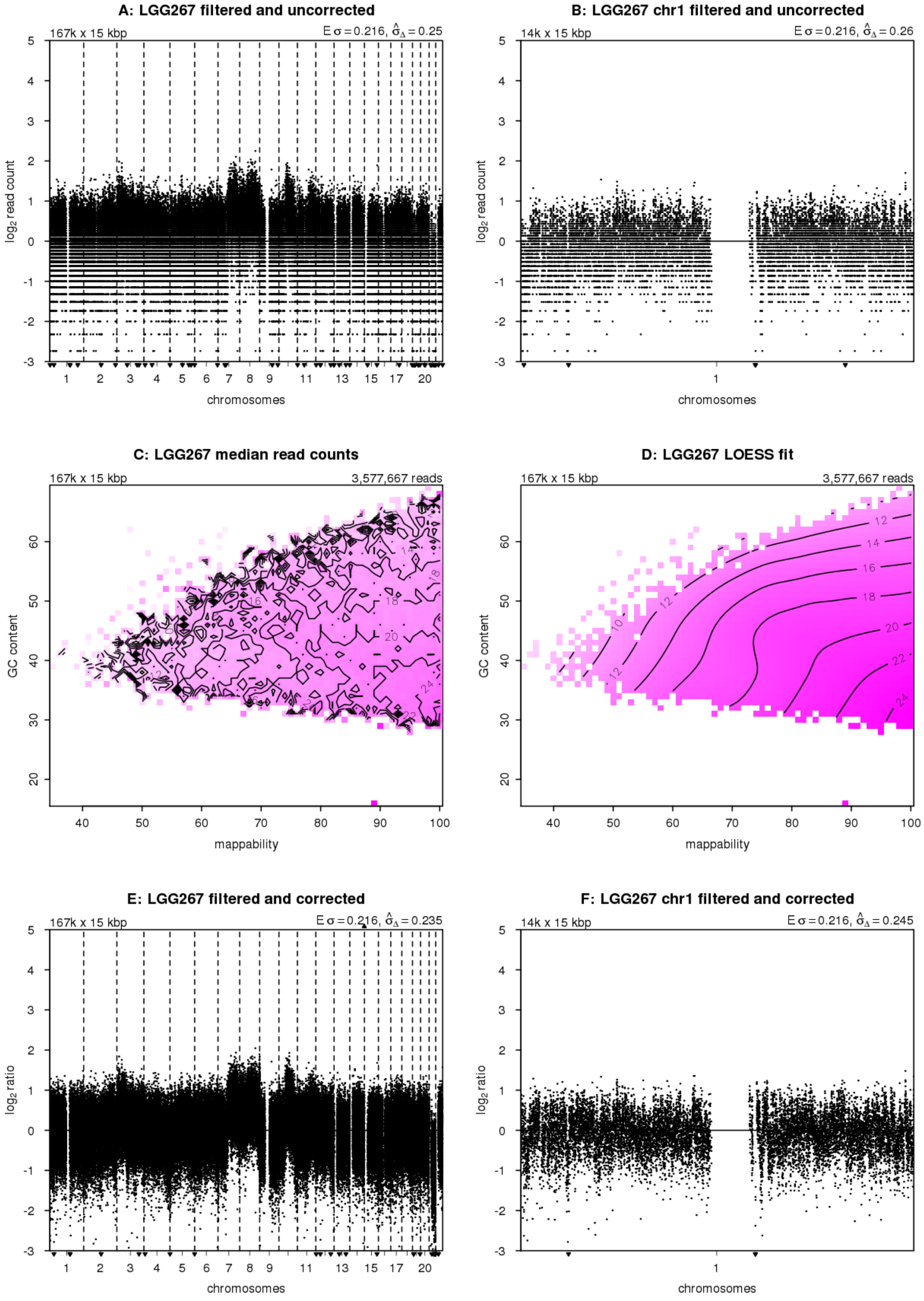

Figure S2: Corrections to filtered read counts (cont.)

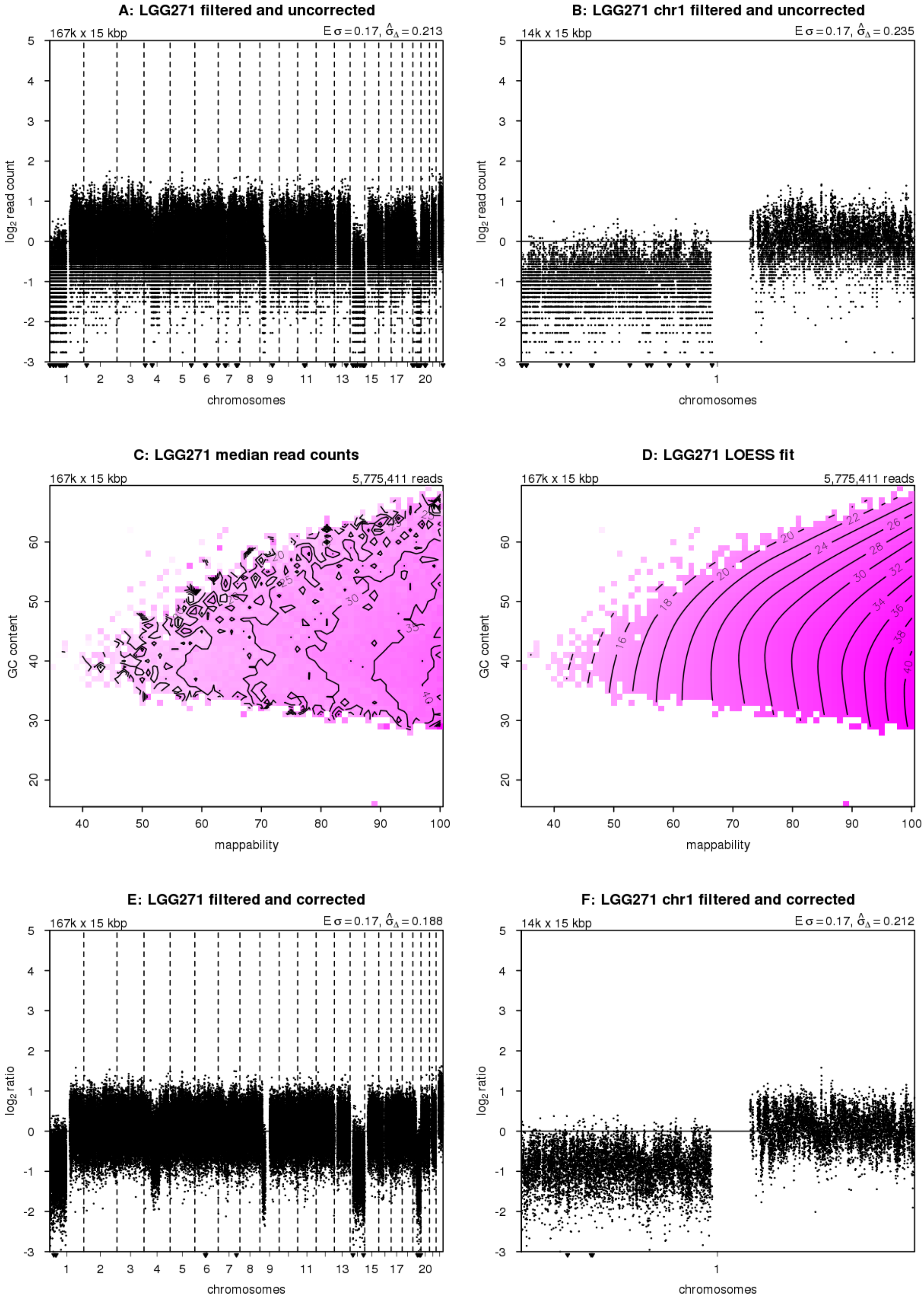

Figure S2: Corrections to filtered read counts (cont.)

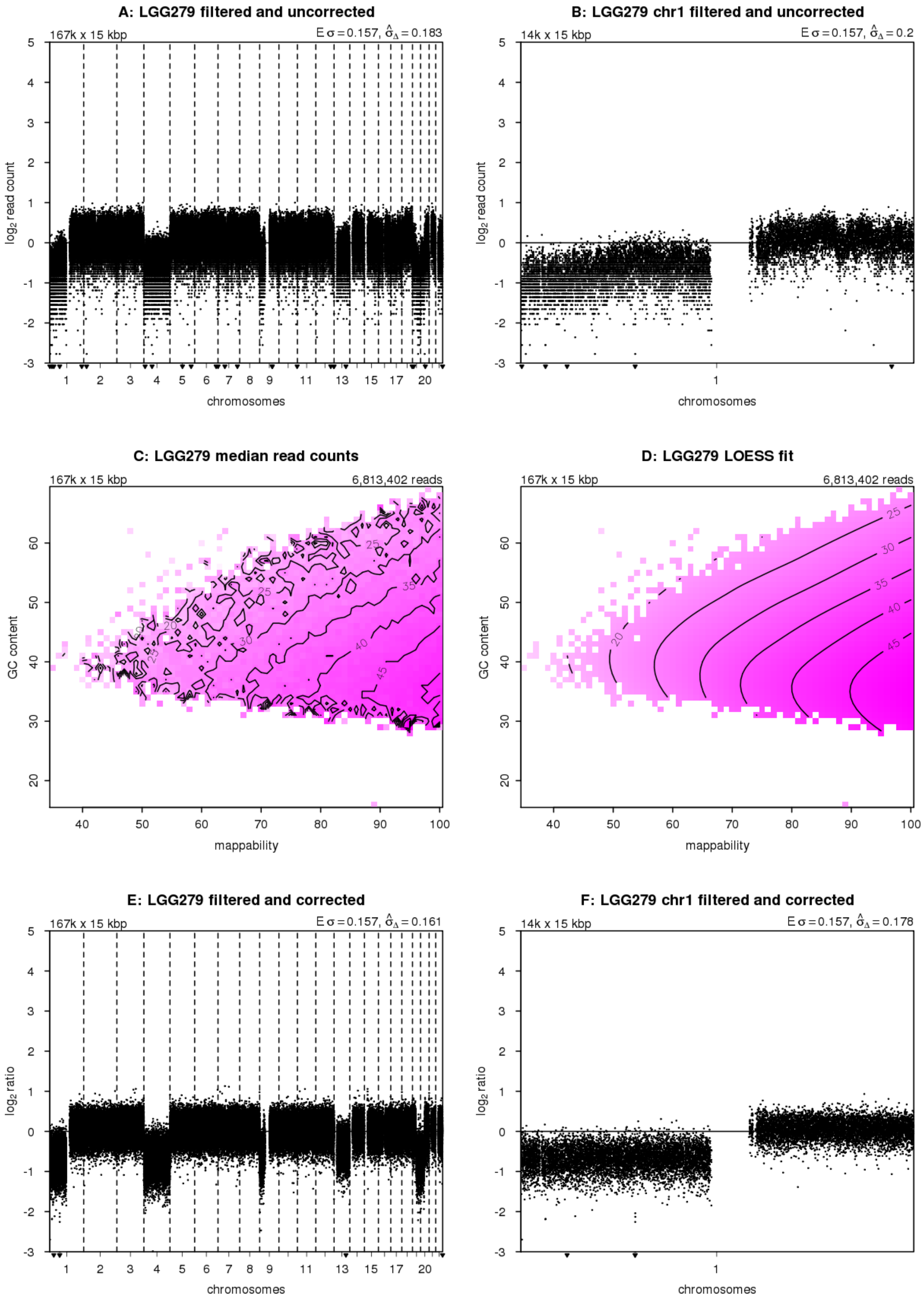

Figure S2: Corrections to filtered read counts (cont.)

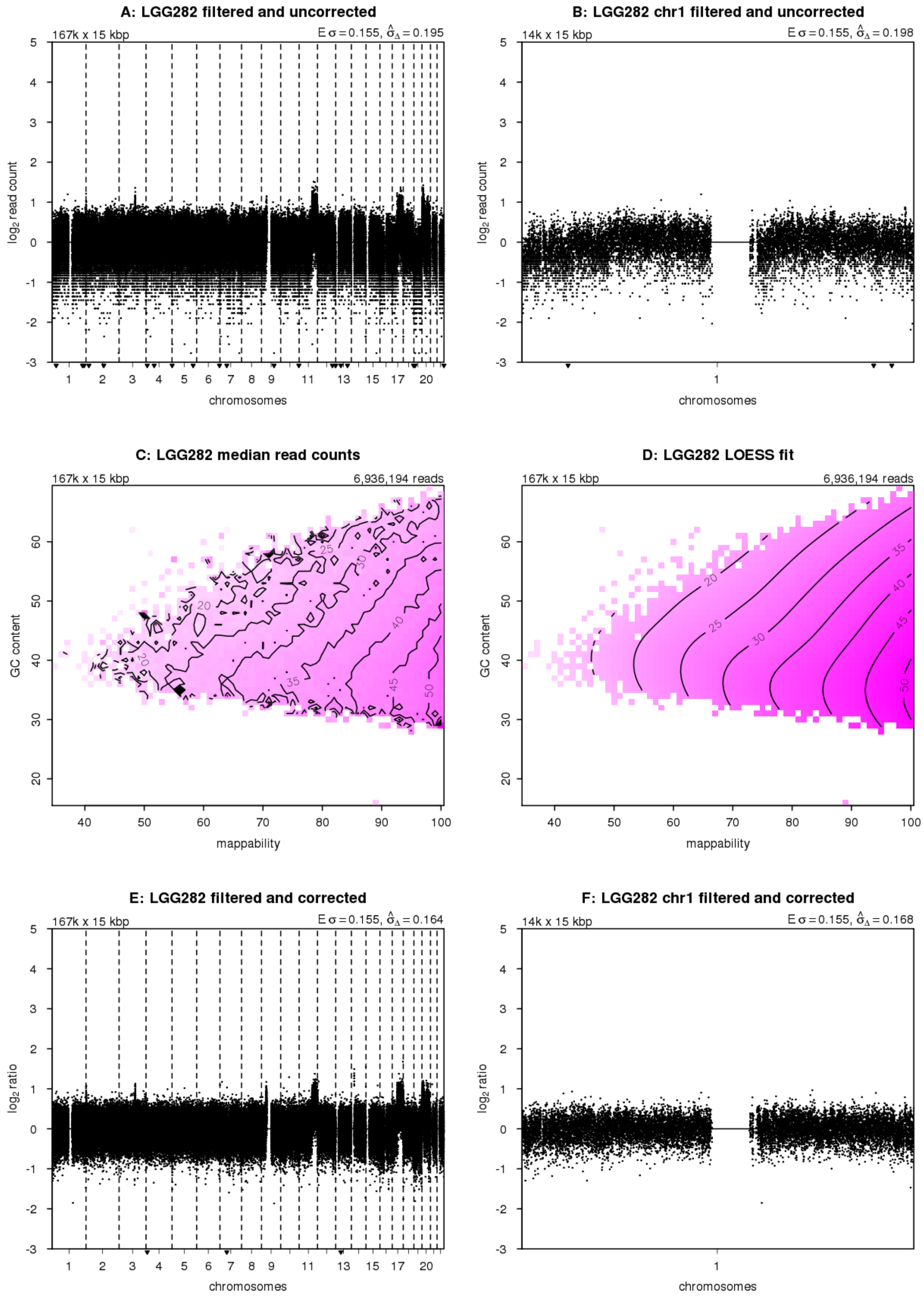

Figure S2: Corrections to filtered read counts (cont.)

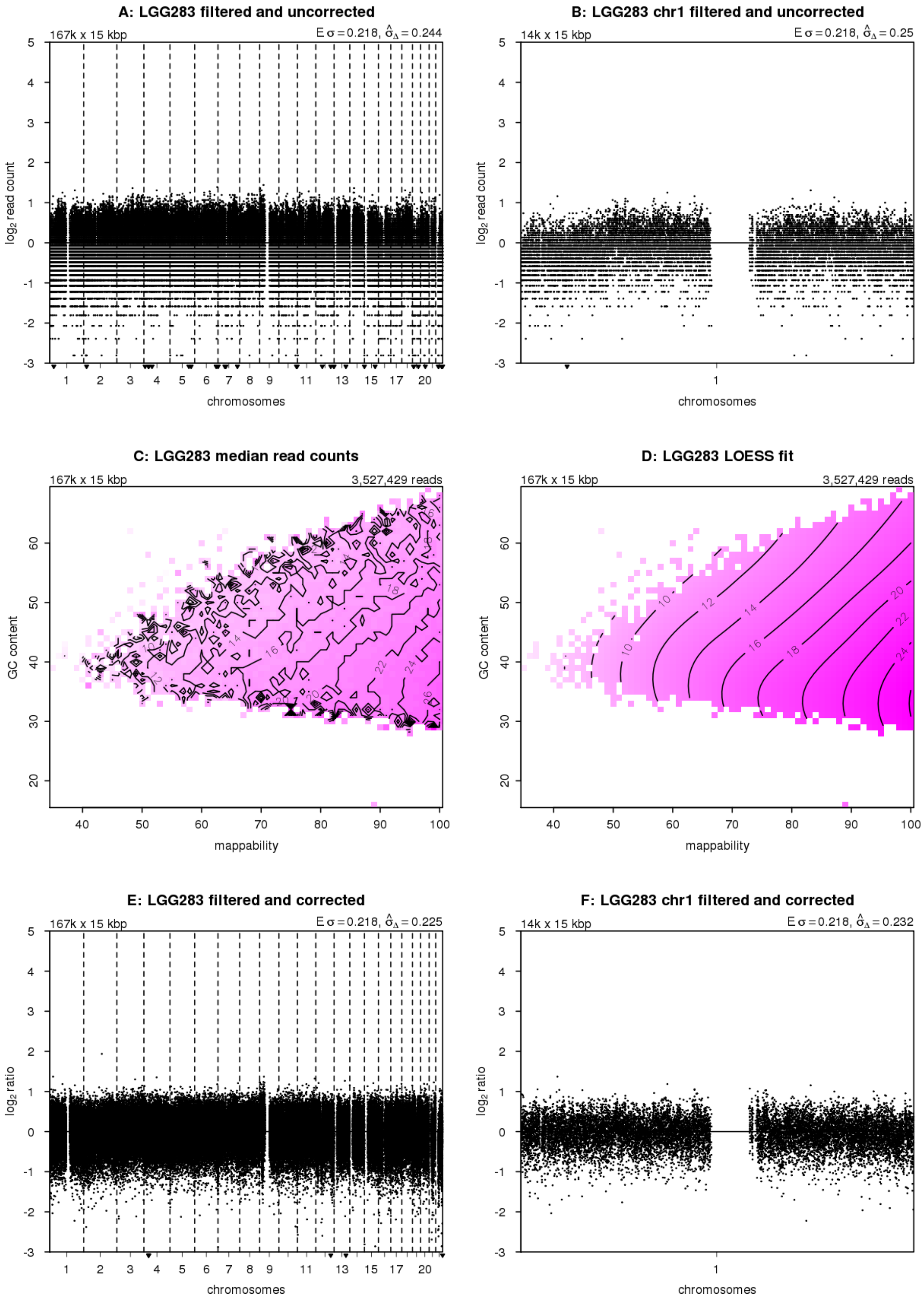

Figure S2: Corrections to filtered read counts (cont.)

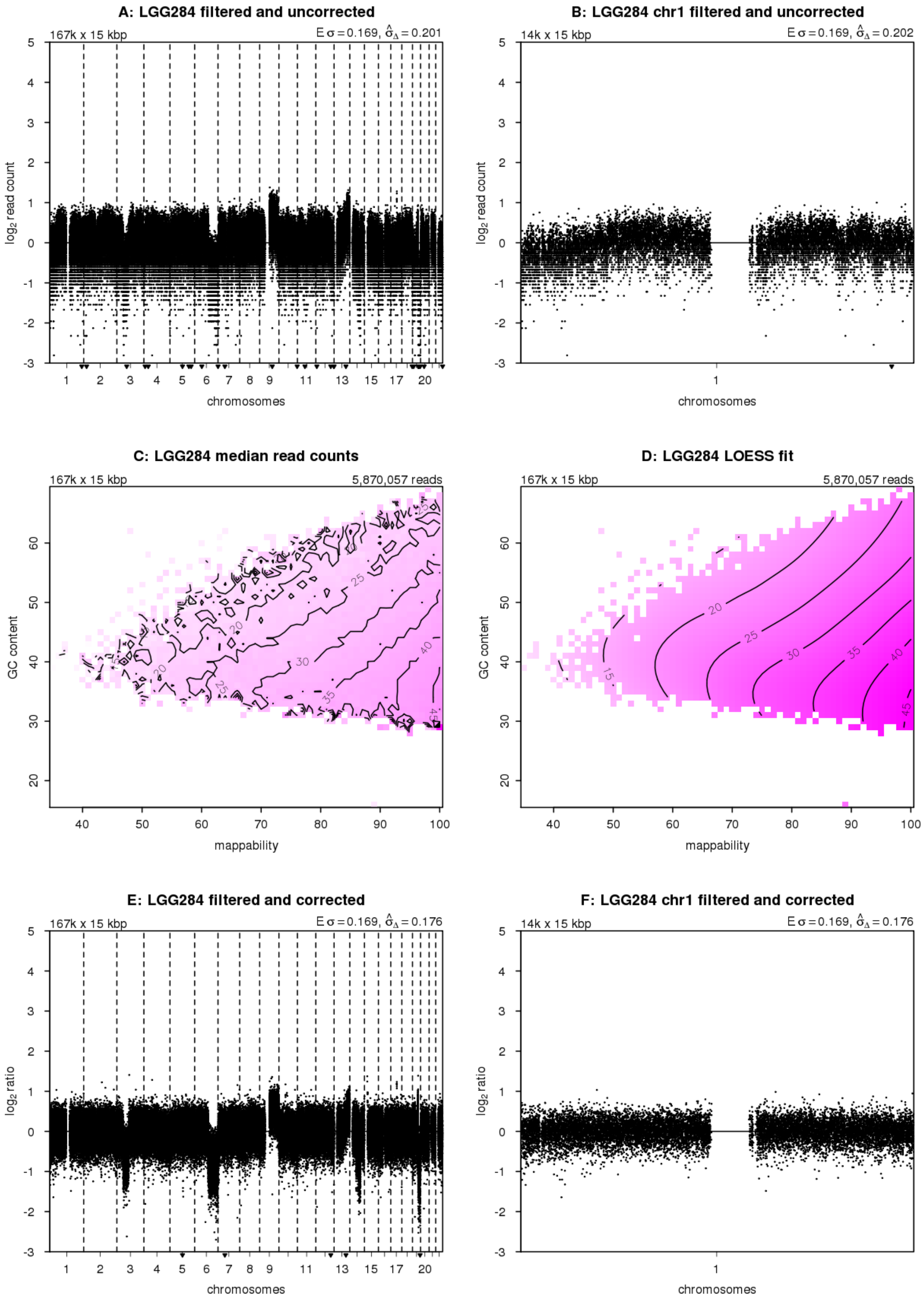

Figure S2: Corrections to filtered read counts (cont.)

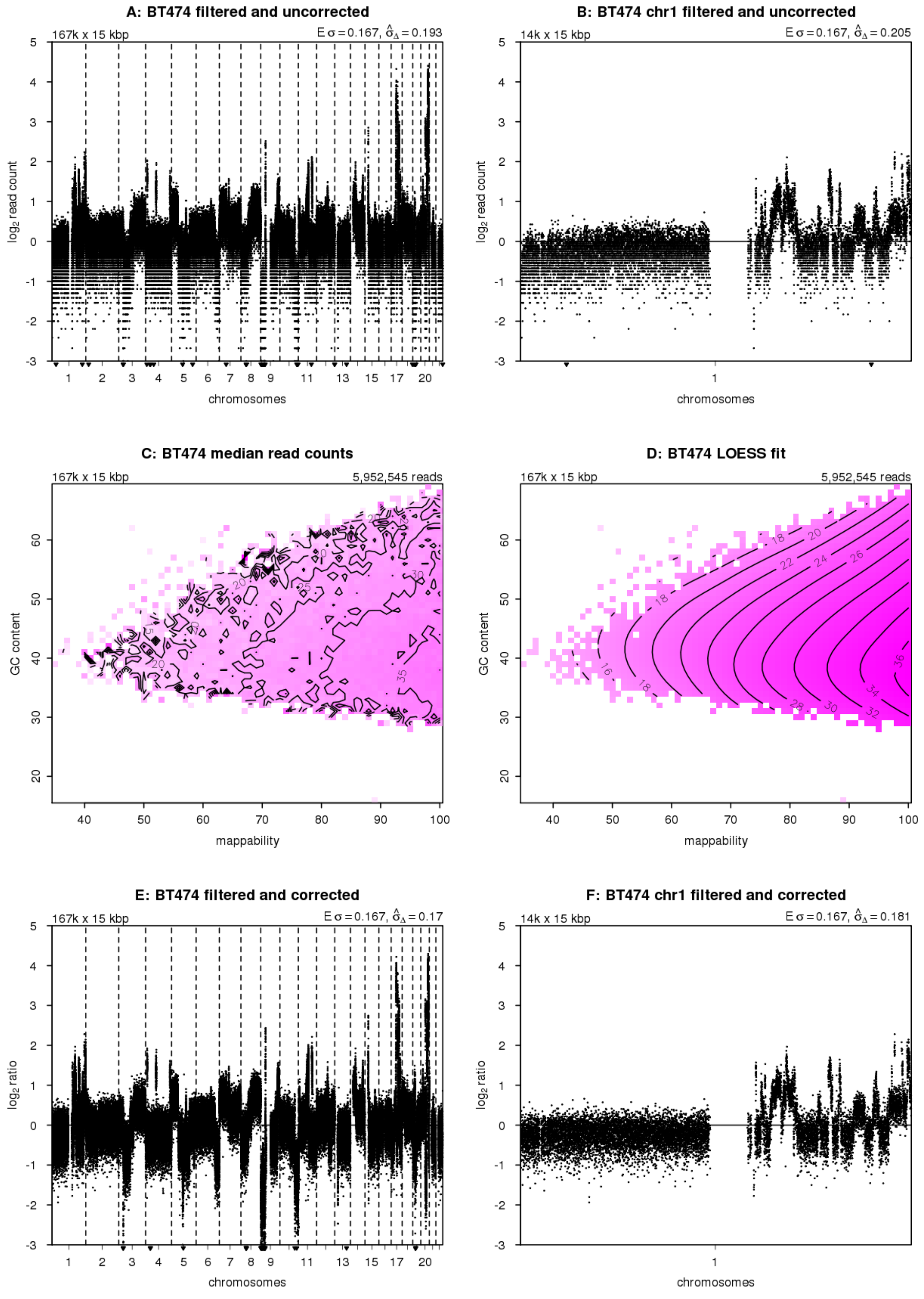

Figure S2: Corrections to filtered read counts (cont.)

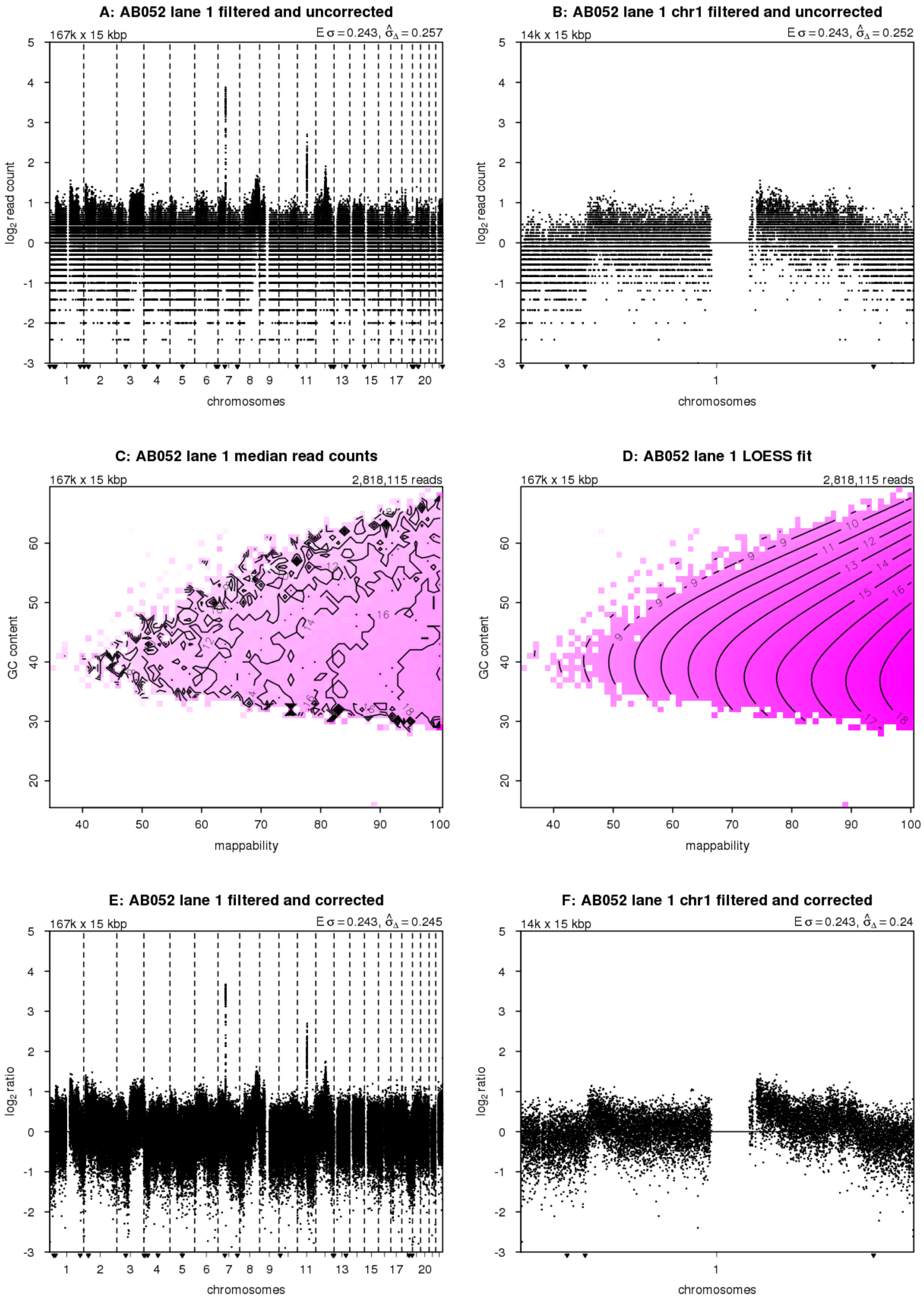

Figure S2: Corrections to filtered read counts (cont.)

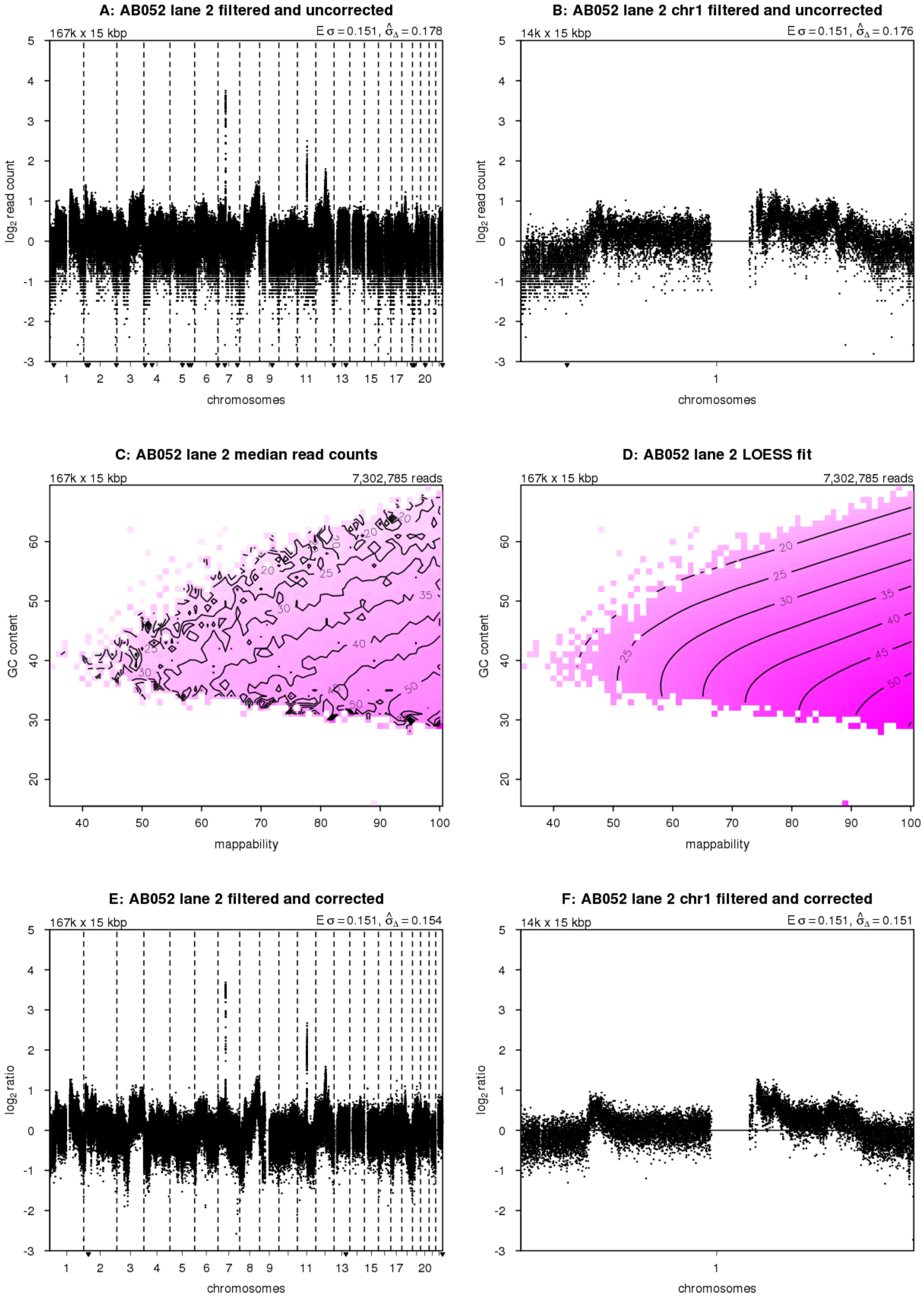

Figure S2: Corrections to filtered read counts (cont.)

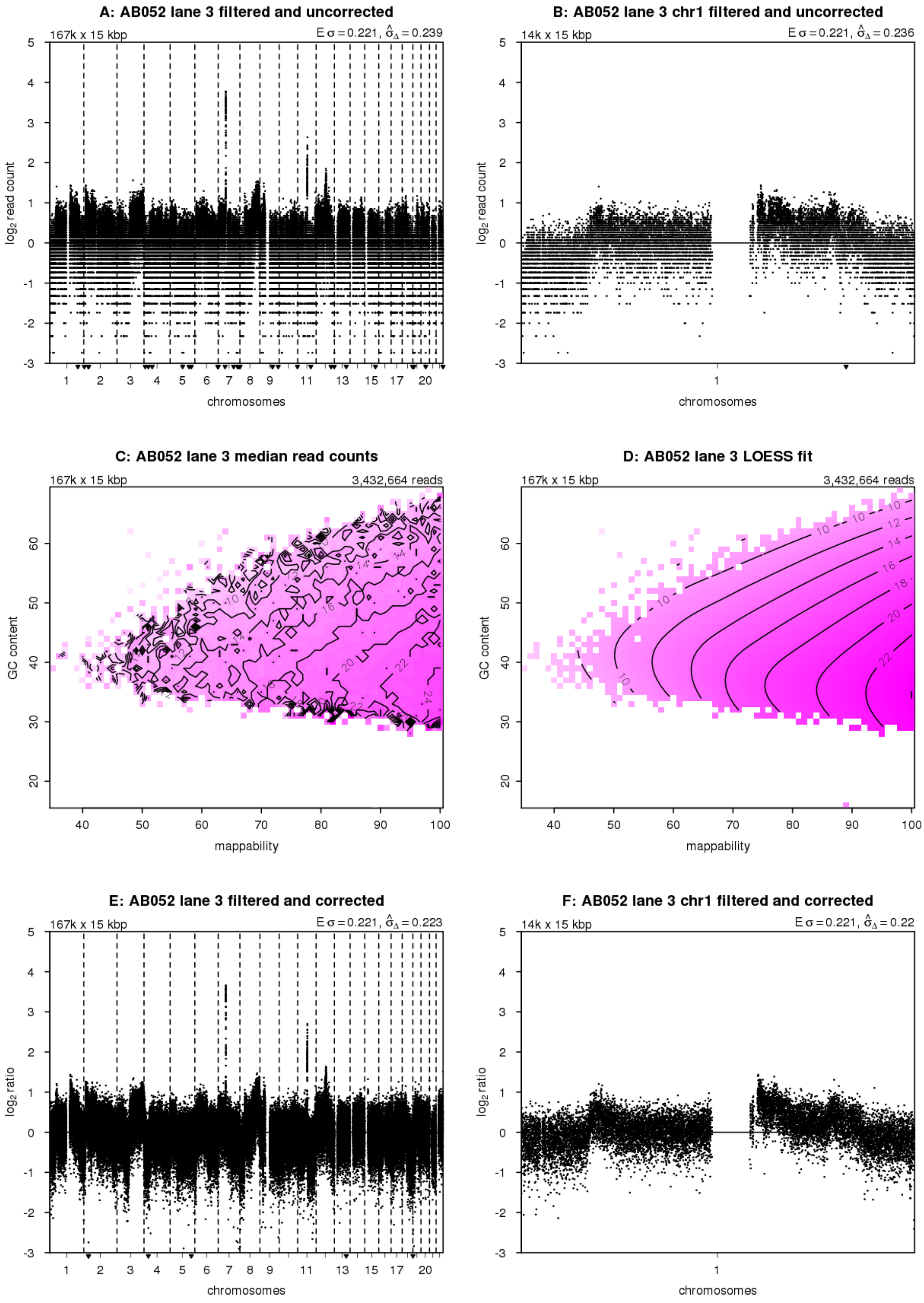

Figure S2: Corrections to filtered read counts (cont.)

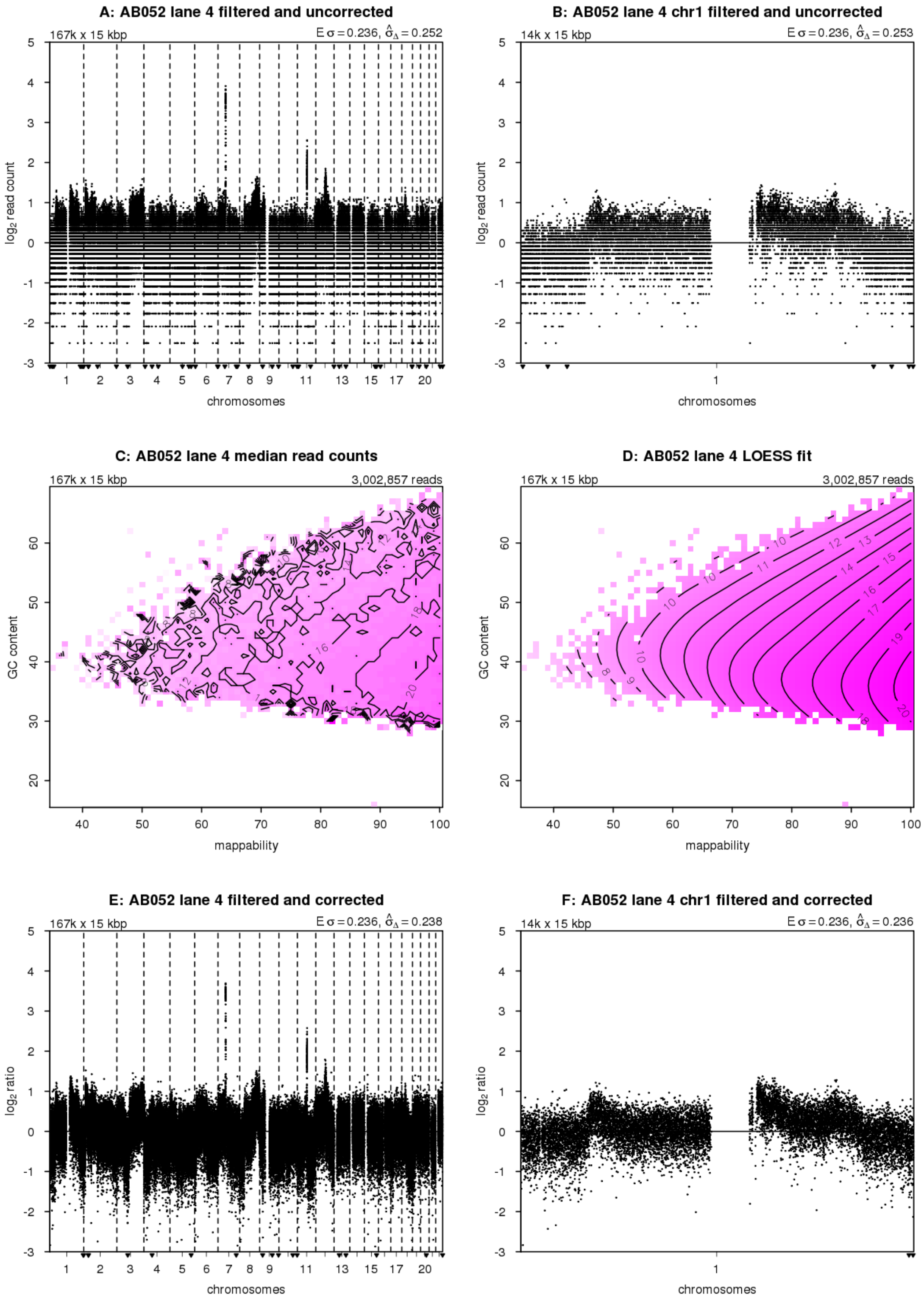

Figure S2: Corrections to filtered read counts (cont.)

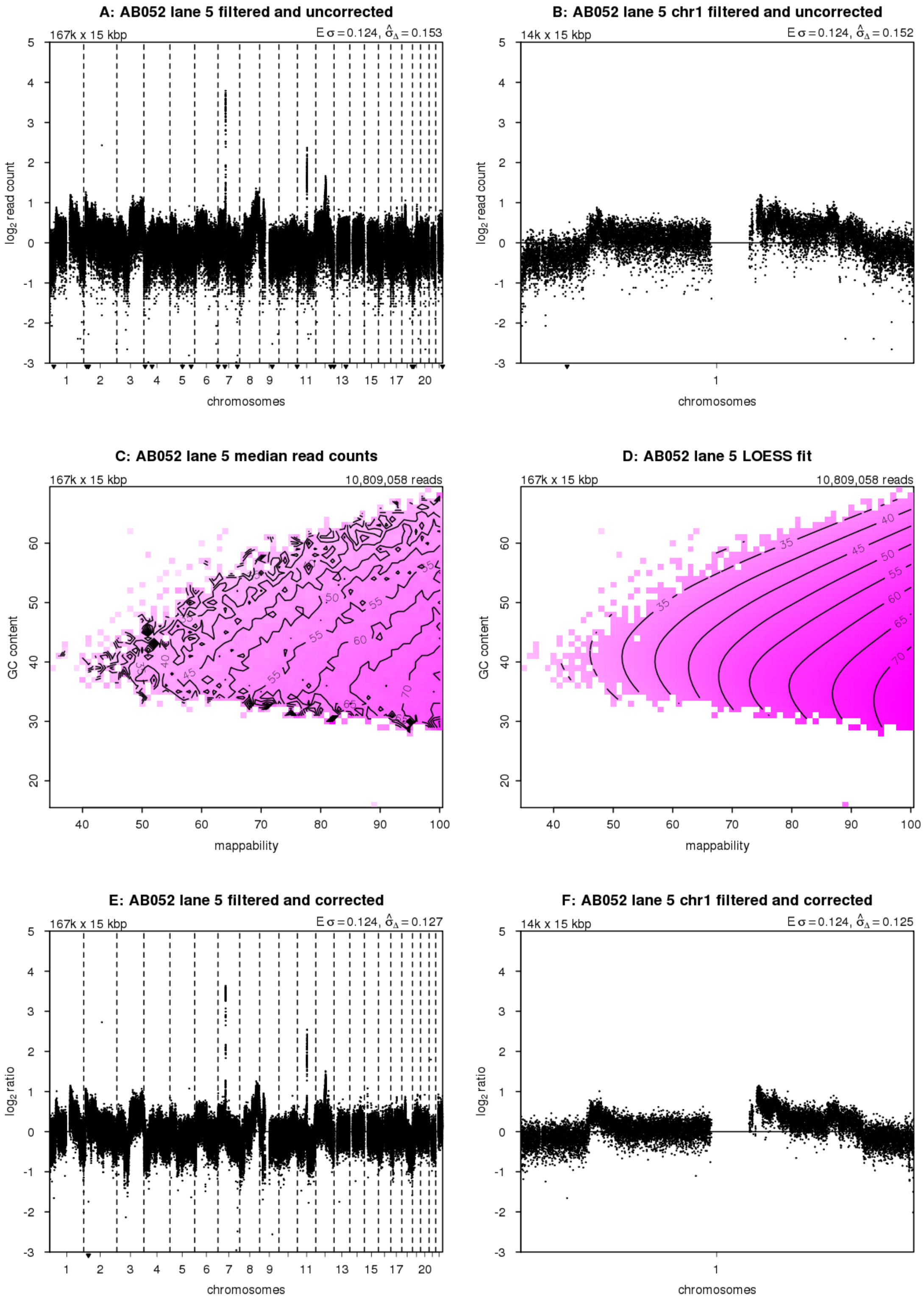

Figure S2: Corrections to filtered read counts (cont.)

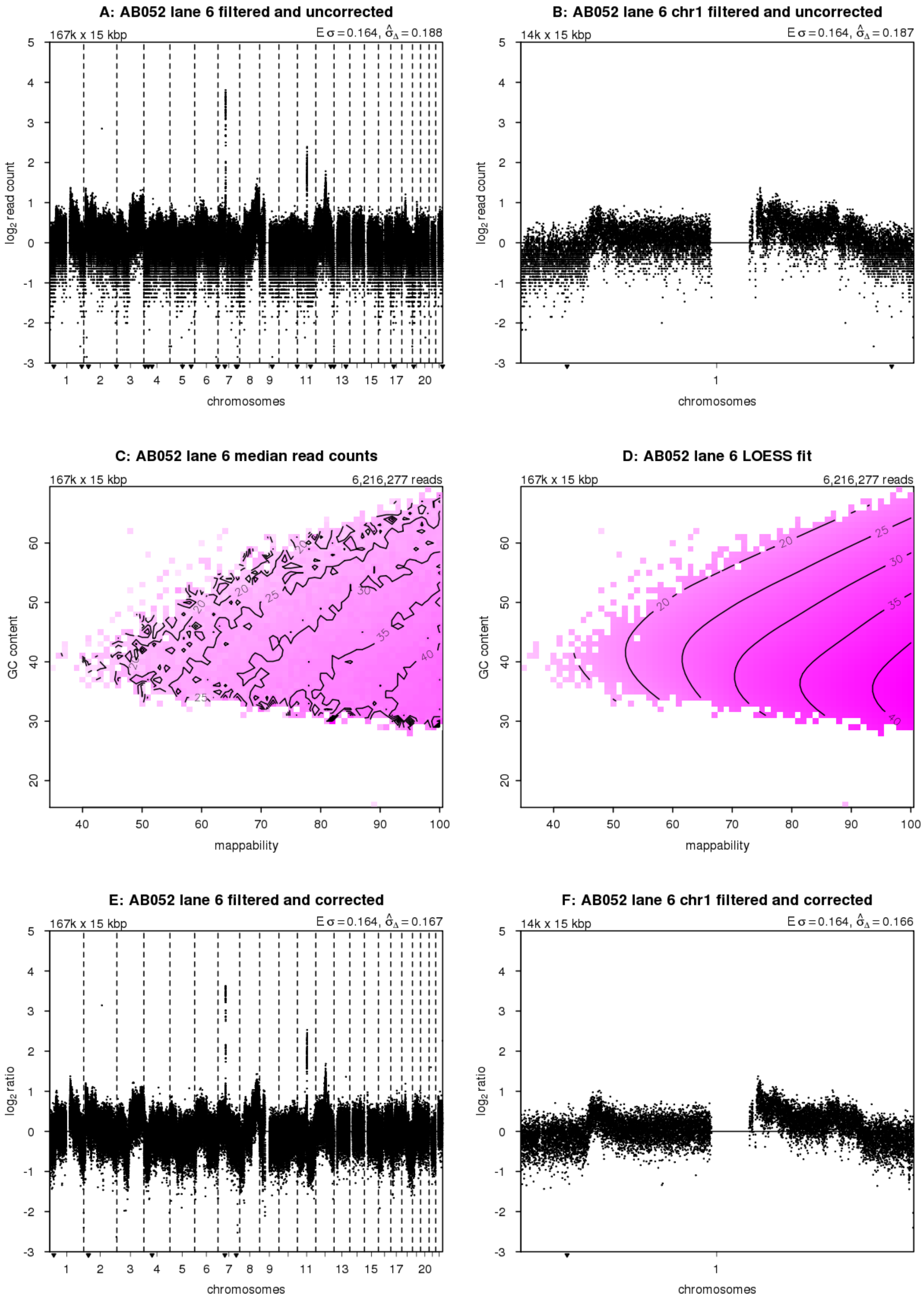

Figure S2: Corrections to filtered read counts (cont.)

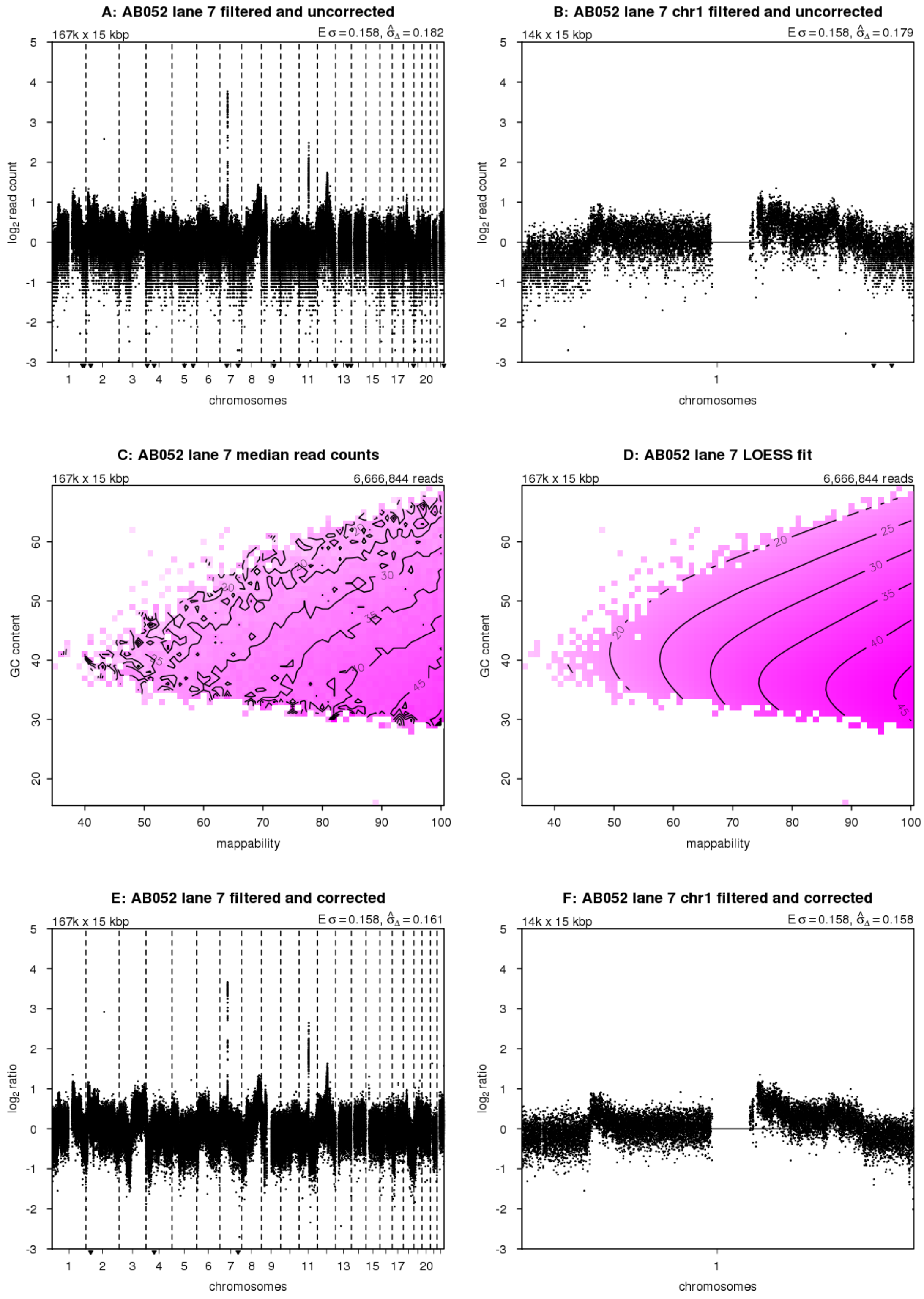

Figure S2: Corrections to filtered read counts (cont.)

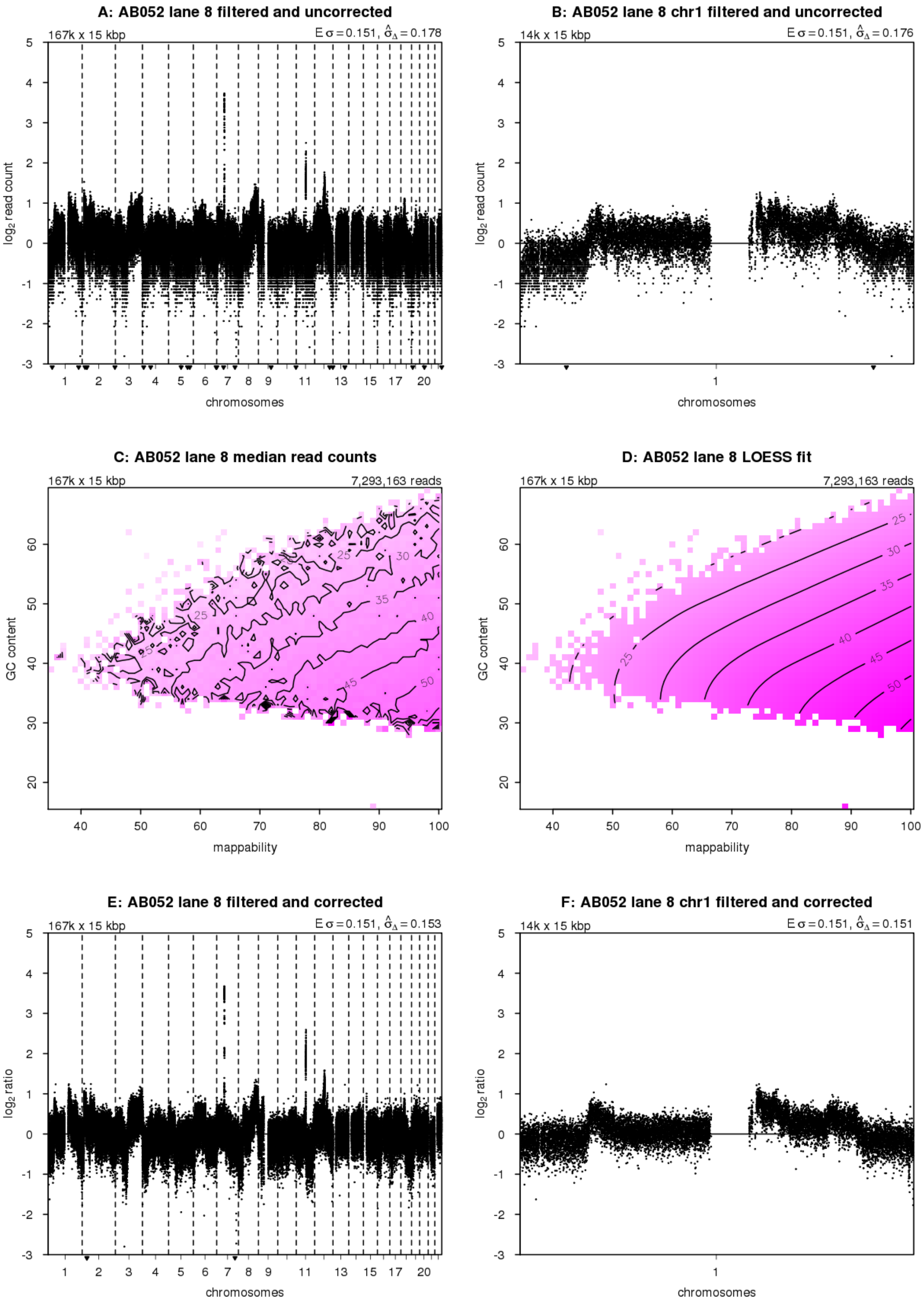

Figure S2: Corrections to filtered read counts (cont.)

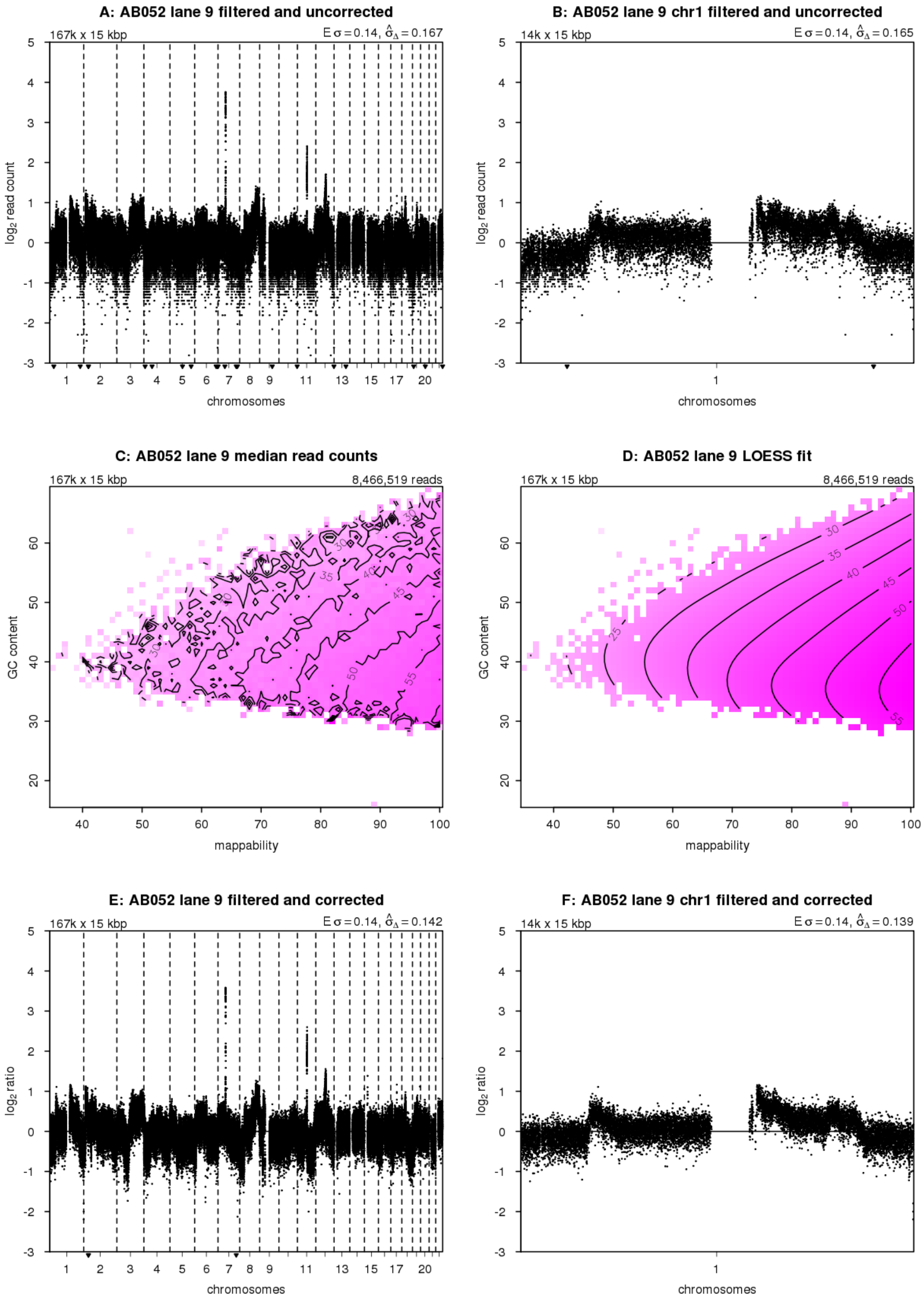

Figure S2: Corrections to filtered read counts (cont.)

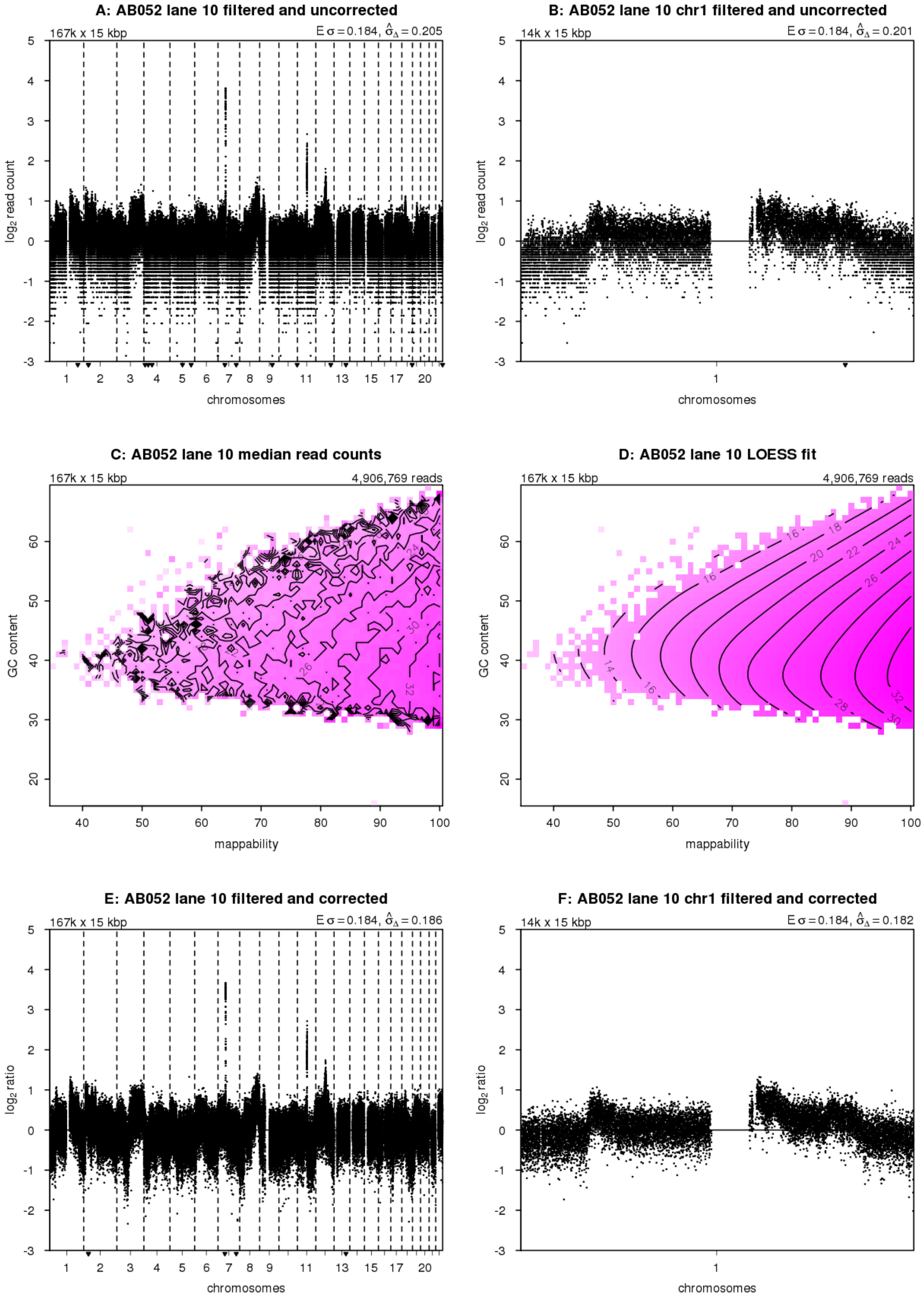

Figure S2: Corrections to filtered read counts (cont.)

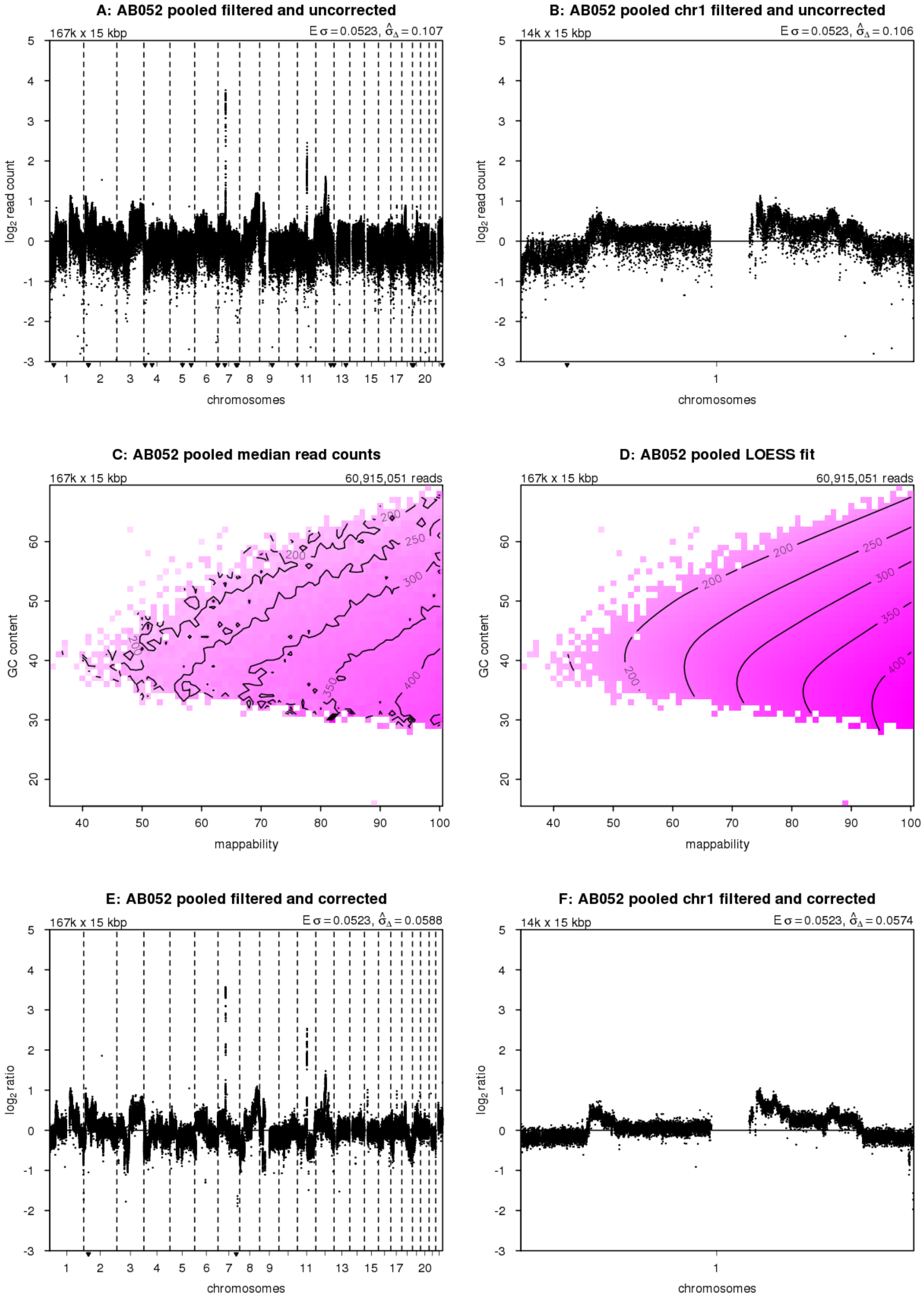

Figure S2: Corrections to filtered read counts (cont.)

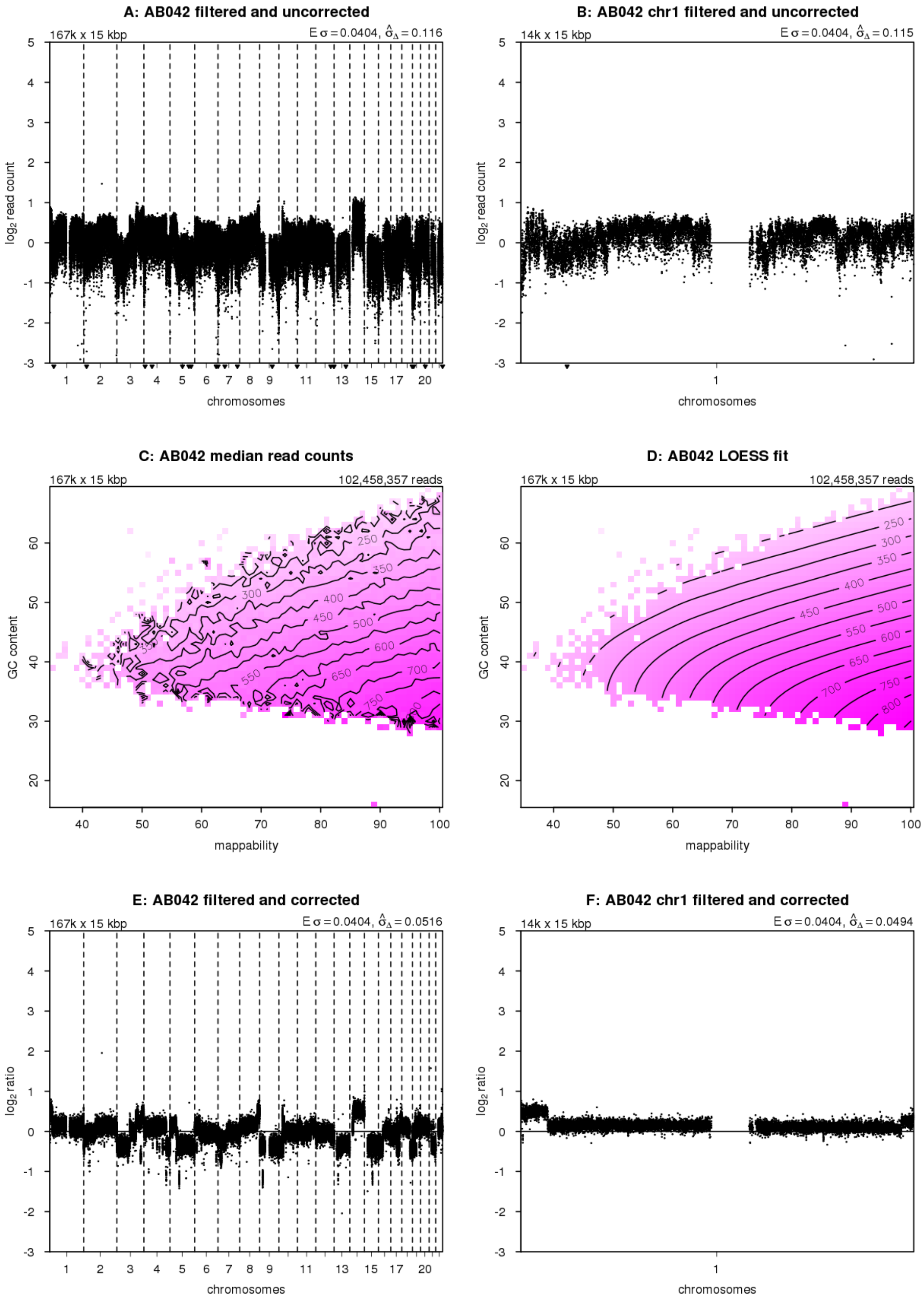

Figure S2: Corrections to filtered read counts (cont.)

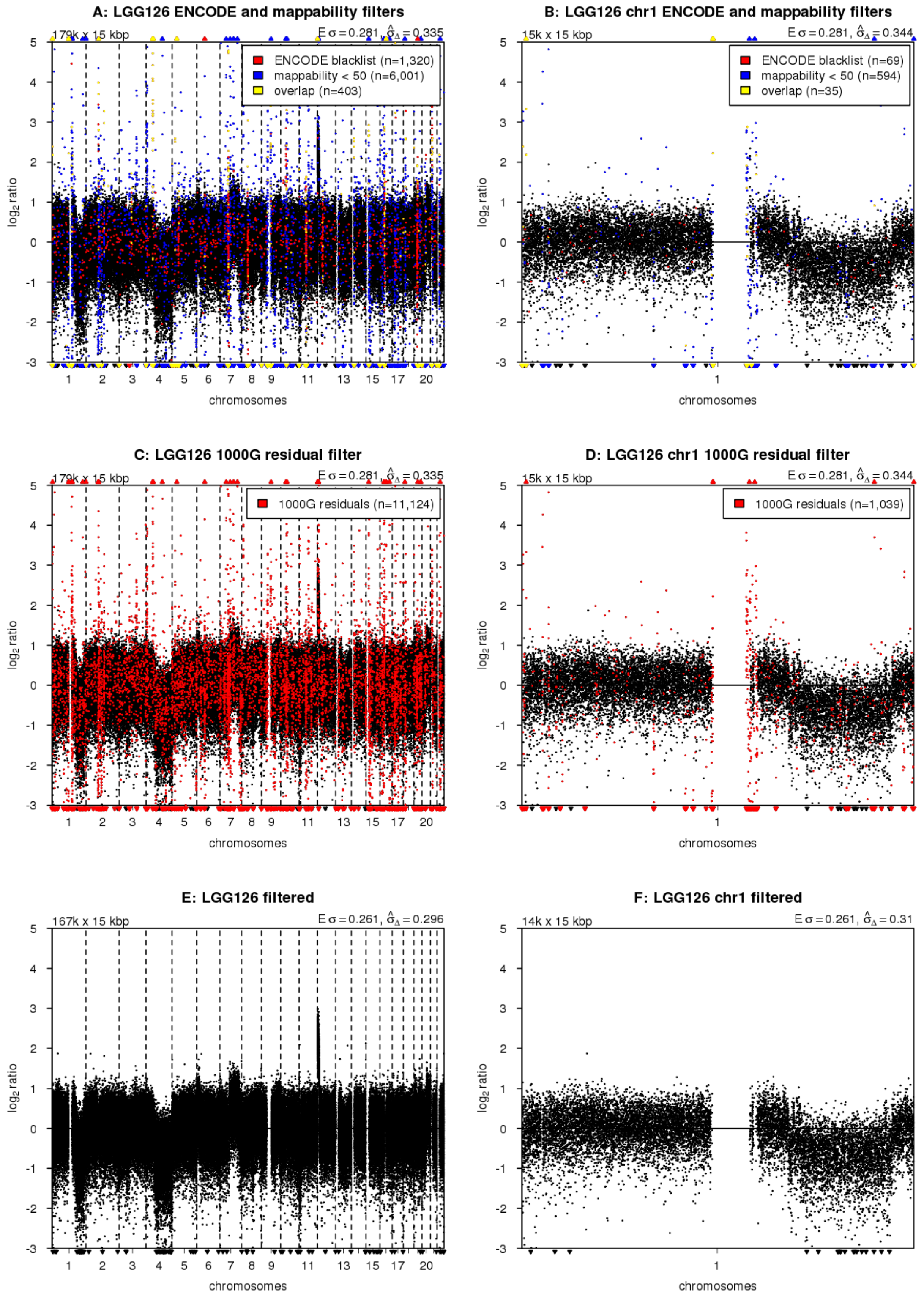

Figure S3: Blacklisting problematic regions. For (A) the whole genome and (B) chromosome 1, copy number profiles with bins overlapping with the ENCODE blacklist highlighted in red, bins with mappabilities below 50 highlighted in blue, and the overlap between the two in yellow. For (C) the whole genome, and (D) chromosome 1, copy number profiles with bins in the novel blacklist based on residuals of the 1000 Genomes samples highlighted in red. For (E) the whole genome and (F) chromosome 1, the final copy number profiles after filtering out bins with the ENCODE and 1000G blacklists. Bins are ordered along the x-axis by their genomic positions, and y-axis shows median-normalized $\log_2$-transformed data. Small triangles at the top and bottom edges represent data points that fall outside the plot area. Top-left corners show the number and size of bins. Top-right corners show the expected and measured standard deviation. The expected standard deviation (E σ) is defined as $\sqrt{1/N}$, where N is the average number of reads per bin. The measured standard deviation ($\hat{\sigma}_\Delta$) is calculated from the data with a 0.1%-trimmed first-order estimate (prior to $\log_2$-transforming the data for plotting).

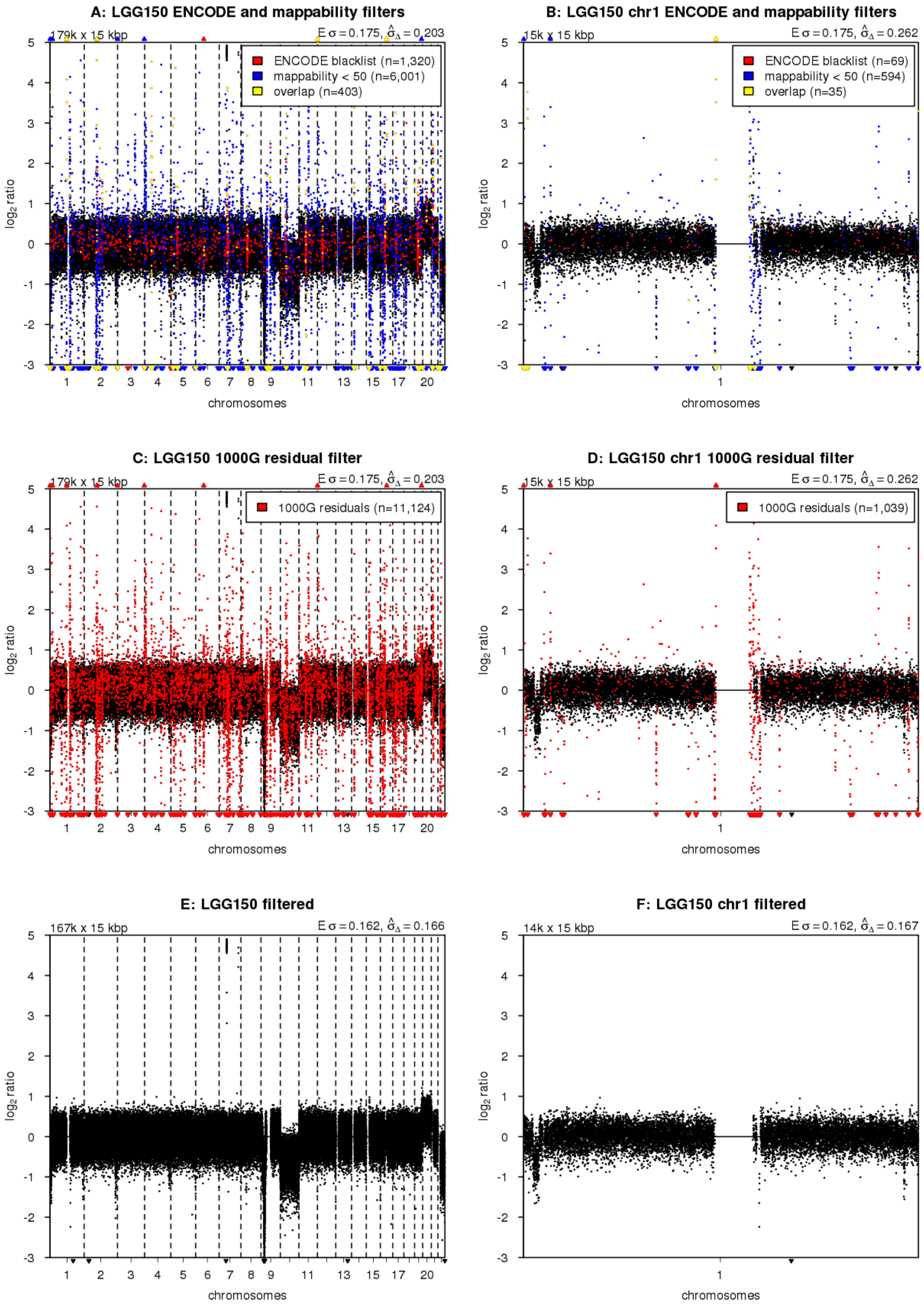

Figure S3: Blacklisting problematic regions (cont.)

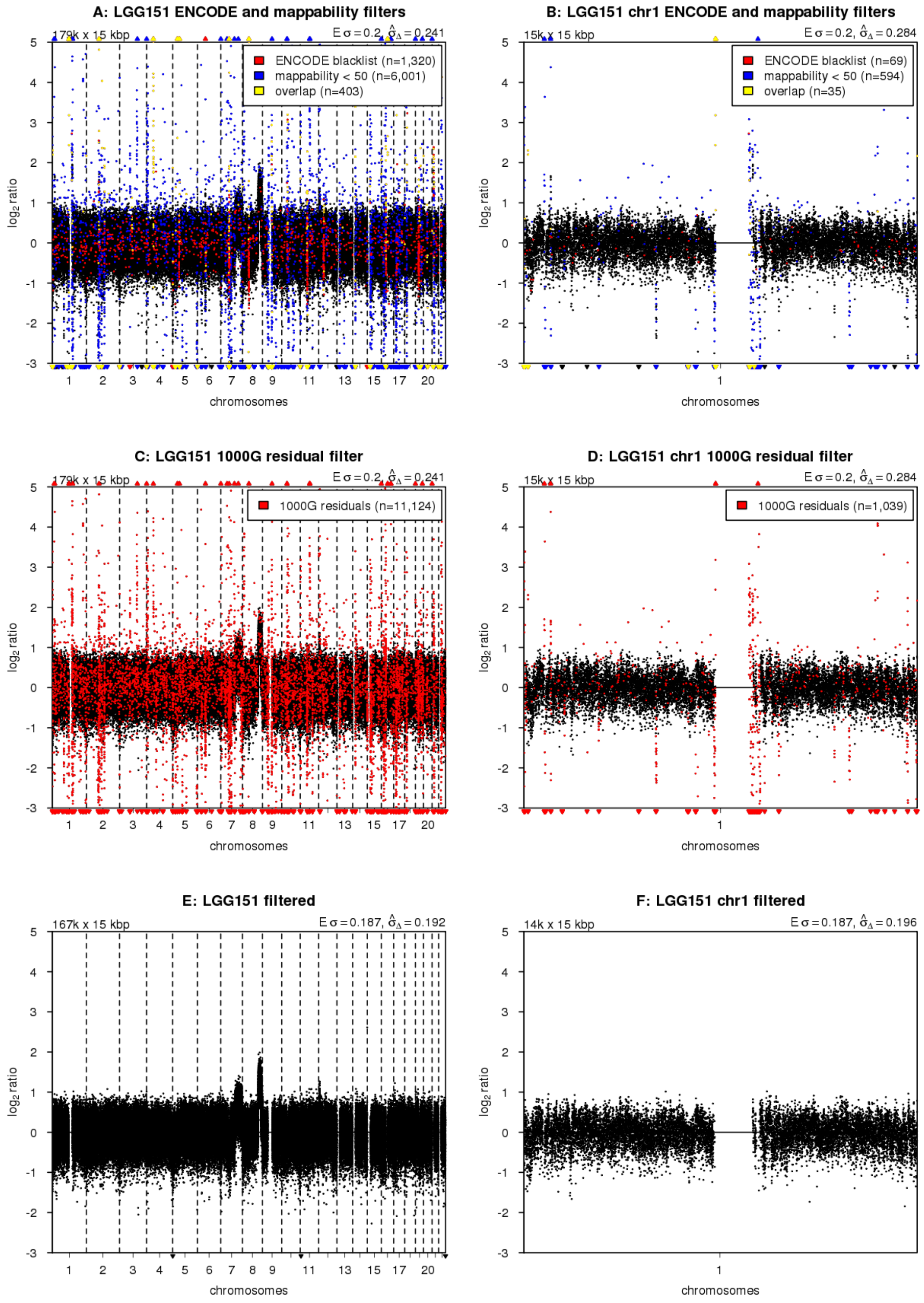

Figure S3: Blacklisting problematic regions (cont.)

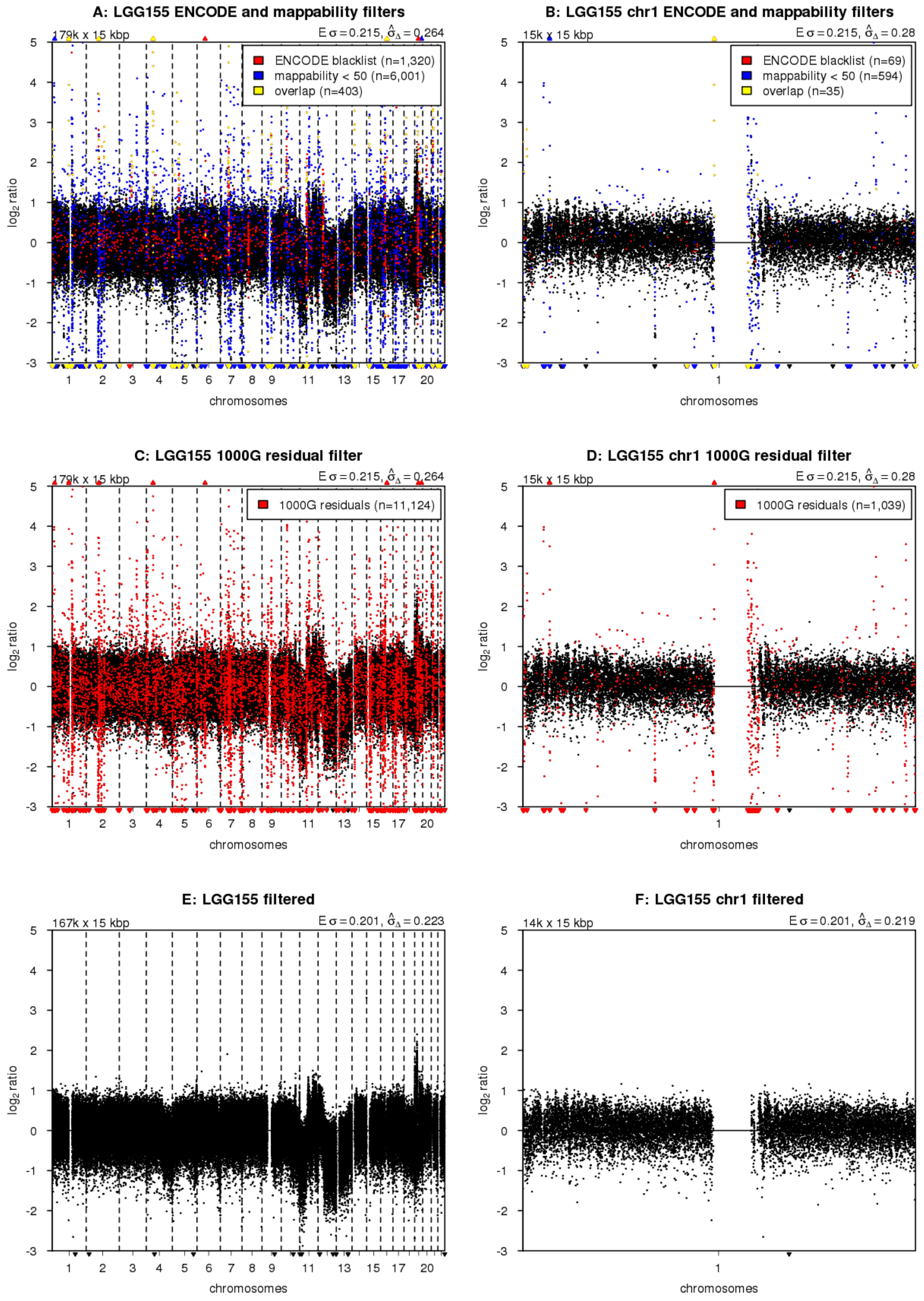

Figure S3: Blacklisting problematic regions (cont.)

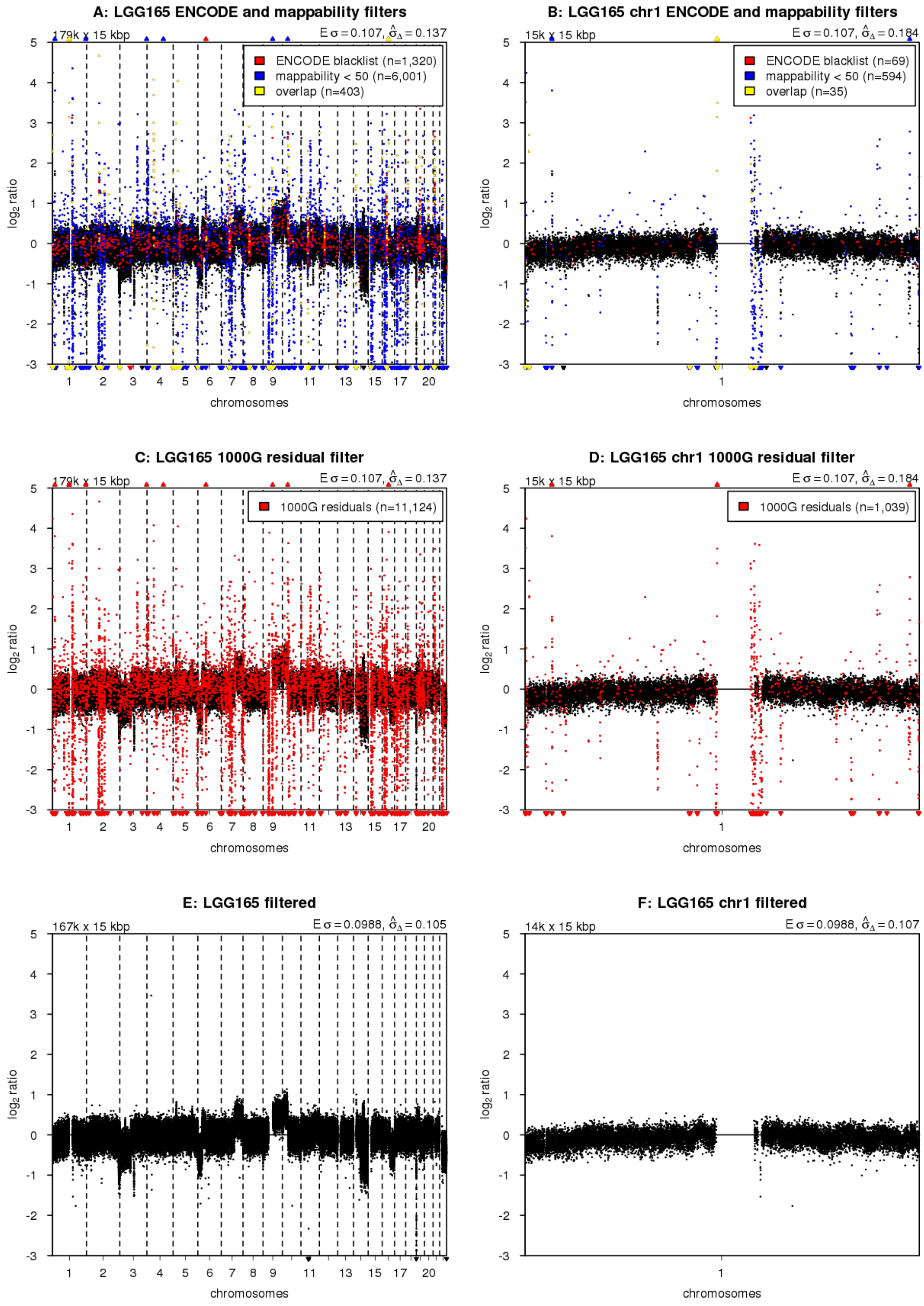

Figure S3: Blacklisting problematic regions (cont.)

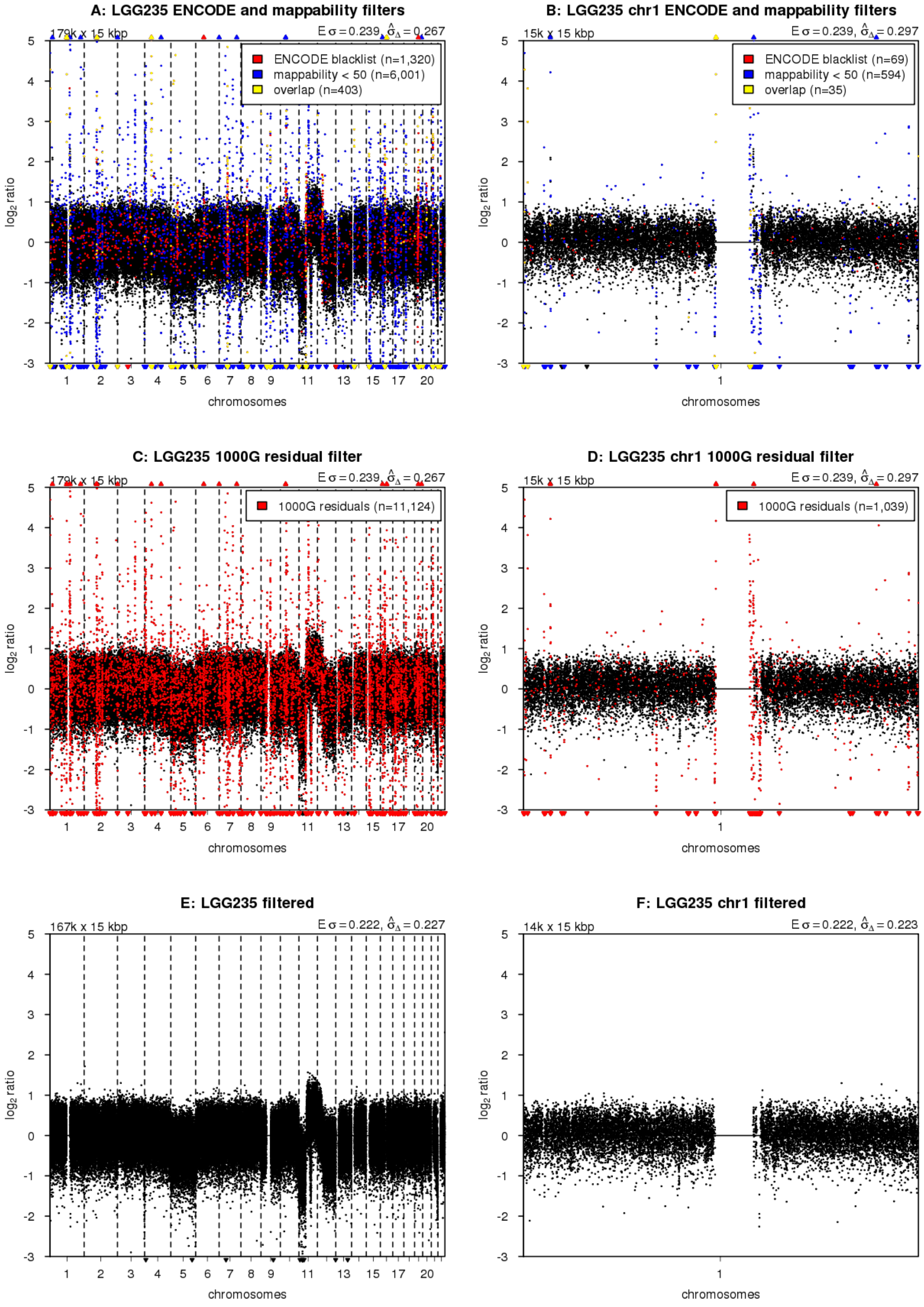

Figure S3: Blacklisting problematic regions (cont.)

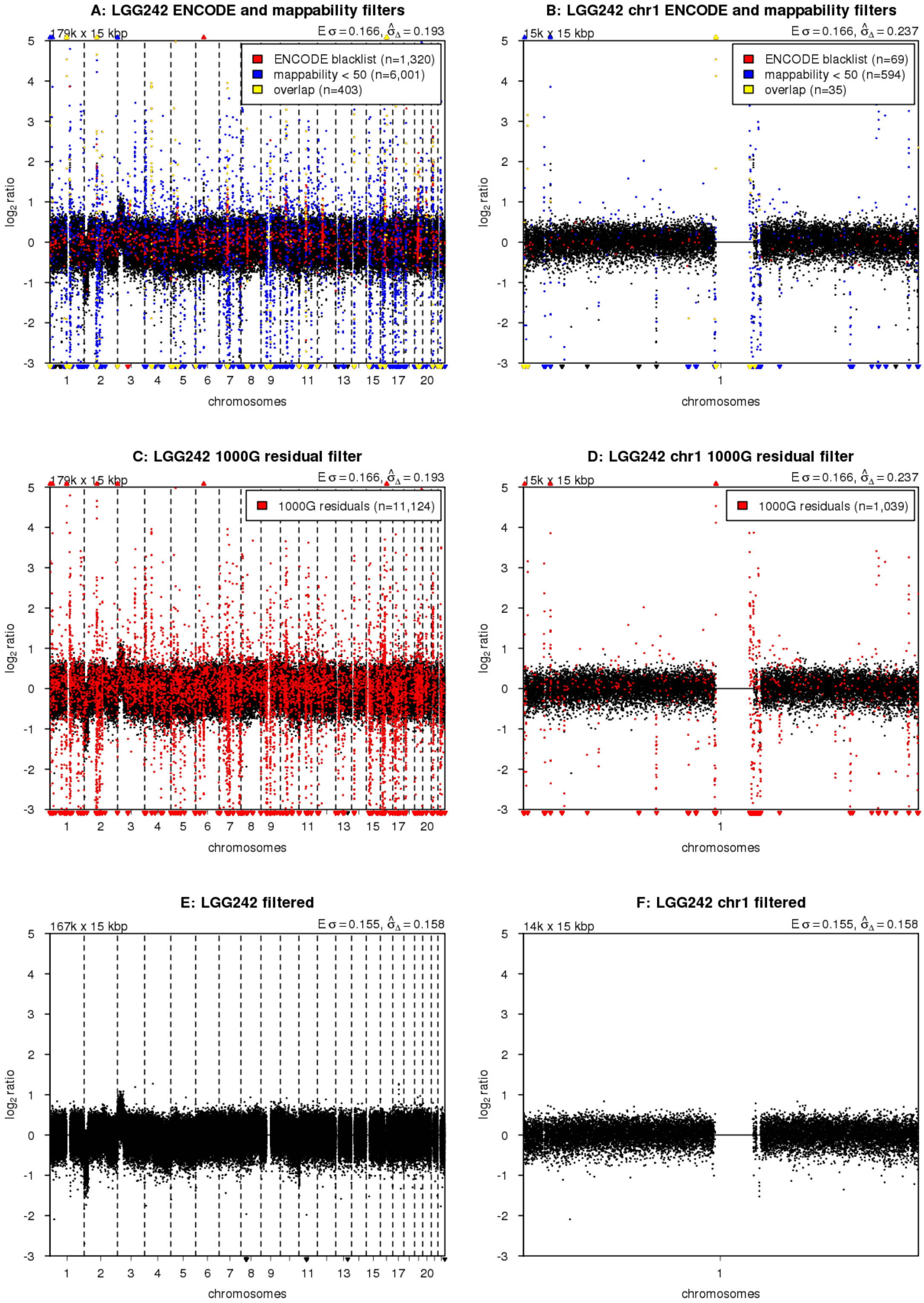

Figure S3: Blacklisting problematic regions (cont.)

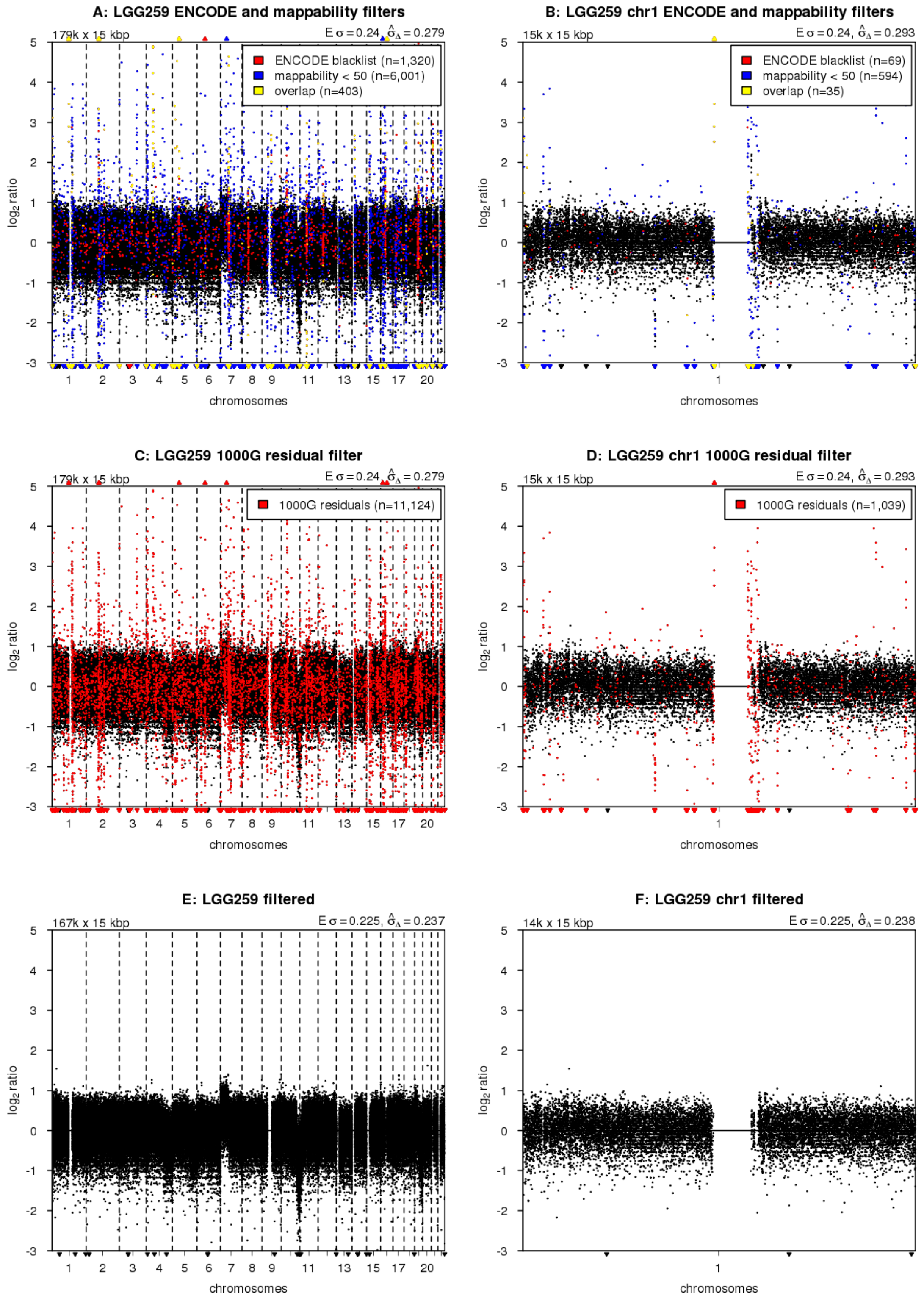

Figure S3: Blacklisting problematic regions (cont.)

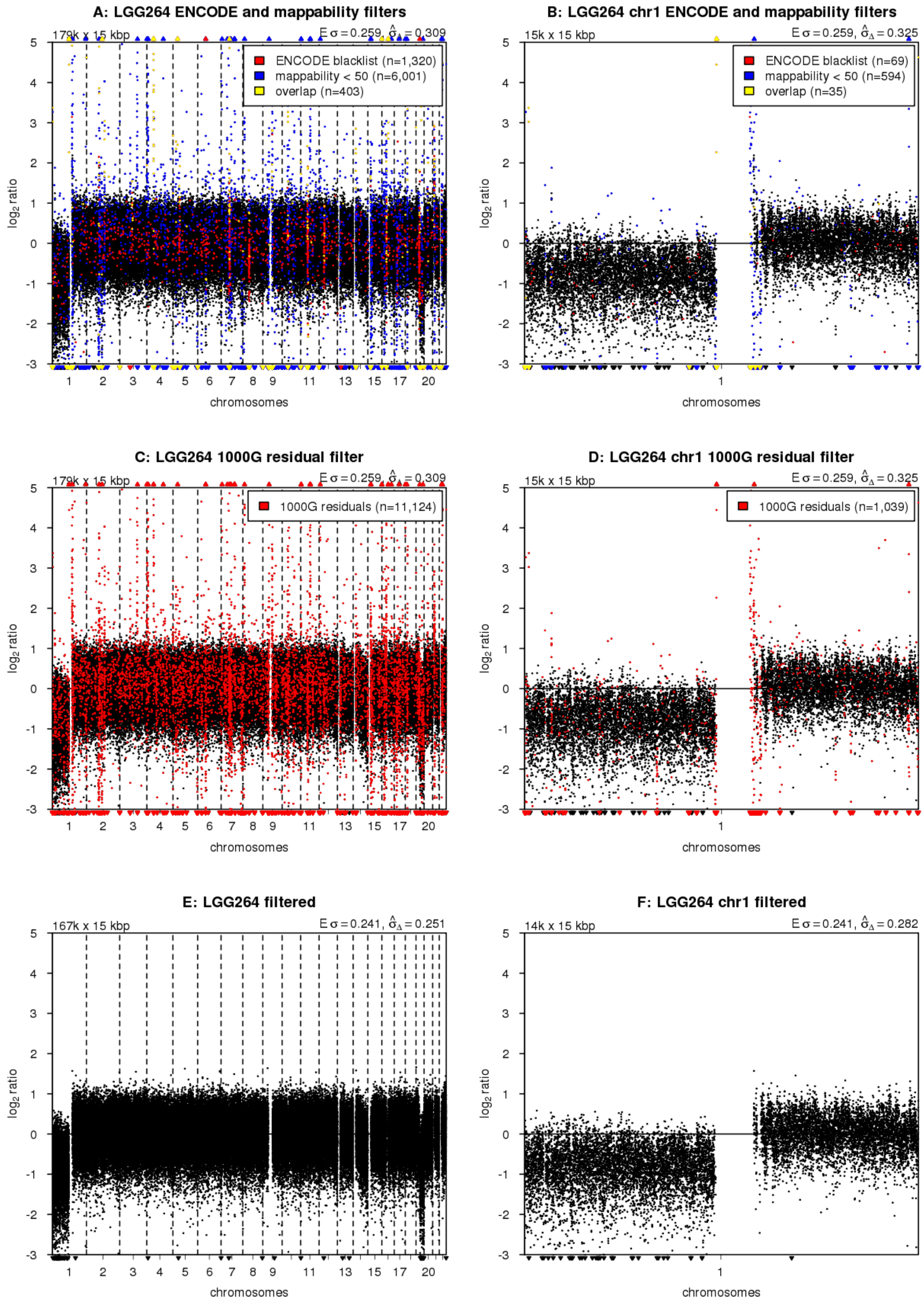

Figure S3: Blacklisting problematic regions (cont.)

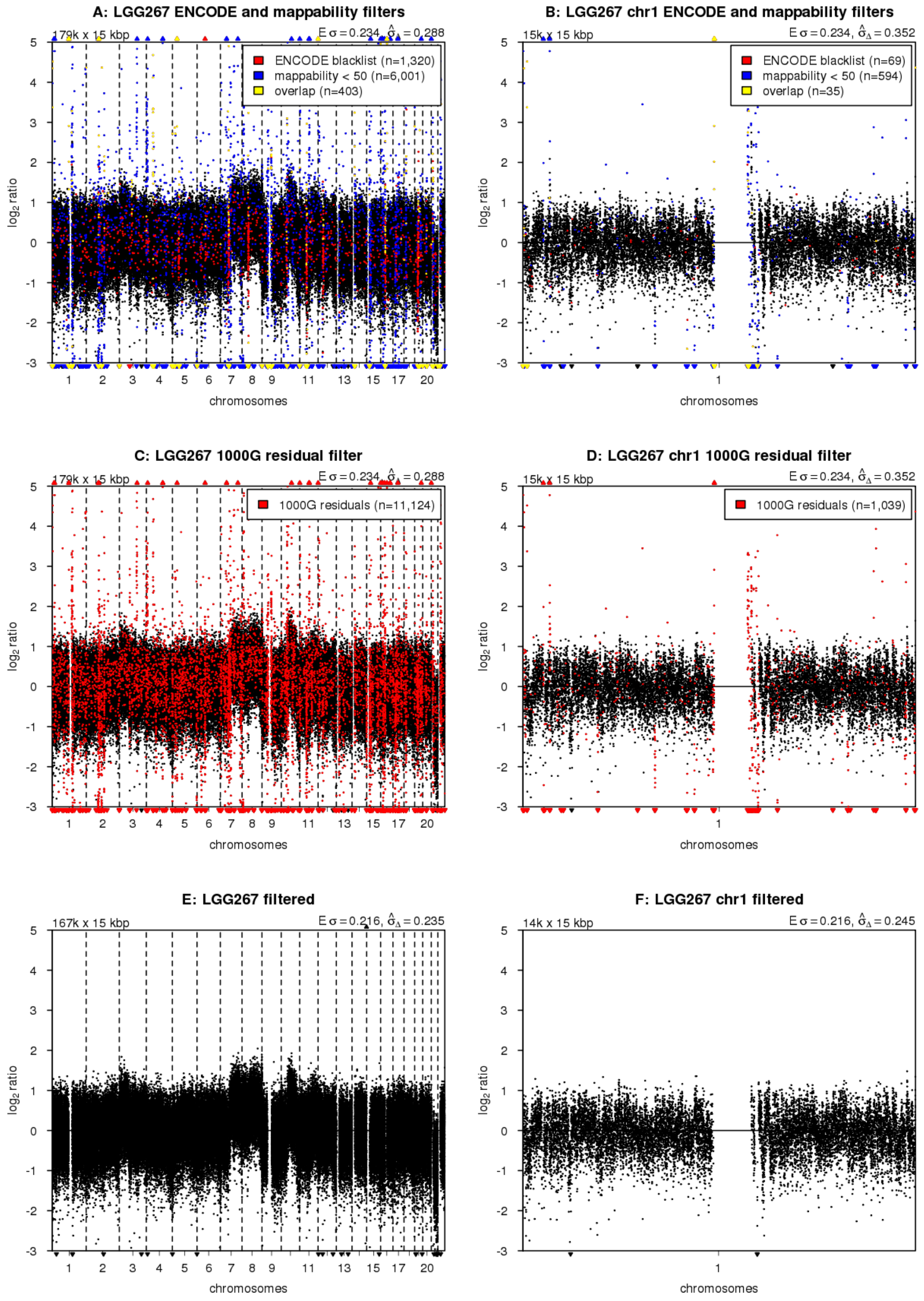

Figure S3: Blacklisting problematic regions (cont.)

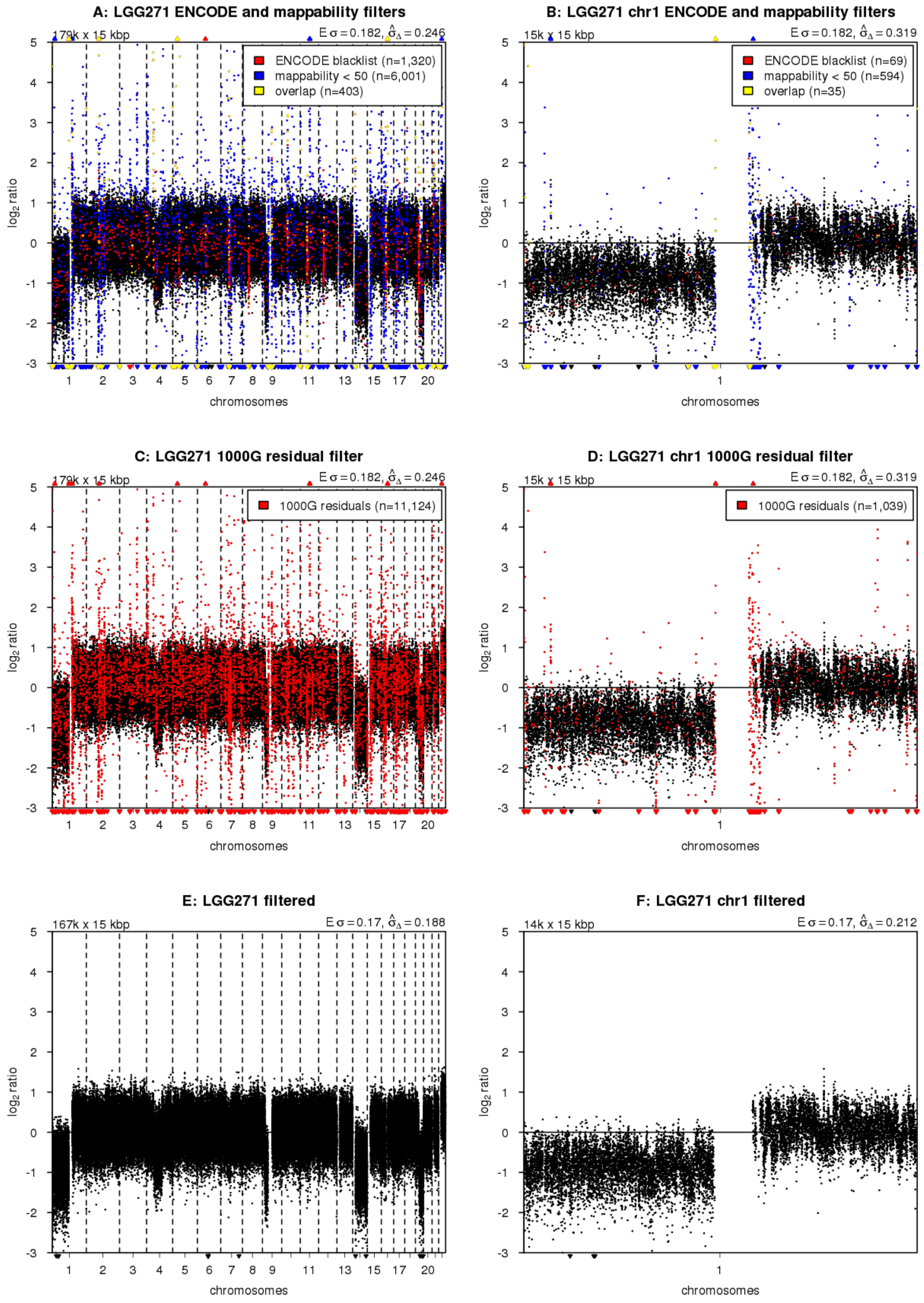

Figure S3: Blacklisting problematic regions (cont.)

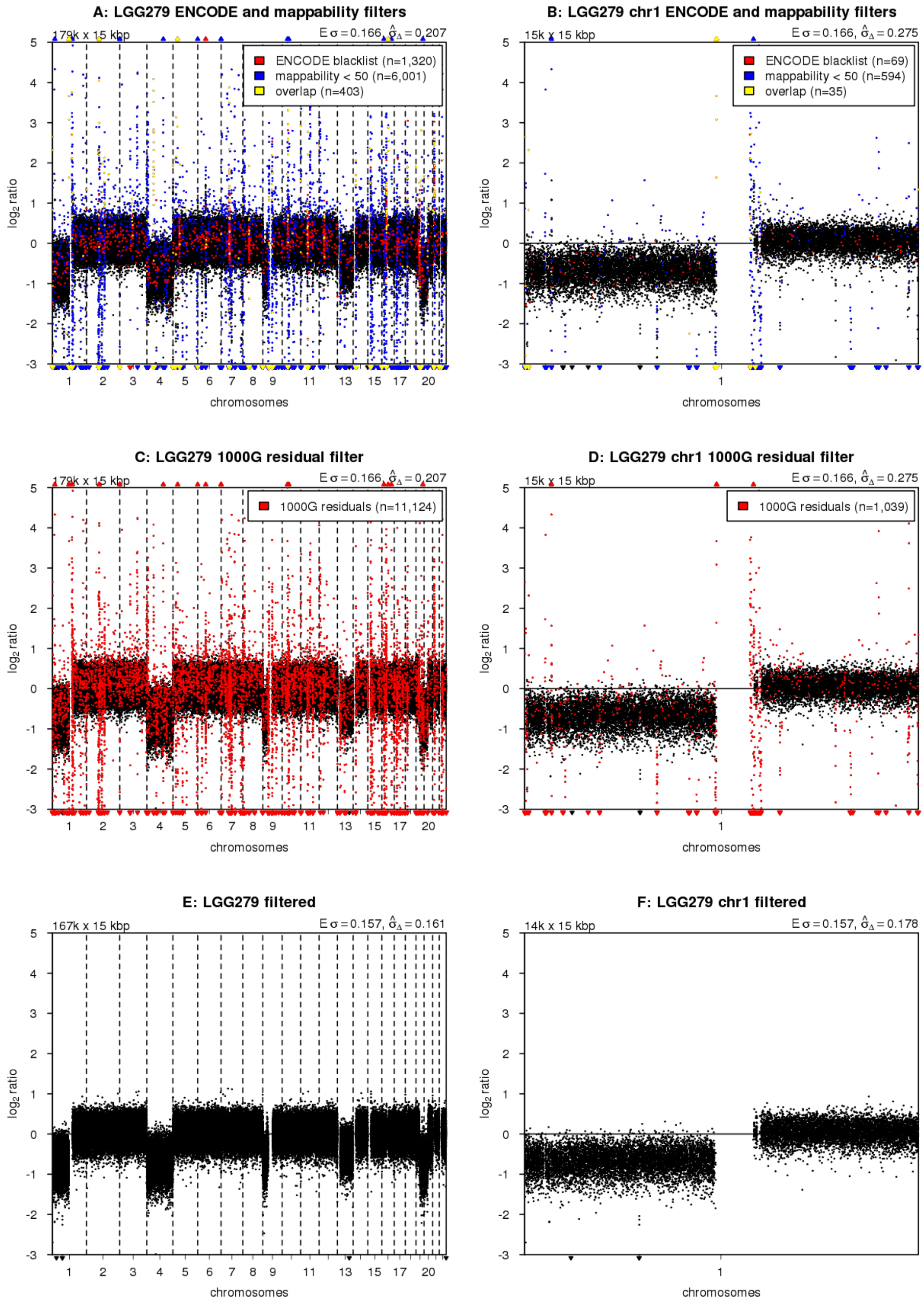

Figure S3: Blacklisting problematic regions (cont.)

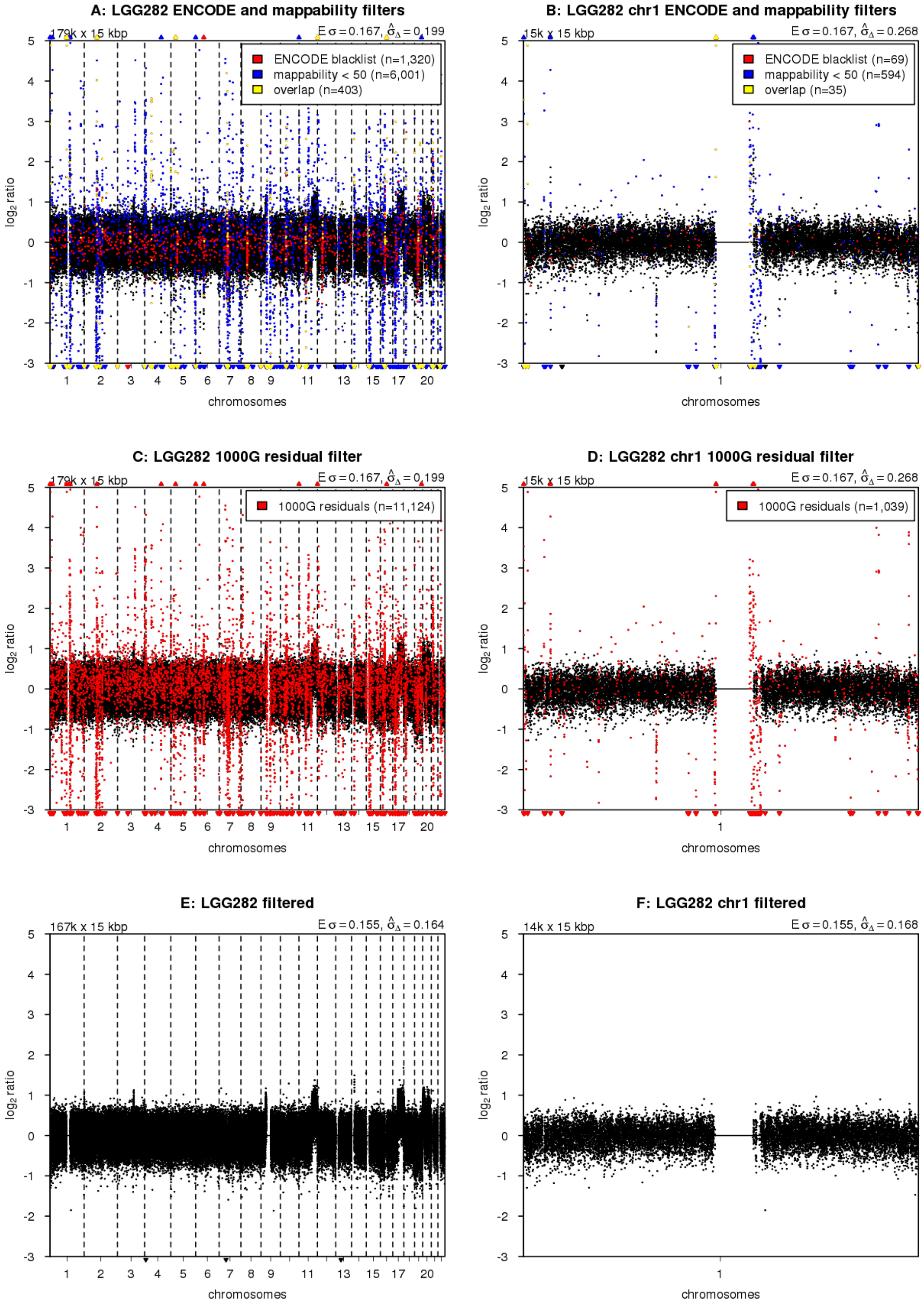

Figure S3: Blacklisting problematic regions (cont.)

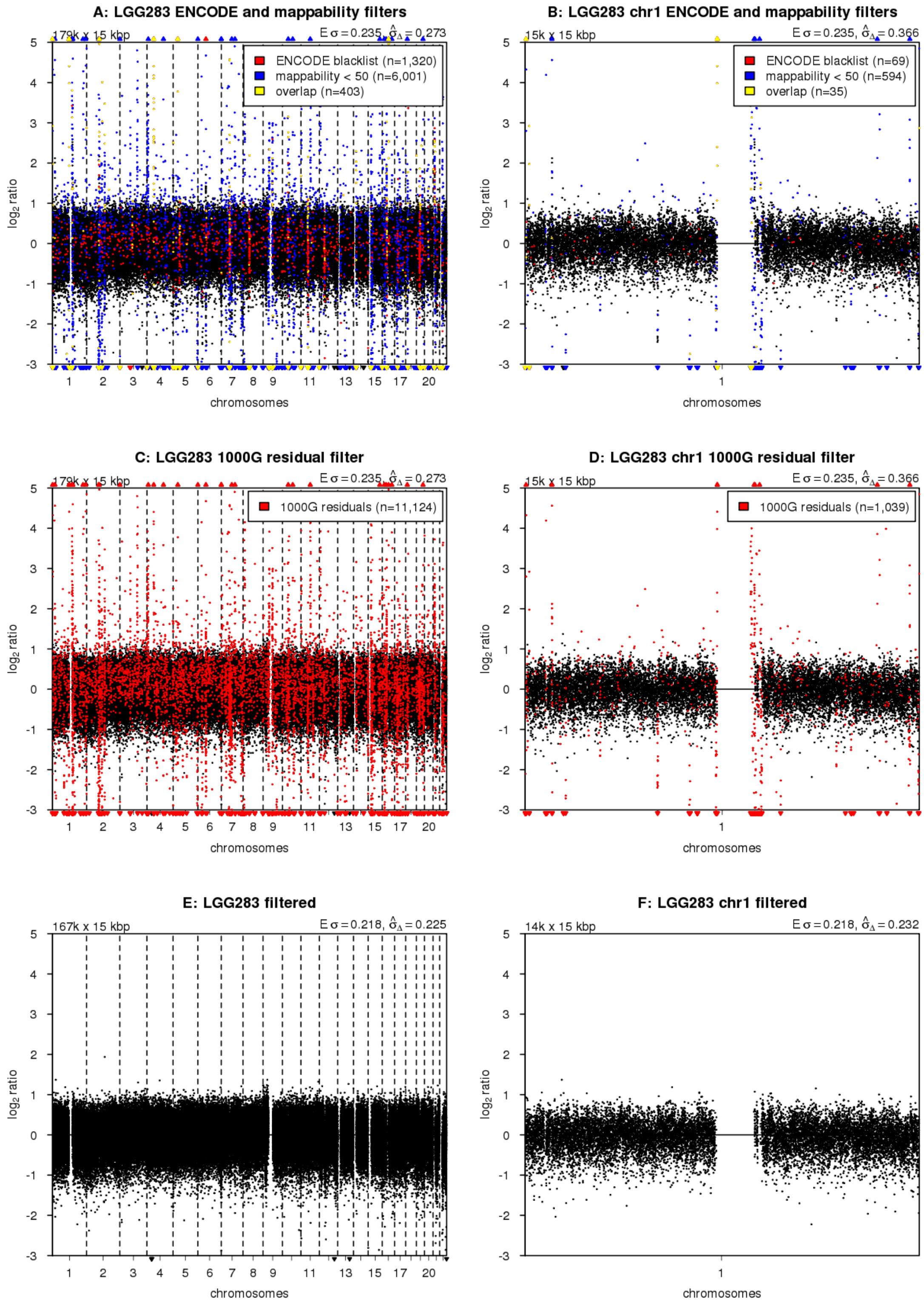

Figure S3: Blacklisting problematic regions (cont.)

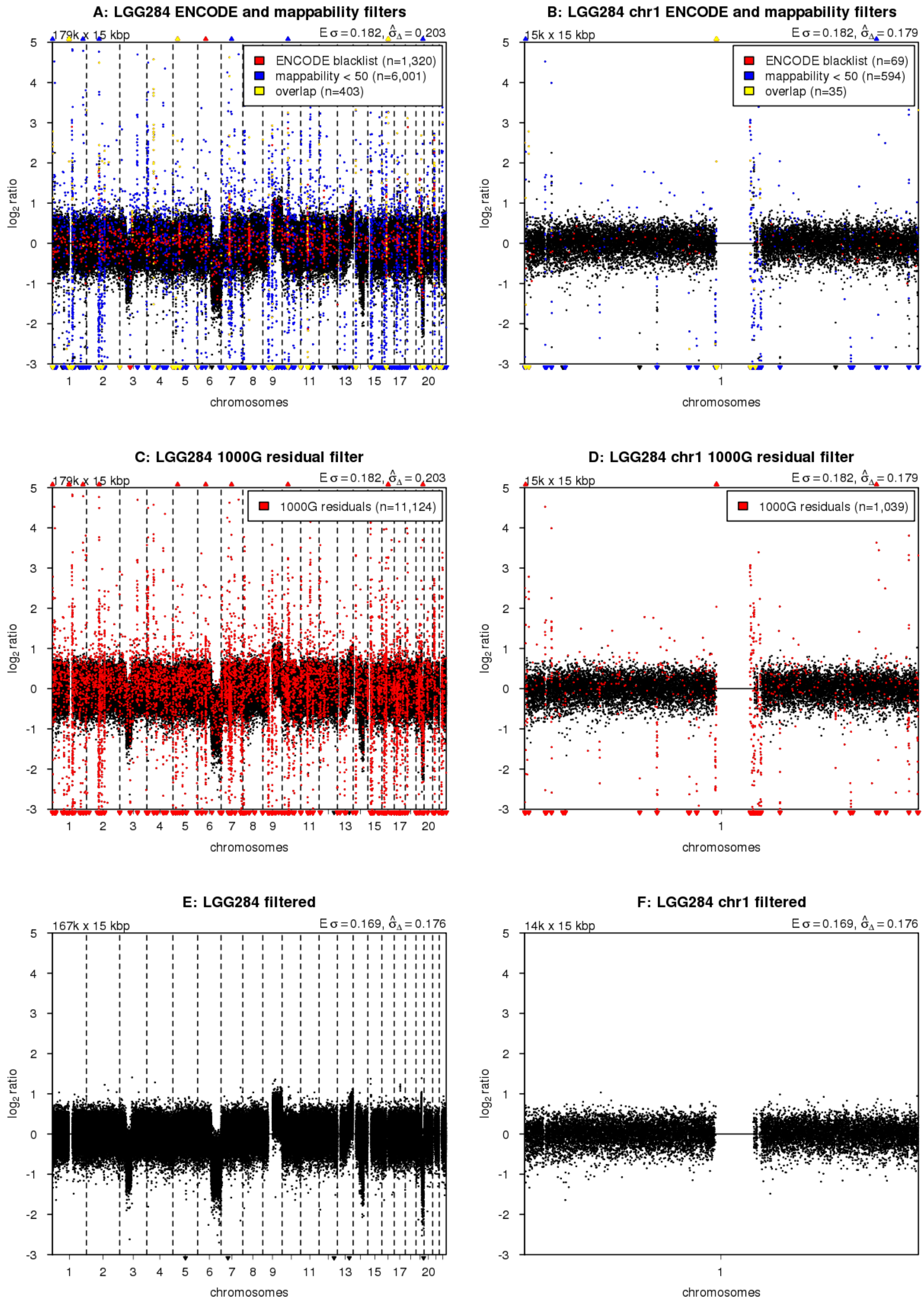

Figure S3: Blacklisting problematic regions (cont.)

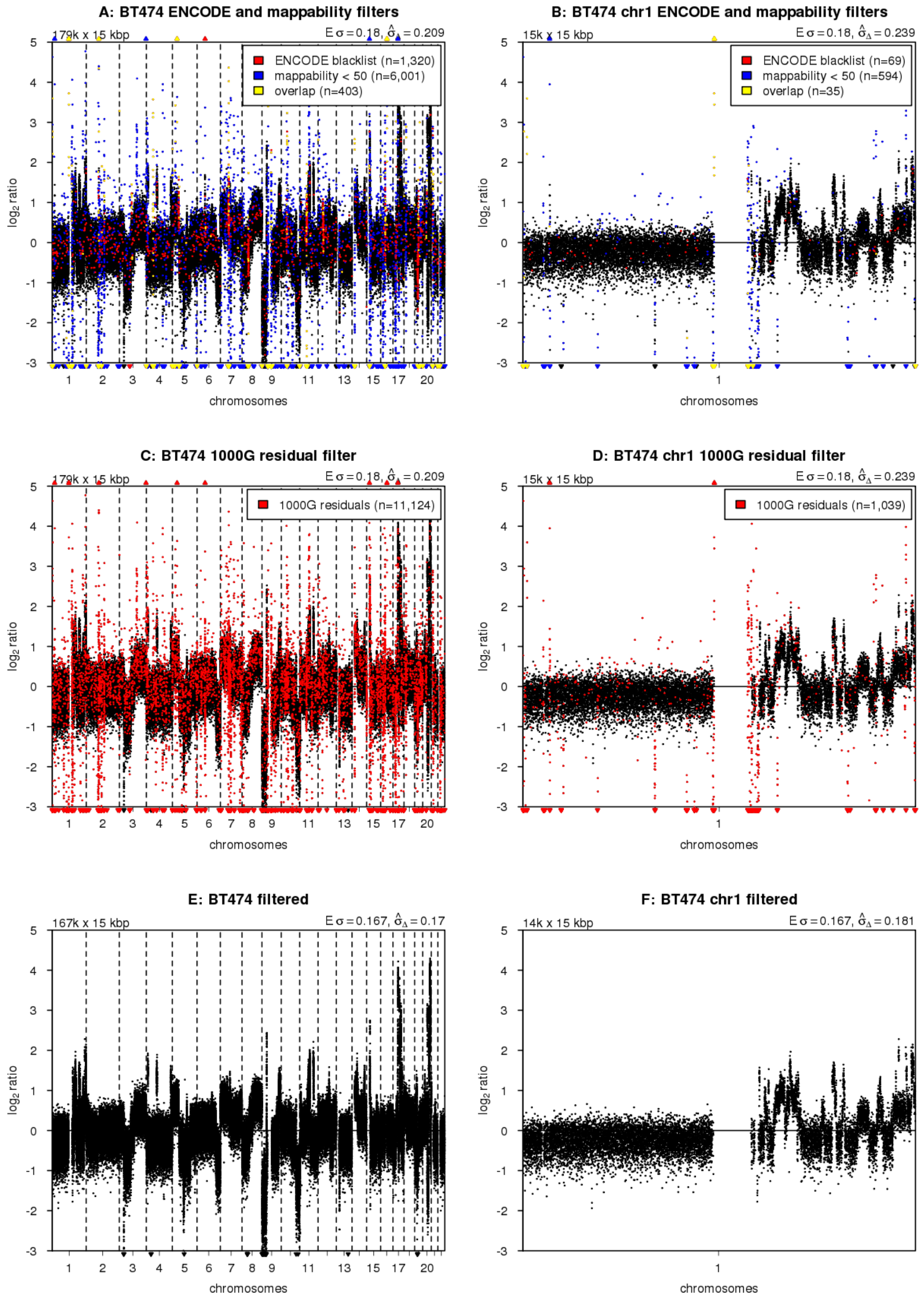

Figure S3: Blacklisting problematic regions (cont.)

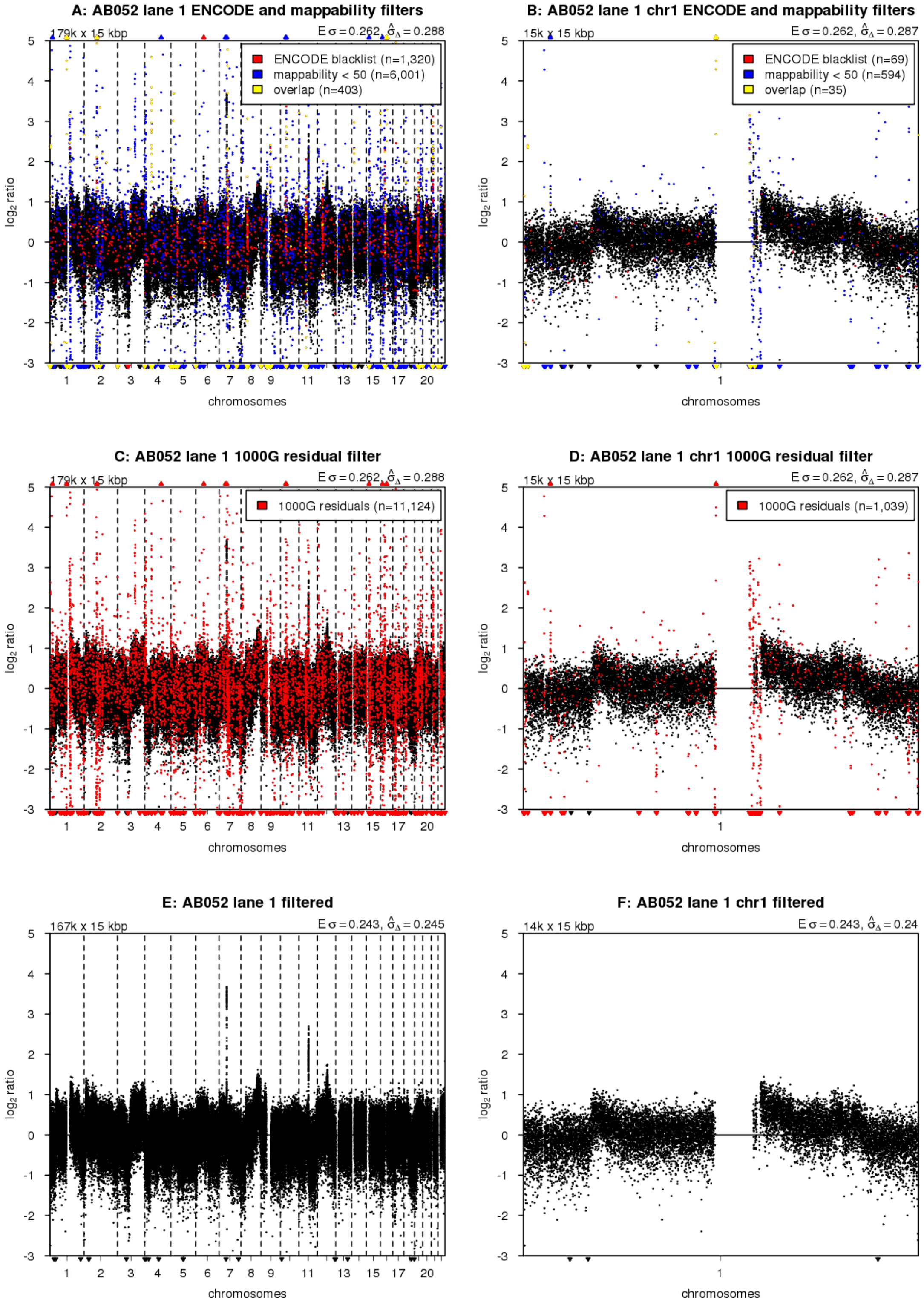

Figure S3: Blacklisting problematic regions (cont.)

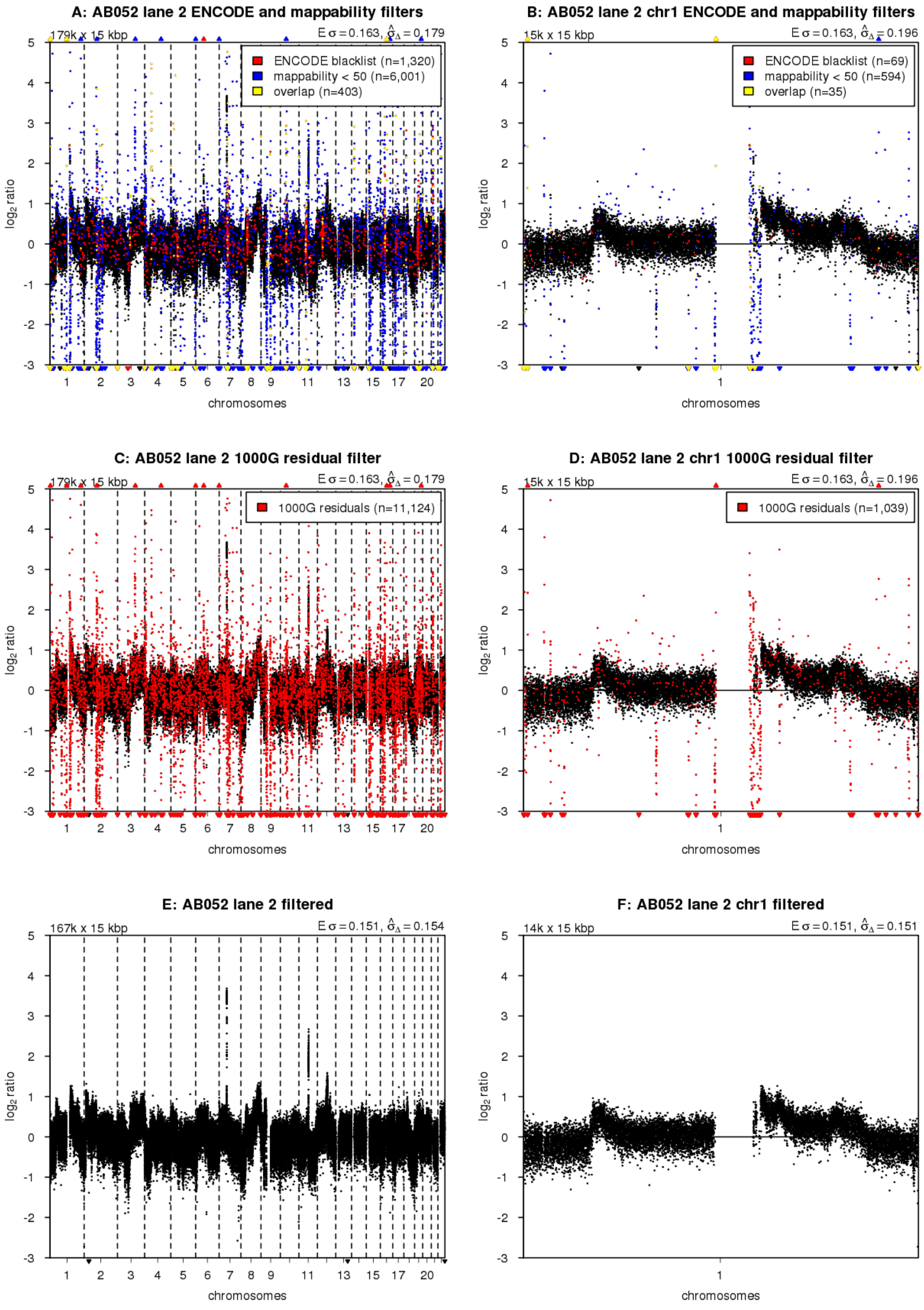

Figure S3: Blacklisting problematic regions (cont.)

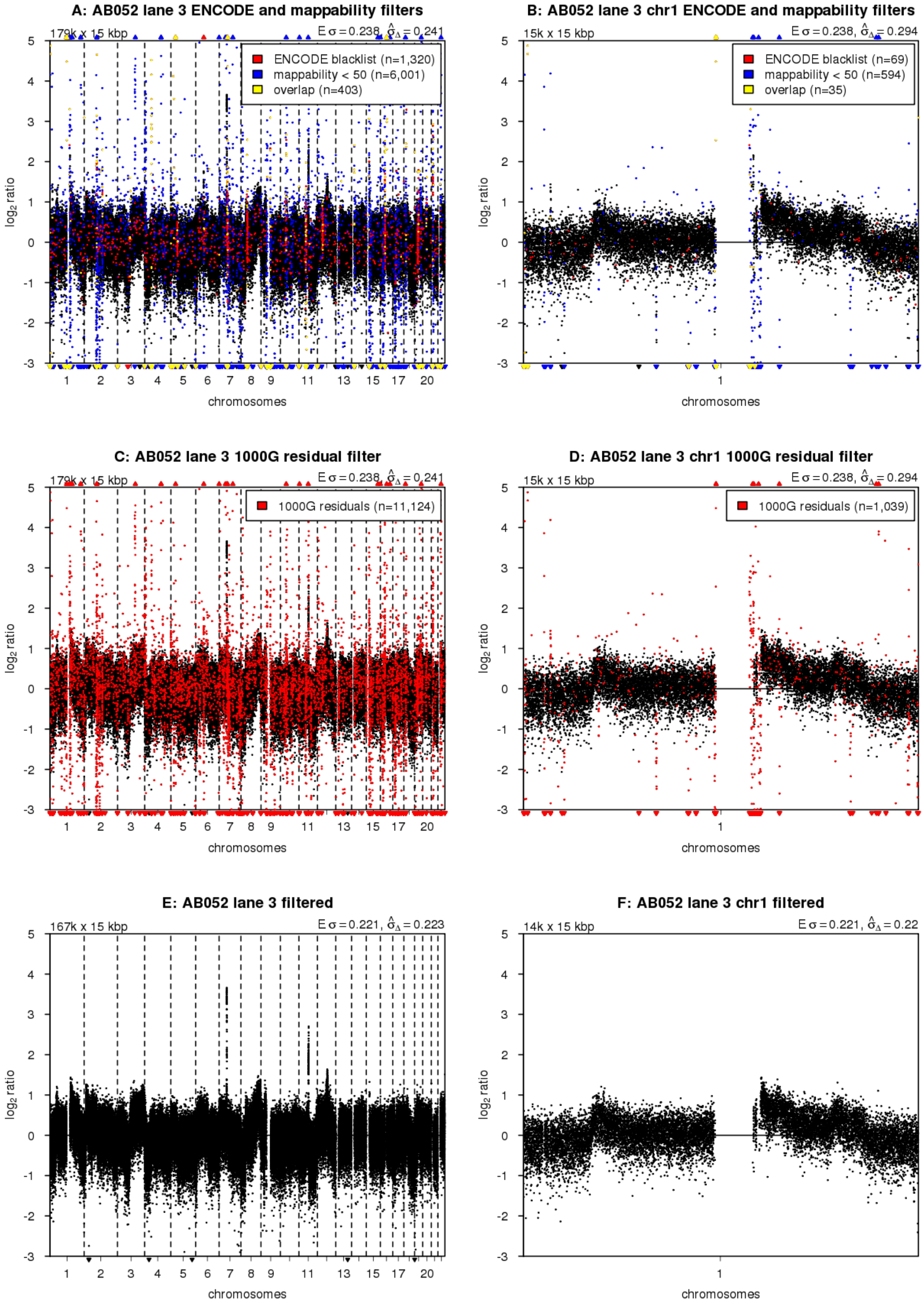

Figure S3: Blacklisting problematic regions (cont.)

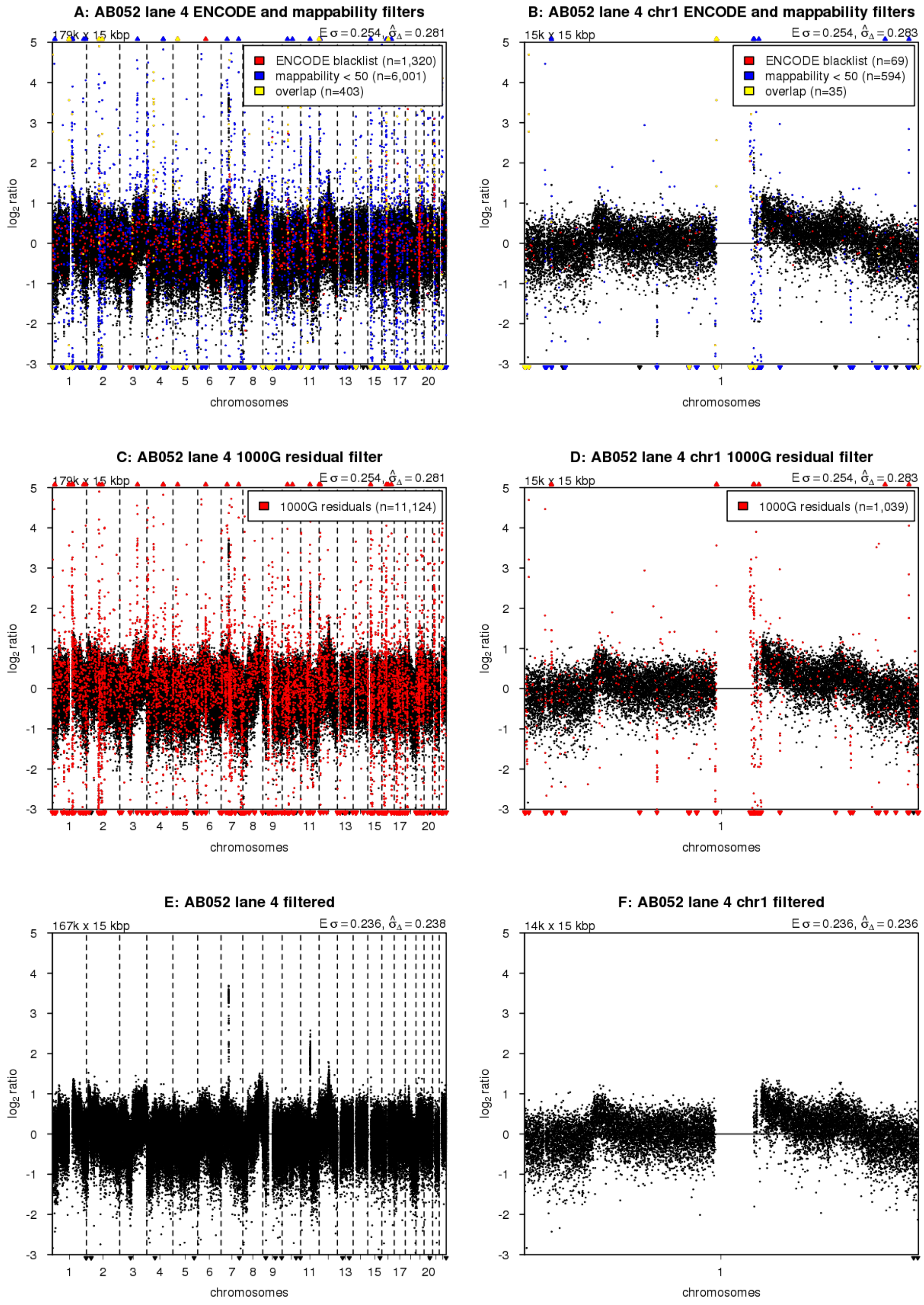

Figure S3: Blacklisting problematic regions (cont.)

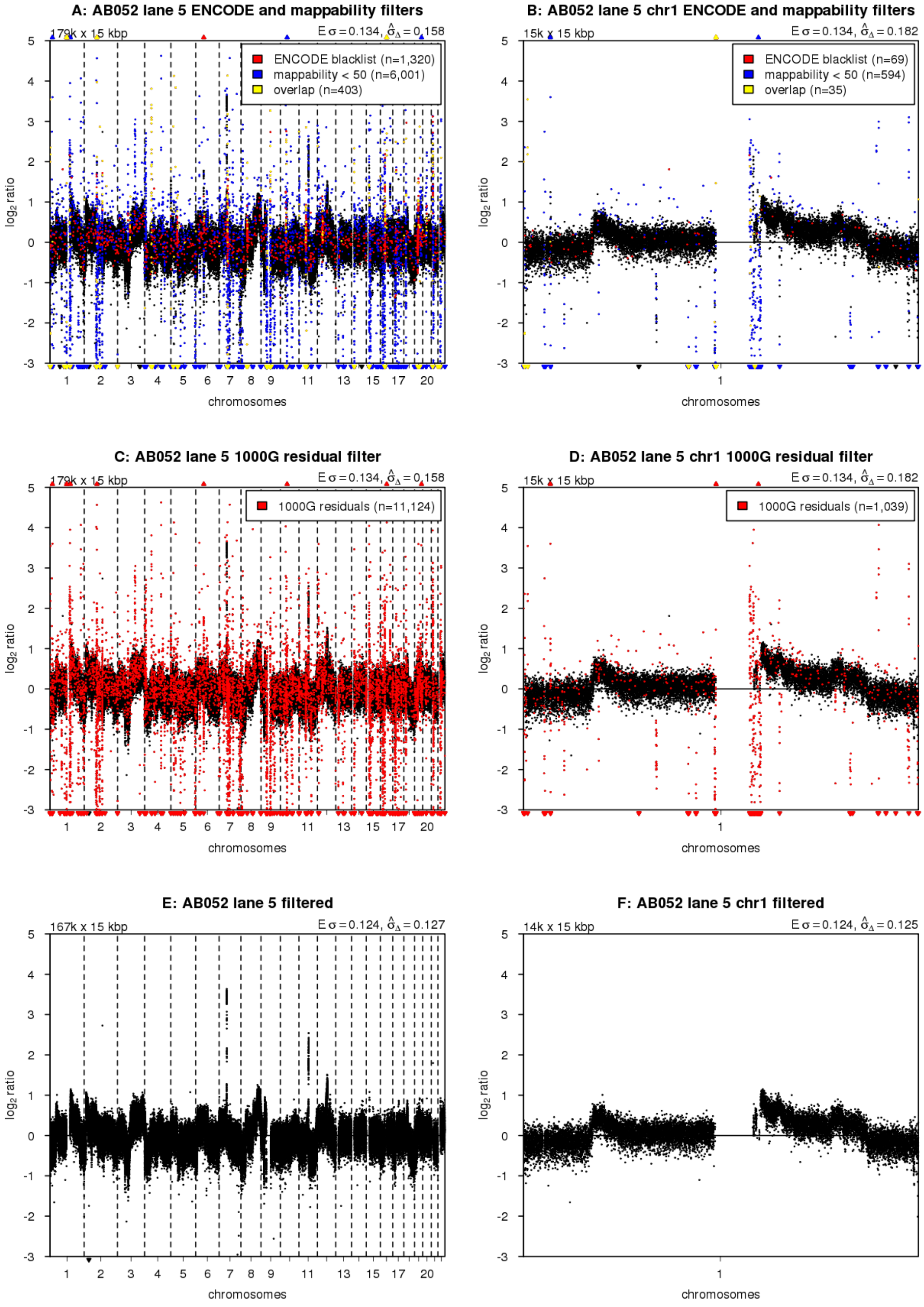

Figure S3: Blacklisting problematic regions (cont.)

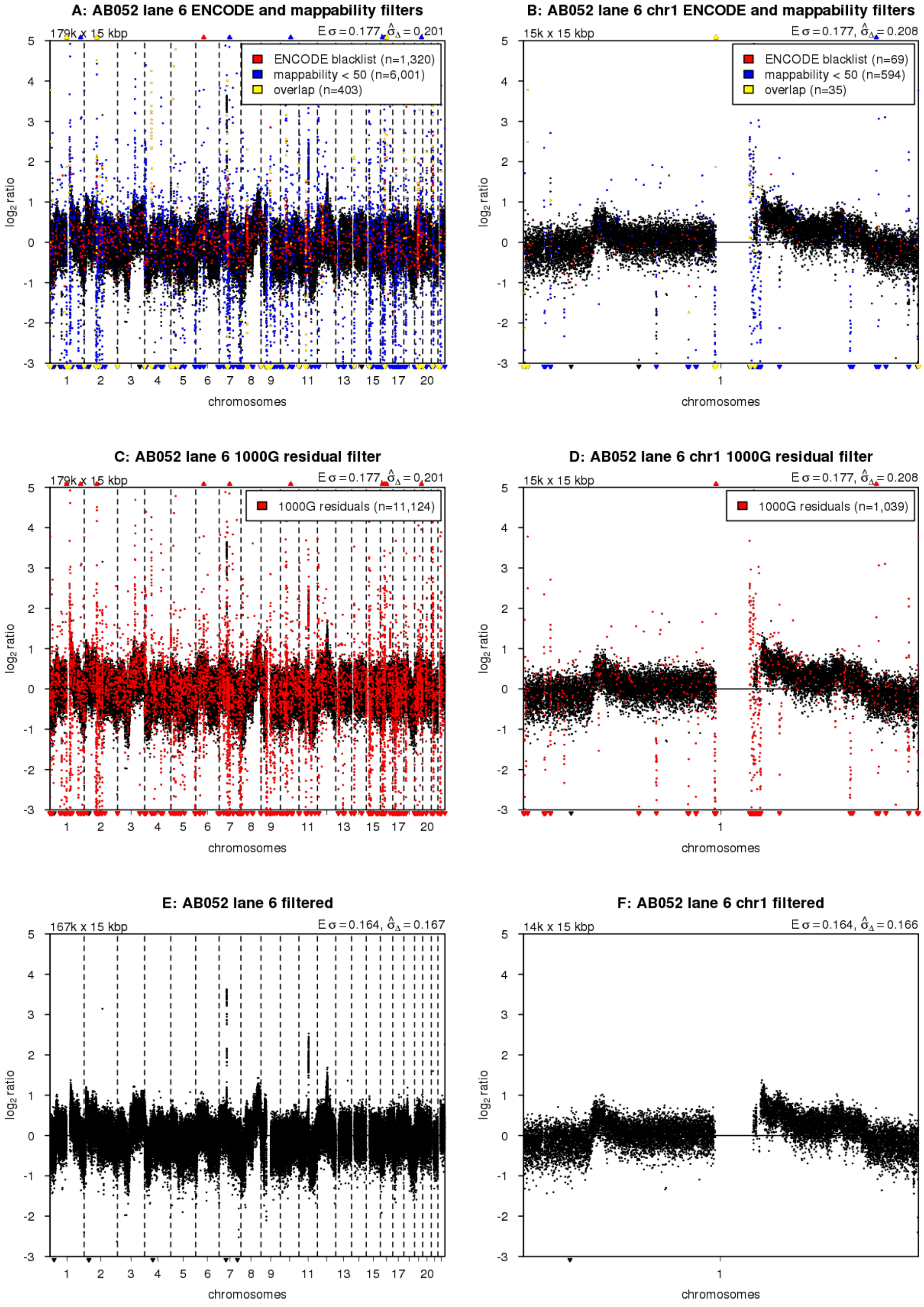

Figure S3: Blacklisting problematic regions (cont.)

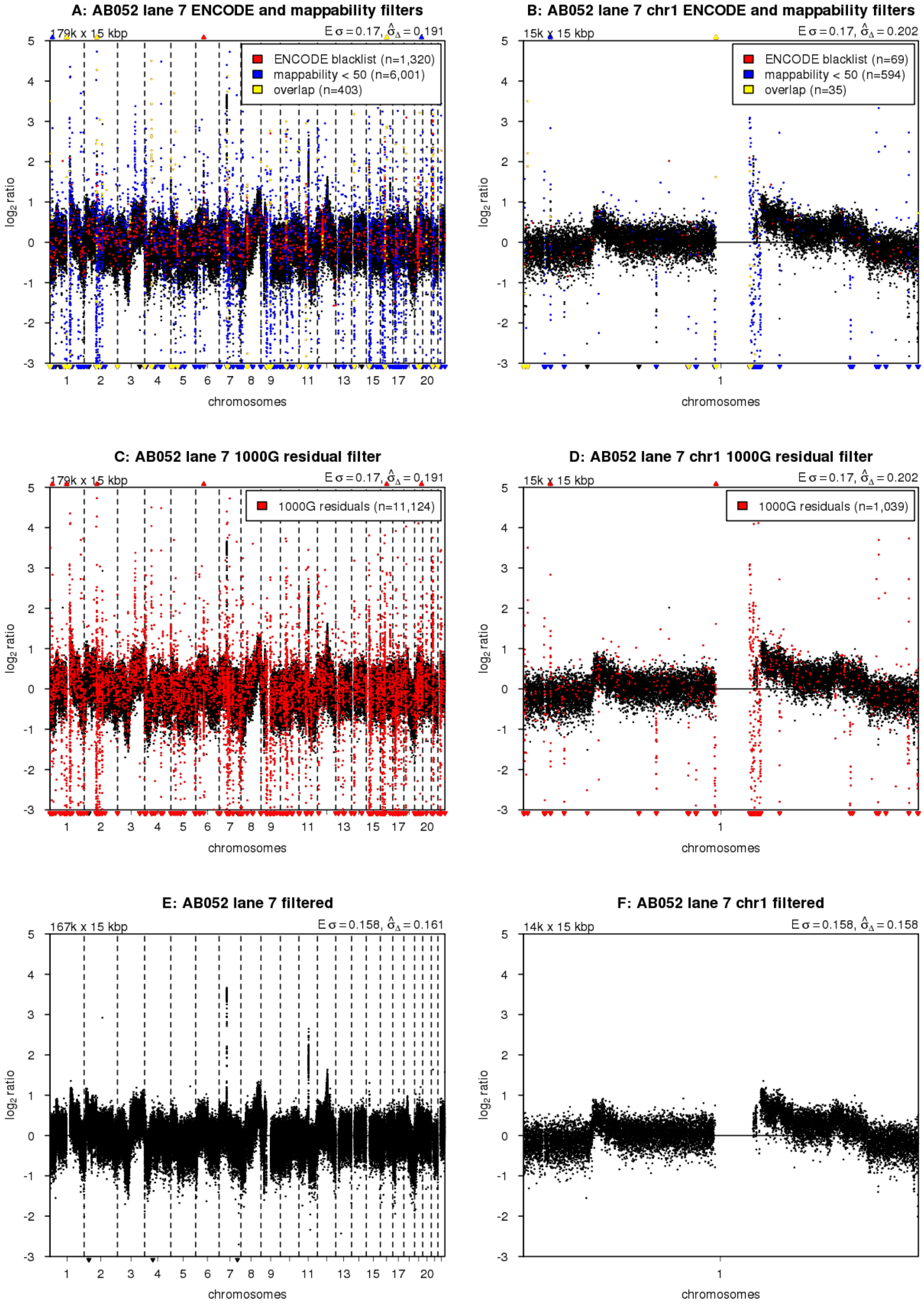

Figure S3: Blacklisting problematic regions (cont.)

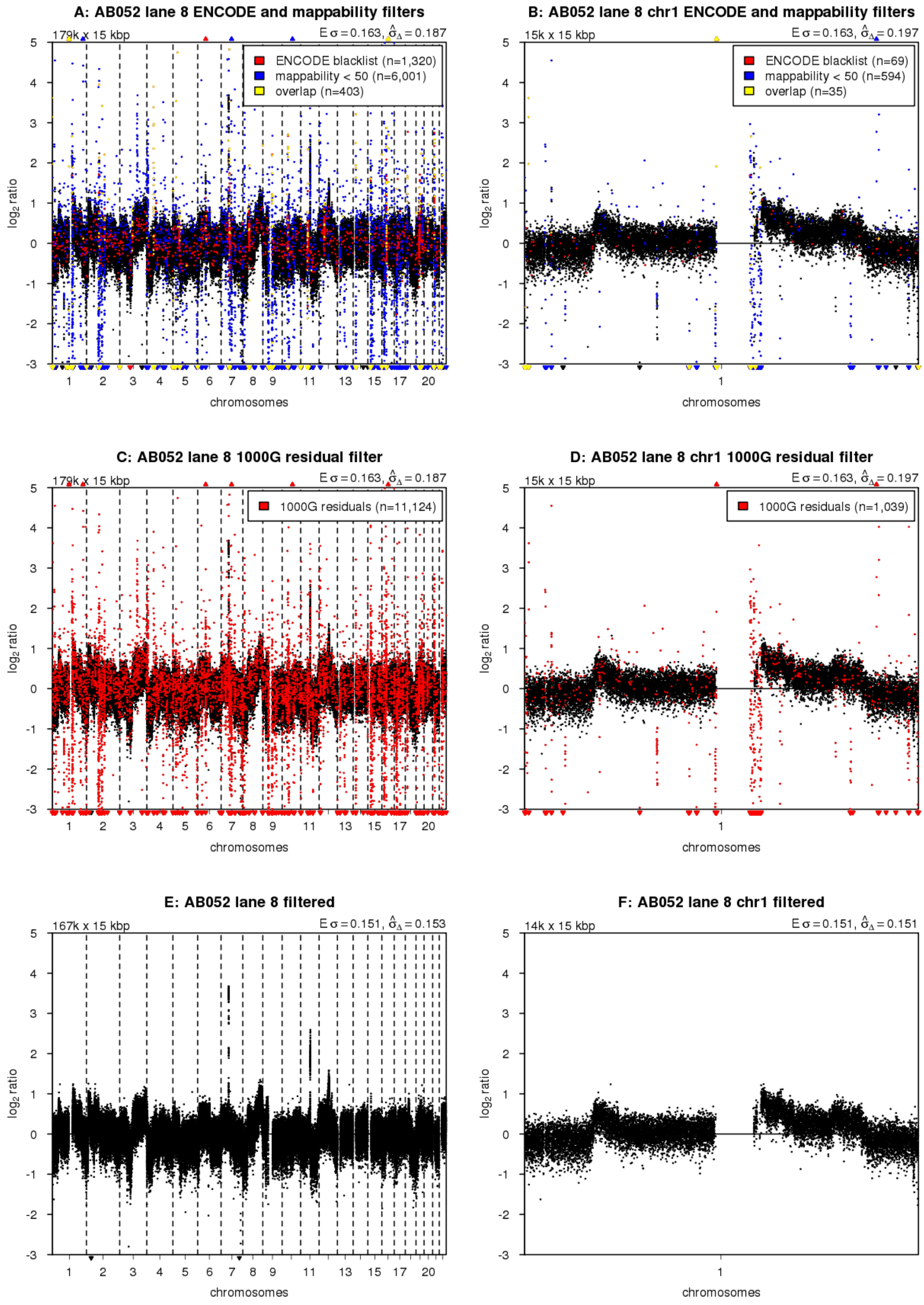

Figure S3: Blacklisting problematic regions (cont.)

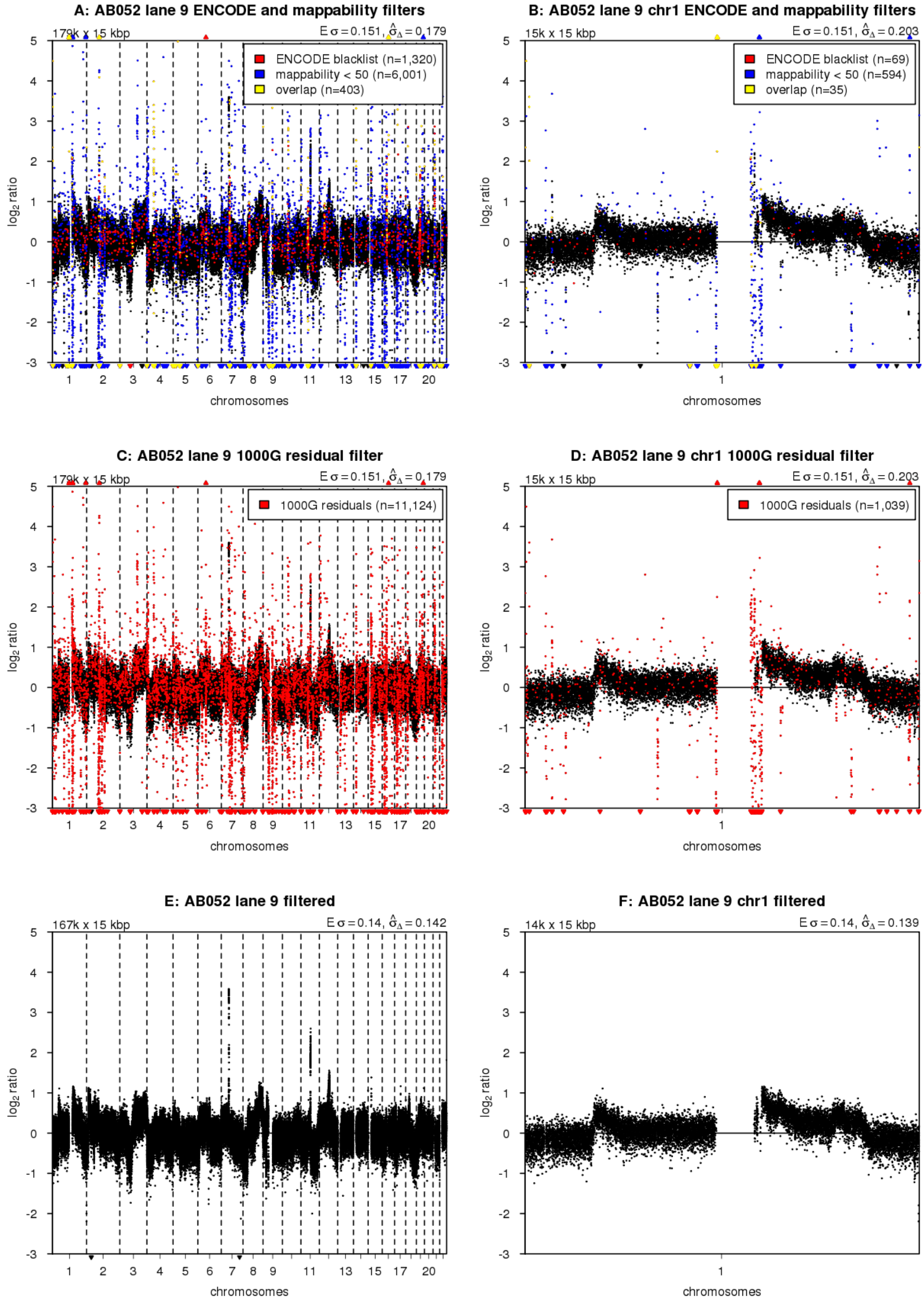

Figure S3: Blacklisting problematic regions (cont.)

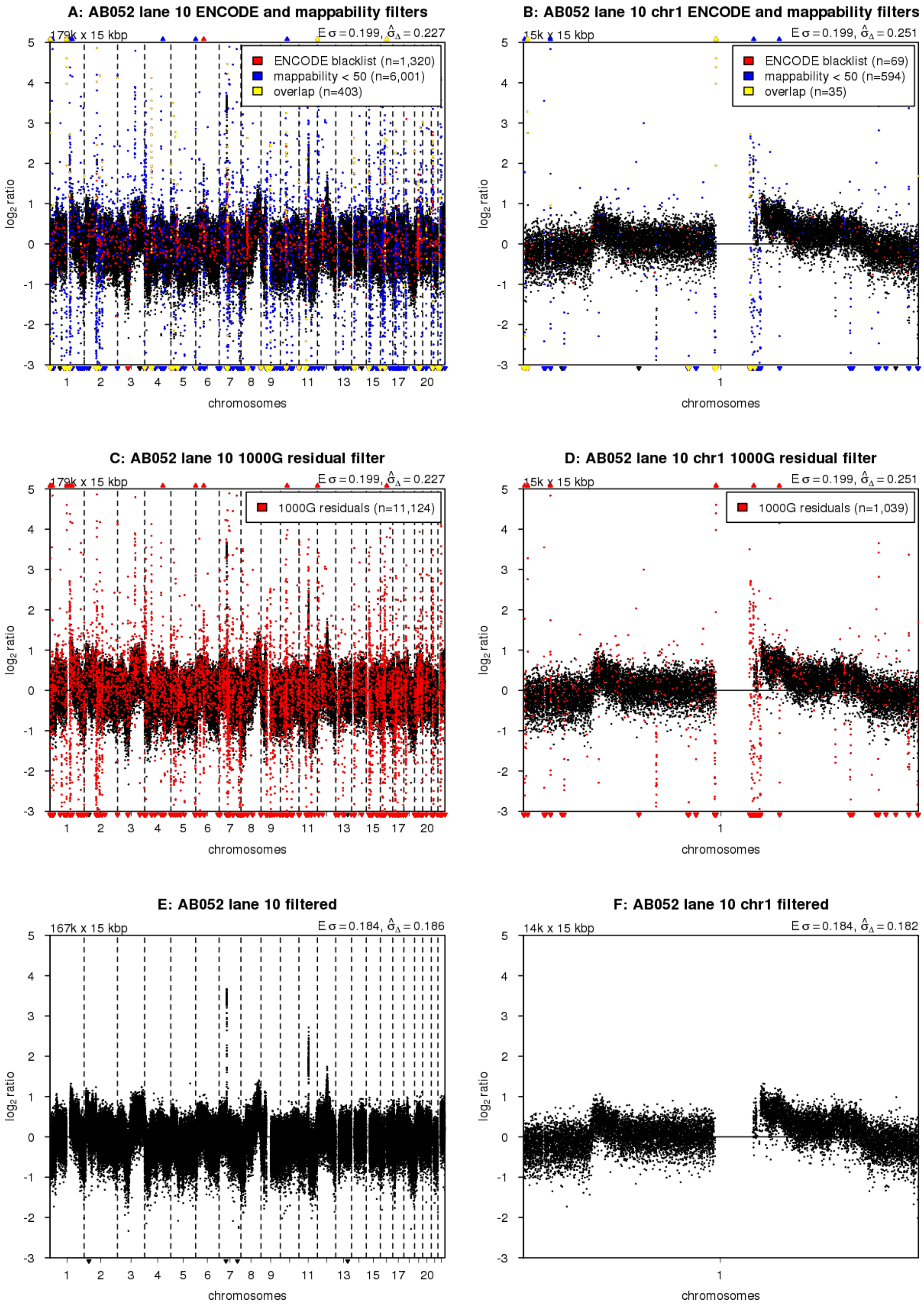

Figure S3: Blacklisting problematic regions (cont.)

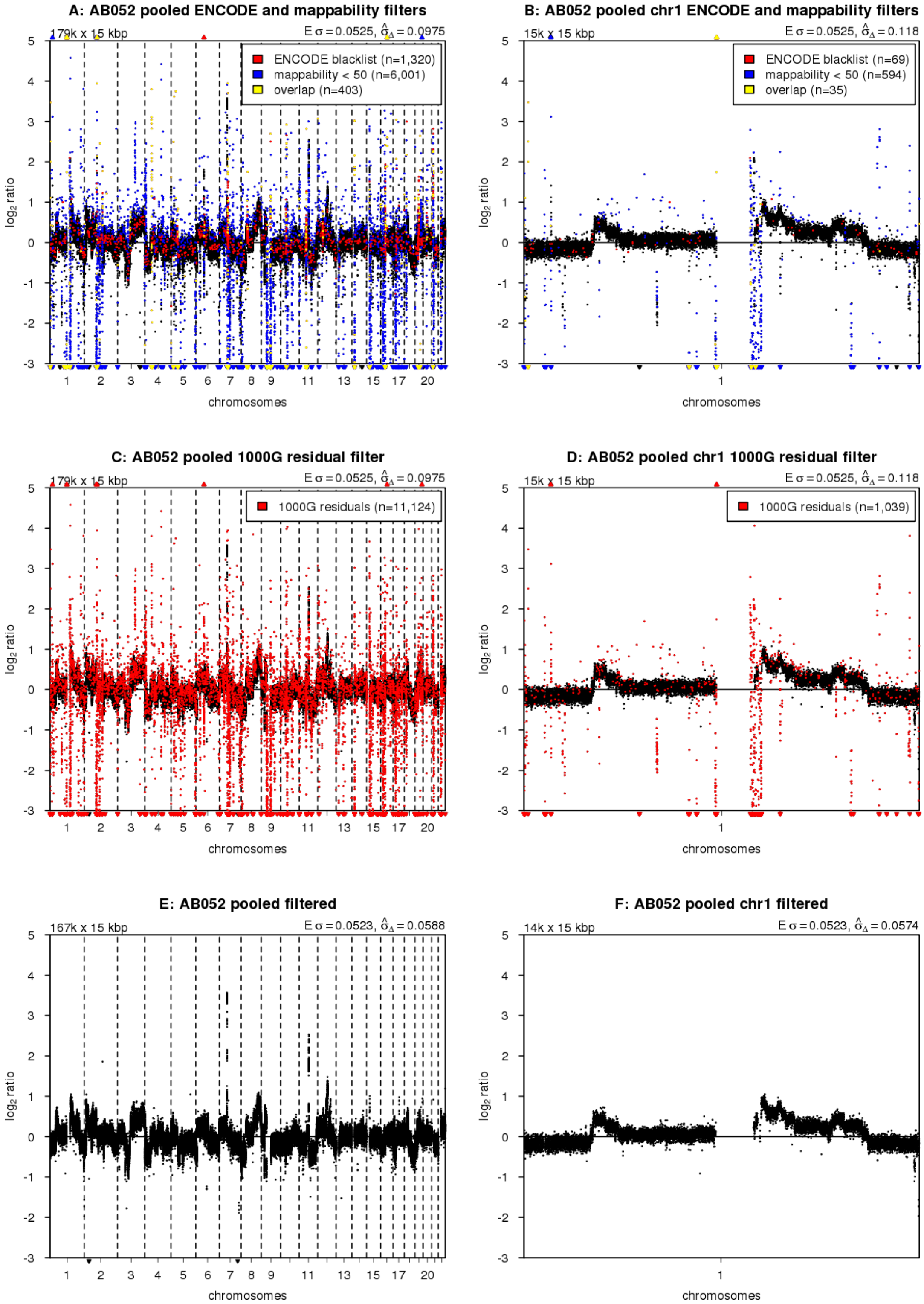

Figure S3: Blacklisting problematic regions (cont.)

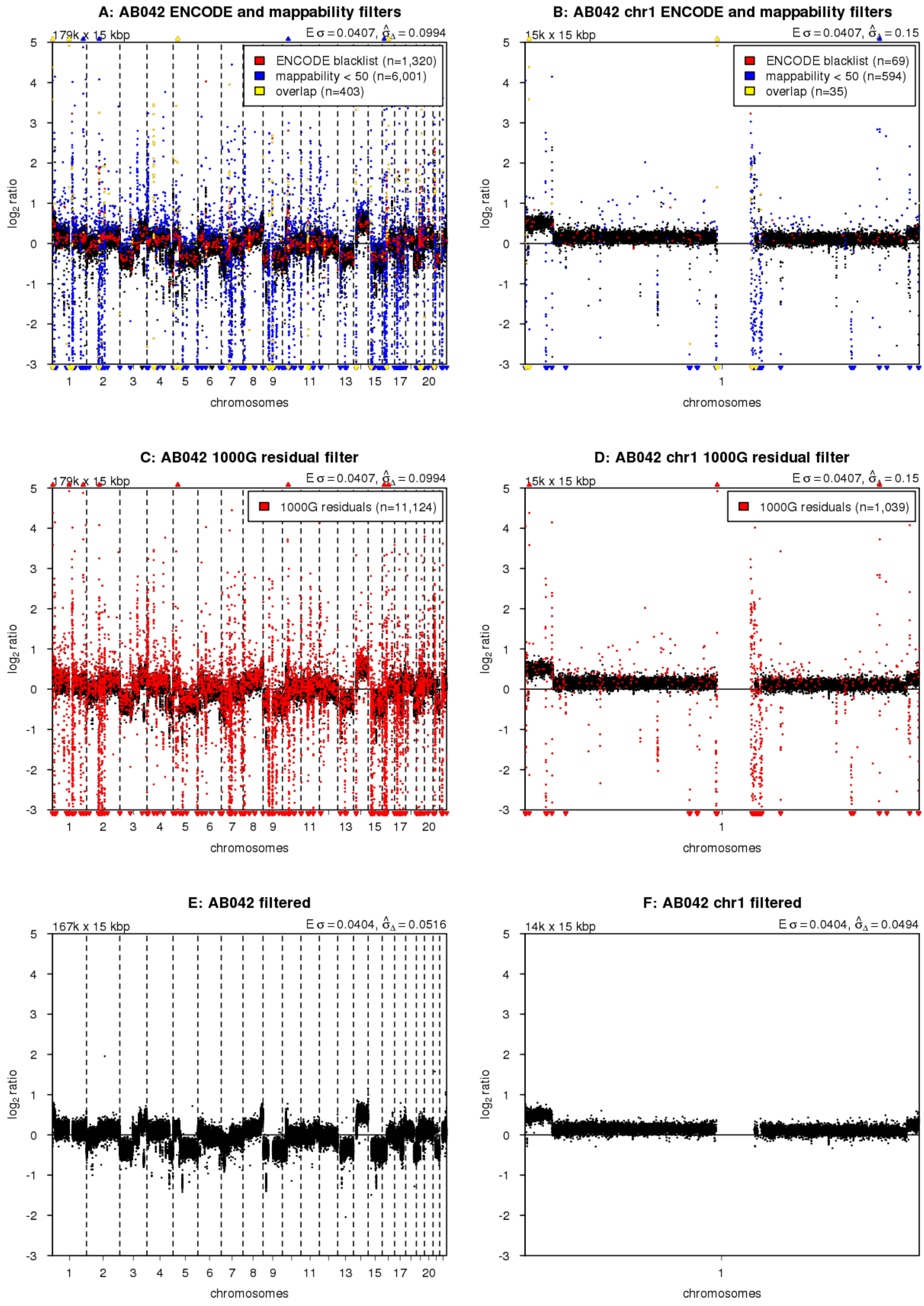

Figure S3: Blacklisting problematic regions (cont.)

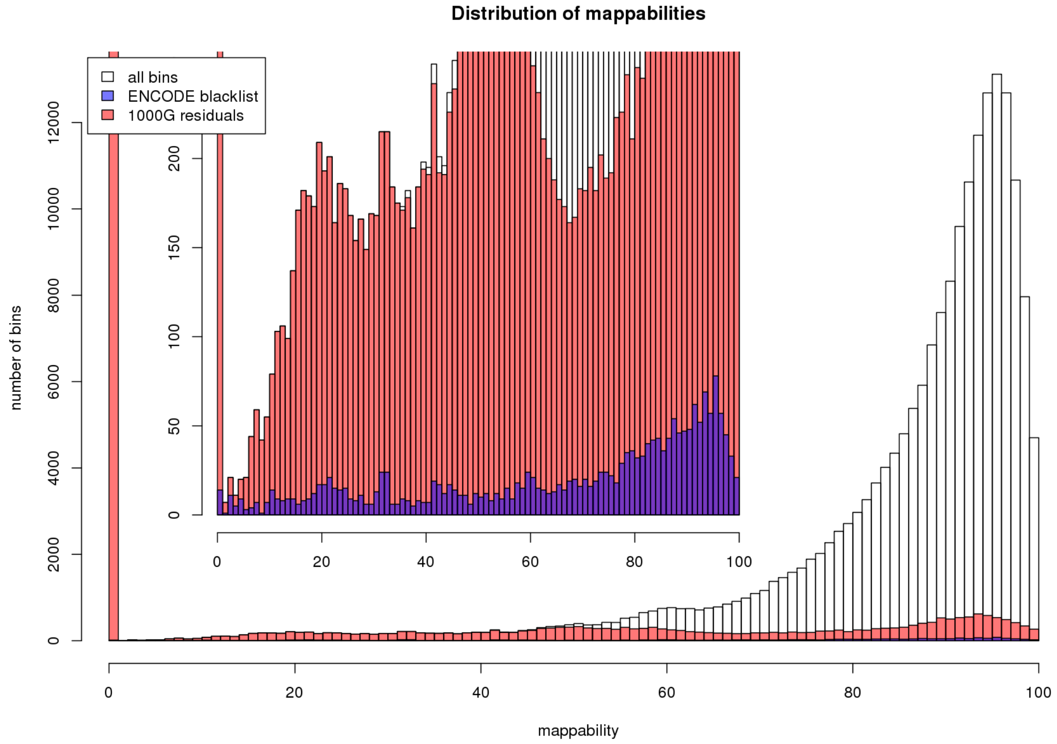

Figure S4: Distribution of mappabilities of 179,187 autosomal bins 15 of kbp. White bars show the number of bins with different mappabilities. Filled with red are the number of bins covered by the novel blacklist based on LOESS residuals and 38 samples from the 1000 Genomes project, and filled with blue are the number of bins with any overlap with ENCODE blacklisted regions. The inset shows a zoomed-in version of the lower part of the graph.

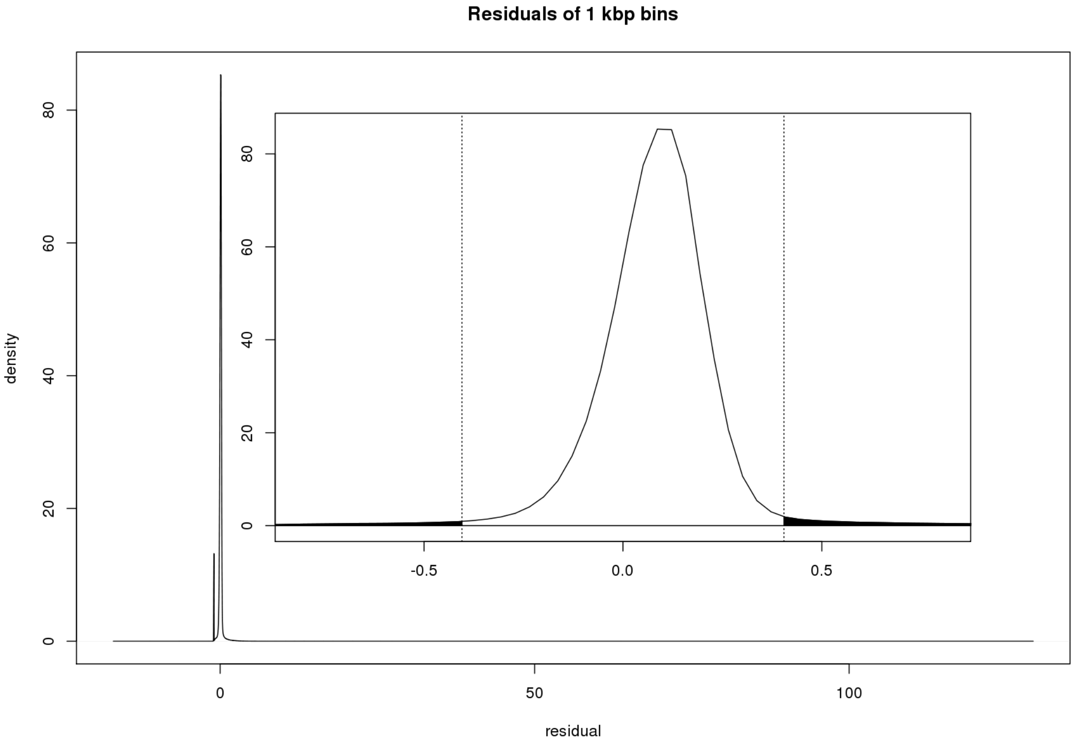

Figure S5: Distribution of median residuals per bin from the 1000 Genomes project across the 38 samples. Residuals are defined as the distance between observed read counts and the fitted LOESS surface. The outer plot shows the entire range of values with two discrete peaks. The minor peak around -1.0 results from repetitive sequences. Reads that align equally well to multiple locations in the genome are filtered out. Repetitive sequences therefore have a lower than expected number of reads mapped. The major peak around zero contains most of the bins, and the inset shows a magnification of the peak, with the dotted vertical bars and the shaded area showing the cutoff of 4.0 standard deviations (as estimated with a robust first-order estimator) for blacklisting. Distribution of median residuals are given for 1 kb bins in the graph on this page and for the 5, 10, 15, 30, 50, 100, 500 and 1000 kb bins on the 4 subsequent pages.

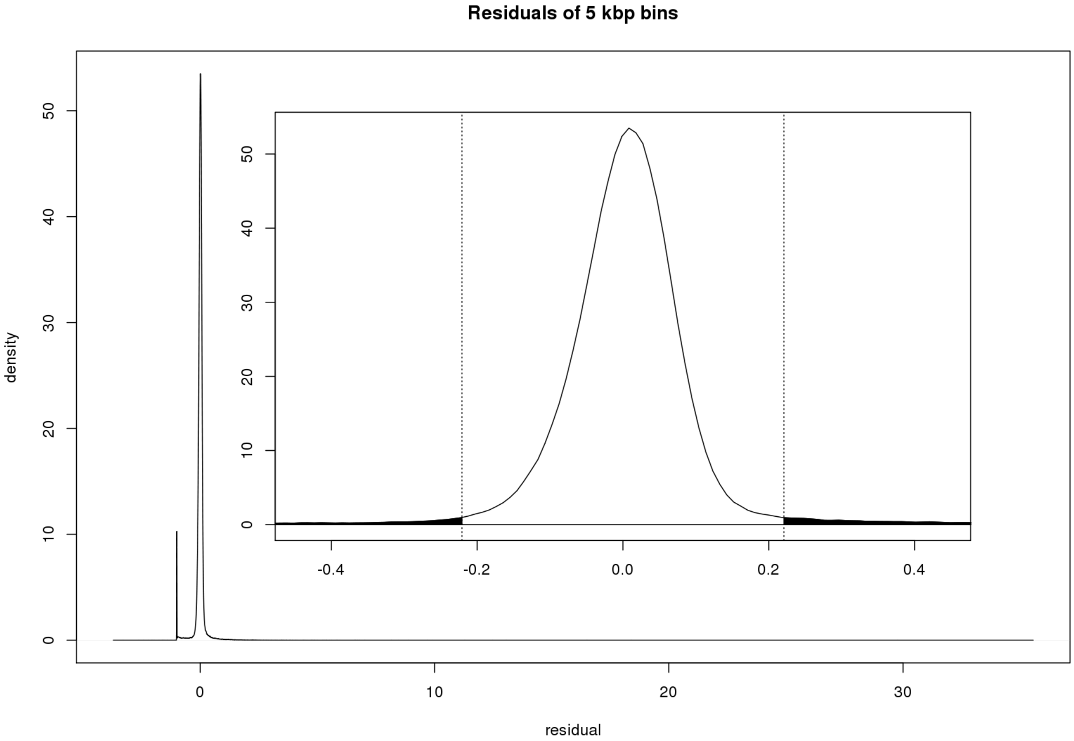

Figure S5: Distributions of median residuals (cont.)

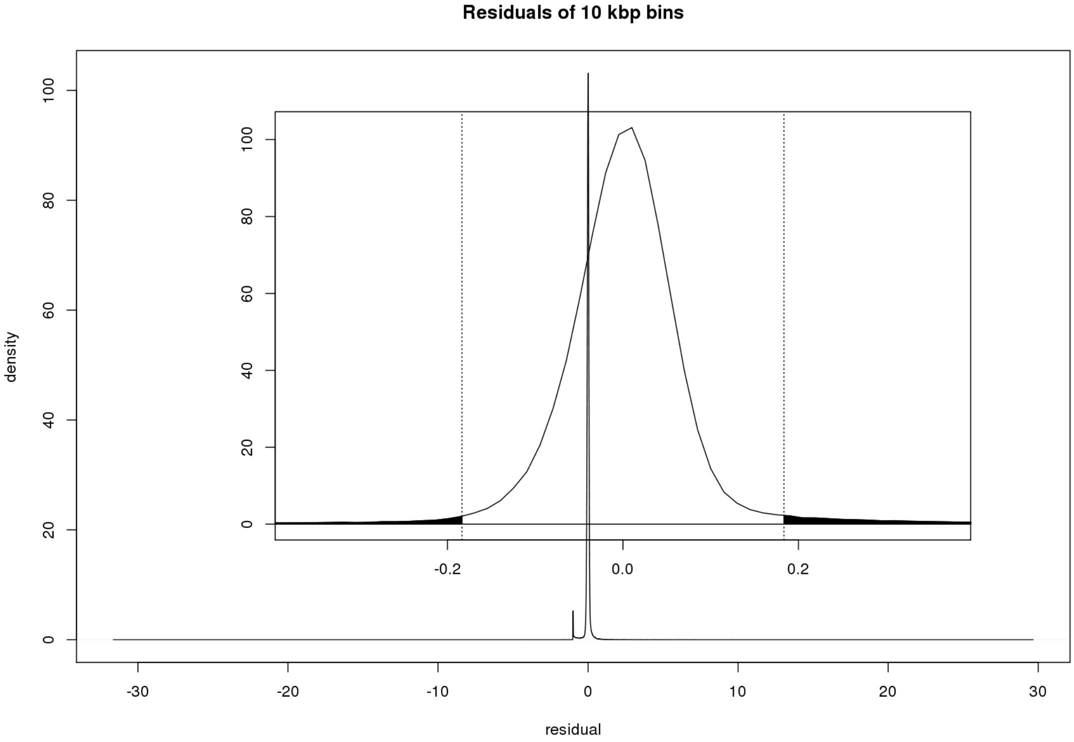

Figure S5: Distributions of median residuals (cont.)

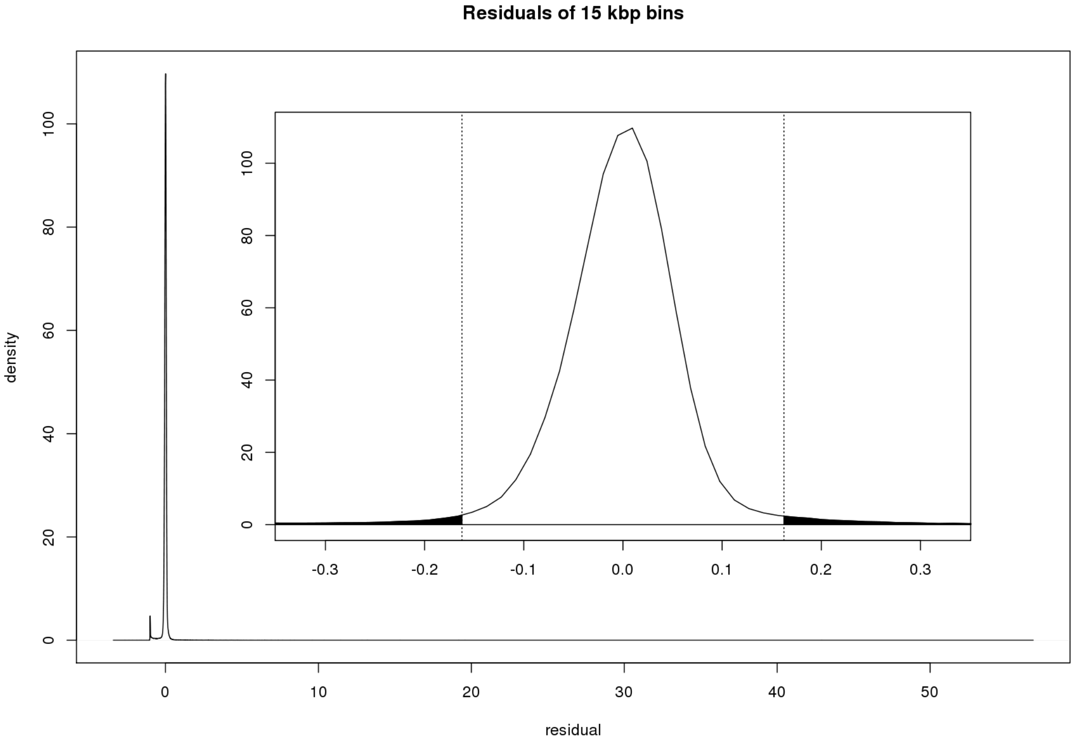

Figure S5: Distributions of median residuals (cont.)

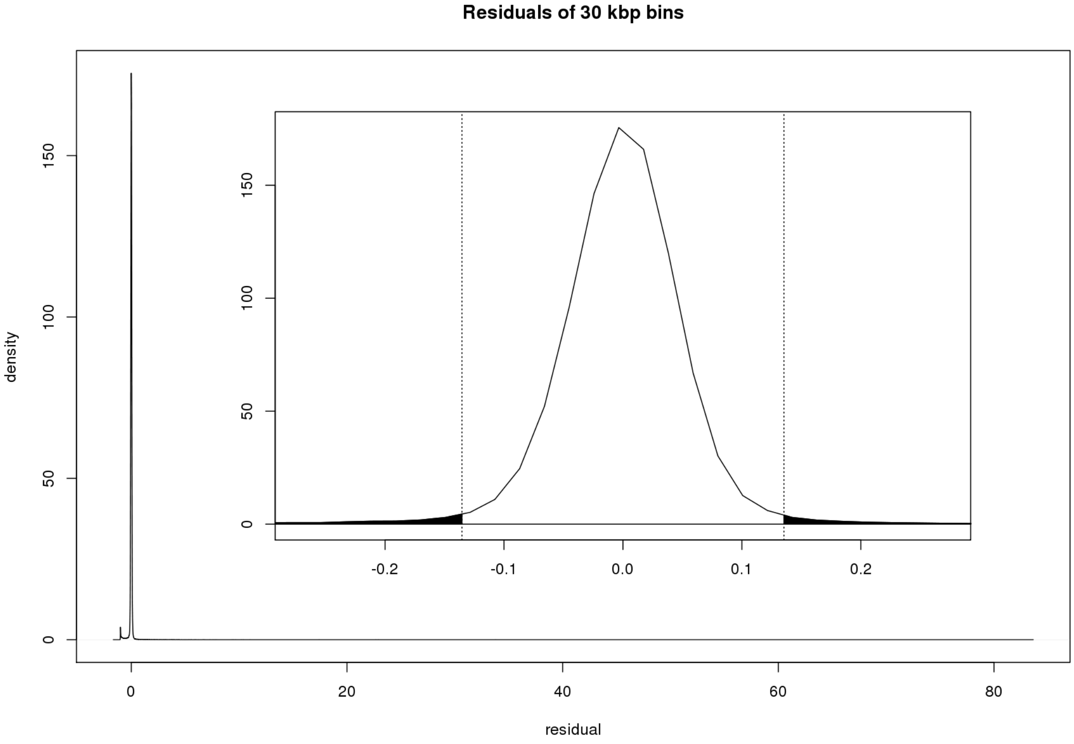

Figure S5: Distributions of median residuals (cont.)

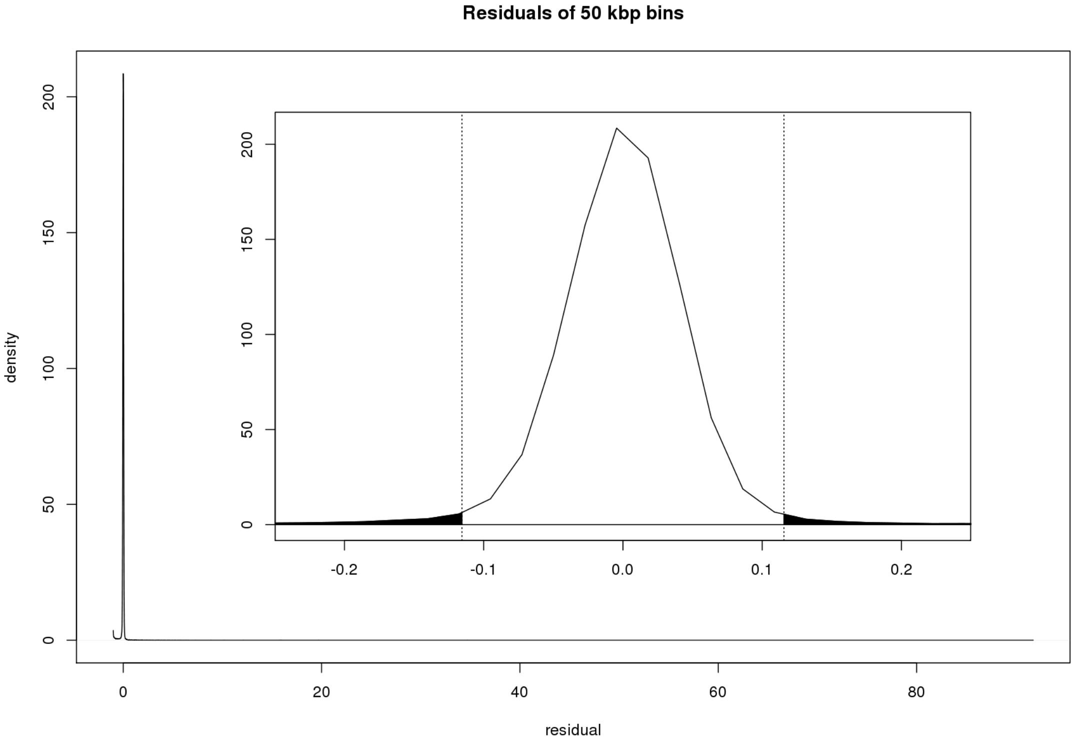

Figure S5: Distributions of median residuals (cont.)

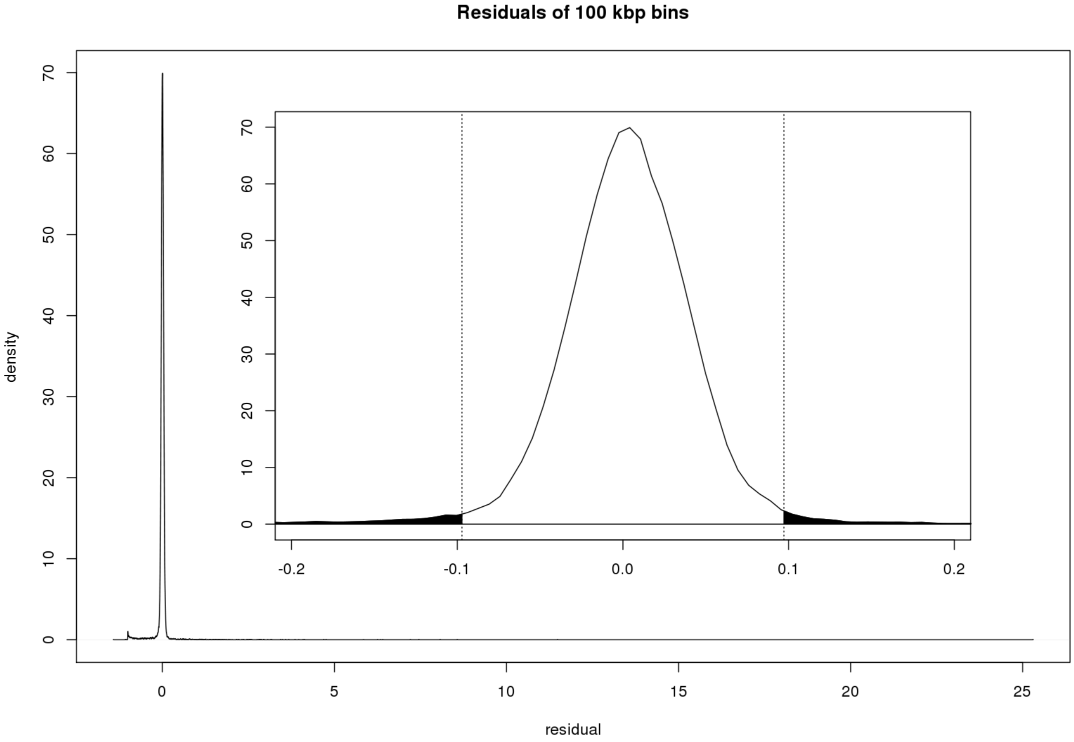

Figure S5: Distributions of median residuals (cont.)

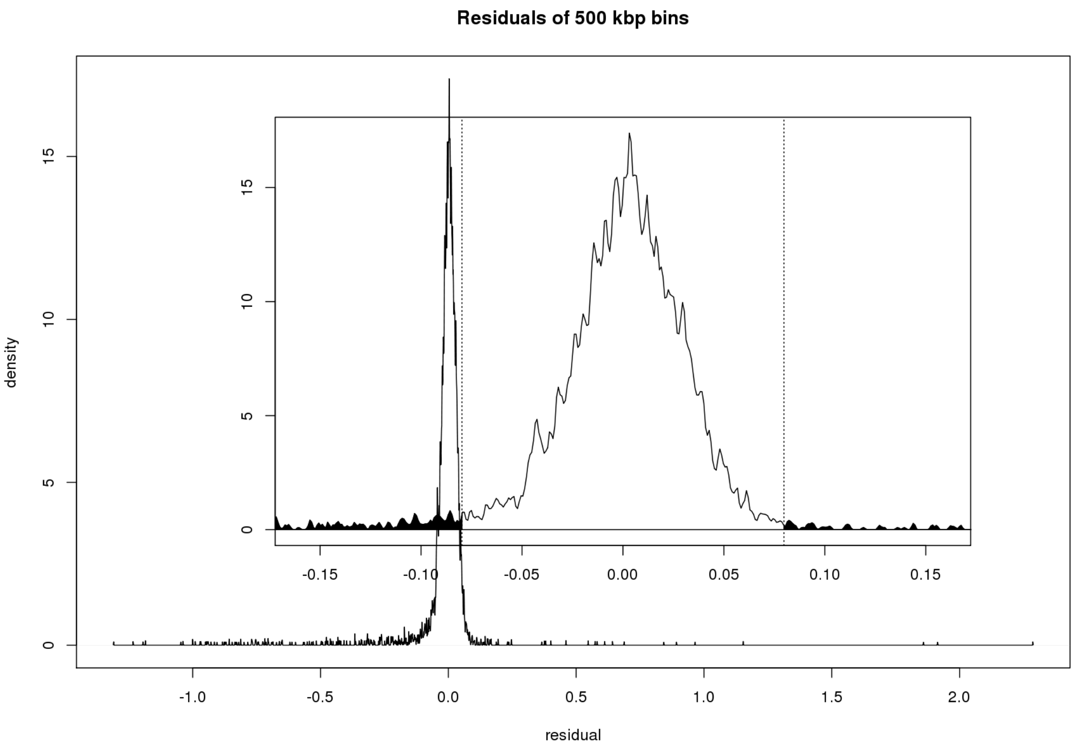

Figure S5: Distributions of median residuals (cont.)

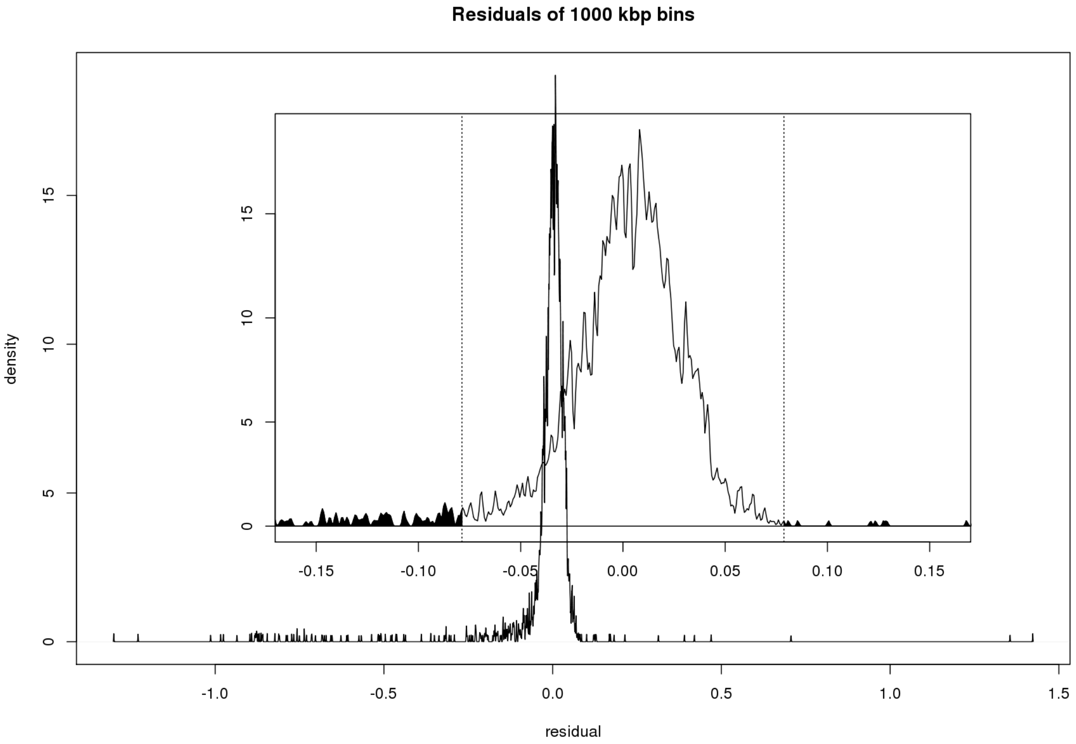

Figure S5: Distributions of median residuals (cont.)

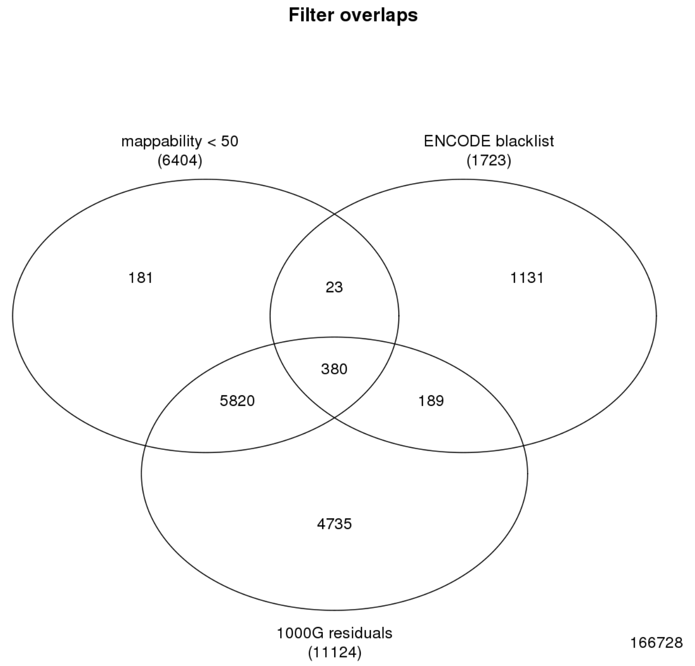

Figure S6: Venn diagram showing the overlaps between bins with mappabilities below 50 (6,404 bins), overlapping with the ENCODE blacklist (1,723 bins), or contained in the novel blacklist based on median LOESS residuals of 38 samples from the 1000 Genomes project (11,124 bins). 166,728 autosomal bins are not contained in any of these three blacklists.

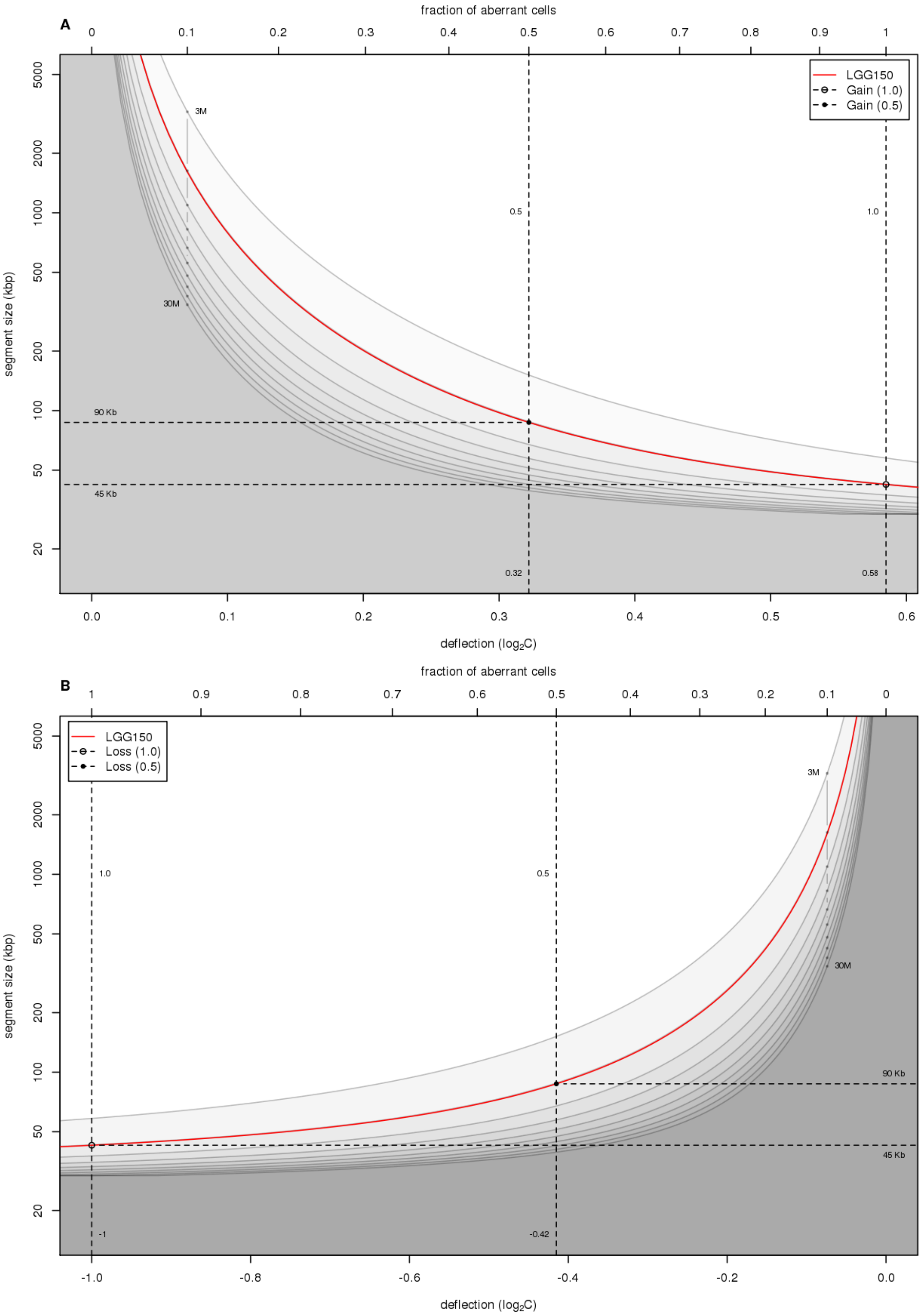

Figure S7: Detection limits for (A) gains and (B) losses. See next two pages for details.

The smallest copy number aberration that can be reliably detected depends on many factors including the magnitude of its copy number deviation, the noise level, and the acceptable error rates. The magnitude of a copy number aberration in the DNA sample depends on its magnitude in the cells in which it occurs and the fraction of cells that contain it. The fraction of cells with the aberration depends on intratumor heterogeneity and the presence of normal cells. The noise level includes the contribution from read count statistics, whose magnitude and distribution over the genome are predictable from the sequencing depth, as well as variations due to other sources which may affect localized regions of the genome and occur sporadically. Detection limitations imposed by read count noise can be addressed by modeling, but the presence of the other types of noise can produce false positive indications of abnormalities that cannot be modeled, emphasizing the importance of improving sequencing and read count correction procedures in order to reduce them.

Here we present modeling data and analytic formulae that provide estimates of the detection limits as a function of the magnitude of the copy number aberration and the read depth for various levels of statistical significance, assuming that only noise due to read counting statistics is relevant. As we have shown, read depth makes the overwhelming contribution to the noise in most of our samples. However, there are other sources of variation so that the estimates we present here represent a best-case scenario.

Imagine the simplest type of heterogeneity where a cell population consists of a fraction A, which ranges from a 0 to 1.0, of cells with the copy number aberration, mixed with a fraction $1 - A$ of normal cells. Assume the genome of the abnormal cells is basically diploid but contains a copy number aberration of magnitude C_T, normalized relative to diploid, and segment size $G = \beta L$, where β is the size of a bin and L is the number of bins in the aberration. Thus C_T is 1.5 for a single copy gain and 0.5 for a single copy loss. The relative copy number C of the aberration in the DNA sample from the entire population is:

$$C = AC_T + 1 - A = A(C_T - 1) + 1$$

Panel A shows the minimum detectable segment size G (in kb) for detection of a single copy gain based on data from sample LGG150. The curves give G (segment length y-axis) as a function of $\log_2 C$, (deflection, x-axis bottom), which is a function of A (fraction of cells with the copy number aberration, x-axis top), for read depths ranging from 3 million to 30 million (in steps of 3 million). The corresponding result for detection of a single copy loss is shown in Panel B. The curves are calculated using a one sided t-test (Krijgsman et al., 2013) with statistical power 0.8 for detection of the aberration and requiring $p < 0.01$. Sample LGG150 had $\sim$ 6.5 million reads distributed over 179k bins of size 15 kb, or about 36 reads per bin. Its noise level was as expected based on read counting statistics, measured standard deviation of 0.166. This noise was scaled for the read depths used in the calculation. The t-test calculation required that an aberration contain at least two bins giving a minimum segment size of 30 kb for the 15 kb bins used here, so that the curves are affected by this lower limit. The red lines show the expected behavior for sample LGG150. The open circles show the size of the minimum detectable segment for a single copy gain or loss if the sample consists entirely of aberrant cells, while the solid dots show the detection limit when the sample contains 50% normal cells.

The statistical criterion of p-value 0.01 used for these calculations is appropriate for evaluating whether or not a pre-defined region of the genome is at aberrant copy number. However, if one desires to examine the entire genome to discover aberrations at any location it would generate a considerable number of false positives. For discovery purposes it would be more appropriate to use more stringent criteria on the order of $p < G/(3 \times 10^6)$, where 3×10^6 is the number of kb in the genome. This is on the order of 10^{-4} or 10^{-5} for aberrations on the order of 100kb.

A simplified analytical expression can be derived that contains the essential characteristics of the detection problem and displays the dependence of the detection limits on the relevant parameters. In order to detect an aberration one needs to set thresholds for gain and loss and determine whether a segment of the genome lies inside or outside of them. A normal region of the genome of length L bins will contain LN reads, which have a standard deviation $[LN]^{1/2}$, where N is the average read counts per bin for the genome. The detection thresholds are set at values $1 \pm \eta[LN]^{1/2}$, where η is chosen to set the p value, and the + and − signs correspond to thresholds for gains and losses respectively. For example $\eta \sim 2.58$ will give $p < 0.01$ that a truly normal segment of the genome will fall outside the thresholds by chance. Similarly $\eta \sim 4$ or a bit more will give $p < 10^{-5}$, which would be appropriate for discovery of novel aberrations.

Similarly, a genomic region of length L that is truly at copy number C will contain CNL reads with a standard deviation of $[CNL]^{1/2}$. In order for this aberration to be reliably detected it must be above the threshold by $\kappa[CNL]^{1/2}$, where $\kappa \sim 0.85$ will give probability of 0.8 to detect the aberration if it is truly present. Thus a gain of length L will be above threshold if

$$CNL - \kappa[CNL]^{1/2} > NL + \eta[NL]^{1/2}$$

A similar relation holds for losses with appropriate sign changes. Solving for L one obtains for both gains and losses:

$$L = \frac{1}{N[C-1]^2}[\kappa\sqrt{C} + \eta]^2$$

The t-statistic-based curves in the Panels A and B follow this equation reasonably well with $\kappa \sim 0.85$ and $\eta \sim 2.58$, except as the lengths decrease towards 2 bins (30 kb for 15 kb bins) due to the limit of a minimum of 2 bins imposed in the full calculation. The equation shows that if $\eta \sim 4$ or 5, which is appropriate for detection of novel aberrations in the genome, the minimum detectable single copy gain and loss are $\sim$ 50 to 60 kb for a sample that is homogeneously abnormal ($A = 1$) and for which 6 million reads were obtained. If the sample contains half normal cells, $A = 0.5$, these number increase a factor of 4 or so. The $\sim A^{-2}$ behavior of the minimal detection length for low p (large η) can be seen by substituting the expression for C into the equation for L.

We note that the fundamentally important feature that governs the detection is the number of read counts in the length of the aberrant region, the product LN. For $\sim$ 6 million reads and 15 kb bins as with sample LGG150, the analysis shows that single copy aberrations as short as 3 bins are potentially detectable, and that may be too few bins to allow exclusion of artifactual variation. If there are sufficient reads for a sample there may be benefit in decreasing the bin size so that there will more bins in an aberration of a given size, and a cutoff on the minimum number of bins required to define an aberration will allow shorter aberrations to be above this threshold. Smaller bin sizes will also permit sharpening the boundaries of aberrations, especially for high level amplifications (large C) and homozygous deletions ($C \sim 0$).

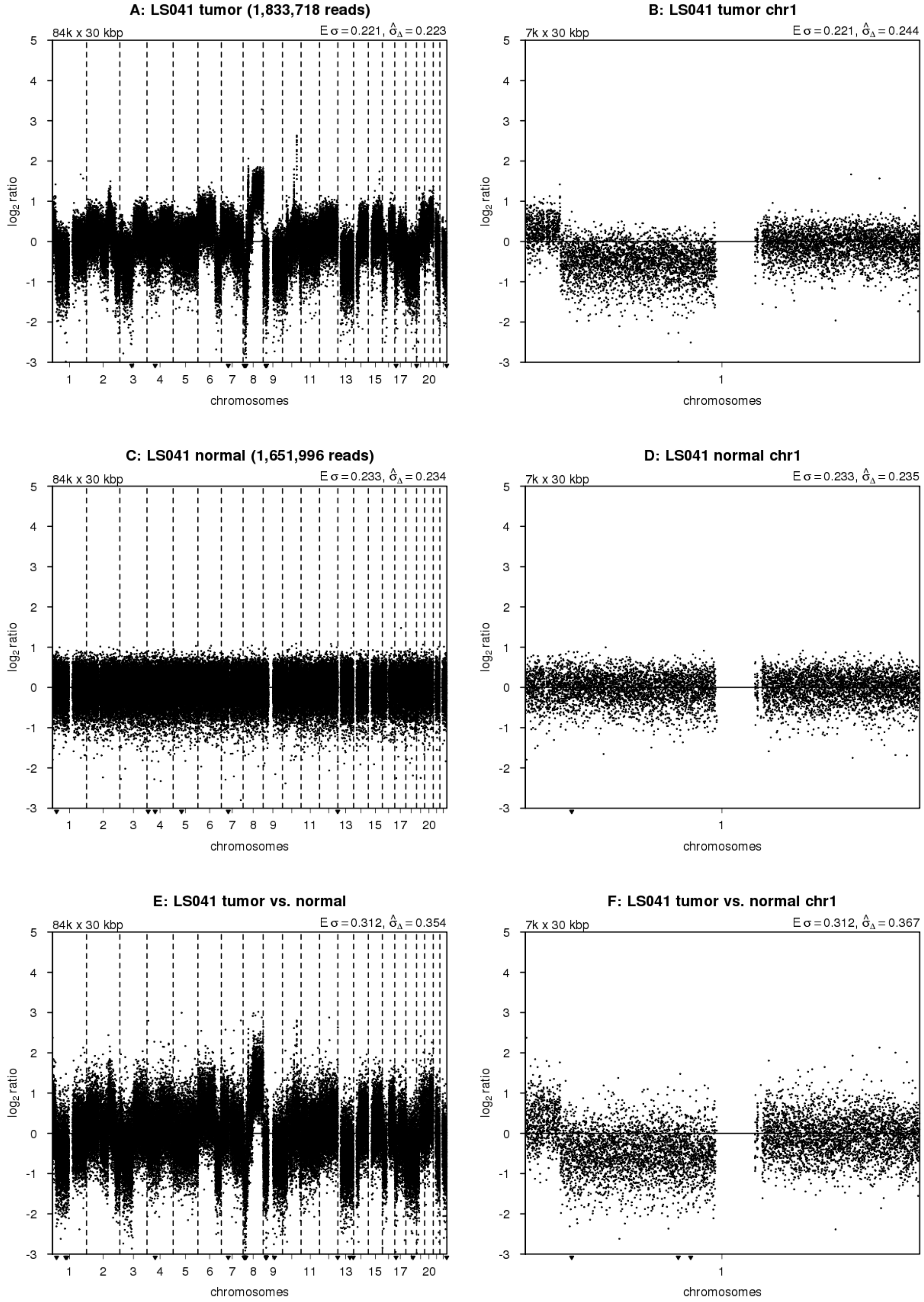

Figure S8: Using a reference sample. Publicly available data for a fresh-frozen lung tumor LS041 and a paired blood sample were downloaded (Gusnanto et al., 2012), and copy number profiles are shown for (A) the whole genome and (B) chromosome 1 of the tumor, and (C) the whole genome and (D) chromosome 1 of the matched normal. The comparative tumor *vs.* normal profile is shown for (E) the whole genome and (F) chromosome 1. The standard deviations ($\hat{\sigma}_\Delta$) were quantified as 0.223 for tumor (0.234 for normal) and 0.354 for the tumor vs. normal comparison, which is close to the expected $\sqrt{2} \approx 1.4$-fold increase. Bins are ordered along the x-axis by their genomic positions, and y-axis shows median-normalized $\log_2$-transformed data. Small triangles at the top and bottom edges represent data points that fall outside the plot area. Top-left corners show the number and size of bins. Top-right corners show the expected and measured standard deviation. The expected standard deviation (E σ) is defined as $\sqrt{1/N}$, where N is the average number of reads per bin. The measured standard deviation ($\hat{\sigma}_\Delta$) is calculated from the data with a 0.1%-trimmed first-order estimate (prior to $\log_2$-transforming the data for plotting).

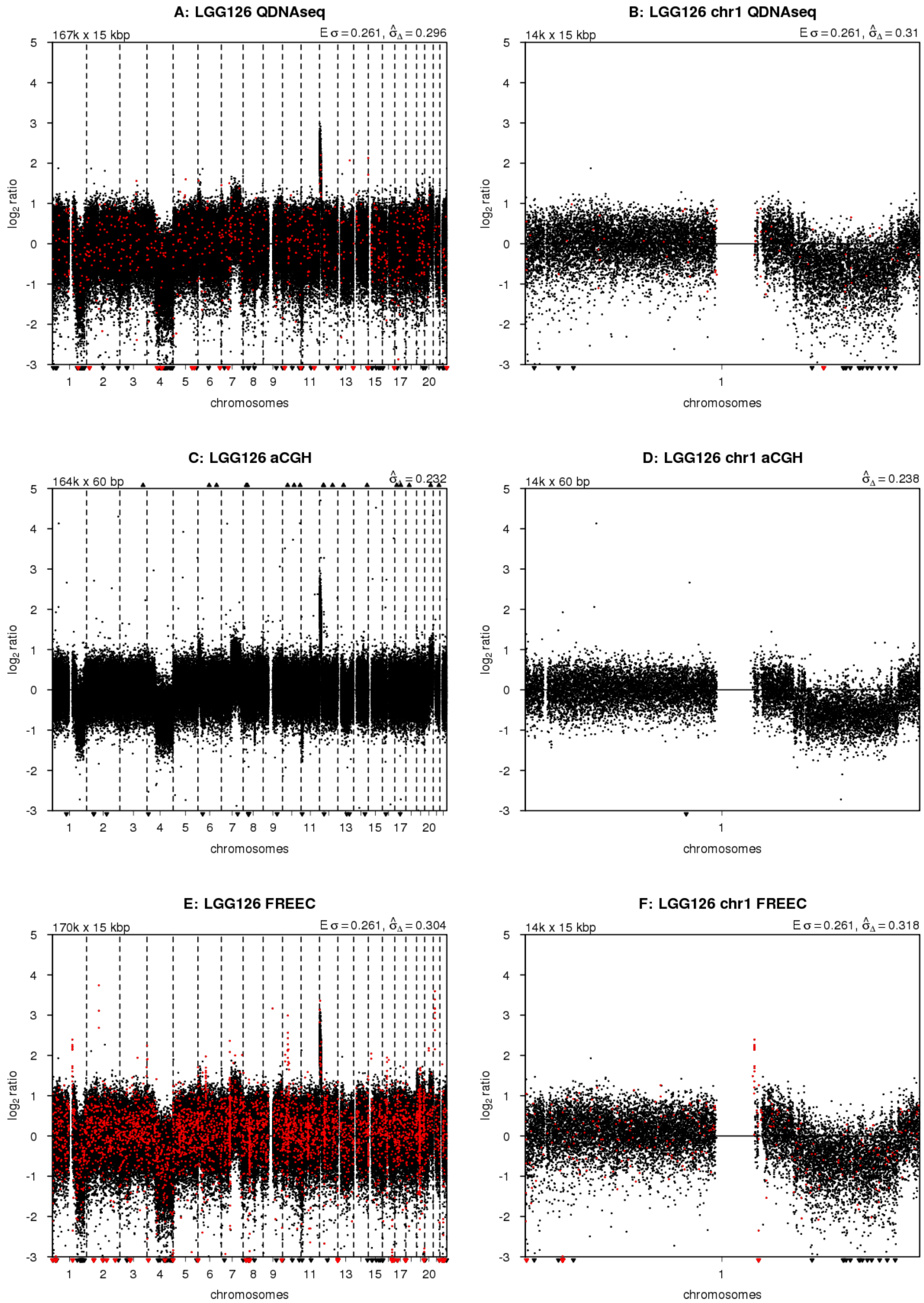

Figure S9: Comparisons to other methods. Final copy number profile obtained with QDNAseq after removing blacklisted bins and correcting read counts for GC content and mappability for (A) the whole genome and (B) chromosome 1. Highlighted in red are bins that are not contained in the output of FREEC. Copy number profile obtained with an Agilent 180K microarray with 164,378 unique array elements for (C) the whole genome and (D) chromosome 1. Copy number profile obtained with FREEC for (E) the whole genome and (F) chromosome 1. Highlighted in red are bins that are not contained in the output of QDNAseq. Bins are ordered along the x-axis by their genomic positions, and y-axis shows median-normalized $\log_2$-transformed data. Small triangles at the top and bottom edges represent data points that fall outside the plot area. Top-left corners show the number and size of bins. Top-right corners show the expected (sequencing methods only) and measured standard deviation. The expected standard deviation (E σ) is defined as $\sqrt{1/N}$, where N is the average number of reads per bin. The measured standard deviation ($\hat{\sigma}_\Delta$) is calculated from the data with a 0.1%-trimmed first-order estimate (prior to $\log_2$-transforming the data for plotting).

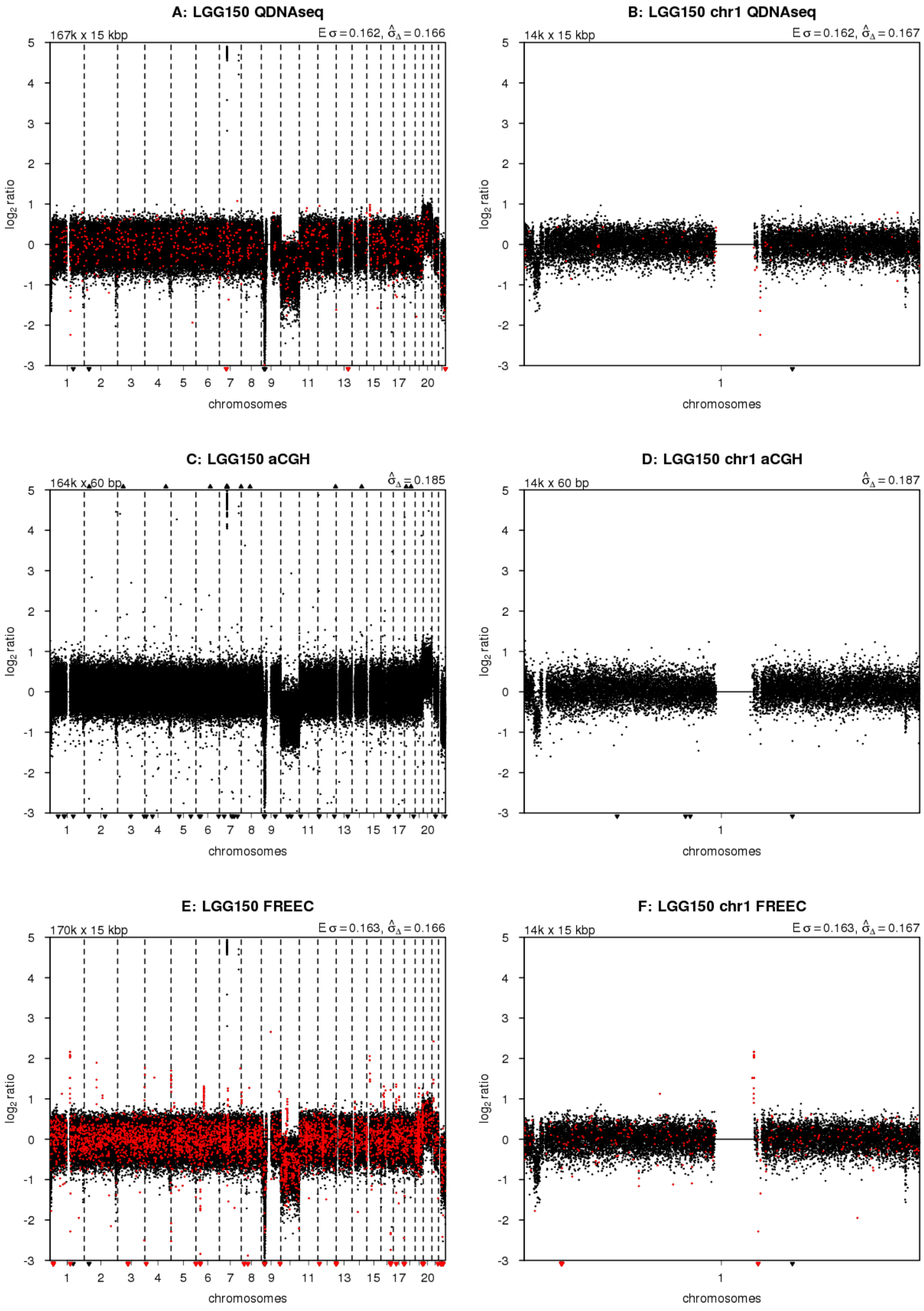

Figure S9: Comparisons to other methods (cont.)

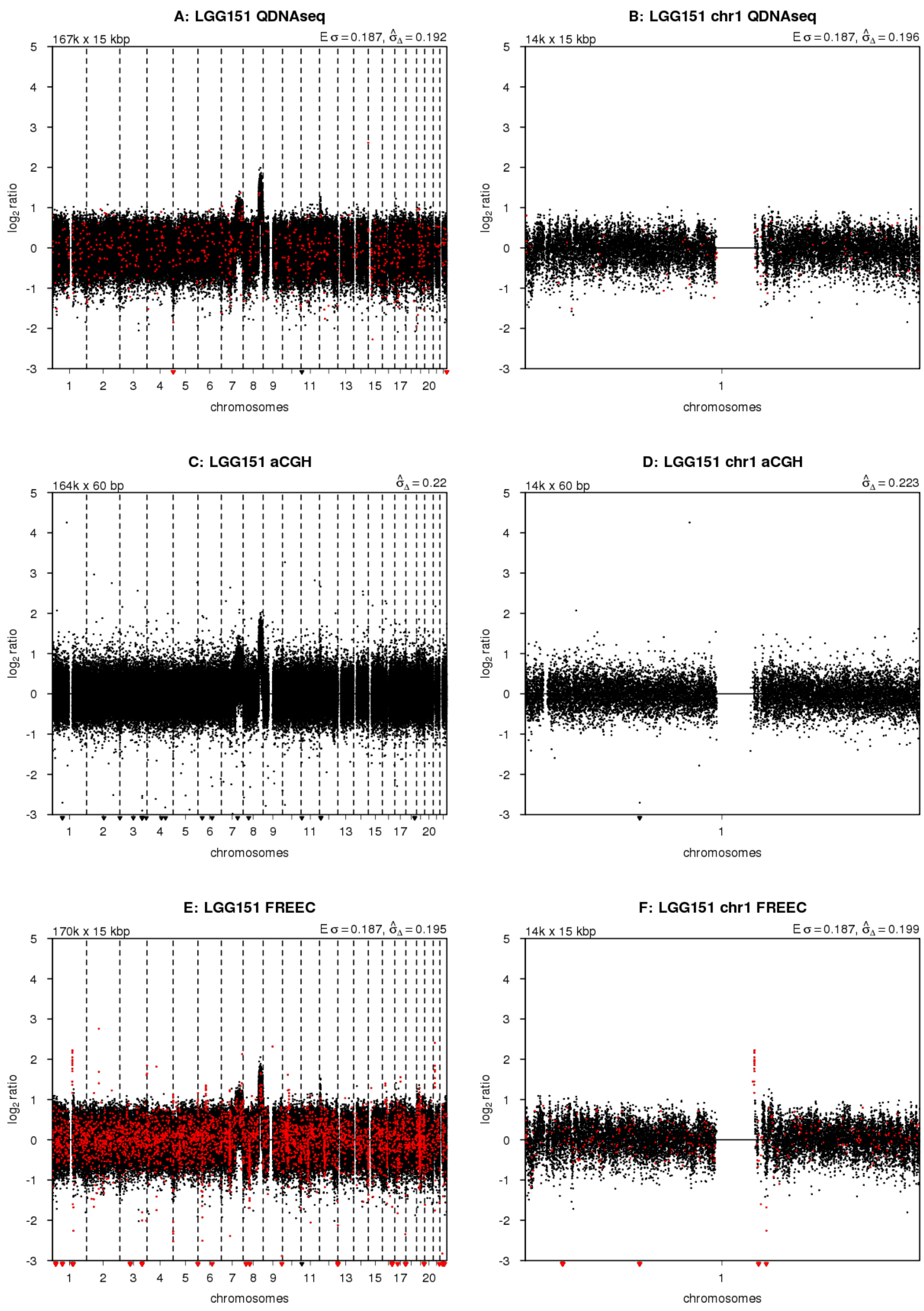

Figure S9: Comparisons to other methods (cont.)

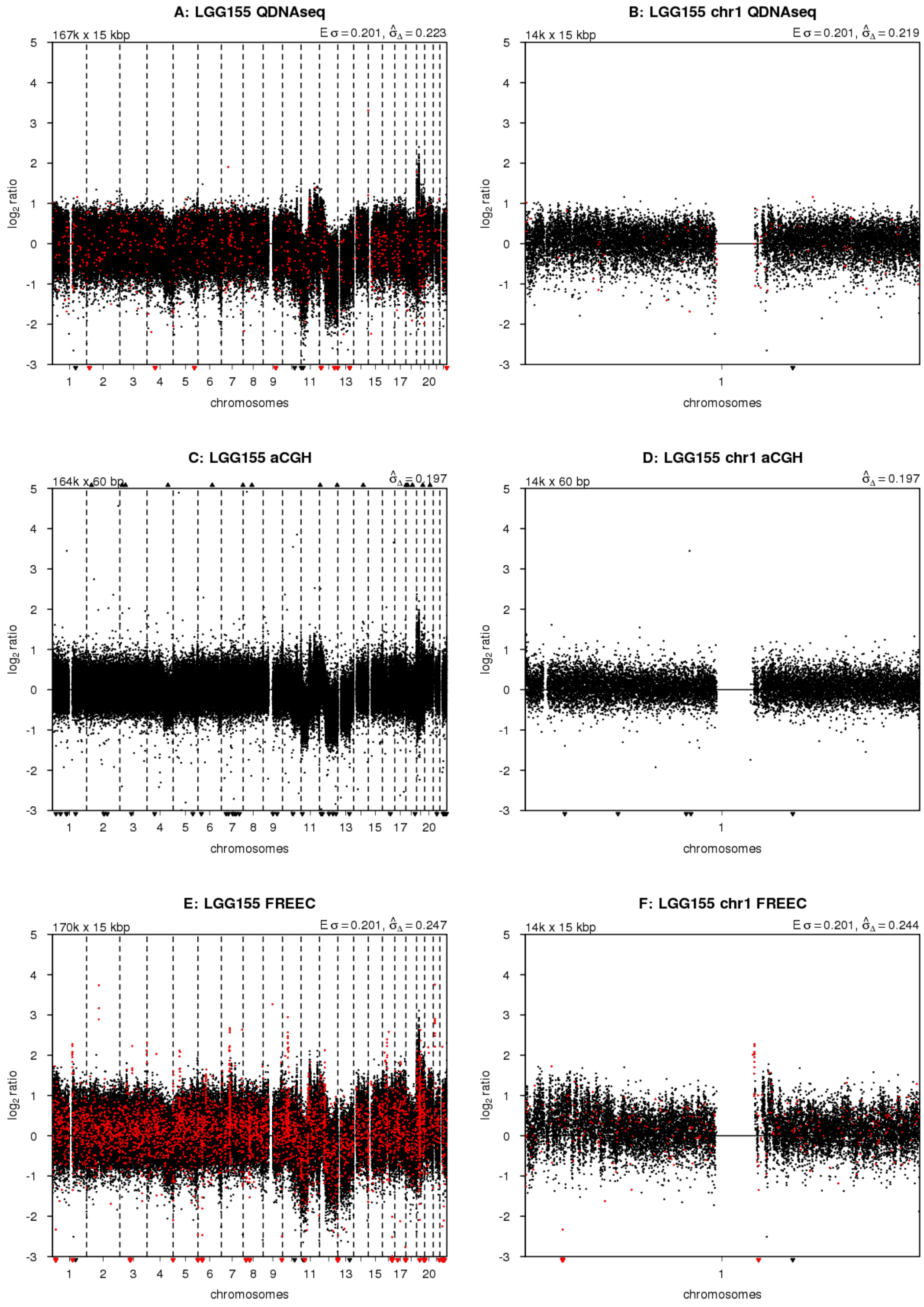

Figure S9: Comparisons to other methods (cont.)

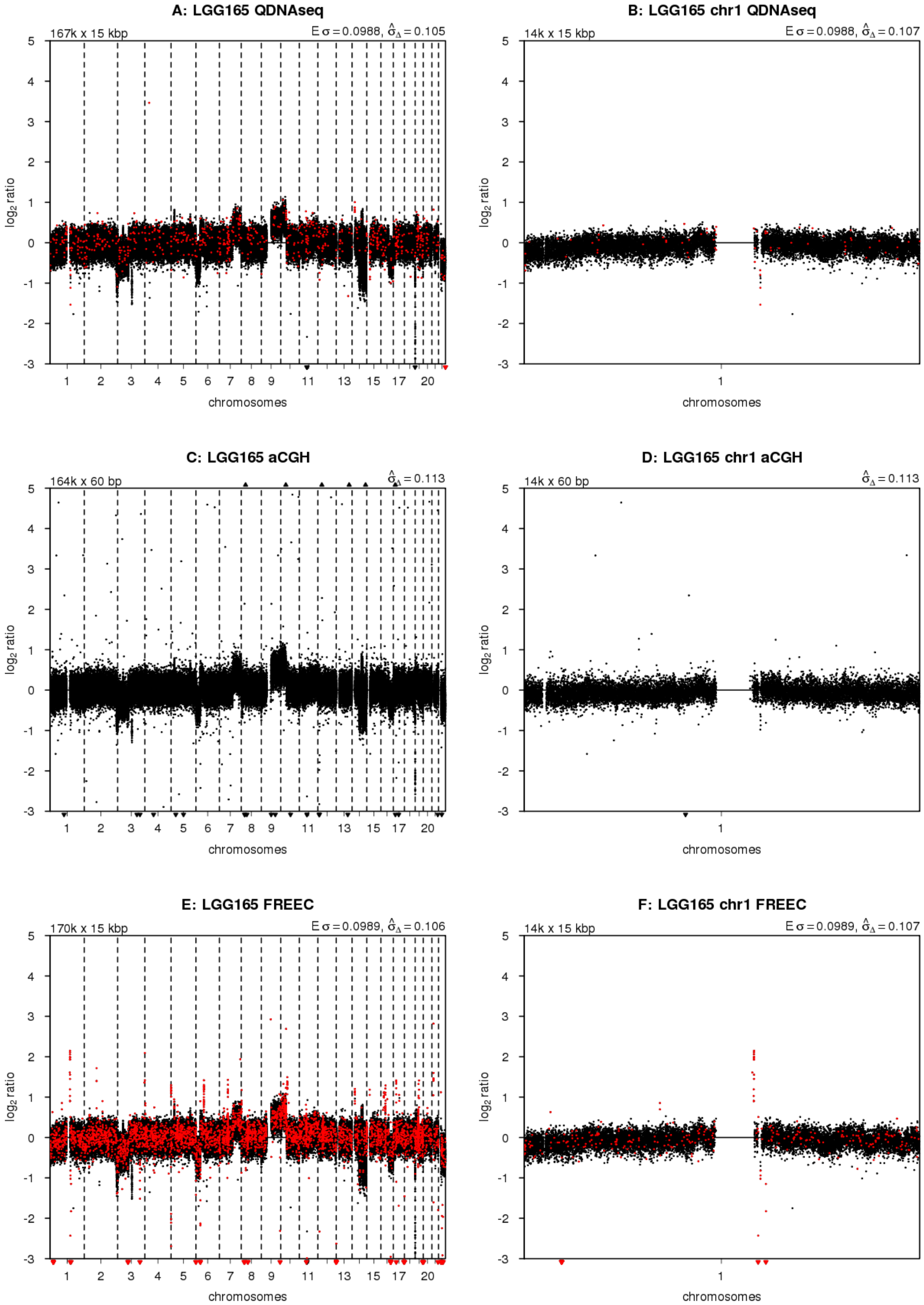

Figure S9: Comparisons to other methods (cont.)

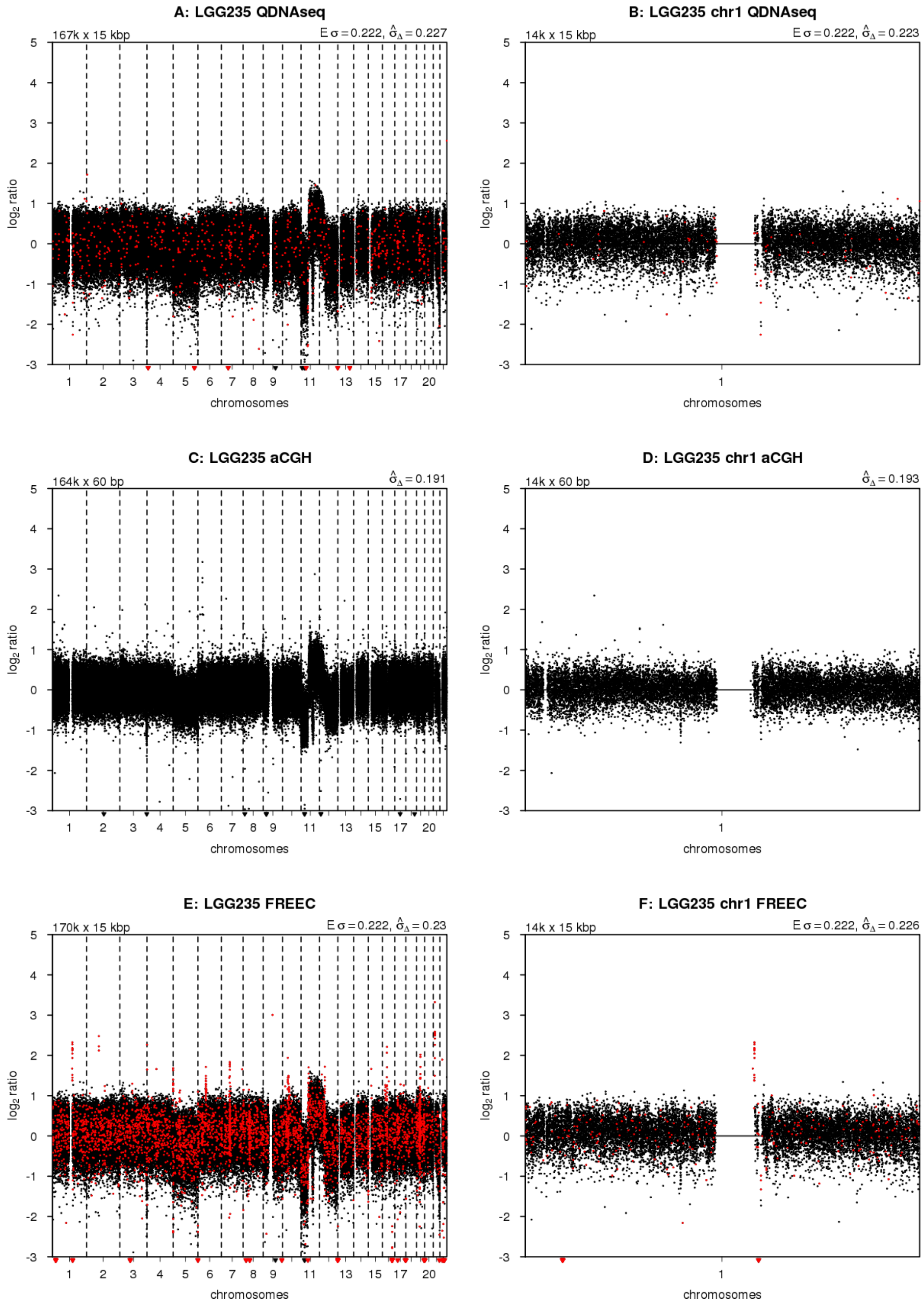

Figure S9: Comparisons to other methods (cont.)

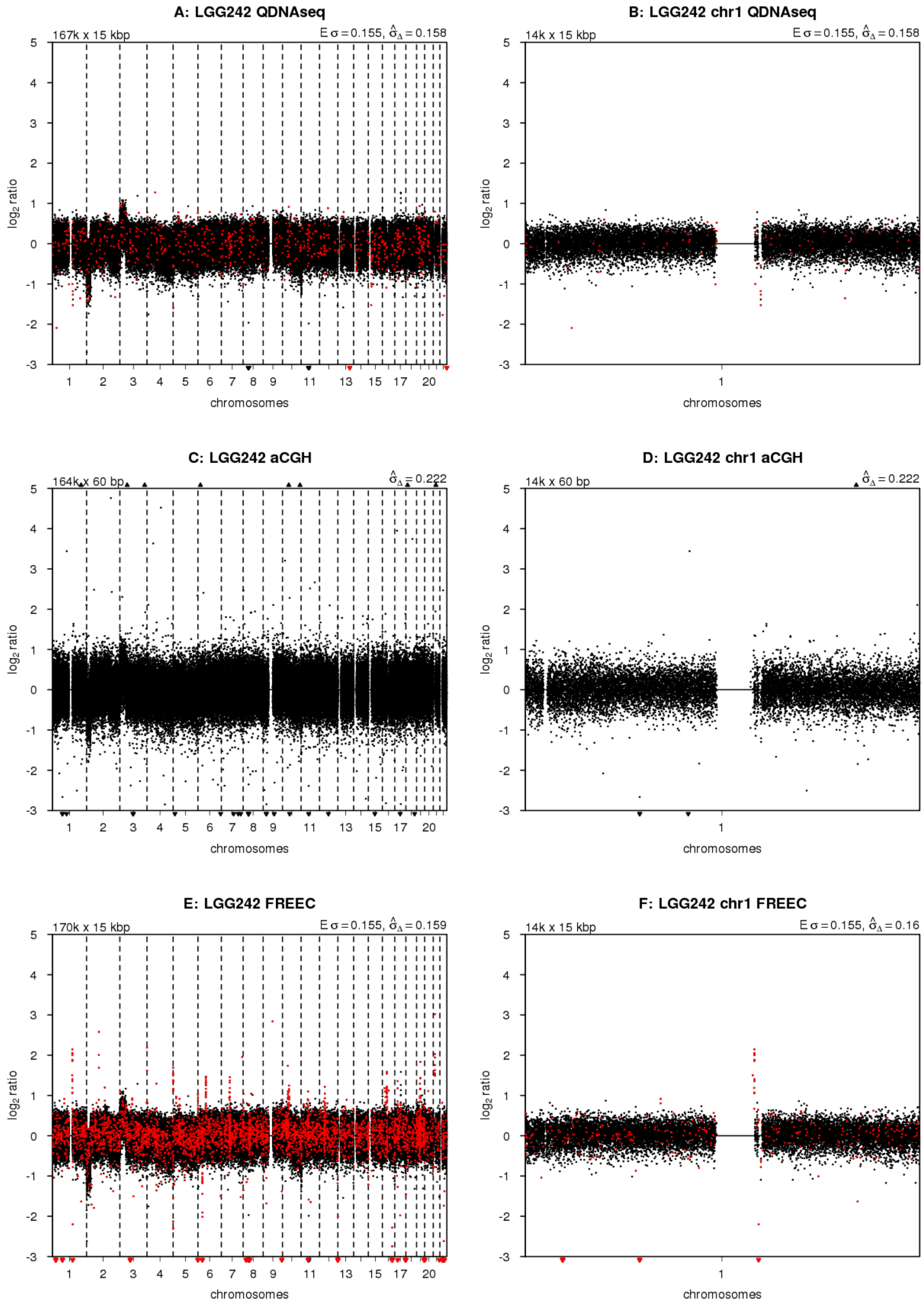

Figure S9: Comparisons to other methods (cont.)

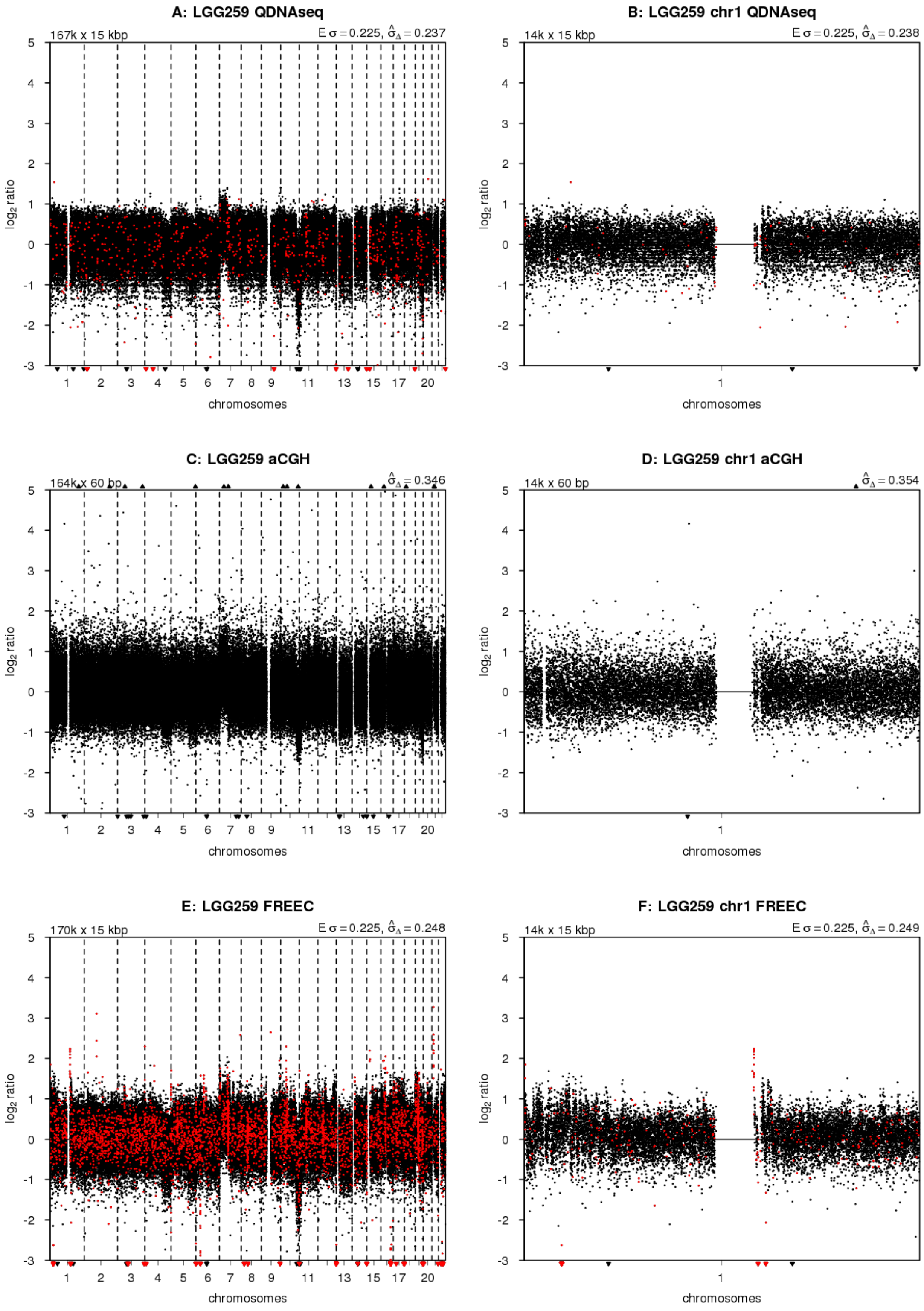

Figure S9: Comparisons to other methods (cont.)

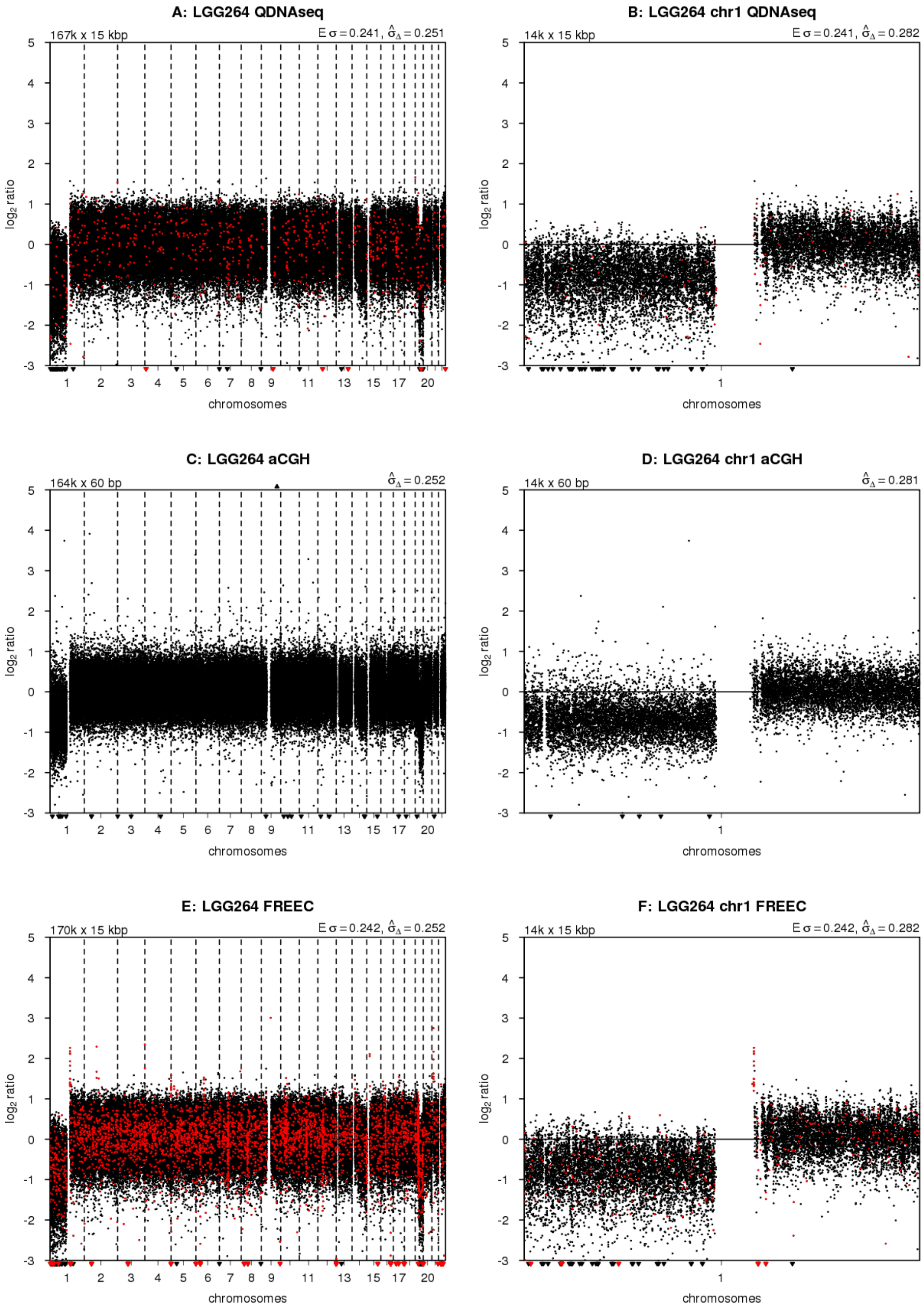

Figure S9: Comparisons to other methods (cont.)

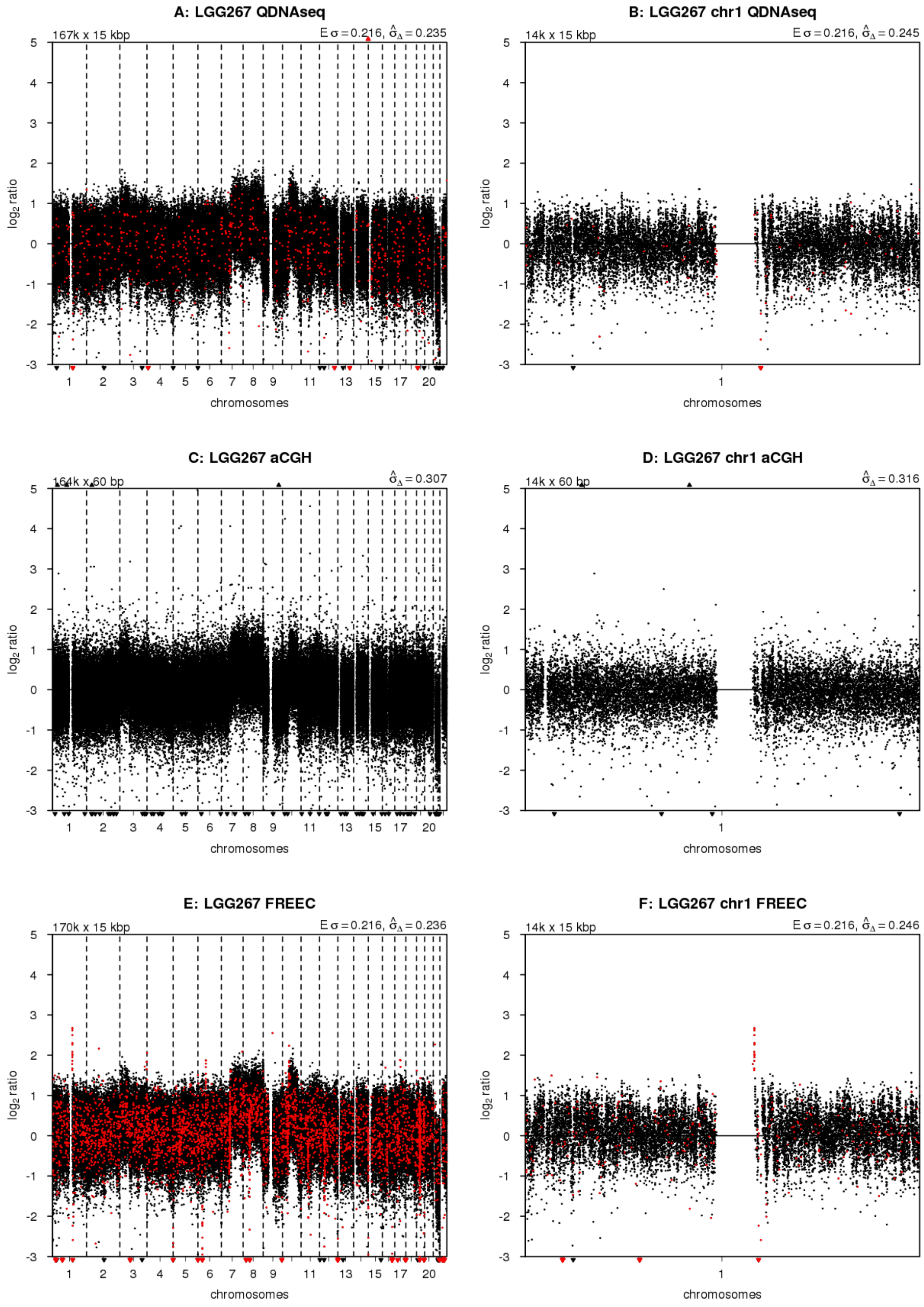

Figure S9: Comparisons to other methods (cont.)

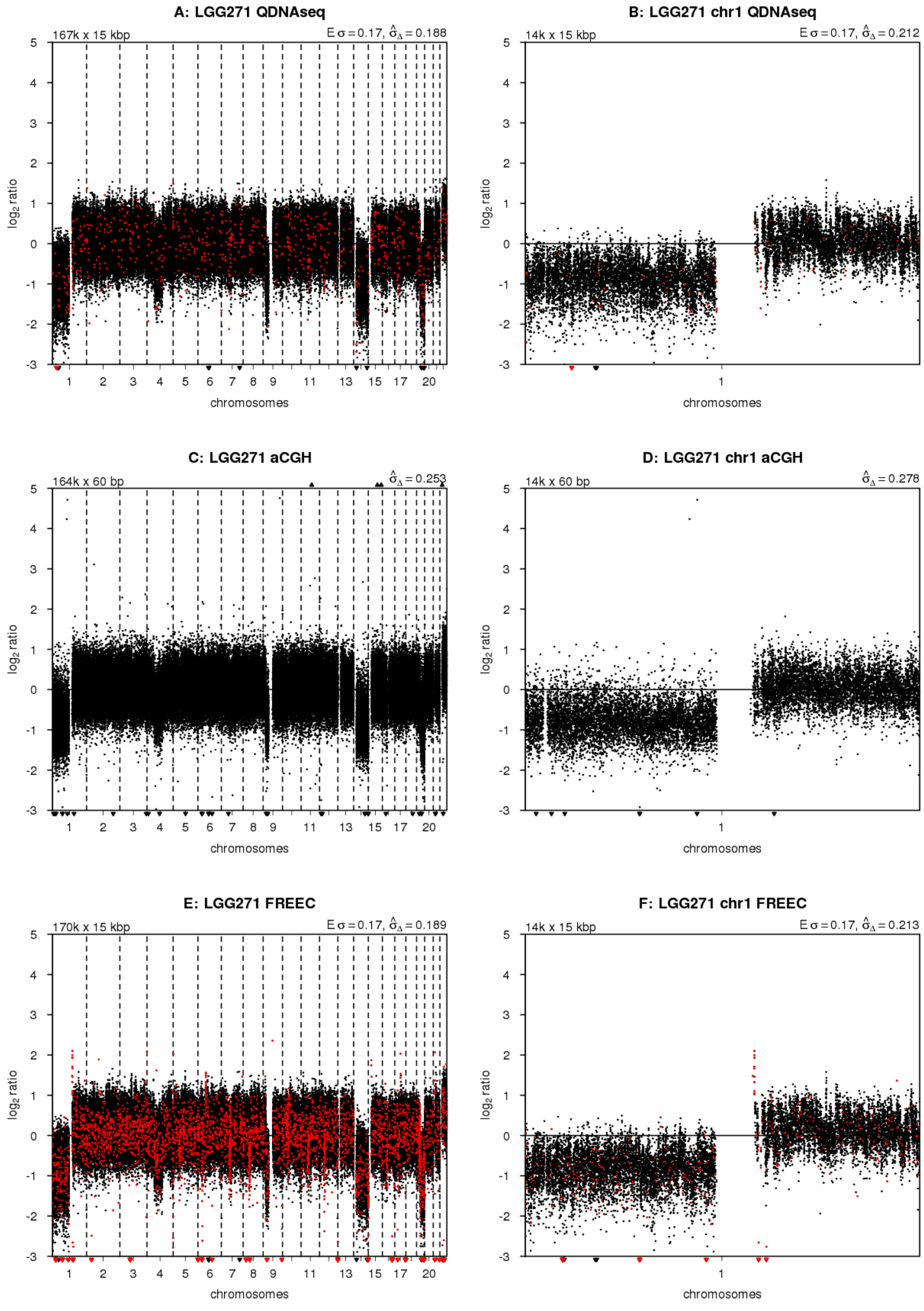

Figure S9: Comparisons to other methods (cont.)

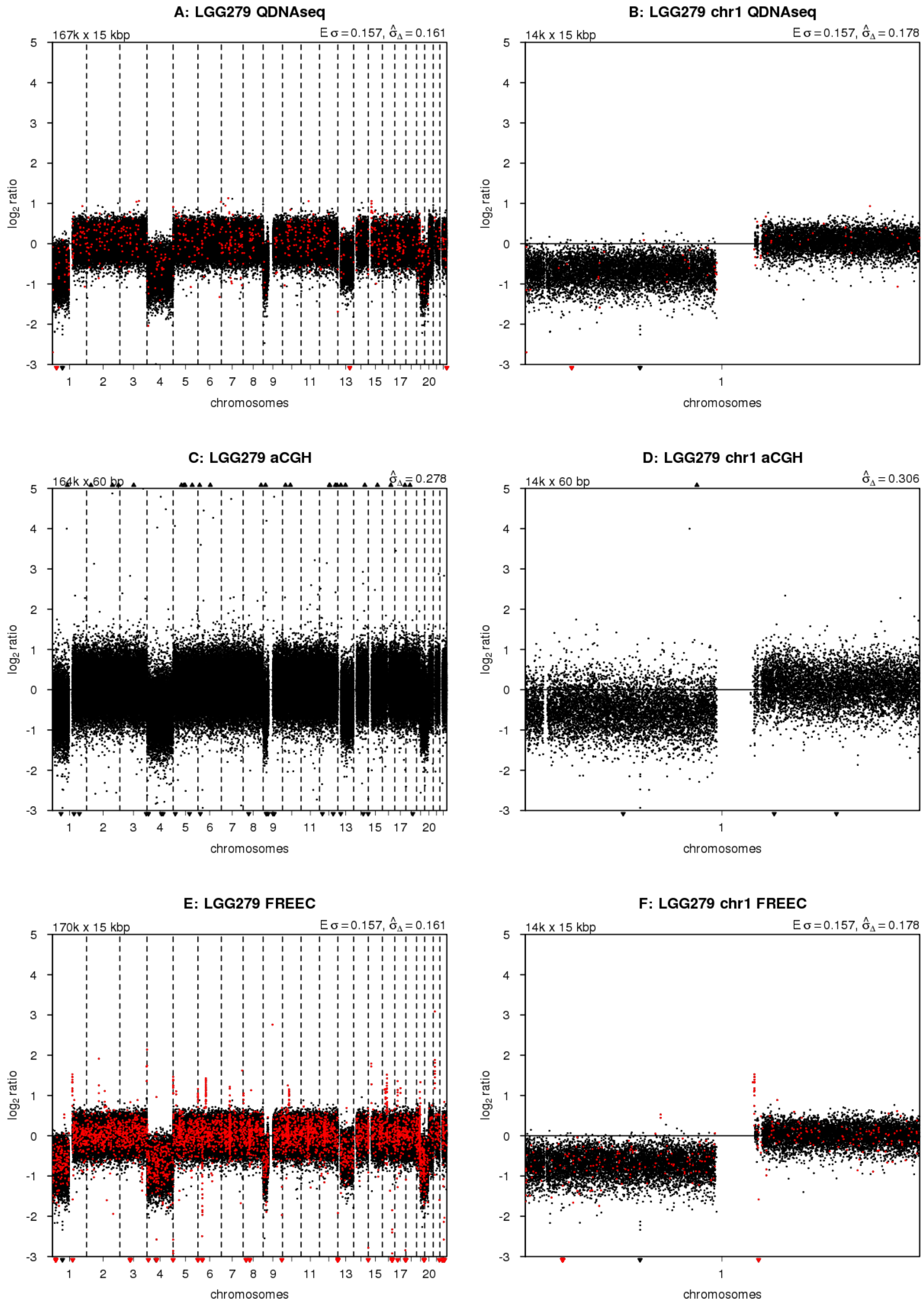

Figure S9: Comparisons to other methods (cont.)

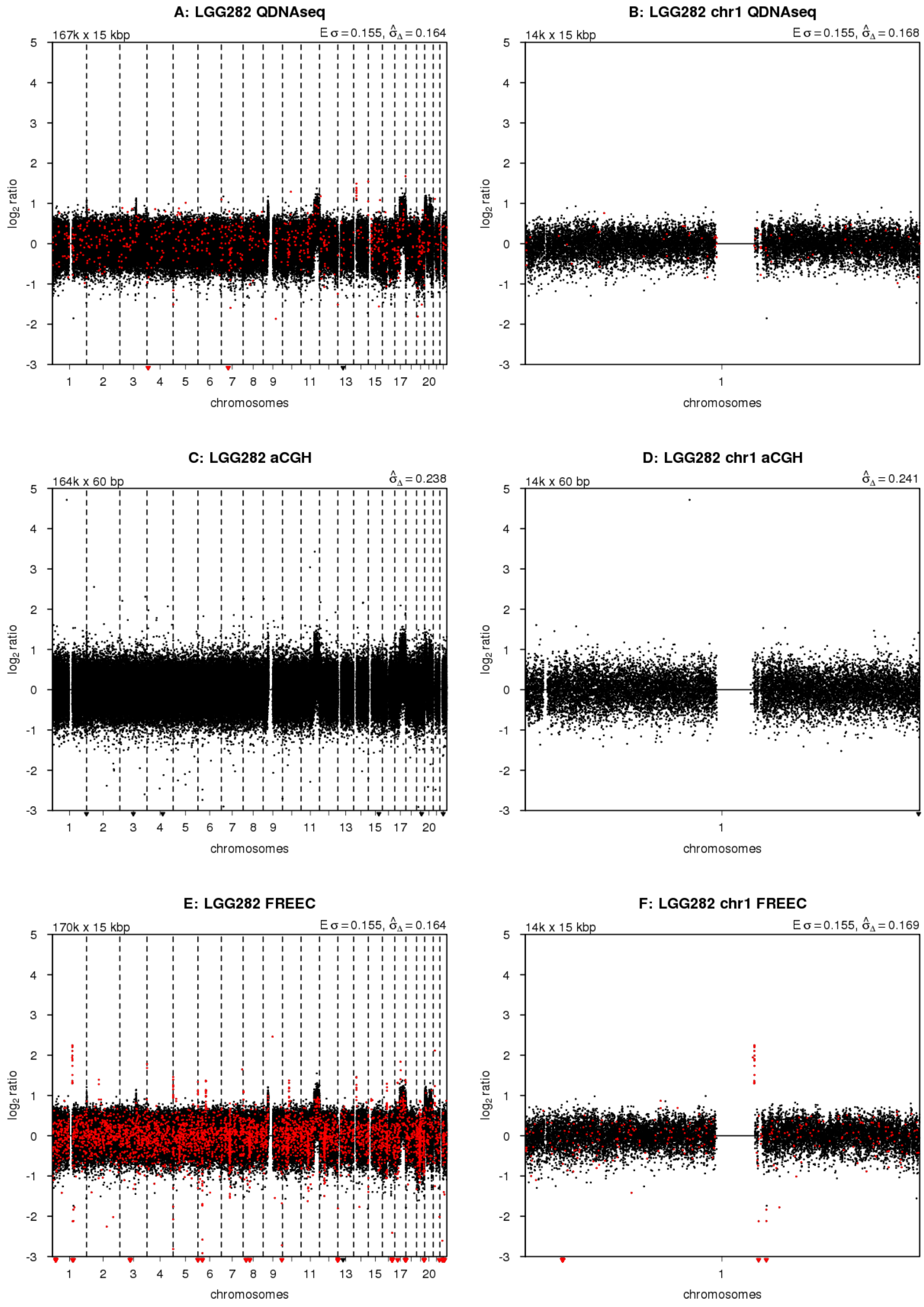

Figure S9: Comparisons to other methods (cont.)

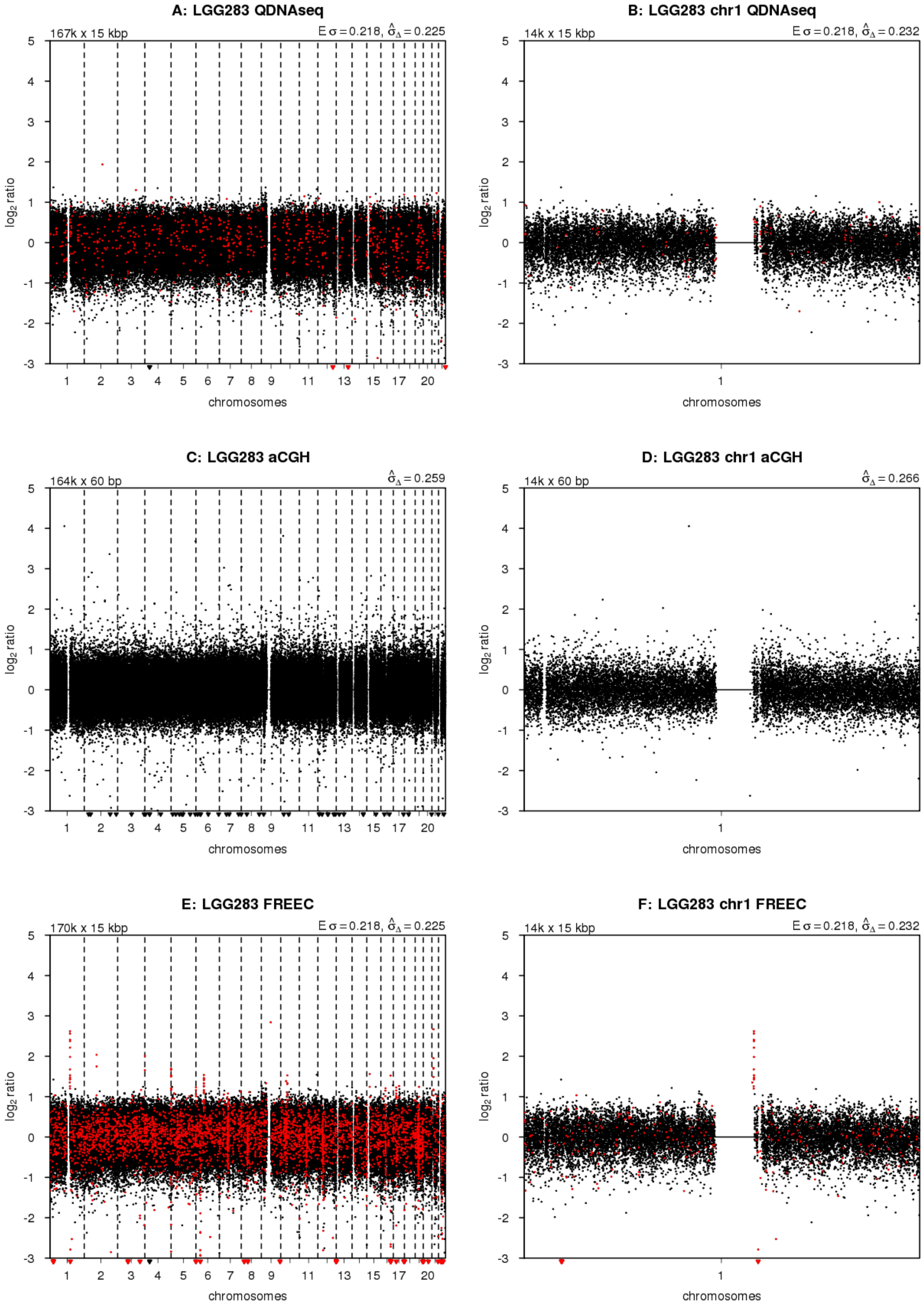

Figure S9: Comparisons to other methods (cont.)

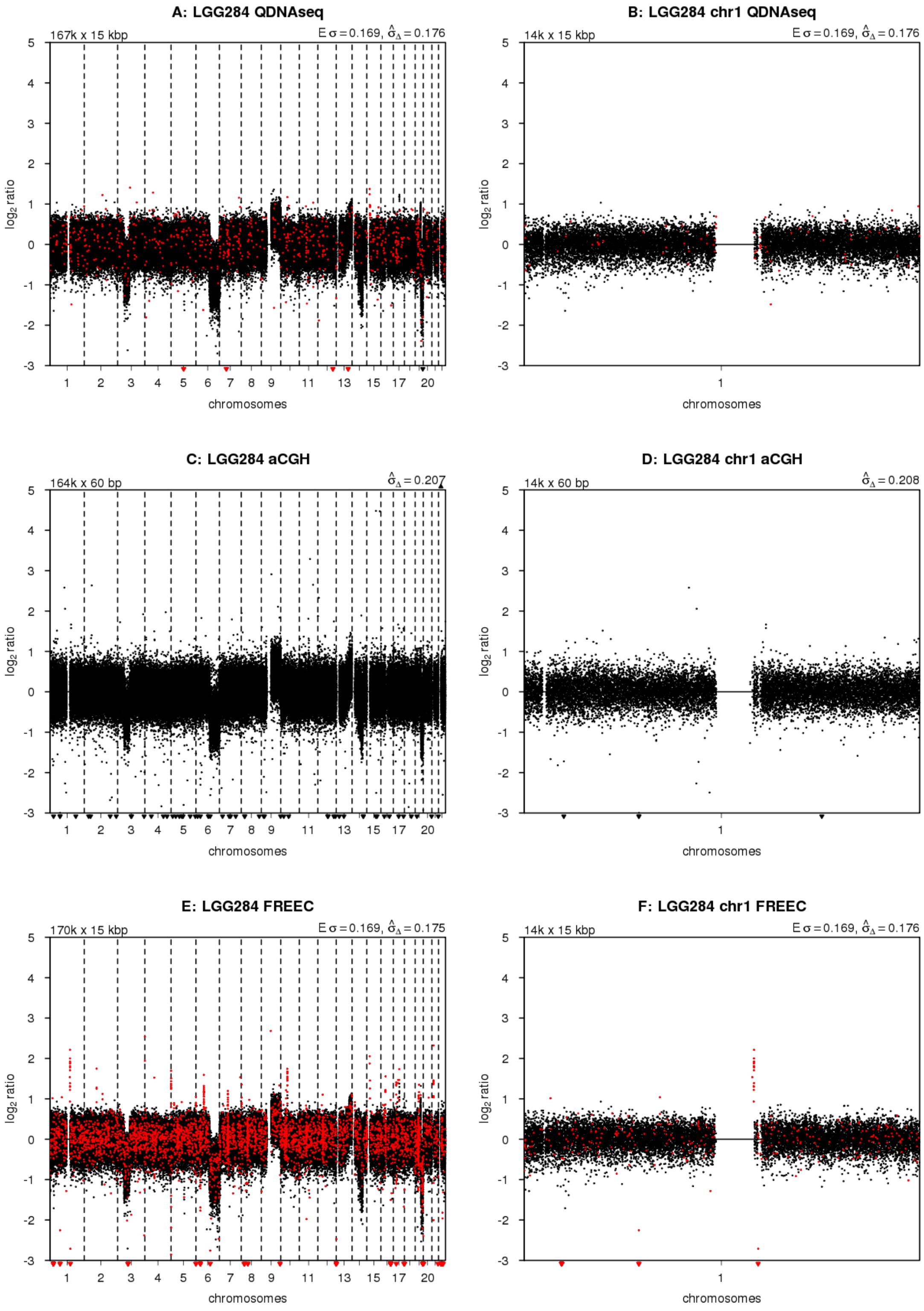

Figure S9: Comparisons to other methods (cont.)

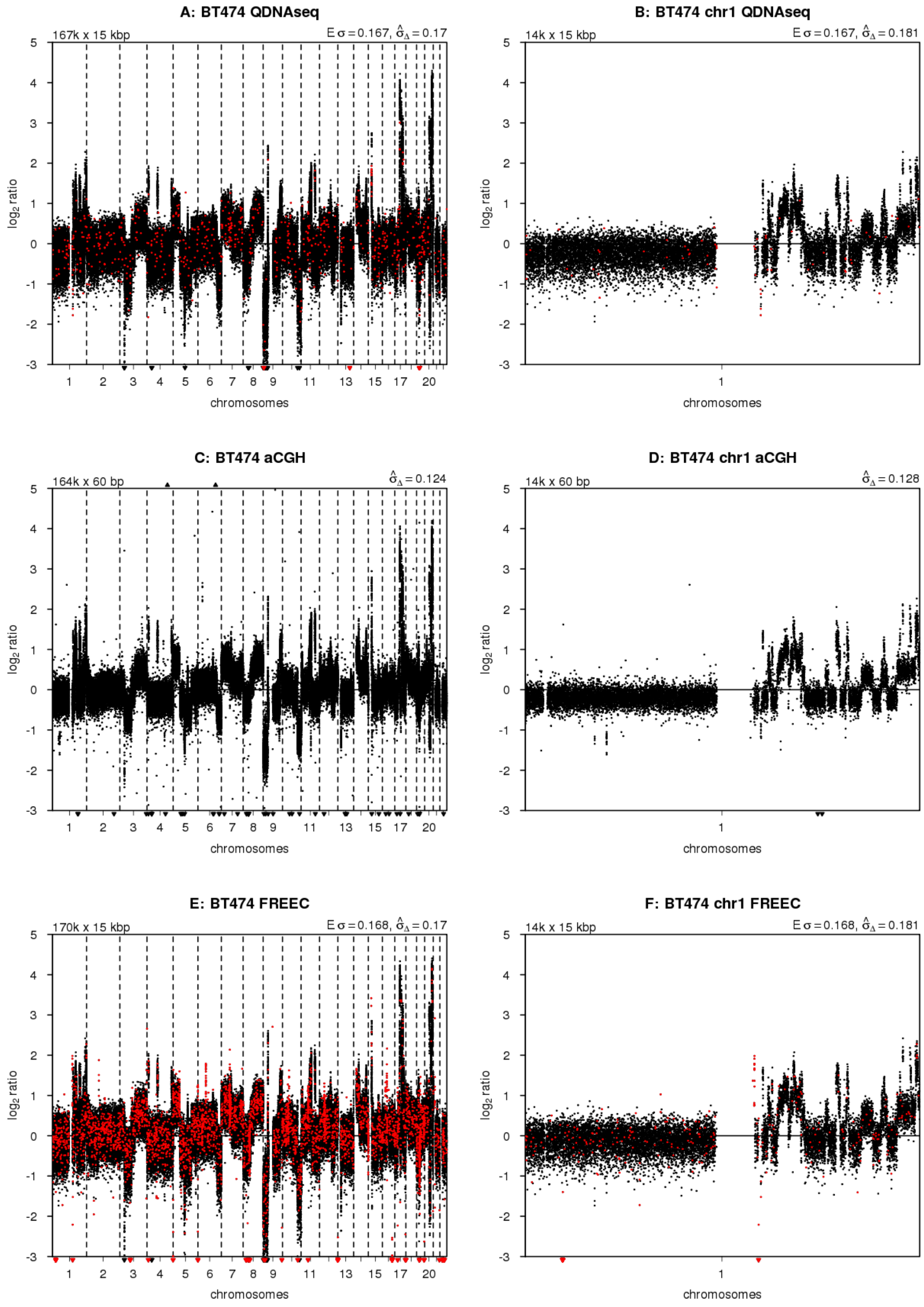

Figure S9: Comparisons to other methods (cont.)

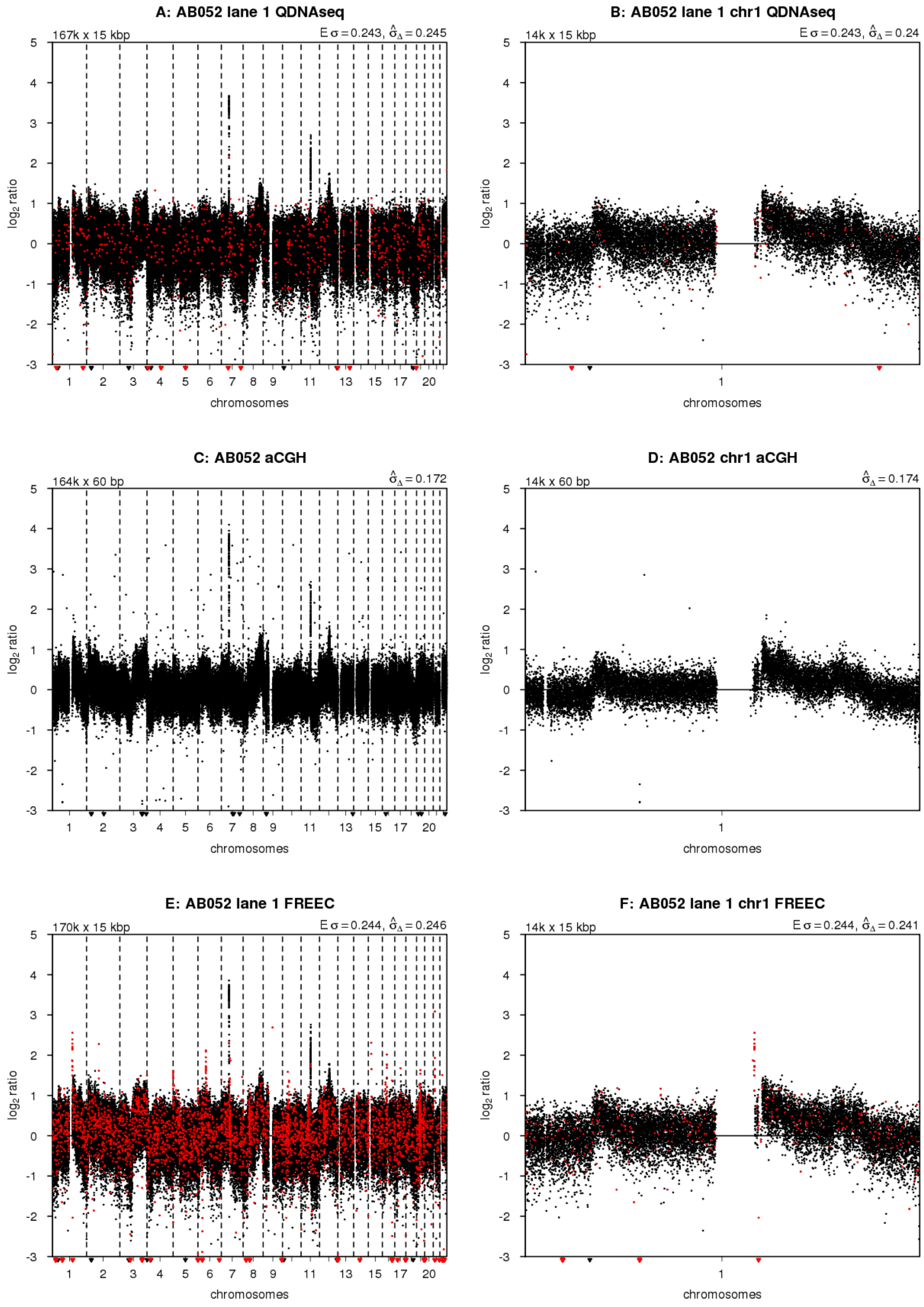

Figure S9: Comparisons to other methods (cont.)

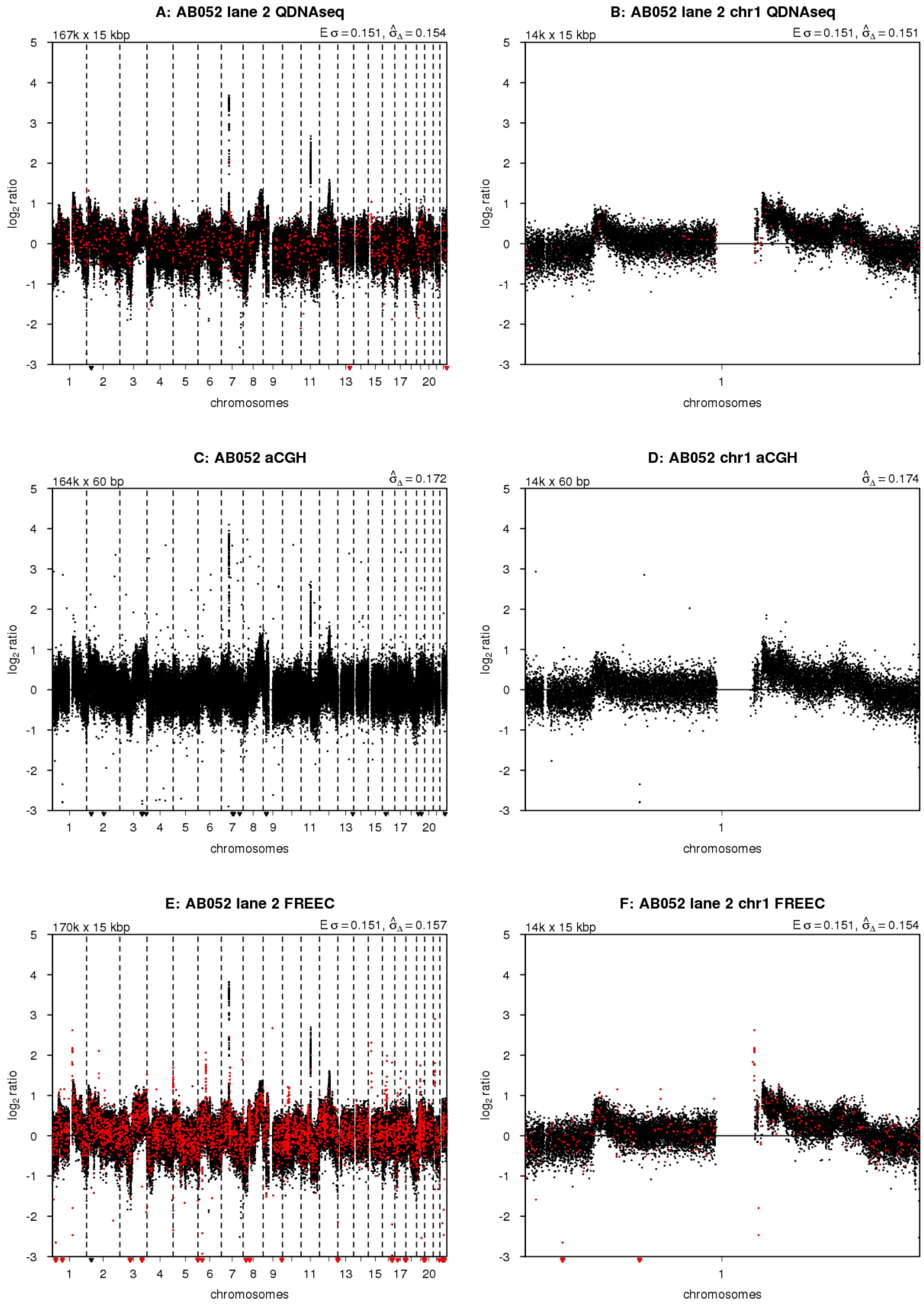

Figure S9: Comparisons to other methods (cont.)

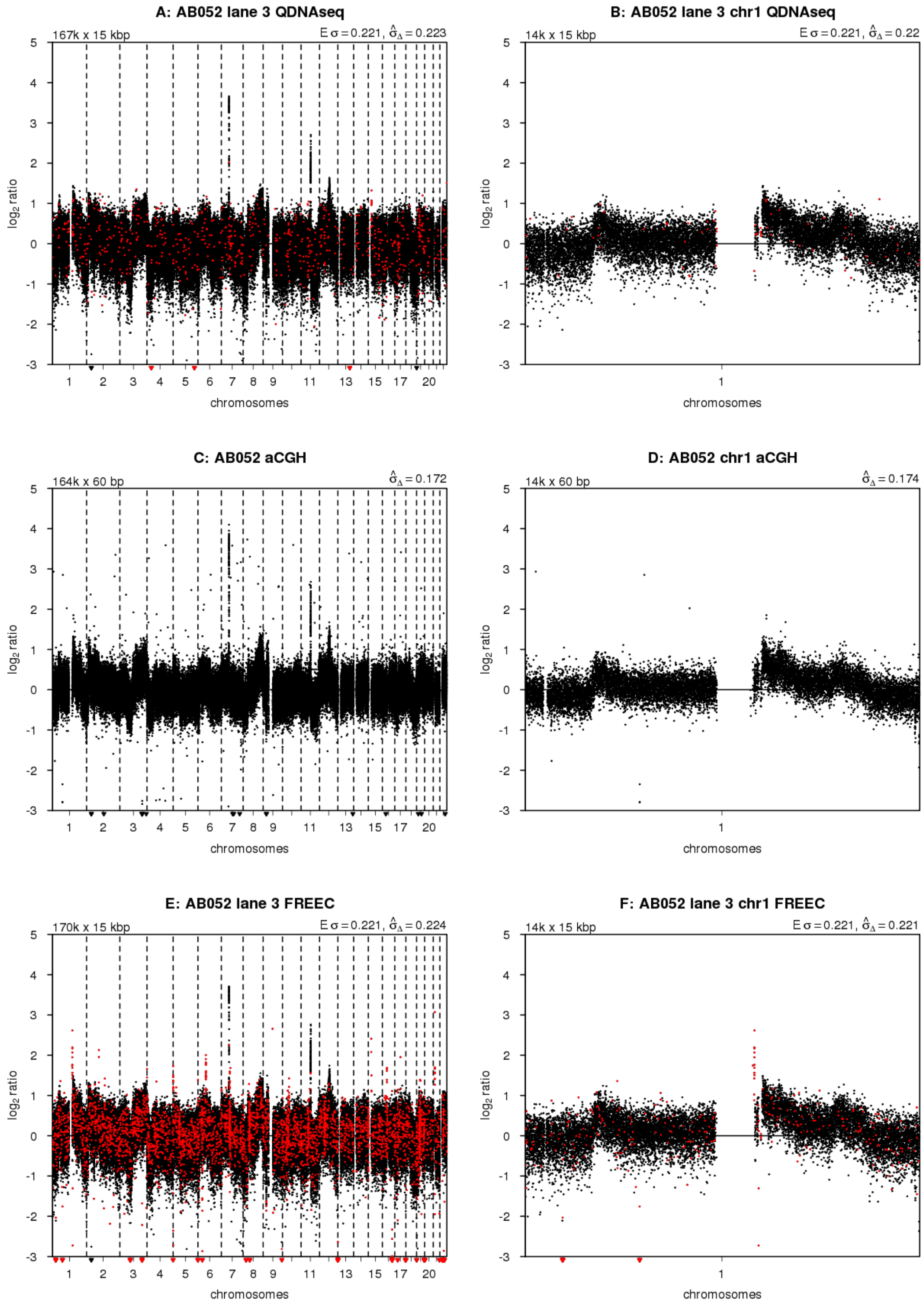

Figure S9: Comparisons to other methods (cont.)

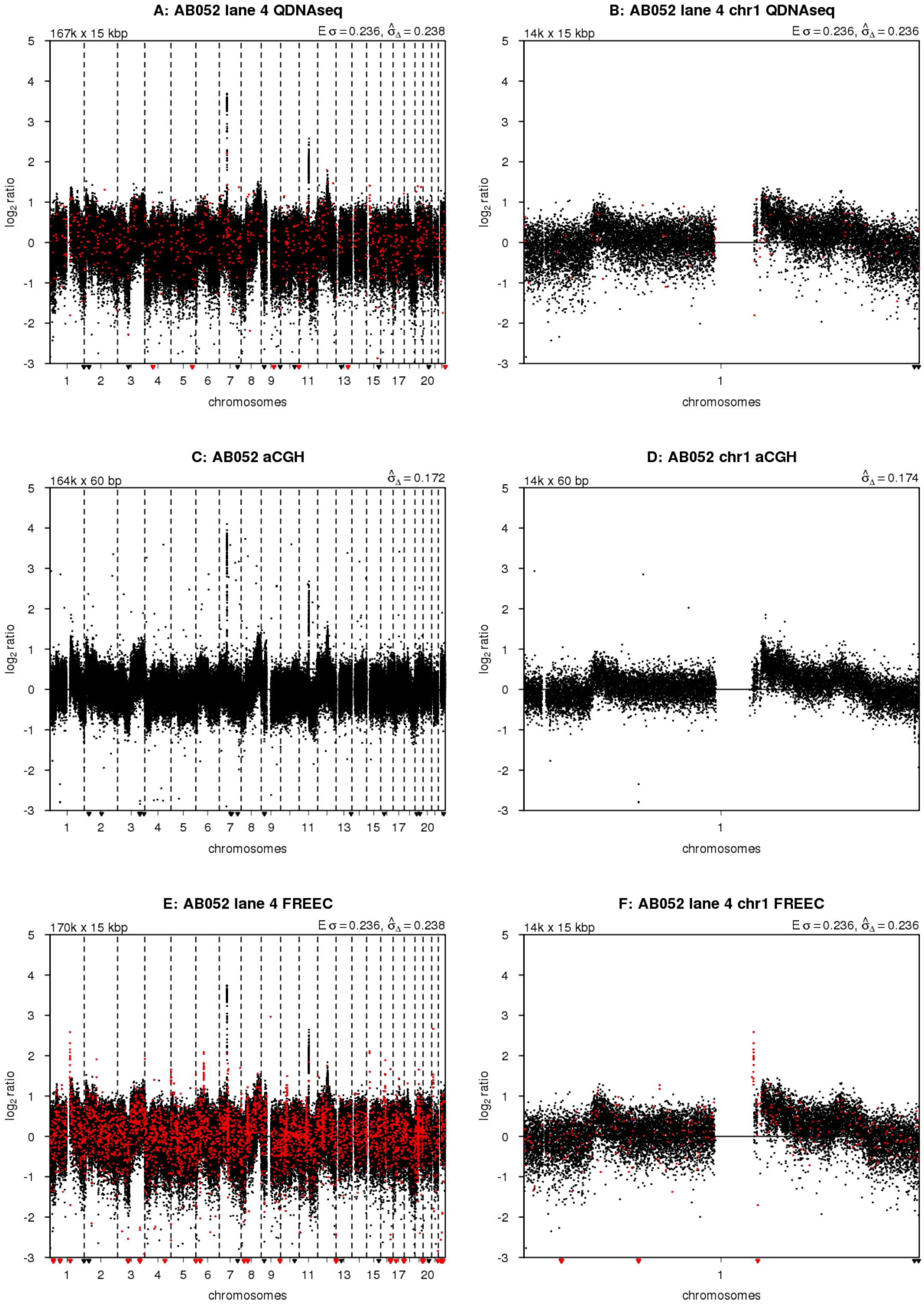

Figure S9: Comparisons to other methods (cont.)

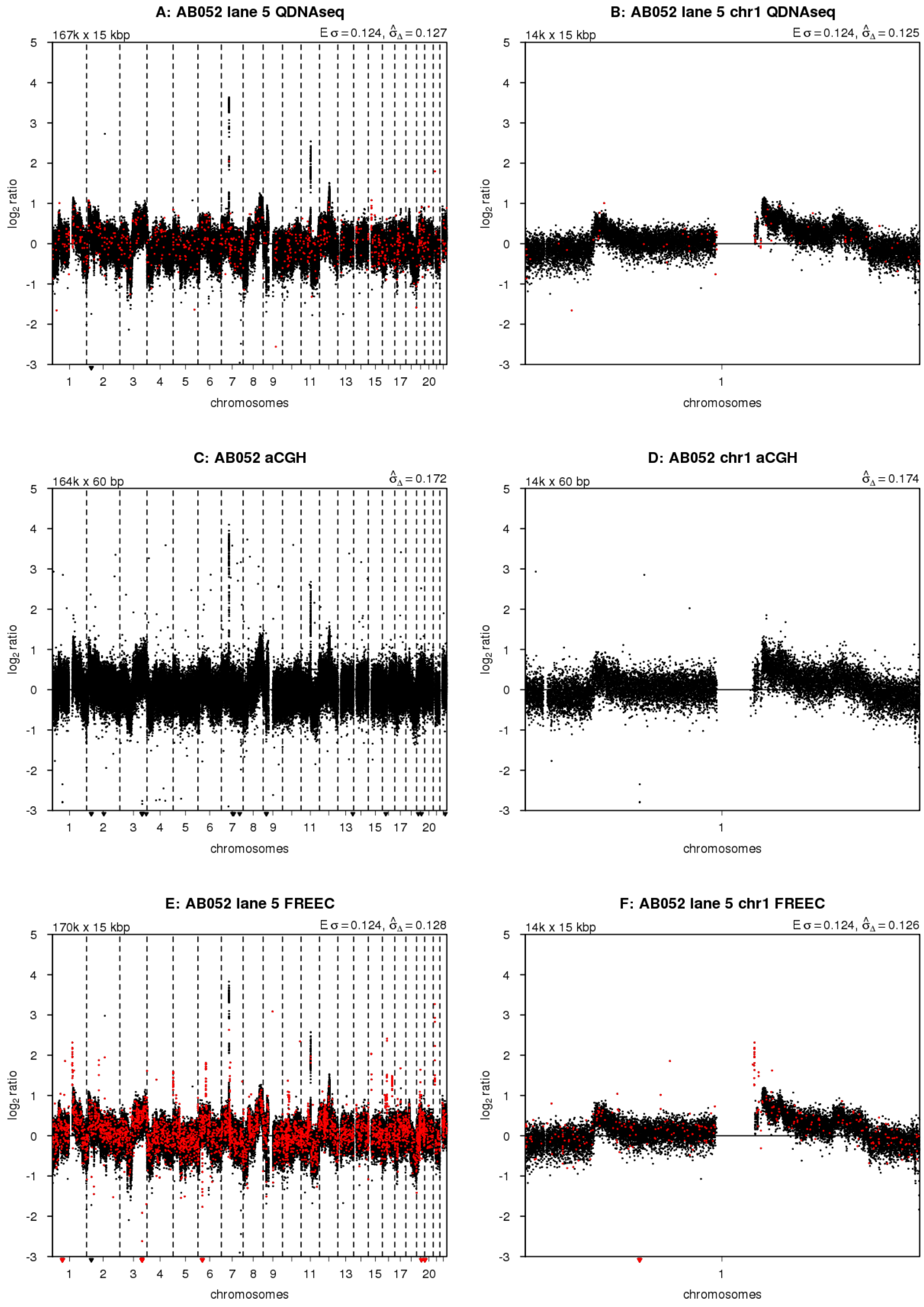

Figure S9: Comparisons to other methods (cont.)

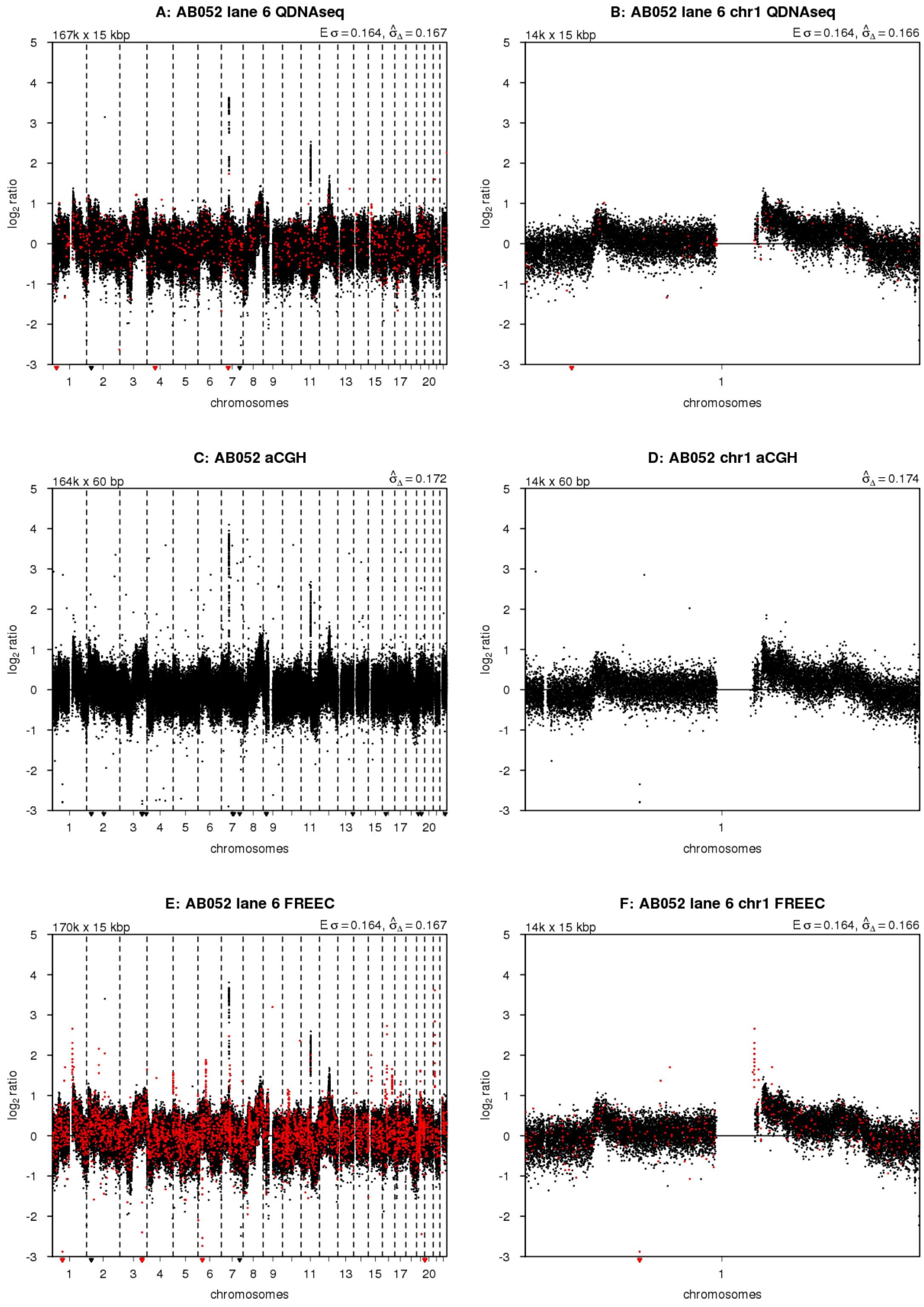

Figure S9: Comparisons to other methods (cont.)

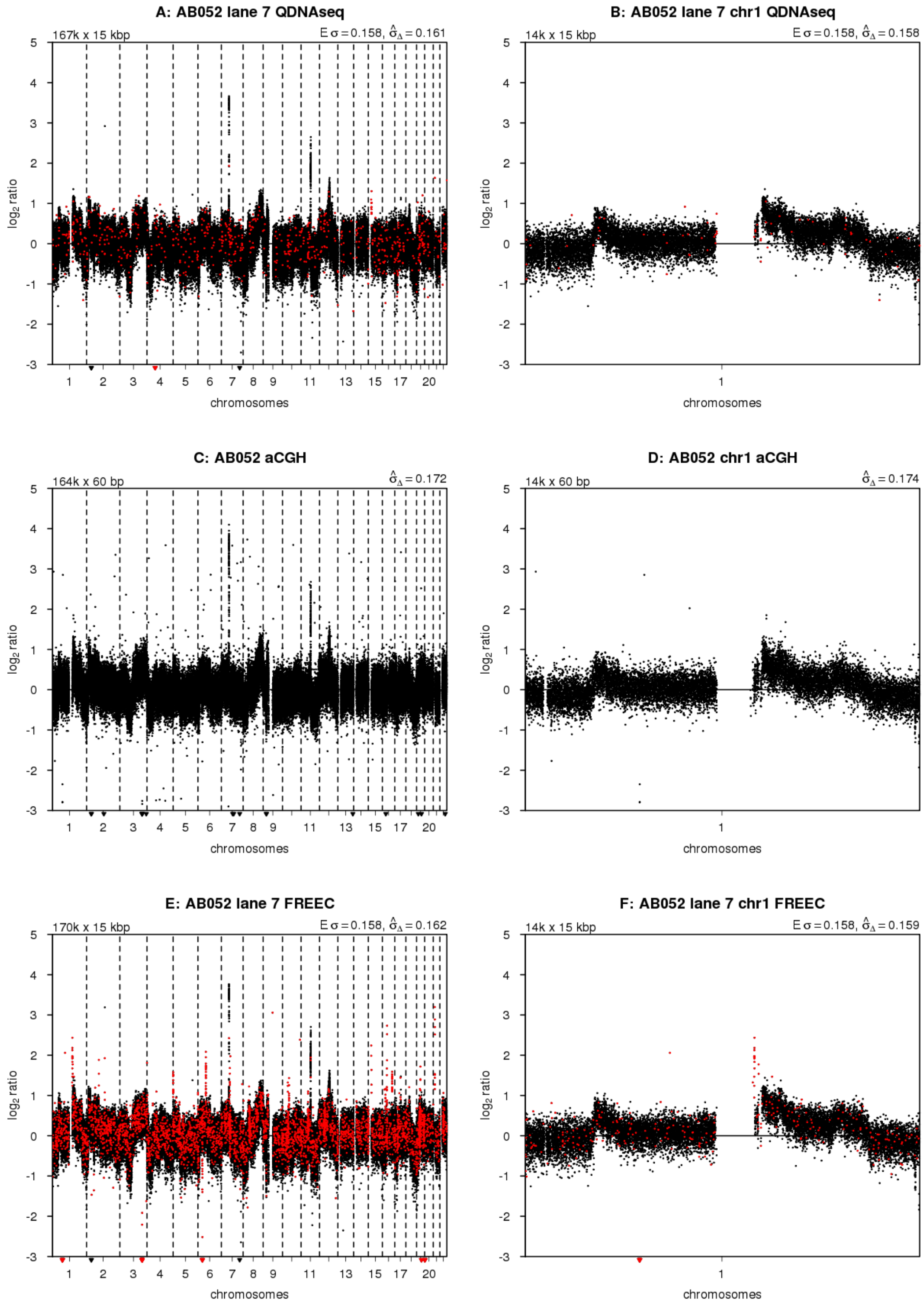

Figure S9: Comparisons to other methods (cont.)

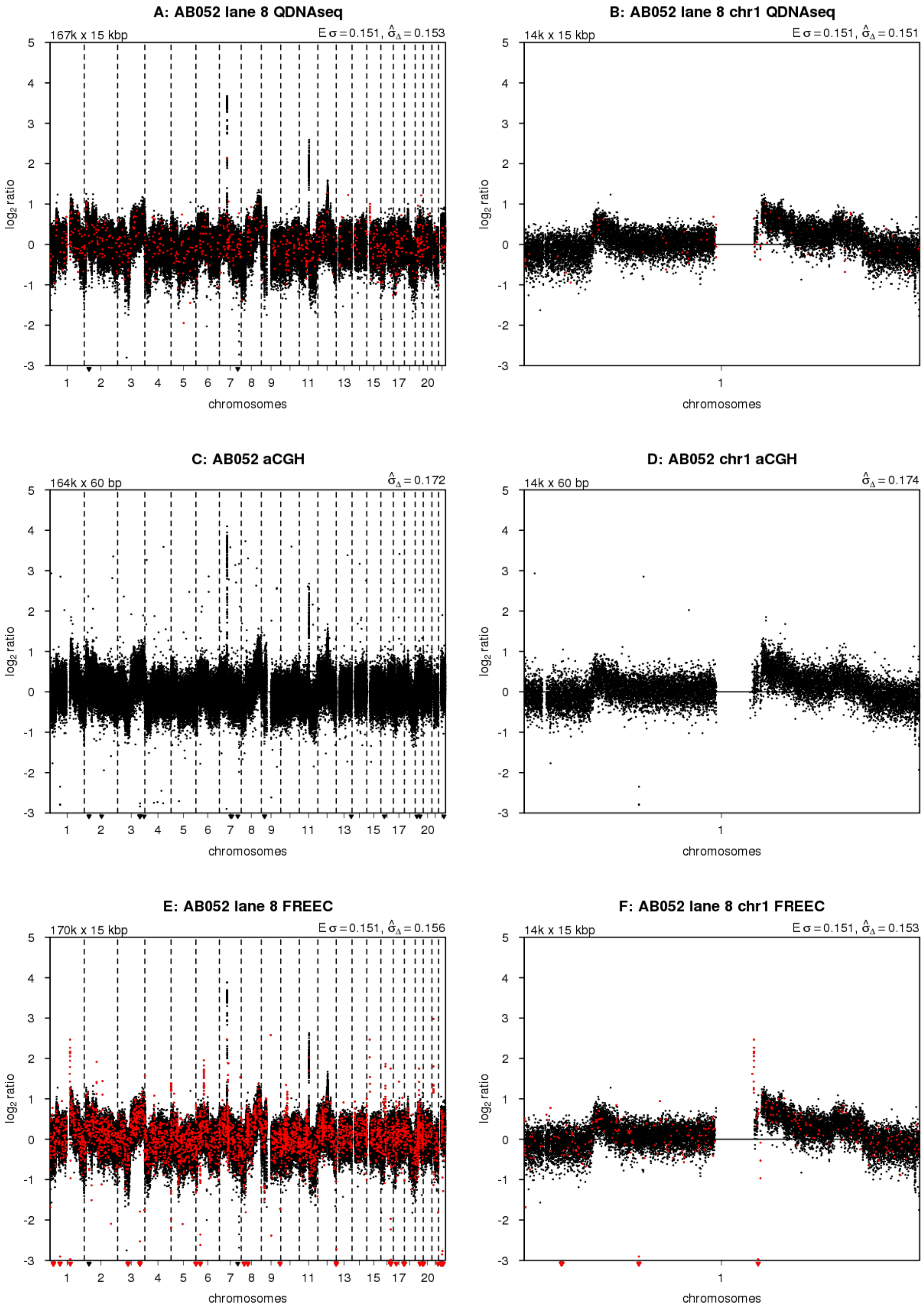

Figure S9: Comparisons to other methods (cont.)

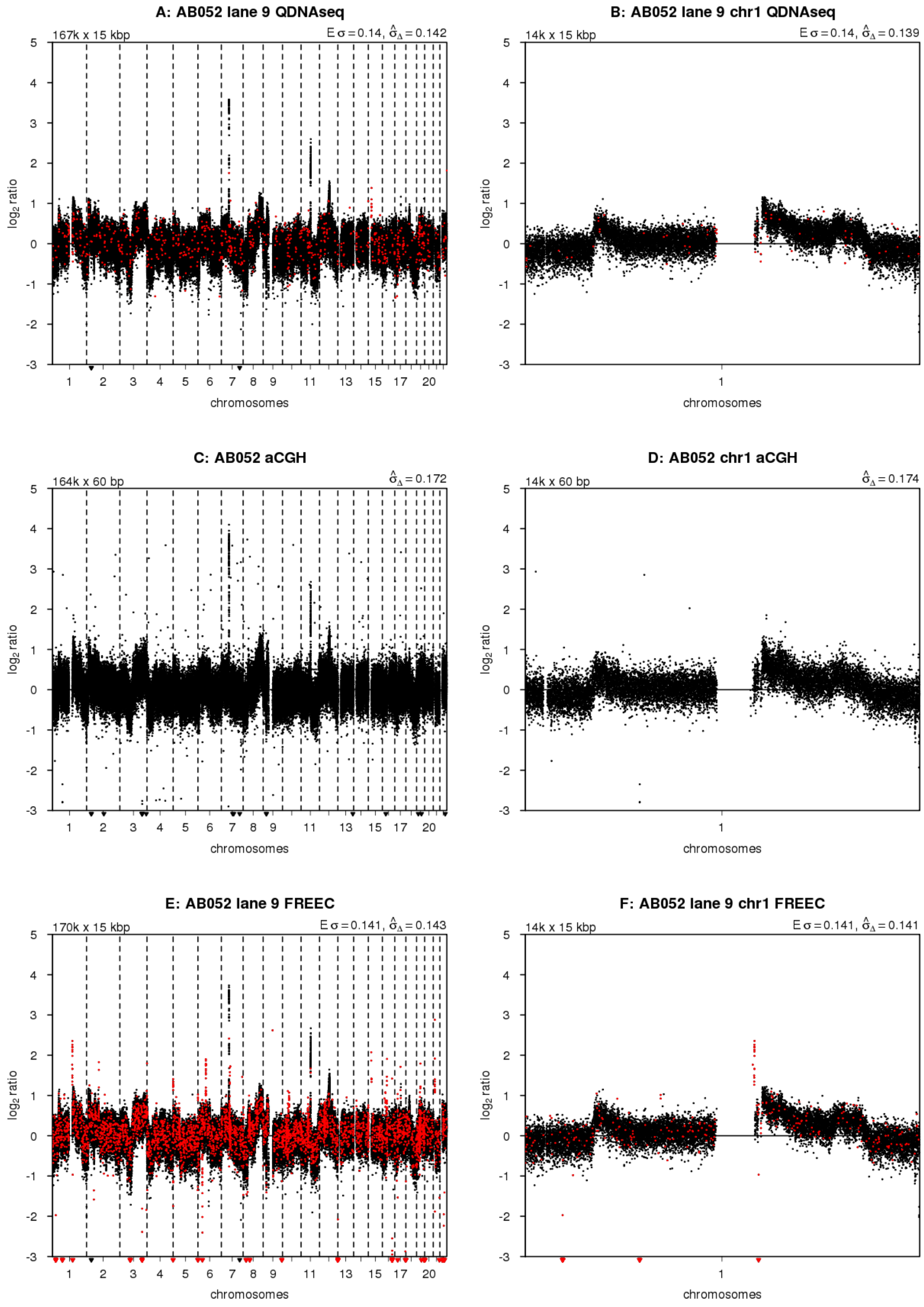

Figure S9: Comparisons to other methods (cont.)

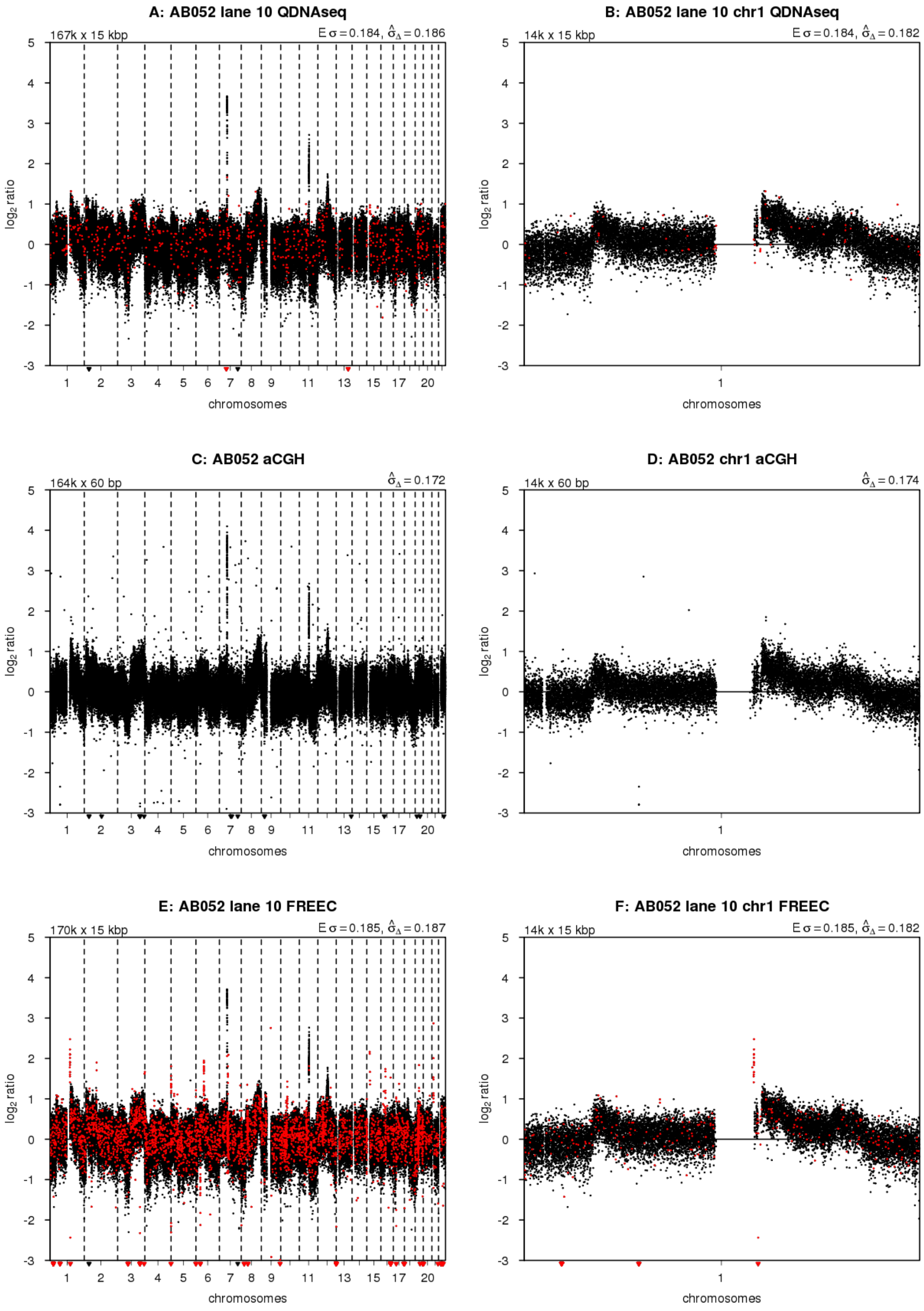

Figure S9: Comparisons to other methods (cont.)

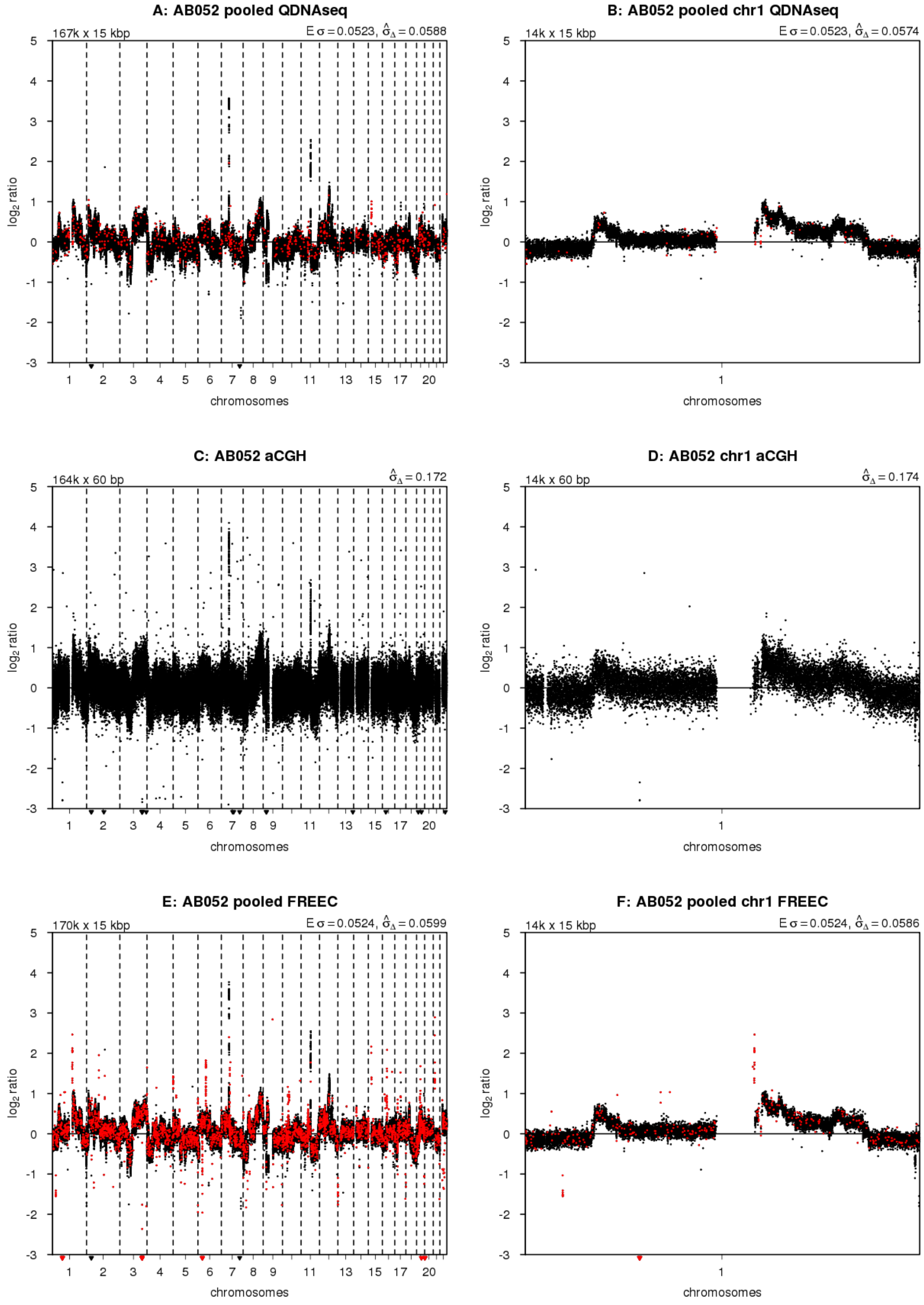

Figure S9: Comparisons to other methods (cont.)

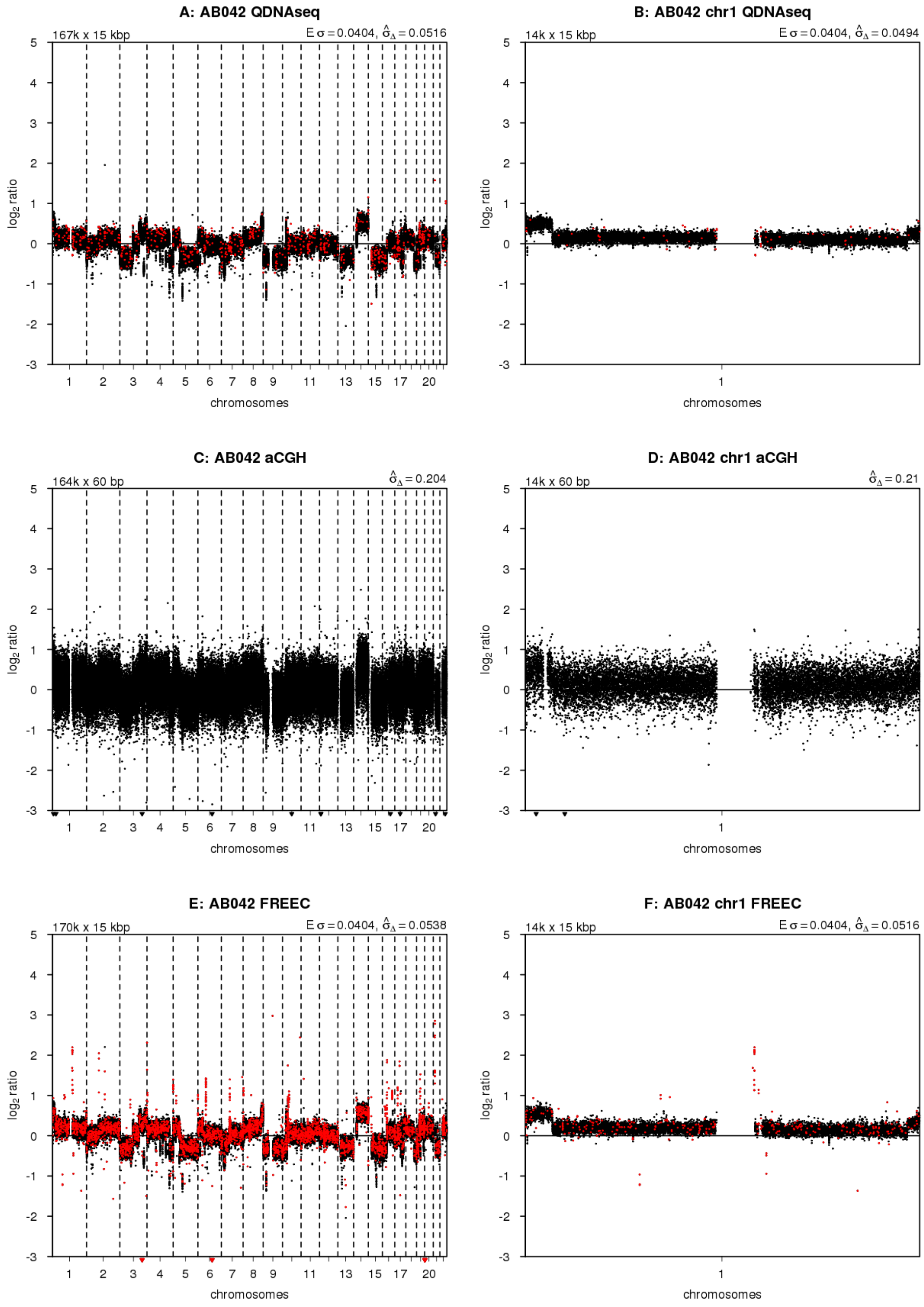

Figure S9: Comparisons to other methods (cont.)
